# 1,5-Phosphonium betaines from *N*-triflylpropiolamides, triphenylphosphane, and active methylene compounds

**DOI:** 10.3762/bjoc.15.253

**Published:** 2019-11-01

**Authors:** Vito A Fiore, Chiara Freisler, Gerhard Maas

**Affiliations:** 1 Institute of Organic Chemistry I, Ulm University, Albert-Einstein-Allee 11, D-89081 Ulm, Germany

**Keywords:** betaines, multi-component reactions, *N*-triflylamides, phospha-Michael reaction, propiolamides

## Abstract

*N*-Phenyl-*N*-(trifluoromethylsulfonyl)propiolamides react with triphenylphosphane in the presence of various active methylene compounds CH_2_XY in a 1:1:1 molar ratio to furnish 1-phosphonium-5-oxabetaines, Ph_3_P^+^–C(R)=CH–C(O^–^)=CXY. These betaines are formed preferentially, but not exclusively, as *E*-diastereoisomers with respect to the vinylic double bond. In some cases, separation of the two diastereoisomers was achieved by fractionating crystallization. Structure determination by X-ray diffraction analysis revealed marked conformational differences around the CH–C(O^–^) single bond of *E* and *Z*-isomers and extended charge delocalization in the anionic part.

## Introduction

Beside the well-known phosphonium ylides (Wittig ylides, methylenephosphoranes), various other types of zwitterions containing a tetravalent phosphonium moiety (phosphonium betaines) exist. They are often considered as reaction intermediates, but reports on their isolation and structural characterization are still less common. A very convenient access to such betaines is provided by the nucleophilic addition of tertiary phosphanes to electron-deficient alkenes, alkynes and allenes (phospha-Michael addition), which generates the betaines as reactive intermediates that can be transformed intra- or intermolecularly into a wide array of acyclic, carbocyclic and heterocyclic structures. In these processes, the tertiary phosphanes can act as nucleophilic organocatalysts (for reviews, see [1–4] or be incorporated in the (pre-)final products, typically as alkylidene phosphoranes which in turn may undergo Wittig olefination with elimination of Ph_3_P=O [1,5]. Recently, a study of the tributylphosphane-catalyzed reaction of ethyl 2-butynoate and ethanol by in-situ ESIMS and NMR techniques has been published [6], and mechanistic aspects of trialkylphosphane-catalyzed reactions of acetylenic ketones and esters with pinacolborane have been discussed [7–8].

**Scheme 1:**
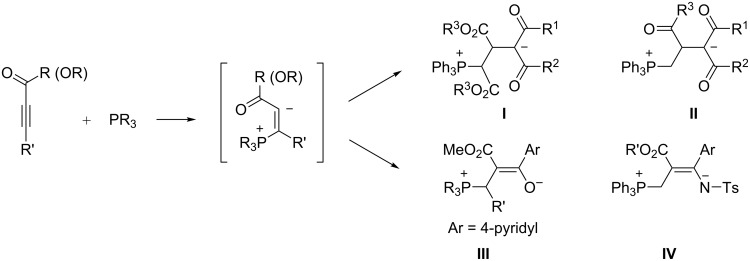
Stable betaines **I**–**IV**.

The Michael addition of PPh_3_ at acetylenic carbonyl compounds generates phosphonium/vinyl anion intermediates which have been trapped with CH-, NH-, OH- and SH-acids [5]. In this manner, the 1:1:1 reaction of triphenylphosphane with alkyl propiolates [9–10] or dialkyl acetylenedicarboxylates [11] and strong CH-acids, such as Meldrum’s acid, 1,3-dimethylbarbituric acid, dimedone and indane-1,3-dione, leads to stable 1,4-diionic phosphonium betaines **I** or **II** (Scheme 1) in high yields. An analogous reaction has been achieved with 1,1,1,5,5,5-hexafluoropentane-2,4-dione as the active methylene component [12]. Some of these three-component reactions were even performed in aqueous media [13]. In mechanistic terms, the initially formed 1,3-betaine is *C*-protonated by the CH-acid, the anion of which then combines with the vinylphosphonium ion, and a proton transfer finally yields the 1,4-betaine. A kinetic study of the PPh_3_/DMAD/Meldrum’s acid reaction by spectrophotometric and stopped-flow methods has been published [14]. The initial 1,3-betaine has also been trapped with other electrophiles. The stable 1,4-betaines betaines **III** (PR_3_ = alkyl-substituted phosphane, but not PPh_3_) have been obtained from isonicotinaldehyde [15], and betaines **IV** from aryl tosylimines [16]. For the stability of betaines **III**, the nature of the aryl substituent is critical; with the combination Ph_3_P/DMAD/EWG-substituted benzaldehydes, cyclization leading to butenolides occurs easily [17].

**Scheme 2:**
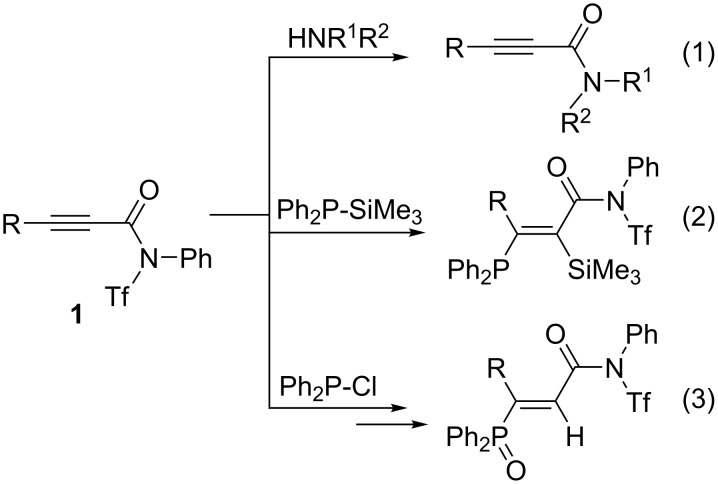
Reactions of *N*-triflyl-propiolamides **1** with *N*- and *P*-nucleophiles. Tf = SO_2_CF_3_.

In contrast to acetylenic esters and ketones, acetylenic carboxamides have rarely been exposed to nucleophilic phosphanes in organocatalytic or stoichiometric reactions. Recently, we have reported on the synthesis and reactivity of *N*-triflylpropiolamides **1** (Scheme 2). They turned out to be excellent substrates for transamidation reactions (reaction 1) [18], whereas tertiary phosphanes Ph_2_P–X (X = SiMe_3_, Cl) underwent Michael addition leading to silylphosphanylation (reaction 2) or hydrophosphorylation (reaction 3) of the C–C-triple bond, respectively [19]. We report now on three-component reactions of alkynes **1** with triphenylphosphane and active methylene compounds, leading to phosphonium betaines which are structurally different from those shown in Scheme 1.

## Results and Discussion

### Synthesis of betaines **3**

**Scheme 3:**
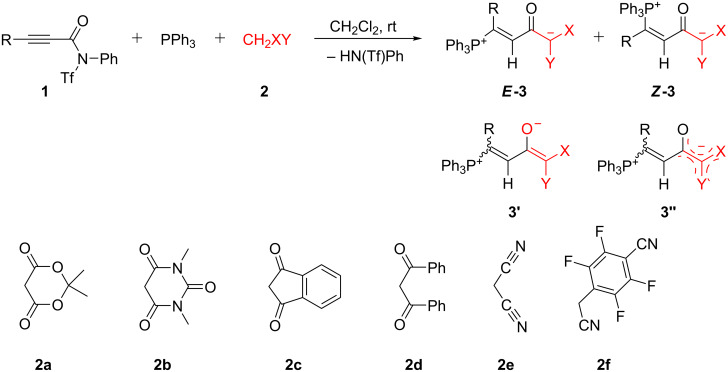
Synthesis of betaines **3** by a three-component reaction. *N*-triflylpropiolamides **1**: R = Ph (**a**), 4-Cl-C_6_H_4_ (**b**), *t*-Bu (**c**), *n*-Bu (**d**), cyclopropyl (**e**), SiMe_3_ (**f**). See Figure 1 for products.

**Figure 1:**
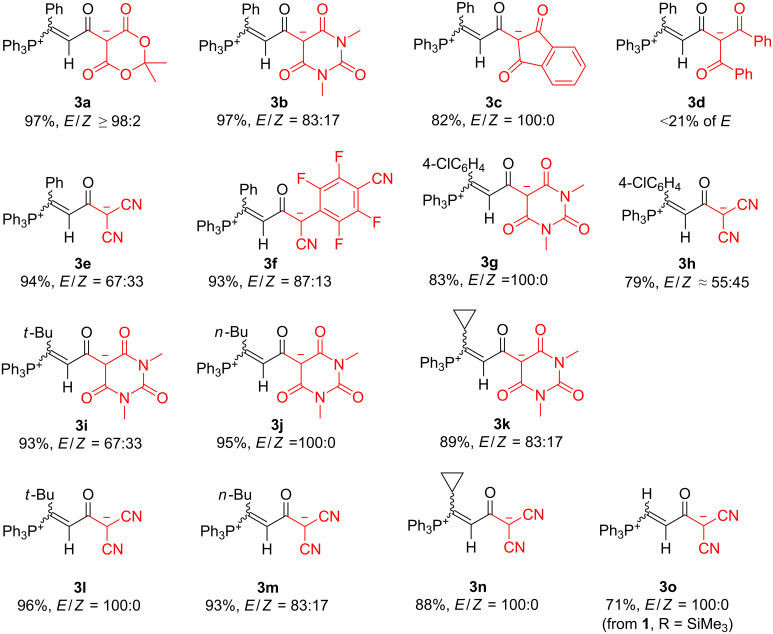
Phosphonium betaines **3** prepared. An *E*:*Z* ratio of 100:0 means that only the *E*-isomer was observed in the NMR spectra.

The combination of equimolar amounts of triphenylphosphane and *N*-triflyl-propiolanilide **1a** in dichloromethane yielded a product mixture, from which only *N*-phenyltriflylamide (HN(Ph)Tf; ^19^F NMR: δ −76.5 ppm rel. to C_6_F_6_) could be extracted, the remainder being undefined oligomeric/polymeric material. However, when an *N*-triflylpropiolamide **1a**–**e** (1.03 molar equivalents) was added to a solution of PPh_3_ and an active methylene compound **2a**–**c,e,f**, a 1:1:1 three-component reaction occurred smoothly within 0.5–6 hours and yielded the phosphonium-1,5-betaines **3a**–**o** in mostly high yields (Scheme 3 and Figure 1). Meldrum’s acid (**2a**), *N*,*N’*-dimethylbarbituric acid (**2b**), 1,3-indanedione (**2c**), malononitrile (**2e**), 4-cyanomethyl-2,3,5,6-tetrafluorobenzonitrile (**2f**) were successfully applied as active methylene compounds; all of them have p*K*_a_ values between 4.70 (**2b**, in dioxane/H_2_O 3:1 [20]) and ≈15.80, reported for 2-(pentafluorophenyl)acetonitrile in DMSO [21], the value for **2f** should be similar). On the other hand, the combination of *N*,3-diphenyl-*N*-triflylpropiolamide (**1a**) with PPh_3_ and dibenzoylmethane (**2d**) was far from clean and furnished in low yield betaine *E*-**3d**, which could not be purified by repeated recrystallization. An undefined polymeric material rather than betaines was obtained from 1,1,1,5,5,5-hexafluoropropane-2,4-dione (p*K*_a_ 2.30 in DMSO) and dimedone (p*K*_a_ 11.42 in DMSO).

**Scheme 4:**
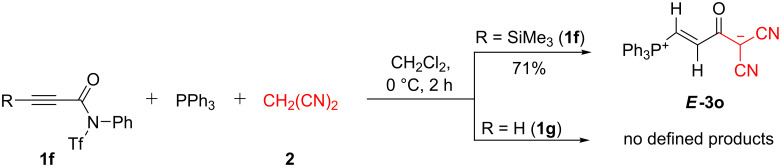
Unexpected synthesis of *E*-**3o**.

Figure 1 also shows that 3-aryl- as well as various 3-alkyl-substituted propiolamides **1** furnish betaines **3** in high yields. A separate case was observed for 3-trimethylsilylpropiolamide **1f**, which reacted with PPh_3_ and malononitrile to furnish the desilylated betaine *E*-**3o**; the required proton probably stems from the adventitious presence of water (Scheme 4). Notably, **3o** could not be obtained directly from the 3-H substituted *N*-triflylpropiolamide **1g**.

Some betaines **3** were isolated solely as *E*-diastereoisomers, others as *E*/*Z* mixtures. In some cases, the two diastereoisomers were separated by fractionating crystallization from a mixed-solvent solution. In the case of **3a**, a trace amount (≈2%) of the *Z*-isomer was detected in the reaction mixture by ^1^H NMR (the chemical shift of the olefinic proton being characteristic), but got lost during work-up. An inspection of Figure 1 reveals no obvious reason for the obtained *E*/*Z* ratios. Beside characteristic spectroscopic data (vide infra), the diastereoisomeric betaines can also be distinguished by their colors: the isolated *E*-isomers are usually colorless, while the *Z*-isomers have a yellow shade. As an exception, *E*-**3f** was obtained as dark red needle-shaped crystals, in contrast to pale yellow blocks of *Z*-**3f**. All betaines could be stored unchanged at least for a year under a dry atmosphere. They appear to be somewhat hygroscopic, as the broad O–H absorption bands in their IR spectra (≈3400–3500 cm^−1^) and the presence of hydrogen-bonded water molecules in the crystal structure of *E*-**3e** (vide infra) indicate.

**Scheme 5:**
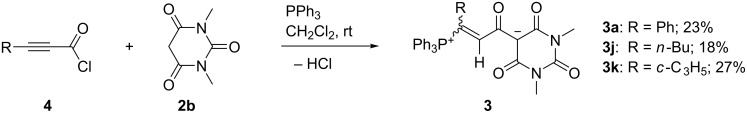
Betaines **3** from propiolic acid chlorides.

The formation of betaines **3** includes the elimination of the *N*-phenyltriflamide anion, which is known as an excellent leaving group in transamidation reactions with nucleophilic amines [18,22]. Therefore, it was interesting to know the performance of propiolic acid chlorides in the same betaine syntheses. We found that acid chlorides **4** indeed underwent the three-component reaction with *N*,*N*’-dimethylbarbituric acid and furnished betaines **3b**, **3j** and **3k**, respectively, with about the same *E*/*Z* ratios as in Figure 1 (Scheme 5). However, the products were obtained in low yield, because unidentified side-reactions occurred and purification was cumbersome. Thus, propiolic acid chlorides are not well suited for these three-component reactions.

**Scheme 6:**
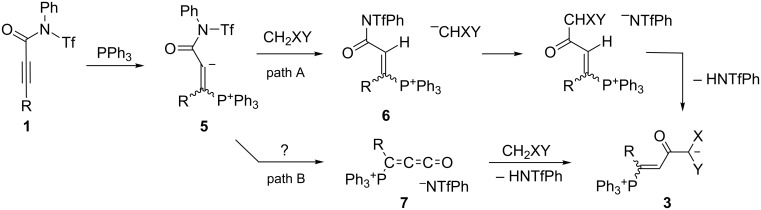
Two mechanistic scenarios for the formation of betaines **3**.

We propose two mechanistic pathways for the synthesis of betaines **3** from *N*-triflylpropiolamides **1** (Scheme 6). Both of them begin with the conjugate addition of PPh_3_ at the C–C-triple bond, leading to the vinyl anion intermediate **5**. On pathway **A**, **5** is protonated by the active methylene compound to furnish the vinylphosphonium ion **6**. These two steps have been proposed earlier for the formation of related betaines from acetylenic ketones and esters (see Introduction). A replacement of the *N*-phenyltriflamide group by the conjugate base of the active methylene compound followed by deprotonation of the CHXY group converts **6** into the final betaine **3**.

On an alternative reaction pathway (path B in Scheme 6), the initial vinyl anion **5** could be split into a 3-phosphonio-substituted propadienone **7** and the *N*-phenyltriflamide anion. The latter is able to deprotonate the active methylene compound, the anion of which finally would add to the propadienone. This pathway is notable insofar as propadienones (methylene ketenes) are short-lived compounds which have been generated by flash vapor pyrolysis at high temperatures and characterized spectroscopically or by follow-up products [23]. Under synthetically more convenient conditions, some propadienones have been generated at subambient temperature from 2-bromoacryloyl chlorides by 1,2-elimination induced by the [Mn(CO)_5_]^−^ anion [24]. For both strategies, the intermediate formation of the cumulene was confirmed in favorable cases by the isolation of [2 + 2] cyclodimerization products (e.g., 3,3-diphenylpropadienone → 2,4-diphenylmethylenecyclobutane-1,3-dione [24]). As we have not obtained so far an experimental evidence for the intermediate formation of cumulenes **7**, pathway **B** is currently speculative but may suggest a novel approach to propadienones.

The presentation of 1,5-zwitterions **3** with the negative charge residing on a carbon atom is more or less a formal one. The structural and spectroscopic data (vide infra) support the view that the mesomeric enolate structure **3’** (see Scheme 3) significantly contributes to the bonding state and that an even wider delocalization of the negative charge, represented by formula **3”**, does occur. Therefore, these zwitterions are better described as 1-phosphonium-5-oxabetaines than -5-carbabetaines. Furthermore, they represent a novel type of phosphonium enolate betaines and may be considered as vinylogues of recently reported acylphosphonium zwitterions [25].

### Structural studies

**Figure 2:**
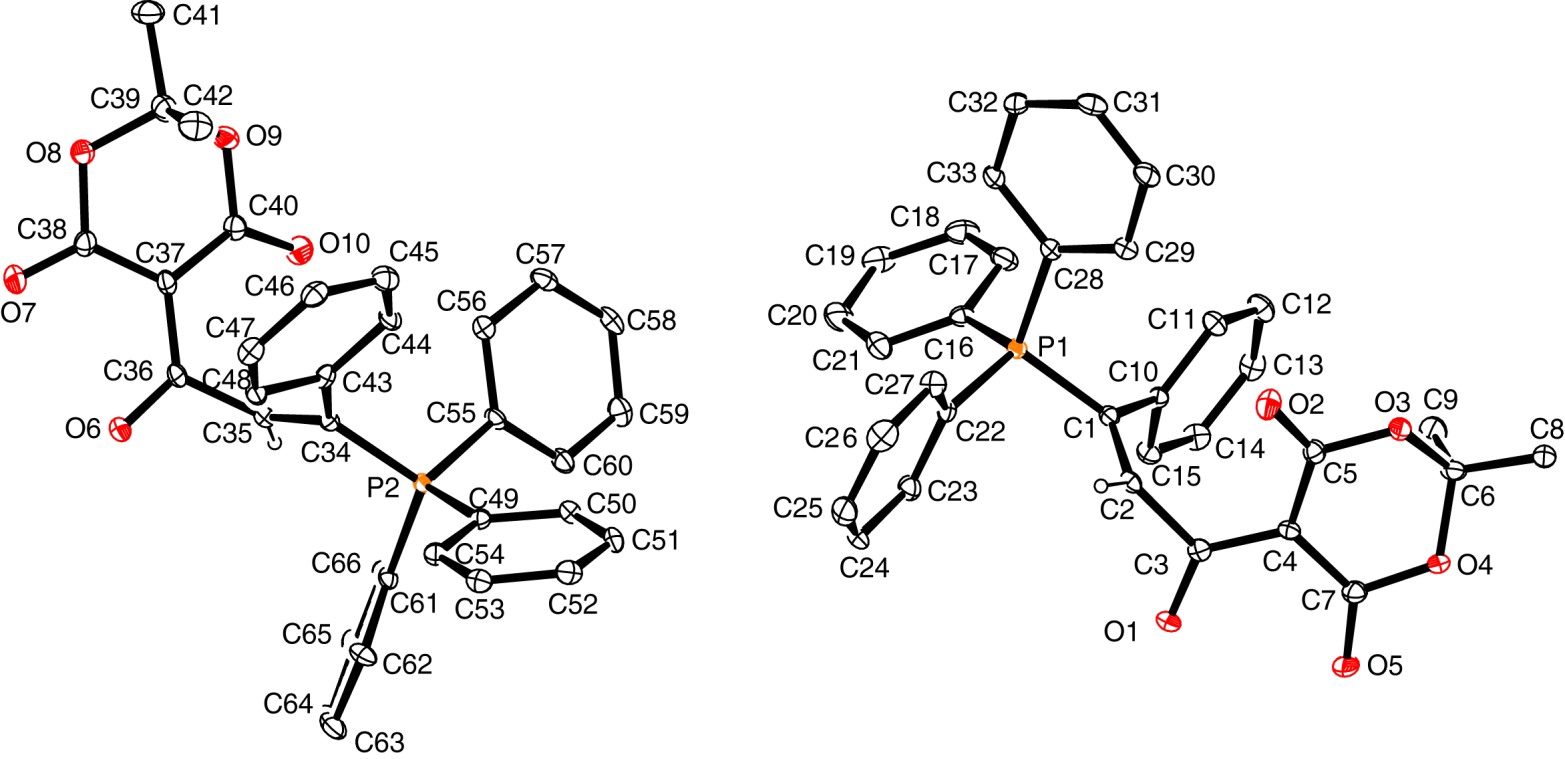
Solid-state structure of *E*-**3a** (ORTEP plot), two symmetry-independent molecules in the triclinic unit cell.

**Figure 3:**
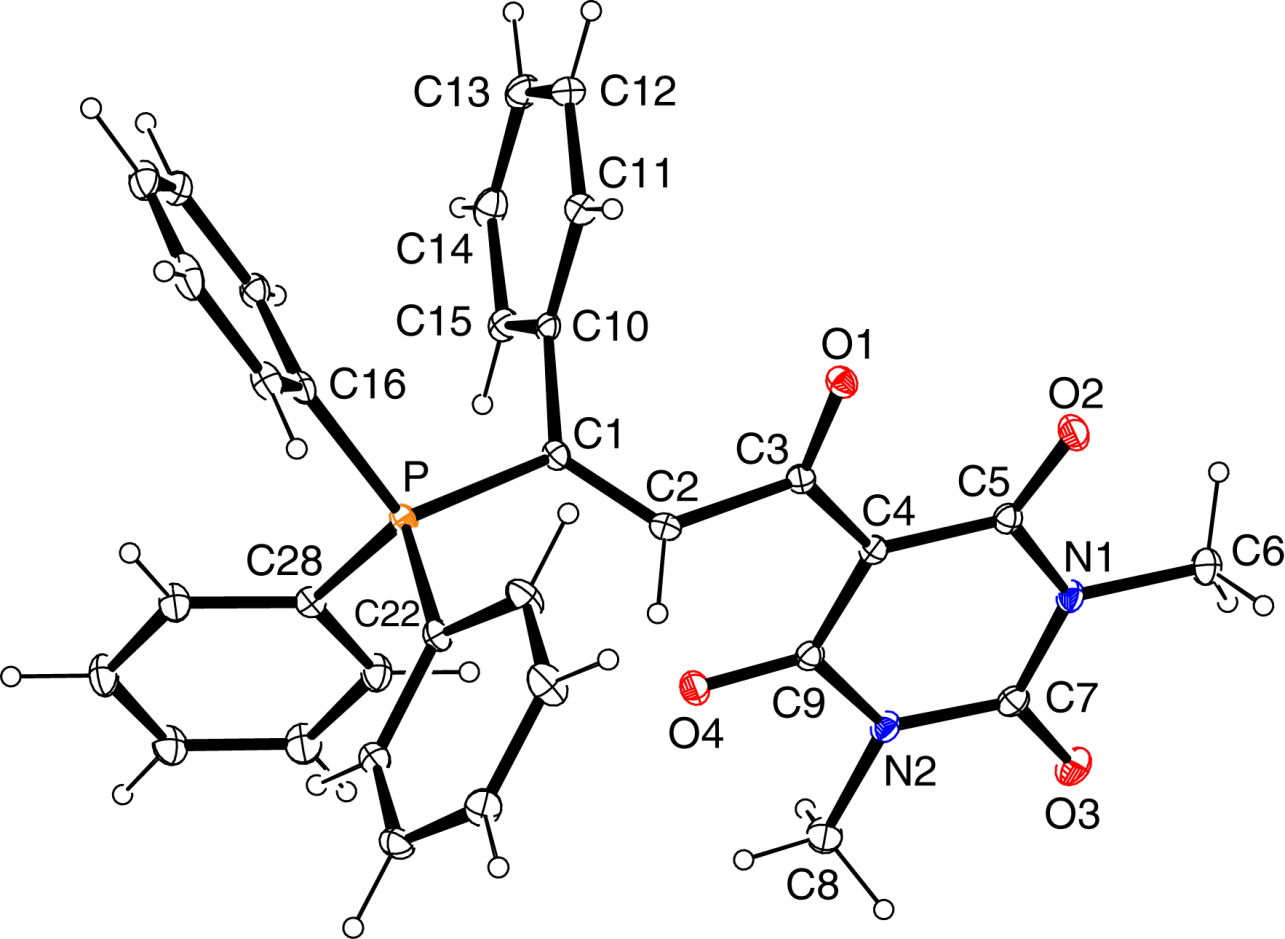
Solid-state structure of *E*-**3b**·CH_2_Cl_2_ (ORTEP plot); CH_2_Cl_2_ solvate molecule not shown.

**Figure 4:**
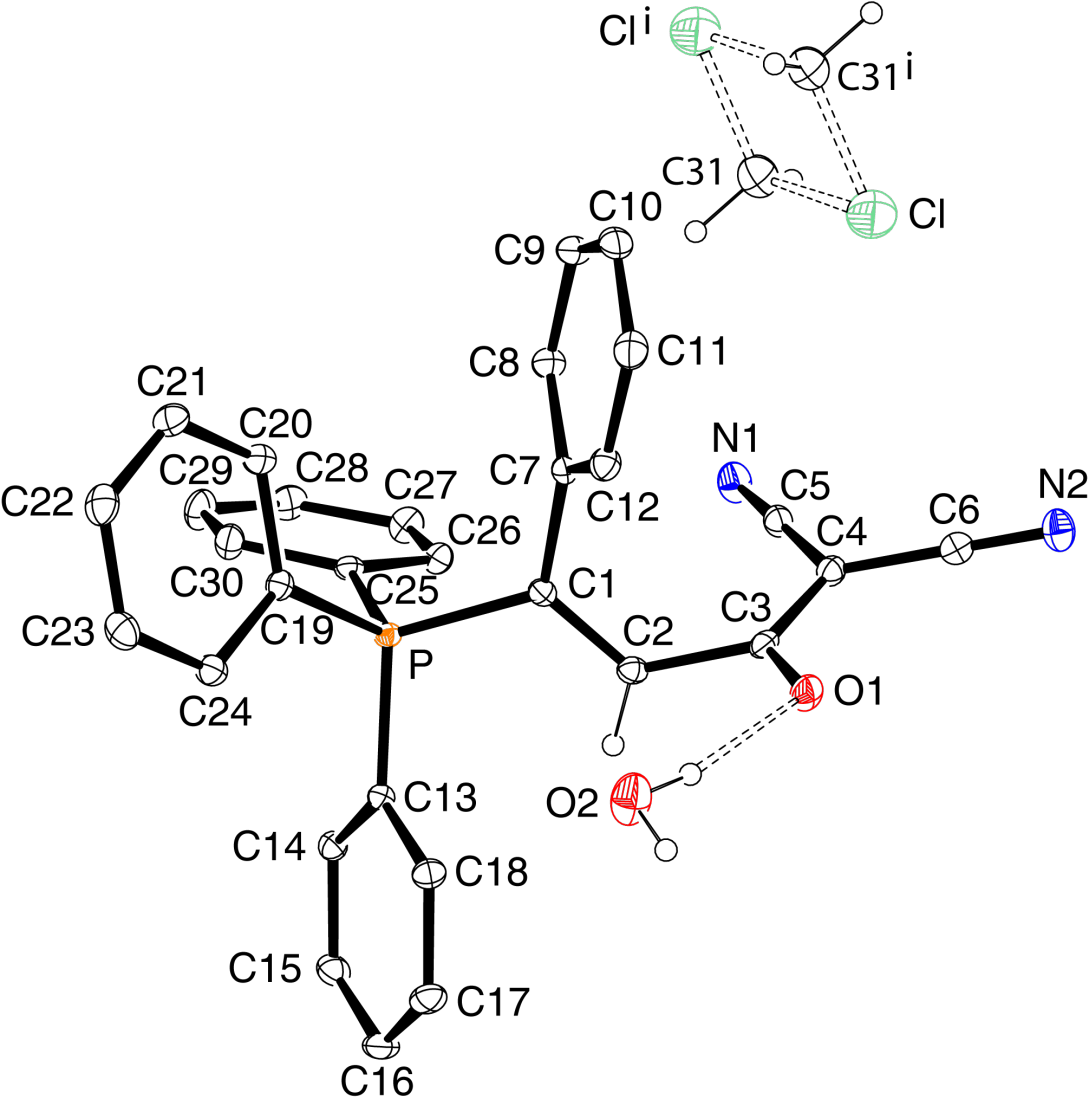
Solid-state structure of *E*-**3e**·H_2_O·CH_2_Cl_2_ (ORTEP plot). The CH_2_Cl_2_ solvate molecule is disordered. Hydrogen-bonded H_2_O molecule: O1···O2 2.907(2) Å, angle O1···H–O2 169(3)°.

**Figure 5:**
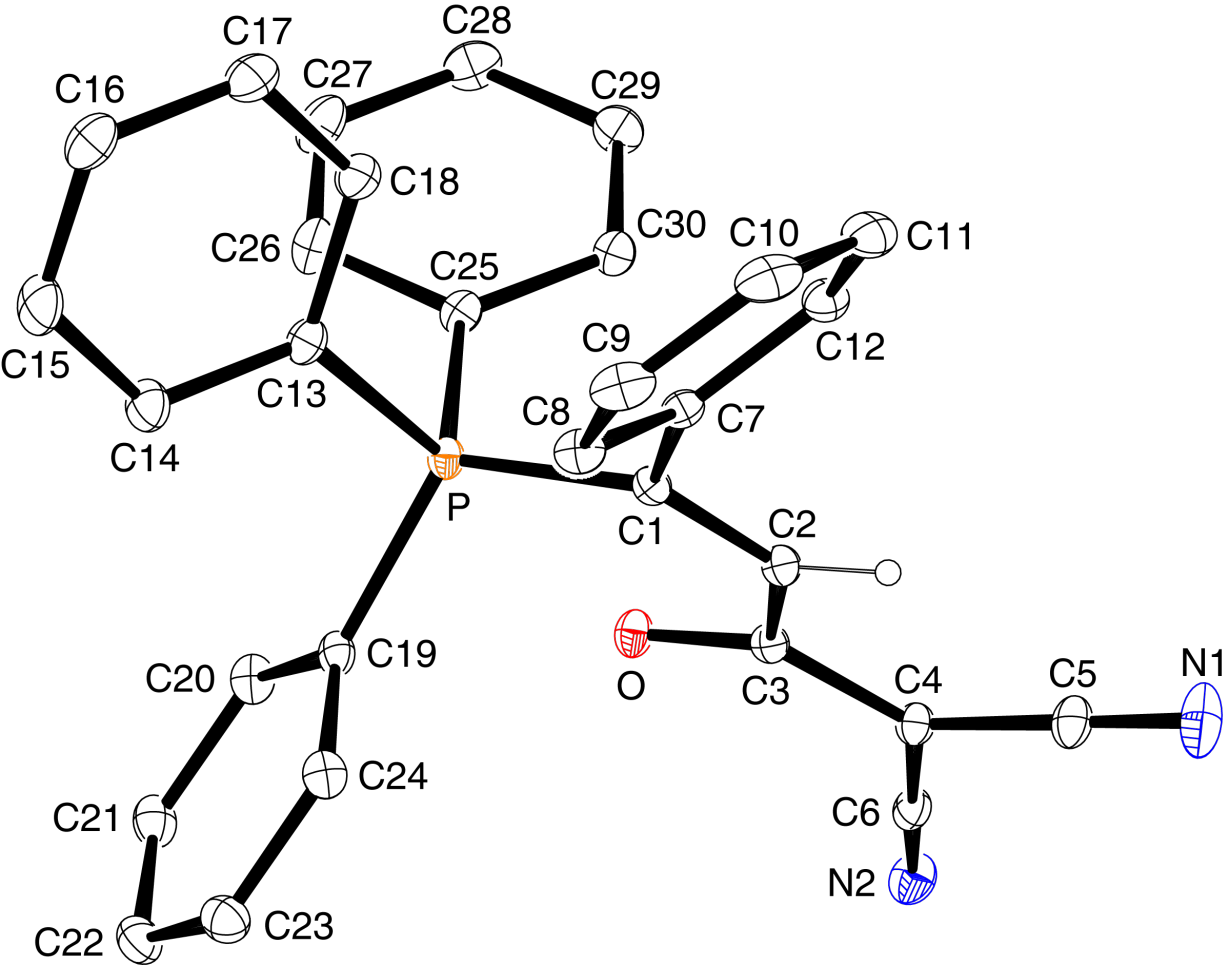
Solid-state structure of *Z*-**3e** (ORTEP plot).

The solid-state molecular structures of betaines *E*-**3a**, *E*-**3b**, *E*-**3e** and *Z*-**3e** were determined by single-crystal X-ray diffraction analysis and are shown in Figures 2–5. Some data characterizing the bonding geometry of the P^+^–C1–C2–C3(–O)–C4 backbone are compiled in Table 1. For all molecules, the bond distances P–C1, C1–C2 and C2–C3 are in the typical ranges of the respective bond type and the values are more or less identical within the estimated standard deviations; exceptions can be seen, however, for *E*-**3e** vs *Z*-**3**e. On the other hand, the bond lengths C3–C4, C4–C5 and C3–O1 clearly indicate a partial double bond character, as can be expected when the negative charge is dispersed over the almost planar O–C3–C4–(X,Y) moiety (see formulae **3’** and **3’’** in Scheme 3). The NMR observation of magnetic non-equivalence of chemically equivalent atoms in the CXY fragment of some betaines (mainly those with X = Y = CN) also indicates that free rotation around the C3–C4 bond is slow on the NMR time scale around 295 K.

**Table 1:** Bond distances and torsion angles in betaines *E*-**3a**, *E*-**3b**, *E*-**3e**, and *Z*-**3e** based on XRD data.^a^

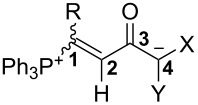					

	*E*-**3a** molecule 1	*E*-**3a** molecule 2^b^	*E*-**3b**	*E*-**3e**	*Z*-**3e**

Distances [Å]					

P–C1	1.794(6)	1.801(6)	1.799(2)	1.8076(16)	1.8237(13)
C1–C2	1.342(9)	1.341(9)	1.332(3)	1.335(2)	1.341(2)
C2–C3	1.507(4)	1.504(9)	1.512(3)	1.508(2)	1.484(2)
C3–C4	1.428(9)	1.416(9)	1.437(3)	1.406(2)	1.411(2)
C4–C5	1.432(9)	1.426(9)	1.435(3)		
C3–O1	1.248(8)	1.241(8)	1.238(3)	1.246(3)	1.249(2)

Torsion angles [deg]					

P–C1–C2–C3	171.4(5)	174.2(5)	−173.84(15)	−178.0(3)	−2.5(2)

C1–C2–C3–O1 C1–C2–C3–C4	−100.0(7) 78.7(8)	−98.4(7) 81.3(8))	63.5(3) −120.0(2)	99.5(2) −82.3(2)	0.8(2) −178.5(1)

C2–C3–C4–C5 C2–C3–C4–C9	12.9(9)	−176.0(6)	−170.9(2) 6.2(3)	0.8(2)	175.7(1)

C2–C3–C4–C6				−177.7(1)	−5.3(2)

^a^Note that the atom numbering refers to the molecule plots shown in Figures 2–5 and is different from the systematic nomenclature. ^b^Bond geometries involving the same atoms as in molecule **1**; compare Figure 2 and Figure 3.

A major difference between the *E* and *Z*-betaines **3** lies in the conformation at the C2–C3 single bond. The torsion angle C1–C2–C3–C4 amounts to 78.7–82.3° in *E*-**3a** and *E*-**3e**, 120.0° in *E*-**3b**, but 178.5° in *Z*-**3e**. Thus, the olefinic π-bond and the delocalized anionic π-system are electronically decoupled in the *E*-isomers, but coplanar in *Z*-**3e**. These conformational differences can explain why the *E*-isomers are colorless and *Z*-**3e** is yellow [UV–vis, acetonitrile, 4 × 10^−4^ mol L^−1^: λ_max_ (lg ε) = 368 nm (2.42) for *E*-**3e**, 390 nm (2.94) for *Z*-**3e**].

**Scheme 7:**
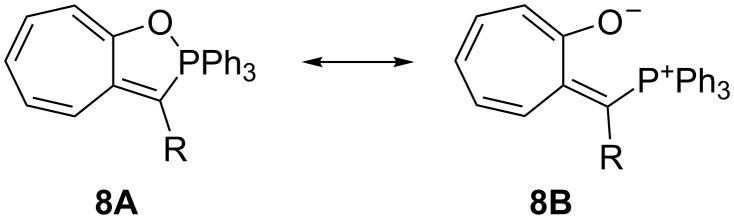
Resonance structures describing the bonding in 1,2-oxaphospholes/1,5-betaines **8**.

Notably, *Z*-**3e** does not undergo a 1,5-cyclization leading to a 5-methylene-2,2,2-triphenyl-2,5-dihydro-1,2λ^5^-oxaphosphole. Other studies have come to the conclusion, that the bonding situation in annelated oxaphospholes **8** (Scheme 7) is best described by the contribution of two canonical structures: an oxaphosphole **8A** with a pentacovalent phosphorus atom and a P–O bond, and a betaine structure **8B** with a phosphonium group and a P···O distance that varies with the electronic properties of the substituent R (Scheme 7) [26–28]. In *Z*-**3e**, the intramolecular P···O distance is 2.691 Å, significantly longer than in compounds **8** (2.00–2.36 Å), but still much shorter than the sum of the van der Waals radii (3.32 Å). A distance similar to that in *Z*-**3e** has been reported for a betaine **III** (Scheme 1) (PR_3_ = PMe_2_Ph, R’ = Ph, 2.597 and 2.620 Å in two symmetry-independent molecules [15]). Furthermore, the coordination around the phosphorus atom comes close to a trigonal-pyramidal geometry with the oxygen and the carbon atom C13 in apical positions: the angle O···P–C13 is 177.1° and the sum of bond angles involving atoms C1, C19 and C25 in the equatorial plane is 346.8^o^ (compared to 328.4° for an ideal tetrahedral coordination). All these data point to the description of *Z*-**3e** as a 1,5-betaine with an attractive electrostatic interaction of phosphorus and oxygen. The ^31^P NMR chemical shift (δ = 23.53 ppm) further confirms the presence of a tetracovalent phosphorus atom (see [28]).

### NMR data of betaines **3**

The ^1^H, ^13^C and ^31^P data of the P–C3–C2–C1 chain in betaines **3** are listed in Table 2.

**Table 2:** Selected NMR data of betaines **3**^a^.

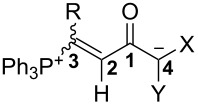								

Betaine	*E* or *Z*	^1^H NMR	^13^C NMR					^31^P NMR^b^
		2-H	C-3	C-2	C-1	C-4	P-C_Ph_^c^	

**3a**	*E*	7.56 (23.52)	114.55 (78.7)	160.26 (7.7)	183.64 (17.7)	89.79 (3.0)	118.35 (88.0)	24.47
**3b**	*E*	≈7.6^d^	113.45 (79.6)	161.68 (8.0)	184.16 (18.0)	96.98 (3.0)	118.80 (87.9)	24.43
	*Z*	8.36 (41.6)	121.07 (80.4)	160.13 (5.9)	182.66 (5.8)	100.14 (0)	121.67 (92.0)	23.17
**3c**	*E*	7.69 (23.5)	120.27 (75.9)	158.34 (6.7)	180.16 (16.8)	109.72 (2.8)	116.97 (88.2)	24.78
**3e**	*E*	7.20 (22.7)	125.41 (72.6)	153.71 (7.9)	182.60 (18.2)	52.20 (2.4)	117.01 (87.9)	25.21
	*Z*	7.91 (38.8)	≈129.5^e^ (≈80 Hz)	148.25 (5.0)	176.93 (4.1)	56.26 (2.2)	123.70 (95.1)	23.53
**3f**	*E*	7.48 (22.8)	124.04 (73.1)	155.95 (7.3)	176.19 (17.7)	65.55 (2.8)	117.42 (87.9)	24.55
	*Z*	8.22 (40.0)	130.84 (56.7)	149.50 (5.5)	170.09 (3.7)	not observed	128.24 (85.1)	19.26
**3g**	*E*	7.69 (23.1)	112.50 (80.9)	162.19 (7.7)	183.77 (17.6)	96.80 (2.6)	118.10 (88.2)	24.38
**3h**^f^	*E*	7.24 (22.2)	?	154.61 (7.6)	182.23 (17.6)	52.55 (2.2)	116.81 (88.0)	25.14
	*Z*	7.89 (38.3)	?	148.68 (5.0)	176.57 (4.0)	56.55 (2.4)	?	22.95
**3i**	*E*	7.18 (30.8)	119.07 (65.2)	163.46 (9.0)	186.80 (21.4)	96.51 (2.2)	121.38 (85.9)	28.96
	*Z*	8.18 (46.1)	125.69 (66.3)	158.34 (6.9)	185.0 (7.4)	97.63 (0)	122.54 (88.0)	23.98
**3j**	*E*	7.42 (25.2)	114.11 (74.4)	161.04 (7.4)	184.98 (19.8)	96.89 (3.4)	118.78 (87.1)	26.24
**3k**	*E*	7.44 (23.9)	114.26 (79.0)	163.33 (9.9)	185.22 (18.8)	96.66 (3.4)	119.36 (88.0)	25.39
	*Z*	8.30 (42.2)	123.77 (81.0)	164.11 (?)	182.75 (15.3)	97.16 (0)	122.64 (92.1)	24.21
**3l**	*E*	6.83 (30.8)	131.87 (58.6)	158.12 (8.5)	185.45 (21.4)	51.40 (2.6)	120.09 (85.8)	30.23
**3m**	*E*	7.02 (25.1)	126.55 (68.8)	151.64 (7.4)	182.21 (20.1)	53.33 (2.9)	117.19 (87.2)	26.92
	*Z*^g^		122.91 (57.3)					26.09
**3n**	*E*	6.86 (24.0)	125.57 (72.2)	153.91 (9.5)	183.49 (19.2)	52.46 (2.4)	117.65 (87.0)	26.48
**3o**	*E*	7.33 (20.3)	113.69 (87.9)	152.49 (3.9)	177.07 (18.6)	56.19 (0)	117.54 (91.3)	20.51

^a^Spectra were recorded in CDCl_3_; chemical shifts (δ/ppm) and (in parentheses) P,H coupling constants (*J*/Hz) are given. ^b^Relative to 85% aqueous H_3_PO_4_ standard. ^c^The signal of the P–C_Ph_ (= C_ipso_) nuclei can be distinguished reliably from the C-3 signal by the larger ^1^*J*_P,C_ coupling constant and its higher intensity. ^d^Signal covered by other signals. ^e^Not determined precisely, since one branch of the doublet signal is hidden. ^f^An *E*:*Z* ratio between 54:46 and 60:40 was determined in different spectra of the *E*/*Z* mixture (CDCl_3_ vs DMSO-*d*_6_, ^1^H vs ^31^P). Not all signals could be assigned reliably to one or the other isomer. The data presented here were assigned by analogy with the pure isomers of other *E*/*Z* pairs. ^g^*Z*-**3m** was obtained only as the minor component in admixture with *E*-**3m**.

Based on the spectra of those betaines, for which the molecular geometry has been established by XRD analysis (vide supra), the stereochemistry at the P-substituted olefinic bond could be determined in all cases from characteristic chemical shifts of the olefinic proton and from ^3^*J*(P,H) and ^3^*J*(P,C) coupling constants. The olefinic proton signal of the *E*-isomers was found in the δ range 6.8–7.7 ppm, the signal of the *Z*-isomers at 7.9–8.4 ppm. In all cases where both diastereoisomers were available, the known relationship ^3^*J*(P,H^cis^) (in the *E*-isomer) < ^3^*J*(P,H^trans^) [29–30] was valid; the values were in the range 20.3–30.8 Hz for the *cis* coupling and 38.3–46.1 Hz for the *trans* coupling. The magnitude of the coupling between the tetravalent phosphorus nucleus and the carbonyl carbon atom C-1 also follows the known trend ^3^*J*(P,C^cis^) < ^3^*J*(P,C^trans^) [29]. The ranges – 3.7–7.4 Hz vs 15.3–21.4 Hz – are sufficiently different to distinguish between *E* and *Z*-isomers even if only one of them is available.

The ^31^P NMR chemical shifts were found between 19.26 (for *Z*-**3f**) and 30.23 ppm (for *E*-**3l**), the typical range being 23–27 ppm. As in other alkenes bearing a tetravalent phosphorus atom [29], the values of the *E*-isomers are always somewhat higher than those of the *Z*-isomers. Considering the question of an intramolecular P···O coordination in the *Z*-isomers (vide supra), the chemical shift values by themselves (compare: δ_P_ = 18.7 ppm for (*E*)-Ph_3_P^+^–CH=CHMe Br^−^ [31]) and the similarity of the chemical shifts for *E*/*Z* pairs indicate a tetracovalent rather than a pentacovalent phosphorus atom [28].

## Conclusion

We have observed that the reaction of *N*-triflyl-*N*-phenylpropiolamides with triphenylphosphane results in undefined polymerization and formation of *N*-phenyltriflamide. In the presence of several active methylene compounds, however, a clean three-component reaction leads to a novel type of phosphonium betaines, which can formally be named 1,5-phosphonium-carbabetaines, but because of the extended delocalization of the negative charge are better described as 1-phosphonium-5-oxabetaines. The excellent leaving group character of the *N*-phenyltriflylamide anion ([N(Ph)SO_2_CF_3_]^−^) is a key factor in the reaction course, because analogous three-component reactions using propiolic acid esters as Michael acceptors are known to furnish another type of betaines in which the ester group (COOR) is retained. Questions on some mechanistic details, in particular at which stage of the reaction sequence the triflamide anion departs, remain to be answered.

## Supporting Information

File 1 Experimental procedures, characterization data, NMR spectra (^1^H, ^13^C, ^31^P, ^19^F) and IR spectra for the synthesized compounds, and data for the X-ray crystal structure determinations.
