# Supplementary material for: 1,5-Phosphonium betaines from N-triflylpropiolamides, triphenylphosphane, and active methylene compounds
Source: Beilstein J Org Chem. 2019 Nov 1;15:2603–11. doi: 10.3762/bjoc.15.253 (PMC6839568; doi:10.3762/bjoc.15.253)
Supplement: File 1 — Experimental procedures, characterization data, NMR spectra (1H, 13C, 31P, 19F) and IR spectra for the synthesized compounds, and data for the X-ray crystal structure determinations. [file Beilstein_J_Org_Chem-15-2603-s001.pdf]

BEILSTEIN JOURNAL OF ORGANIC CHEMISTRY

Supporting Information

for

1,5-Phosphonium betaines from *N*-triflylpropiolamides, triphenylphosphane, and active methylene compounds

Vito A. Fiore, Chiara Freisler and Gerhard Maas

Beilstein J. Org. Chem. **2019,** *15,* 2603–2611. doi:10.3762/bjoc.15.253

Experimental procedures, characterization data, NMR spectra (^{1}H, ^{13}C, ^{31}P, ^{19}F) and IR spectra for the synthesized compounds, and data for the X-ray crystal structure determinations

License and Terms: This is a supporting information file under the terms of the Creative Commons Attribution License (http://creativecommons.org/licenses/by/4.0). Please note that the reuse, redistribution and reproduction in particular requires that the authors and source are credited.
The license is subject to the *Beilstein Journal of Organic Chemistry* terms and conditions: (https://www.beilstein-journals.org/bjoc)

Table of contents

1. General information ………………………………………………………………………….. S2

2. Syntheses and characterization of betaines 3 ………..………………………………………… S2

3. X-ray crystal structure determinations ....…………………………………………… S13

3.1. Crystallographic data for (*E*)-**3a** ..……………………………………………………... S13

3.2. Crystallographic data for (*E*)-**3b**·CH_2Cl_2 ..…………………………………….…...... S15

3.3. Crystallographic data for (*Z*)-**3e** ……..………………..………………………………...S16

3.4. Crystallographic data for (*E*)-**3e**·H_2O·CH_2Cl_2 …………………..…..………..…………... S17

3. References S18

4. Spectra of betaines 3 (^{1}H, ^{13}C, ^{19}F, ^{31}P NMR; IR) …… …………………………………...…. S19

1. General information

Melting points were determined in open capillaries with a Büchi B-540 instrument at a heating rate of 2 °C min^{-1}. IR spectra of solids were taken from solids as KBr pellets or from oils between NaCl plates and were recorded on a Bruker Vector 22 FT-IR instrument. NMR spectra: Bruker Avance 400 (^{1}H: 400.13 MHz; ^{13}C: 100.62 MHz; ^{19}F: 376.47 MHz; ^{31}P: 161.98 MHz) and Bruker Avance 500 (^{1}H: 500.14 MHz; ^{13}C: 125.77 MHz). ^{1}H and ^{13}C spectra were referenced to the residual solvent signal [^{1}H: δ = 7.26 ($CHCl_3$) 1.94 (CD_3CN); ^{13}C: δ = 77.16 ($CDCl_3$), 1.32/118.26 (CD_3CN) ppm. ^{19}F NMR spectra were referenced to internal C_6F_6 (−162.90 ppm) and ^{31}P spectra to external 85% aqueous H_3PO_4 (δ = 0.00 ppm). ^{13}C NMR spectra were recorded in the broad-band ^{1}H-decoupled mode. Mass spectra: Finnigan-MAT SSQ-7000 (CI, 100 eV) and SolariX (HRMS, ESI). Elemental analyses: elementar Hanau vario MICRO cube analyser.

The synthesis of *N*-triflyl-propiolamides **1** has been published [1].

2. Syntheses and characterization of betaines 3

2.1. General procedure

Triphenylphosphane (PPh_3, 1.00 equiv) and a methylene compound **2** (1.00 equiv) were dissolved in anhydrous dichloromethane (5 mL/mmol) at room temperature and an *N*-triflyl-propiolamide **1** (1.03 equiv) was added. The solution was stirred until the reaction was complete (0.5–6 hours, reaction control by TLC, ^{19}F or ^{31}P NMR spectroscopy), then slowly added to ice-cold diethyl ether (≈50 mL), whereupon betaines **3** precipitated. They were isolated by filtration and washed with a few milliliters of cold ether to furnish a product which in most cases was pure by elemental analysis. The ratio of *E* and *Z* diastereoisomers was determined by ^{31}P NMR signal integration. A separation of the diastereoisomers, which can be distinguished by color and habit, was achieved in several cases by the solvent diffusion method from CH_2Cl_2/pentane at 8 °C. The *E* isomer, as the major component, crystallized first and was isolated by filtration. The *Z* isomer crystallized from the mother liquor, when the solvent diffusion procedure was continued.

2.1.1. (*E*)-2,2-Dimethyl-4,6-dioxo-5-(3-phenyl-3-(triphenylphosphonio)acryloyl)-1,3-dioxan-5-ide ((*E*)-3a)

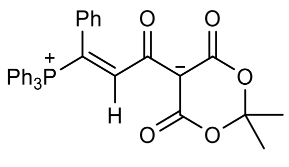

Prepared from triflamide **1** (361 mg, 1.03 mmol), PPh_3 (262 mg, 1.00 mmol) and Meldrum's acid (144 mg, 1.00 mmol); 2 hours. Yield: 518 mg (0.97 mmol, 97%). Pale yellow crystals of **(*E*-) 3a**, m.p. 194 °C. – ^{1}H NMR ($CDCl_3$, 500.13 MHz): δ [ppm] = 1.43 (s, 6H, CH_3), 6.98–7.00 (m, 2H, H_{Ph}), 7.08–7.12 (m, 2H, H_{Ph}), 7.18–7.21 (m, 1H, H_{Ph}), 7.56 (d, $^{3}J_{H,P}$ = 23.52 Hz, 1H, C=CH), 7.59–7.66 (m, 12H, H_{Ph}), 7.76–7.80 (m, 3H, H_{Ph}). – ^{13}C NMR ($CDCl_3$, 100.62 MHz): δ [ppm] = 26.41 (CH_3), 89.79 (d, $^{4}J_{C,P}$ = 3.0 Hz, C^{-}), 100.65 (OCO), 114.55 (d, $^{1}J_{C,P}$ = 78.7 Hz, P<u>C</u>=CH), 118.35 (d, $^{1}J_{C,P}$ = 88.0 Hz, PC_{Ph}), 128.66

(d, $J_{C,P}$ = 1.8 Hz, C_{sp2}), 129.04 (d, $J_{C,P}$ = 1.9 Hz), 130.15 (d, $^3J_{C,P}$ = 12.7 Hz, C_{PPh}), 130.41 (d, $J_{C,P}$ = 12.8 Hz), 130.83 (d, $J_{C,P}$ = 4.2 Hz), 131.42 (d, $J_{C,P}$ = 10.1 Hz), 134.84 (d, $J_{C,P}$ = 10.0 Hz), 135.04 (d, $^2J_{C,P}$ = 10.0 Hz, C_{PPh}), 135.13, 160.26 (d, $^2J_{C,P}$ = 7.7 Hz, C=$\underline{C}$H), 183.64 (d, $^3J_{C,P}$ = 17.7 Hz, CH-$\underline{C}$=O). – ^{31}P NMR ($CDCl_3$): δ [ppm] = 24.47.

IR (KBr): $\tilde{\nu}$ [cm^{-1}] = 1724 (s), 1659 (vs), 1560 (m), 1439 (m), 1384 (vs), 1312 (m), 1263 (m), 1197 (m), 1156 (m), 1107 (m), 1031 (m), 932 (m), 755 (m), 727 (m), 699 (m), 544 (m), 526 (m). – MS (CI, 100 eV): *m/z* (%) = 359 (28), 331 (24), 313 (15), 263 $[PPh_3 + H]^+$ (39), 185 $[C_8H_8O_5 + H]^+$ (100). – Anal. for $C_{33}H_{27}O_5P$ (534.55 g/mol): calcd. C 74.15, H 5.09 N 0.00; found C 74.26, H 5.18, N 0.02.

In the ^{1}H NMR spectra of the crude product, a trace of *(Z)*-**3a** was detected: δ = 8.32 ppm ($^3J_{H,P}$ = 41.3 Hz).

2.1.2. 1,3-Dimethyl-2,4,6-trioxo-5-(3-phenyl-3-(triphenylphosphonio) acryloyl)hexahydropyrimidin-5-ide ((*E*)- and (*Z*)-3b)

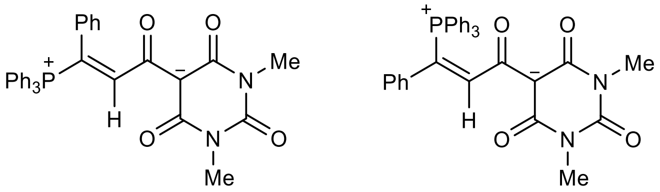

From triflamide **1** (R = Ph; 538 mg, 1.53 mmol), PPh_3 (393 mg, 1.50 mmol) and *N,N*-dimethylbarbituric acid (234 mg, 1.50 mmol); 2 hours. Yield: 795 mg (1.45 mmol, 97%) of a light yellow mixture of diastereoisomers, *E*/*Z* = 83:17, which could be separated by fractionating solvent diffusion crystallization.

(*E*)-3b: colorless, m.p. 242 °C. – ^{1}H NMR ($CDCl_3$, 400.13 MHz): δ [ppm] = 3.24 (s, 6H, NCH_3), 6.93–6.96 (m, 2H, H_{Ph}), 7.06–7.10 (m, 2H, H_{Ph}), 7.16–7.20 (m, 1H, H_{Ph}), 7.58–7.68 (m, 13H, H_{Ph}, C=CH), 7.75–7.80 (m, 3H, H_{Ph}). – ^{13}C NMR ($CDCl_3$, 100.62 MHz): δ [ppm] = 27.24 (NCH_3), 96.98 (d, $^4J_{C,P}$ = 3.0 Hz, C^-), 113.45 (d, $^1J_{C,P}$ = 79.6 Hz, P$\underline{C}$=CH), 118.80 (d, $^1J_{C,P}$ = 87.9 Hz, PC_{Ph}), 128.60 (d, $J_{C,P}$ = 1.5 Hz, C_{Ph}), 128.95 (d, $J_{C,P}$ = 2.3 Hz, C_{Ph}), 130.09 (d, $^3J_{C,P}$ = 12.6 Hz, C_{PPh}), 130.92 (d, $J_{C,P}$ = 4.5 Hz, C_{Ph}), 131.71 (d, $J_{C,P}$ = 10.3 Hz, C_{Ph}), 135.00 (d, $^4J_{CP}$ = 2.9 Hz, C_{PPh}), 135.12 (d, $^2J_{C,P}$ = 10.0 Hz, C_{PPh}), 153.28 (N,N-C=O), 161.68 (d, $^2J_{C,P}$ = 8.0 Hz, C=$\underline{C}$H), 164.06 (br, N-C=O), 184.16 (d, $^3J_{C,P}$ = 18.0 Hz, CH-$\underline{C}$=O). – ^{31}P NMR ($CDCl_3$): δ [ppm] = 24.43.

IR (KBr): $\tilde{\nu}$ [cm^{-1}] = 1701 (w), 1649 (s), 1615 (s), 1419 (s), 1386 (m). – HRMS (MALDI-TOF): *m/z* = 547.17828 $[M+H]^+$; calcd. 547.17867 – Anal. for $C_{33}H_{27}N_2O_4P$ (546.56 g/mol): calcd. C 72.52, H 4.98, N 5.13; found C 72.50, H 4.97, N 5.01.

(*Z*)-3b: yellow, m.p. 244 °C. – ^{1}H NMR ($CDCl_3$, 400.13 MHz): δ [ppm] = 3.24 (s, 6H, NCH_3), 6.99–7.01 (m, 2H, H_{Ph}), 7.07–7.10 (m, 2H, H_{Ph}), 7.13–7.17 (m, 1H, H_{Ph}), 7.41–7.45 (m, 6H, H_{Ph}), 7.55–7.63 (m, 9H, H_{Ph}), 8.36 (d, $^3J_{H,P}$ = 41.58 Hz, 1H, C=CH). – ^{13}C NMR ($CDCl_3$, 125.77 MHz): δ [ppm] = 27.42 (NCH_3), 100.14 (C^-), 121.07 (d, $^1J_{C,P}$ = 80.4 Hz, P-$\underline{C}$=CH), 121.67 (d, $^1J_{C,P}$ = 92.0 Hz, PC_{Ph}), 128.41, 128.50, 129.20 (d, $^3J_{C,P}$ = 13.1 Hz), 129.81 (d, J_{CP} = 4.1 Hz), 130.12 (d, $J_{C,P}$ = 12.6 Hz), 133.47 (d, $^4J_{C,P}$

= 2.9 Hz), 134.42 (d, $^{2}J_{C,P}$ = 9.9 Hz), 135.14 (d, $J_{C,P}$ = 9.9 Hz), 153.06 (N,N-C=O), 160.13 (d, $^{2}J_{C,P}$ = 5.9 Hz, C=C̲H), 182.66 (d, $^{3}J_{C,P}$ = 5.8 Hz, CH-C̲=O). – ^{31}P NMR ($CDCl_3$): δ [ppm] = 23.17.
IR (KBr): $\tilde{\nu}$ [cm^{-1}] = 1698 (m), 1639 (s), 1618 (s), 1553 (m), 1477 (m), 1415 (s), 1387 (m), 1106 (m), 1009 (w). – HRMS (MALDI-TOF): *m/z* = 547.17828 [M+H]$^+$; calcd. 547.17867. – Anal. for $C_{33}H_{27}N_2O_4P$ (546.56 g/mol): calcd. C 72.52, H 4.98, N 5.13; found C 72.50, H 4.97, N 5.01.

2.1.3. (*E*)-1,3-Dioxo-2-(3-phenyl-3-(triphenylphosphonio)acryloyl)-2,3-dihydro-1*H*-inden-2-ide ((*E*)-3c)

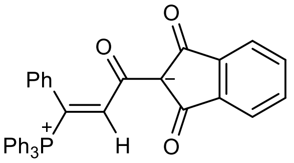

From triflamide **1** (R = Ph; 361 mg, 1.03 mmol), PPh_3 (262 mg, 1.00 mmol) and indane-1,3-dione (146 mg, 1.00 mmol); 2 hours. Only the *E* isomer was observed. Yield: 440 mg (0.81 mmol, 82%); orange solid, m.p. 234 °C. –
^{1}H NMR ($CDCl_3$, 500.13 MHz): δ [ppm] = 6.90–6.92 (m, 2H, H_{Ar}), 7.01–7.4 (m, 2H, H_{Ar}), 7.12–7.15 (m, 1H, H_{Ar}), 7.30–7.62 (m, br, 16H, H_{Ar}), 7.69 (d, $^{3}J_{H,P}$ = 23.51 Hz, 1H, C=CH), 7.76–7.79 (m, 3H, H_{Ar}). – ^{13}C NMR ($CDCl_3$, 125.77 MHz): δ [ppm] = 109.72 (d, $^{4}J_{C,P}$ = 2.8 Hz, C$^-$), 116.97 (d, $^{1}J_{C,P}$ = 88.2 Hz, PC_{Ph}), 119.68, 120.27 (d, $^{1}J_{C,P}$ = 75.9 Hz, PC̲=CH), 120.52, 128.39, 128.94, 130.00 (d, $^{3}J_{C,P}$ = 12.8 Hz), 130.47 (d, $J_{C,P}$ = 4.0 Hz), 130.66 (d, $J_{C,P}$ = 9.6 Hz), 131.66 (d, $J_{C,P}$ = 20.2 Hz), 134.47 (d, $J_{C,P}$ = 10.1 Hz), 134.84 (d, $^{2}J_{C,P}$ = 10.1 Hz), 135.16 (d, $^{4}J_{C,P}$ = 1.8 Hz), 138.95 (C_{indene}), 139.69 (C_{indene}), 158.34 (d, $^{2}J_{C,P}$ = 6.7 Hz, C=C̲H), 180.16 (d, $^{3}J_{C,P}$ = 16.8 Hz, CH-C̲=O), 191.14 ($C{=}O_{indene}$), 193.31 ($C{=}O_{indene}$). – ^{31}P NMR ($CDCl_3$): δ [ppm] = 24.79.
IR (KBr): $\tilde{\nu}$ [cm^{-1}] = 1683 (w), 1631 (s), 1587 (s), 1550 (m), 1437 (s), 1417 (s), 1201 (m), 1150 (m), 1106 (m), 695 (m), 600 (m), 529 (m). – HRMS (MALDI-TOF): *m/z* = 537.16133 [M + H]$^+$; calcd. 537.16196. – Anal. for $C_{36}H_{25}O_3P$ (536.57 g/mol): calcd. C 80.59, H 4.70, N 0.00; found C 80.51, H 4.62, N 0.00.

2.1.4. (*E*)-2-Benzoyl-1,3-dioxo-1,5-diphenyl-5-(triphenylphosphonio)pent-4-en-2-ide ((*E*)-3d)

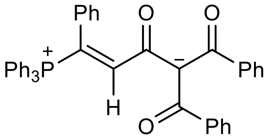

From triflamide **1** (R = Ph; 361 mg, 1.03 mmol), PPh_3 (262 mg, 1.00 mmol) and 1,3-diphenylpropane-1,3-dione (242 mg, 1.00 mmol); 2 hours. In contrast to all other preparations, the solution turned black after addition of the triflamide and by-products were formed, which could not be removed completely even after repeated recrystallization. The purification efforts ensued a considerably reduced yield of the desired product: 132 mg (0.21 mmol, 21%) of still impure product; beige solid, m.p. 116 °C. Only the *E* isomer was observed. – ^{13}C NMR ($CDCl_3$, 100.62 MHz): δ [ppm] = 93.24 (C$^-$), 115.11 (d, $^{1}J_{C,P}$ = 71.4

Hz), 118.15 (d, $^1J_{C,P}$ = 87.5 Hz, PC_{Ph}), 122.90, 123.14, 127.24, 128.53, 128.73 (d, $J_{C,P}$ = 1.4 Hz), 128.79, 129.03 (d, J_{CP} = 4.5 Hz), 130.24 (d, $^3J_{C,P}$ = 12.5 Hz), 130.27, 130.34, 130.39, 130.92 (d, $J_{C,,P}$ = 12.8 Hz), 132.16 (d, $J_{C,P}$ = 10.1 Hz), 132.58, 134.69 (d, $^2J_{C,P}$ = 9.8 Hz), 134.92, 135.05 (d, $J_{C,P}$ = 5.6 Hz), 135.17, 135.22 (d, $J_{C,P}$ = 3.1 Hz), 135.57, 135.91 (d, $J_{C,P}$ = 3.0 Hz), 136.39 (d, $J_{C,P}$ = 3.3 Hz), 156.06 (C=C̲H), 166.97 (d, $^2J_{C,P}$ = 20.1 Hz, CH-C̲=O), 185.85 (Ph-C=O); some of the reported data may result from impurities, see spectrum. – ^{31}P NMR ($CDCl_3$): δ [ppm] = 24.90.

IR (KBr): $\tilde{\nu}$ [cm^{-1}] = 1760 (m), 1655 (s), 1593 (s), 1488 (s), 1440 (s), 1303 (vs), 1198 (vs), 1153 (vs), 1107 (vs), 1001 (m), 754 (s), 726 (s), 699 (vs), 599 (m), 525 (s). – HRMS (MALDI-TOF): *m/z* = 615.20843 $[M+H]^+$; calcd. for $C_{42}H_{32}O_3P^+$ 615.20836.

2.1.5. 1,1-Dicyano-2-oxo-4-phenyl-4-(triphenylphosphonio)but-3-en-1-ide ((*E*)- and (*Z*)-3e)

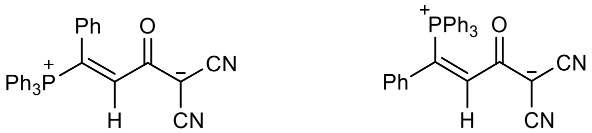

From triflamide **1** (R = Ph; 538 mg, 1.53 mmol), PPh_3 (393 mg, 1.50 mmol) and malononitrile (99 mg, 1.50 mmol); 30 minutes. Yield: 644 mg (1.41 mmol, 94%) of a mixture of diastereoisomers, *E*/*Z* = 67:33), which could be separated by solvent diffusion crystallization.

(*E*)-3e: colorless, m.p. 292 °C. – ^{1}H NMR ($CDCl_3$, 400.13 MHz): δ [ppm] = 7.03–7.06 (m, 2H, H_{Ph}), 7.17–7.23 (m, 2H, H_{Ph}), 7.20 (d, $^3J_{HP}$ = 22.7 Hz, 1H, C=CH), 7.28–7.33 (m, 1H, H_{Ph}), 7.47–7.53 (m, 6H, H_{Ph}), 7.61–7.66 (m, 6H, H_{Ph}), 7.79–7.84 (m, 3H, H_{Ph}). – ^{13}C NMR ($CDCl_3$, 100.62 MHz): δ [ppm] = 52.20 (d, $^4J_{C,P}$ = 2.4 Hz, C^-), 117.01 (d, $^1J_{C,P}$ = 87.9 Hz, PC_{Ph}), 118.02 (C≡N), 122.31 (C≡N), 125.41 (d, $^1J_{C,P}$ = 72.6 Hz, PC̲=CH), 128.89 (d, $J_{C,P}$ = 1.7 Hz), 129.81 (d, $J_{C,P}$ = 2.5 Hz), 129.97 (d, $J_{C,P}$ = 4.4 Hz), 130.47 (d, $^3J_{C,P}$ = 12.8 Hz), 130.78 (d, $J_{C,P}$ = 9.1 Hz), 134.83 (d, $^2J_{C,P}$ = 10.1 Hz), 135.65 (d, $^4J_{C,P}$ = 3.0 Hz), 153.71 (d, $^2J_{C,P}$ = 7.9 Hz, C=C̲H), 182.60 (d, $^3J_{C,P}$ = 18.2 Hz, CH-C̲=O). – ^{31}P NMR ($CDCl_3$): δ [ppm] = 24.78.

UV (acetonitrile, $4 \cdot 10^{-4}$ mol L^{-1}): λ_{max} [nm] (lg ε) = 207 (3.64), 368 (2.42). – IR (KBr): $\tilde{\nu}$ [cm^{-1}] = 2201 (m), 2176 (s), 1634 m), 1549 (s), 1486 (w), 1437 (m), 1367 (s), 1106 (m), 754 (m), 728 (m), 696 (m), 518 (m), 501 (m). – HRMS (MALDI-TOF): *m/z* = 457.14658 $[M + H]^+$; calcd. 457.14698. – Anal. for $C_{30}H_{21}N_2OP$ (456.48 g/mol): calcd. C 78.94, H 4.64, N 6.14; found C 79.09, H 4.73, N 6.01.

(*Z*)-3e: yellow, m.p. 272 °C. – ^{1}H NMR ($CDCl_3$, 400.13 MHz): δ [ppm] = 6.82–6.84 (m, 2H, H_{Ph}), 7.04–7.08 (m, 2H, H_{Ph}), 7.13–7.19 (m, 1H, H_{Ph}), 7.37–7.45 (m, 12H, H_{Ph}), 7.52–7.59 (m, 3H, H_{Ph}), 7.91 (d, $^3J_{H,P}$ = 38.76 Hz, 1H, C=CH). – ^{13}C NMR ($CDCl_3$, 100.62 MHz): δ [ppm] = 56.26 (d, $^4J_{C,P}$ = 2.2 Hz, C^-), 117.40 (C≡N), 119.93 (C≡N), 123.70 (d, $^1J_{C,P}$ = 95.1 Hz, PC_{Ph}), 128.45 (d, $J_{C,P}$ = 1.6 Hz), 128.75 (d, $J_{C,P}$ = 2.3 Hz), 129.22 (d, $J_{C,P}$ = 13.1 Hz), 129.40 (d, $J_{C,P}$ = 4.5 Hz), 129.95 (probably one branch of the doublet for PC̲=CH, the other one being covered by the signal at 129.22), 133.08 (d, $^2J_{C,P}$ = 9.7 Hz), 133.08 (d, $^4J_{C,P}$ = 3.3 Hz), 134.87 (d, $J_{C,P}$ = 10.1 Hz), 135.88 (d, $J_{C,P}$ = 10.6 Hz), 148.25 (d, $^2J_{C,P}$ = 5.0 Hz, C=C̲H), 176.93 (d, $^3J_{C,P}$ = 4.1 Hz, CH-C̲=O). – ^{31}P NMR ($CDCl_3$): δ [ppm] = 22.80.

UV (acetonitrile, $4\cdot10^{-4}$ mol L^{-1}): λ_{max} [nm] (lg ε) = 215 (3.56), 390 (2.94). – IR (KBr): $\tilde{\nu}$ [cm^{-1}] = 2198 (s), 2175 (m), 1602 (m), 1543 (s), 1438 (m), 1376 (m), 1102 (m), 750 (m), 705 (m), 690 (m), 583 (m). – HRMS (MALDI-TOF): *m/z* = 457.14658 $[M + H]^+$; calcd. 457.14698. – Anal. for $C_{30}H_{21}N_2OP$ (456.48 g/mol): calcd. C 78.94, H 4.64, N 6.14; found C 79.09, H 4.73, N 6.01.

2.1.5. Cyano-1-(4-cyano-2,3,5,6-tetrafluorophenyl)-2-oxo-4-phenyl-4-(triphenylphosphonio)but-3-en-1-ide ((*E*)- and (*Z*)-3f)

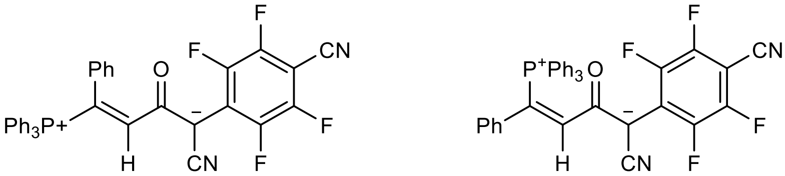

From triflamide **1** (R = Ph; 361 mg, 1.03 mmol), PPh_3 (262 mg, 1.00 mmol) and 4-cyanomethyl-2,3,5,6-tetrafluorobenzonitrile (214 mg, 1.00 mmol); 1 hour. Yield: 562 mg (0.93 mmol, 93%) of a deep orange mixture of diastereoisomers, E/Z = 87:13, which could be separated by solvent diffusion crystallization.

(*E*)-3f: 480 mg (0.80 mmol, 80%); dark red needle-shaped crystals, m.p. 190 °C.

^{1}H NMR ($CDCl_3$, 500.13 MHz): δ [ppm] = 7.11–7.14 (m, 2H, H_{Ph}), 7.17–7.21 (m, 2H, H_{Ph}), 7.27–7.31 (m, 1H, H_{Ph}), 7.48 (d, $^3J_{H,P}$ = 22.79 Hz, 1H, C=CH), 7.53–7.68 (m, 12H, H_{Ph}), 7.80–7.85 (m, 3H, H_{Ph}). – ^{13}C NMR ($CDCl_3$, 100.62 MHz): δ [ppm] = 65.55 (d, $^4J_{C,P}$ = 2.8 Hz, C^-), 108.96 (C≡N), 117.42 (d, $^1J_{C,P}$ = 87.9 Hz, PC_{Ph}), 122.69 (C≡N), 124.04 (d, $^1J_{C,P}$ = 73.1 Hz, P<u>C</u>=CH), 124.62, 124.82 (d, *J* = 1.4 Hz, C_{Ar}), 128.58, 129.46 (d, *J* = 2.3 Hz, C_{Ar}), 130.03 (d, *J* = 4.5 Hz, C_{Ar}), 130.34 (d, $^3J_{C,P}$ = 12.7 Hz, C_{PPh}), 131.17 (d, *J* = 9.5 Hz, C_{Ar}), 134.80 (d, $^2J_{C,P}$ = 10.0 Hz, C_{PPh}), 135.43 (d, $^4J_{C,P}$ = 3.0 Hz, C_{PPh}); 142.5, 144.5, 145.7, 147.8 (4 weak m, CF and <u>C</u>CF), 155.65 (d, $^2J_{C,P}$ = 7.3 Hz, C=<u>C</u>H), 176.19 (d, $^3J_{C,P}$ = 17.7 Hz, CH-<u>C</u>=O). – ^{31}P NMR ($CDCl_3$): δ [ppm] = 24.55. – ^{19}F NMR ($CDCl_3$): δ [ppm] = -131.64 (m), -138.65 (m). – IR (KBr): $\tilde{\nu}$ [cm^{-1}] = 2234 (w-m), 2171 (m), 1645 (m), 1561 (m), 1480 (s), 1438 (m), 1420 (m), 1276 (m), 1105 (m), 694 (m). – HRMS (MALDI-TOF): *m/z* = 605.14012 $[M + H]^+$; calcd. 605.14059. – Anal. for $C_{36}H_{21}F_4N_2OP$ (604.54 g/mol): calcd. C 71.52, H 3.5, N 4.63; found C 71.81, H 3.91, N 4.41.

(*Z*)-3f: 70 mg (12%); light yellow blocs, m.p. 214 °C.

^{1}H NMR ($CDCl_3$, 500.13 MHz, *T* = 294 K): δ [ppm] = 6.84–6.86 (m, 2H, H_{Ph}), 7.03–7.07 (m, 2H, H_{Ph}), 7.11–7.17 (m, 1 H), 7.26–7.57 (two broad unstructured m, 15H, H_{Ph}), 8.22 (d, $^3J_{H,P}$ = 40.03 Hz, 1H, C=CH). – ^{13}C NMR ($CDCl_3$, 100.62 MHz, *T* = 294 K): δ [ppm] = 108.64 (C≡N), 122.46 (C≡N), 128.24 (d, $^1J_{C,P}$ = 85.1 Hz, PC_{Ph}), 128.29 (d, *J* = 1.2 Hz, C_{Ar}), 128.35 (d, *J* = 2.2 Hz, C_{Ar}), 128.94 (d, $^3J_{C,P}$ = 13.2 Hz, C_{PPh}), 129.60 (d, *J* = 4.6 Hz, C_{Ar}), 130.84 (d, $^1J_{C,P}$ = 56.7 Hz, P<u>C</u>=CH), 132.41 (d, $^4J_{C,P}$ = 2.9 Hz, C_{PPh}), 132.80 (d, $^2J_{C,P}$ = 9.0 Hz, C_{PPh}), 136.47 (d, $J_{C,P}$ = 11.6 Hz), 149.50 (d, $^2J_{C,P}$ = 5.5 Hz, C=<u>C</u>H), 170.09 (d, $^3J_{C,P}$ = 3.7 Hz, CH-<u>C</u>=O). – ^{31}P NMR ($CDCl_3$): δ [ppm] = 19.26. – ^{19}F NMR ($CDCl_3$): δ [ppm] = -134.45 (m), -136.25 (m).

IR (KBr): $\tilde{\nu}$ [cm^{-1}] = 2176 (m), 1644 (m), 1531 (s), 1482 (s), 1438 (m), 694 (m), 534 (m), 504 (m). – HRMS (MALDI): *m/z* = 604.13036 [M]$^+$; calcd. 604.13276. – Anal. for $C_{36}H_{21}F_4N_2OP$ (604.54 g/mol): calcd. C 71.52, H 3.50, N 4.63; found C 71.52, H 3.51, N 4.72.

2.1.6. (*E*)-5-(3-(4-Chlorophenyl)-3-(triphenylphosphonio)acryloyl)-1,3-dimethyl-2,4,6-trioxohexahydropyrimidin-5-ide ((*E*)-3g)

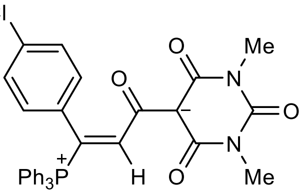

From triflamide **1** (R = 4-chlorophenyl; 399 mg, 1.03 mmol), PPh_3 (262 mg, 1.00 mmol) and *N,N*-dimethylbarbituric acid (156 mg, 1.00 mmol); 1 hour. Yield: 482 mg (0.83 mmol, 83%) of a pale yellow solid, m.p. 212 °C. Only the *E* isomer was observed.

^{1}H NMR ($CDCl_3$, 400.13 MHz): δ [ppm] = 3.25 (s, 6H, NCH_3), 6.88 (dd, $^3J_{H,H}$ = 8.0 Hz, 2 H_{Ar}), 7.06 (broadened d, $^3J_{H,H}$ = 8.0 Hz, 2 H_{Ar}), 7.60–7.70 (m, 12 H, H_{Ph}), 7.69 (d, $^3J_{H,P}$ = 23.11 Hz, 1H, C=CH), 7.77–7.82 (m, 3 H). – ^{13}C NMR ($CDCl_3$, 100.62 MHz): δ [ppm] = 27.08 (NCH_3), 96.80 (d, $^4J_{C,P}$ = 2.6 Hz, C^-), 112.50 (d, $^1J_{C,P}$ = 81.0 Hz, P$\underline{C}$=CH), 118.10 (d, $^1J_{C,P}$ = 88.2 Hz, PC_{Ph}), 128.68 (d, $J_{C,P}$ = 1.8 Hz), 130.08 (d, $^3J_{C,P}$ = 12.7 Hz), 130.25, 132.09 (d, $J_{C,P}$ = 4.3 Hz), 134.87 (d, $^2J_{C,P}$ = 10.0 Hz), 134.98 (d, $J_{C,P}$ = 2.9 Hz), 135.14 (d, $^4J_{C,P}$ = 3.0 Hz), 153.03 (N,N-C=O), 162.19 (d, $^2J_{C,P}$ = 7.7 Hz, C=$\underline{C}$H), 163.83 (N-C=O), 183.77 (d, $^3J_{C,P}$ = 17.6 Hz, CH-$\underline{C}$=O). ^{31}P NMR ($CDCl_3$): δ [ppm] = 24.38.

IR (KBr): $\tilde{\nu}$ [cm^{-1}] = 1701 (m), 1647 (vs), 1612 (s), 1482 (m), 1415 (s), 1387 (m), 1107 (m), 724 (m). – HRMS (MALDI): *m/z* = 581.13791 [M + H]$^+$; calcd. 581.13699. – Anal. for $C_{33}H_{26}ClN_2O_4P$ (581.00 g/mol): calcd. C 68.22, H 4.51, N 4.82; found C 68.43, H 4.38, N 4.81.

2.1.7. 4-(4-Chlorophenyl)-1,1-dicyano-2-oxo-4-(triphenylphosphonio)but-3-en-1-ide ((*E*)- and (*Z*)-3h)

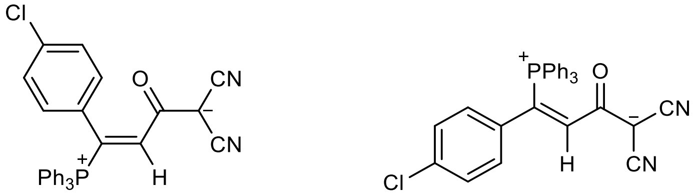

From triflamide **1** (R = 4-chlorophenyl; 399 mg, 1.03 mmol), PPh_3 (262 mg, 1.00 mmol) and malononitrile (66 mg, 1.00 mmol); 2 hours. Yield: dark yellow mixture of diastereoisomers (388 mg, 0.79 mmol, 79%, which were not separated. M.p. 283 °C (decomp.). The *E*/*Z* ratio was determined from the ^{1}H and ^{31}P NMR spectra and exhibited a small solvent-dependent variation; values between 54:46 and 60:40 were found.

^{1}H NMR ($CDCl_3$, 500.13 MHz): *E*:*Z* = 54:46, δ [ppm] = 6.77 and 7.05 (AA'BB'X spin system, X = P, $^3J_{H,H}$ = 8.43 Hz, 2H_{Ar}, *E*), 6.99 and 7.19 (AA'X,X', $^3J_{H,H}$ = 8.43 Hz, 2H_{Ar}, *Z*), 7.24 (d, $^3J_{H,P}$ = 22.21 Hz,

1H, C=CH, *E*), 7.36–7.47 (m, 13H, H_{Ph}), 7.50–7.61 (m, 9H, H_{Ph}), 7.64–7.69 (m, 5H, H_{Ph}), 7.82–7.87 (m, 3H, H_{Ph}), 7.89 (d, $^3J_{H,P}$ = 38.26 Hz, 1H, C=CH, *Z*). – ^{1}H NMR (DMSO-d_6, see spectrum) *E*:*Z* = 60:40; olefinic proton signal of *Z* isomer covered by aromatic proton signals. – ^{13}C NMR ($CDCl_3$, 100.62 MHz) of *E*/*Z* mixture; δ [ppm] = 52.55 (d, $^4J_{C,P}$ = 2.2 Hz, C^-), 56.55 (d, $^4J_{C,P}$ = 2.4 Hz, C^-), 116.81 (d, $^1J_{C,P}$ = 88.0 Hz, PC_{Ph}); 117.20–124.62 (4 C≡N, 4 C_{sp2}), 128.23–136.28 (14 C), 148.68 (d, $^2J_{C,P}$ = 5.0 Hz, C=$\underline{\text{C}}$H), 154.61 (d, $^2J_{C,P}$ = 7.6 Hz, C=$\underline{\text{C}}$H), 176.57 (d, $^3J_{C,P}$ = 4.0 Hz, CH-$\underline{\text{C}}$=O), 182.23 (d, $^3J_{C,P}$ = 17.6 Hz, CH-$\underline{\text{C}}$=O). – ^{31}P NMR (DMSO-d_6): *E*/*Z* = 55:45, δ [ppm] = 22.50 ($^+PPh_3$, *Z*), 24.87 ($^+PPh_3$, *E*).
IR (KBr): $\tilde{\nu}$ [cm^{-1}] = 2198 (s), 2175 (s), 1570 (s), 1547 (s), 1485 (m), 1438 (m), 1358 (m), 1105 (s), 723 (m), 692 (m), 541 (m), 514 (m). – HRMS (MALDI): *m/z* = 491.10746 [M + H]$^+$; calcd. 491.10800. – Anal. for $C_{30}H_{20}ClN_2OP$ (490.93 g/mol): calcd. C 73.40, H 4.11, N 5.71; found C 73.41, H 4.10, N 5.77.

2.1.8. 5-(4,4-Dimethyl-3-(triphenylphosphonio)pent-2-enoyl)-1,3-dimethyl-2,4,6-trioxohexahydropyrimidin-5-ide ((*E*)- and (Z)-3i)

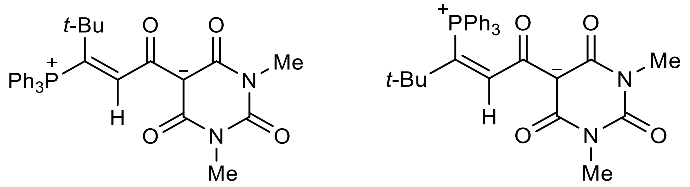

From triflamide **1** (R = *tert*-butyl; 343 mg, 1.03 mmol), PPh_3 (262 mg, 1.00 mmol) and *N*,*N*-dimethylbarbituric acid (156 mg, 1.00 mmol), 6 hours. A mixture of diastereoisomers was obtained (490 mg, 0.93 mmol, 93%, *E*/*Z* = 2:1), which were not separated. M.p. 204 °C.
^{1}H NMR ($CDCl_3$, 500.13 MHz): δ [ppm] for *E* isomer: 1.17 (s, 9H, C$\underline{\text{Me}}_3$), 3.27 (s, 6H, NMe), 7.18 (d, $^3J_{H,P}$ = 30.8 Hz, 1H, C=CH); for *Z* isomer: 1.13 (s, 9H, C$\underline{\text{Me}}_3$), 3.19 (s, 6H, NMe), 8.18 (d, $^3J_{H,P}$ = 46.06 Hz, 1H, C=CH); for both isomers: 7.45–7.99 (several m, all H_{Ph}). – ^{13}C NMR ($CDCl_3$, 100.62 MHz): δ [ppm] for *E* isomer: 27.19 (NMe), 31.33 (d, $^3J_{C,P}$ = 5.1 Hz, C$\underline{\text{Me}}_3$), 38.51 (d, $^2J_{C,P}$ = 11.0 Hz, $\underline{\text{C}}$Me$_3$), 96.51 (d, $^4J_{C,P}$ = 2.2 Hz, C^-), 119.07 (d, $^1J_{C,P}$ = 65.2 Hz, P$\underline{\text{C}}$=CH), 121.38 (d, $^1J_{C,P}$ = 85.9 Hz, PC_{Ph}), 130.02 (d, $^3J_{C,P}$ = 12.5 Hz, C_{PPh}), 134.51 (d, $^4J_{C,P}$ = 3.0 Hz, C_{PPh}), 134.93 (d, $^2J_{C,P}$ = 9.8 Hz, C_{PPh}), 153.16 (N,N-C=O), 163.46 (d, $^1J_{C,P}$ = 9.0 Hz, C=$\underline{\text{C}}$H), 186.80 (d, $^3J_{C,P}$ = 21.4 Hz, CH-$\underline{\text{C}}$=O); for *Z* isomer: 27.23 (NMe), 31.33 (d, $^3J_{C,P}$ = 3.6 Hz, C$\underline{\text{Me}}_3$), 39.19 (d, $^2J_{C,P}$ = 10.3 Hz, $\underline{\text{C}}$Me$_3$), 97.63 (C^-), 122.54 (d, $^1J_{C,P}$ = 88.0 Hz, PC_{Ph}), 125.69 (d, $^1J_{C,P}$ = 66.3 Hz, P$\underline{\text{C}}$=CH), 128.98 (d, $^3J_{C,P}$ = 12.7 Hz, C_{PPh}), 133.57 (d, $^4J_{C,P}$ = 3.1 Hz, C_{PPh}), 135.25 (d, $^2J_{C,P}$ = 10.0 Hz, C_{PPh}), 152.91 (N,N-C=O), 158.34 (d, $^1J_{C,P}$ = 6.9 Hz, C=$\underline{\text{C}}$H), 185.04 (d, $^3J_{C,P}$ = 7.4 Hz, CH-$\underline{\text{C}}$=O). – ^{31}P NMR ($CDCl_3$): δ [ppm] = 28.96 ($^+PPh_3$, *E*); 23.98 ($^+PPh_3$, *Z*). – IR (KBr): $\tilde{\nu}$ [cm^{-1}] = 1699 (m), 1643 (vs), 1552 (m), 1479 (m), 1414 (vs), 1385 (vs), 1103 (m), 759 (m), 730 (m), 693 (m), 517 (m). – HRMS (MALDI): *m/z* = 527.20926 [M + H]$^+$; calcd. 527.20997. – Anal. for $C_{31}H_{31}N_2O_4P$ (526.57 g/mol): calcd. C 70.71, H 5.93 N 5.32; found C 70.72, H 5.92, N 5.22.

2.1.9. (*E*)-1,3-Dimethyl-2,4,6-trioxo-5-(3-(triphenylphosphonio)hept-2-enoyl)hexahydropyrimidin-5-ide ((*E*)-3j)

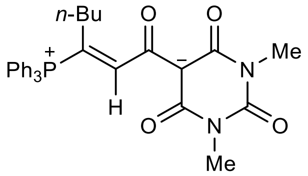

From triflamide **1** (R = *n*-Bu; 343 mg, 1.03 mmol), PPh_3 (262 mg, 1.00 mmol) and *N*,*N*-dimethylbarbituric acid (156 mg, 1.00 mmol); 2.5 hours. *E*-**3j** was obtained as a yellow solid (500 mg, 0.95 mmol, 95%), m.p. 220 °C.

^{1}H NMR ($CDCl_3$, 500.13 MHz): δ [ppm] = 0.54 (t, $^3J_{H,H}$ = 7.25 Hz, 3H, $CH_2C\underline{H}_3$), 1.00 (h, $^3J_{H,H}$ = 7.12 Hz, 2H, CH_2), 1.09–1.16 (m, 2H, CH_2), 2.40–2.49 (m, 2H, CH_2), 3.26 (s, 6H, NCH_3), 7.42 (d, $^3J_{H,P}$ = 25.24 Hz, 1H, C=CH), 7.63–7.68 (m, 6H, H_{Ph}), 7.77–7.84 (m, 9H, H_{Ph}). – ^{13}C NMR ($CDCl_3$, 100.62 MHz): δ [ppm] = 13.49 (CH_3), 22.48 (CH_2), 27.23 (NCH_3), 28.94 (d, $^2J_{C,P}$ = 11.0 Hz, CH_2), 31.98 (d, $^3J_{C,P}$ = 1.3 Hz, CH_2), 96.91 (d, $^4J_{C,P}$ = 3.4 Hz, C^-), 114.11 (d, $^1J_{C,P}$ = 74.4 Hz, P$\underline{C}$=CH), 118.78 (d, $^1J_{C,P}$ = 87.1 Hz, PC_{Ph}), 130.16 (d, $^3J_{C,P}$ = 12.5 Hz, C_{PPh}), 134.79 (d, $^2J_{C,P}$ = 10.0 Hz, C_{PPh}), 134.99 (d, $^4J_{C,P}$ = 3.0 Hz, C_{PPh}), 153.21 (N,N-C=O), 161.04 (d, $^2J_{C,P}$ = 7.4 Hz, C=$\underline{C}$H), 164.10 (N-C=O), 184.98 (d, $^3J_{C,P}$ = 19.8 Hz, CH-$\underline{C}$=O). – ^{31}P NMR ($CDCl_3$): δ [ppm] = 26.24.

IR (KBr): $\tilde{\nu}$ [cm^{-1}] = 1697 (w), 1642 (s), 1481 (m), 1437 (m), 1414 (s), 1387 (s), 1107 (m), 999 (m), 726 (s), 694 (m). – Anal. for $C_{31}H_{21}N_2O_4P$ (526.57 g/mol): calcd. C 70.71, H 5.93, N 5.32; found C 70.41, H 5.94, N 5.27.

2.1.10. 5-(3-Cyclopropyl-3-(triphenylphosphonio)acryloyl)-1,3-dimethyl-2,4,6-trioxohexahydropyrimidin-5-ide ((*E*)- and (*Z*)-3k)

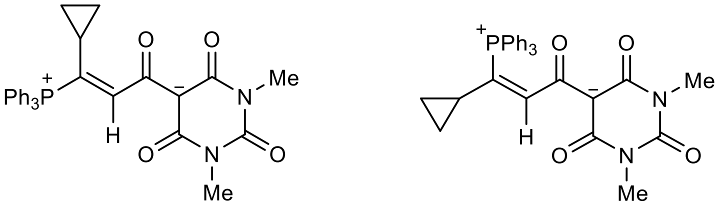

From triflamide **1** (R = cyclopropyl; 327 mg, 1.03 mmol), PPh_3 (262 mg, 1.00 mmol) and *N*,*N*-dimethylbarbituric acid (156 mg, 1.00 mmol); 2 hours. A mixture of diastereoisomers was obtained (454 mg, 0.89 mmol, 89%; *E*/*Z* = 83:17), which were not separated. M.p. 199 °C. –

^{1}H NMR ($CDCl_3$, 500.13 MHz): δ [ppm], *E* isomer: 0.62–0.83 (m, 4H, CH_2), 1.43–1.51 (m, 1H, CH), 3.30 (s, 6H, NCH_3), 7.44 (dd, $J_{H,P}$ = 23.87 Hz, 1.83 Hz, 1H, C=CH); *Z* isomer: 0.62–0.83 (m, 4H, CH_2), 1.00–1.07 (m, 1H, CH), 3.24 (s, 6H, NCH_3), 8.30 (dd, $J_{H,P}$ = 42.16 Hz, 1.62 Hz, 1H, C=CH); both isomers: 7.49-7.89 (several m, all H_{Ph}). – ^{13}C NMR ($CDCl_3$, 100.62 MHz): δ [ppm], *E* isomer: 8.66 (d, $^3J_{C,P}$ = 5.8 Hz, CH_2), 12.58 (d, $^2J_{C,P}$ = 14.9 Hz, CH), 27.27 (NCH_3), 96.66 (d, $^4J_{C,P}$ = 3.4 Hz, C^-), 114.26 (d, $^1J_{C,P}$ = 79.0 Hz, P$\underline{C}$=CH), 119.36 (d, $^1J_{C,P}$ = 88.0 Hz, PC_{Ph}), 130.11 (d, $^3J_{C,P}$ = 12.5 Hz, C_{PPh}), 134.87 (d, $^2J_{C,P}$ = 10.4 Hz, C_{PPh}), 134.96 (C_{PPh}), 153.15 (N,N-C=O, both isomers?), 154.43 (NC=O), 163.33 (d, $^2J_{C,P}$ = 9.9 Hz, C=$\underline{C}$H), 185.22 (d, $^3J_{C,P}$ = 18.8 Hz, CH-$\underline{C}$=O); *Z* isomer: 9.32 (d, $^3J_{C,P}$ = 4.8 Hz, CH_2),

17.65 (d, $^2J_{C,P}$ = 17.8 Hz, CH), 27.38 (NCH_3), 97.16 (C^-), 122.64 (d, $^1J_{C,P}$ = 92.1 Hz, C_{PPh}), 123.77 (d, $^1J_{C,P}$ = 81.0 Hz, P<u>C</u>=CH), 129.17 (d, $^3J_{C,P}$ = 12.9 Hz, PC_{Ph}), 133.33 (d, $^4J_{C,P}$ = 3.1 Hz, C_{PPh}), 134.05 (d, $^2J_{C,P}$ = 9.9 Hz, C_{PPh}), 153.15 (N,N-C=O, coinciding with *E* isomer?), 154.50 (N–C=O?), 164.11 (C=<u>C</u>H), 182.75 (d, $^3J_{C,P}$ = 15.3 Hz, CH-<u>C</u>=O). – ^{31}P NMR ($CDCl_3$): *δ* [ppm] = 25.39 ($^+PPh_3$, *E*); 24.21 ($^+PPh_3$, *Z*).

IR (KBr): $\tilde{\nu}$ [cm^{-1}] = 1698 (s), 1644 (s), 1615 (vs), 1480 (m), 1418 (s), 1387 (vs), 1107 (s), 998 (m), 757 (s), 724 (s), 696 (s). – HRMS (MALDI): *m/z* = 511.17820 $[M + H]^+$; calcd. 511.17867. – Anal. for $C_{30}H_{27}N_2O_4P$ (510.53 g/mol): calcd. C 70.58, H 5.33, N 5.49; found C 70.45, H 5.22, N 5.39.

2.1.11. (*E*)-1,1-Dicyano-5,5-dimethyl-2-oxo-4-(triphenylphosphonio)hex-3-en-1-ide ((*E*)-3l)

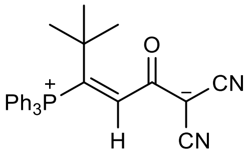

From triflamide **1** (R = *tert*-butyl; 343 mg, 1.03 mmol), PPh_3 (262 mg, 1.00 mmol) and malononitrile (66 mg, 1.00 mmol); 3 hours. ***E*-3l** was obtained as a light yellow solid (419 mg, 0.96 mmol, 96%), which decomposed at 272 °C.

^{1}H NMR ($CDCl_3$, 500.13 MHz): *δ* [ppm] = 1.28 (s, 9H, CH_3), 6.83 (d, $^3J_{H,P}$ = 30.77 Hz, 1H, C=CH), 7.69–7.74 (m, 6H, H_{Ph}), 7.80–7.85 (m, 9H, H_{Ph}). – ^{13}C NMR ($CDCl_3$, 100.62 MHz): *δ* [ppm] = 31.81 (d, $^3J_{C,P}$ = 4.9 Hz, C<u>C</u>H$_3$), 39.55 (d, $^2J_{C,P}$ = 10.2 Hz, <u>C</u>CH$_3$), 51.40 (d, $^4J_{C,P}$ = 2.6 Hz, C^-), 117.93 (C≡N), 120.09 (d, $^1J_{C,P}$ = 85.8 Hz, C_{PPh}), 122.36 (C≡N), 130.56 (d, $^3J_{CP}$ = 12.6 Hz, C_{PPh}), 131.87 (d, $^1J_{C,P}$ = 58.6 Hz, P<u>C</u>=CH), 134.66 (d, $^2J_{C,P}$ = 9.8 Hz, PC_{Ph}), 135.20 (d, $^4J_{C,P}$ = 3.0 Hz, C_{PPh}), 158.12 (d, $^5J_{C,P}$ = 8.5 Hz, C=<u>C</u>H), 185.45 (d, $^3J_{C,P}$ = 21.4 Hz, CH-<u>C</u>=O). – ^{31}P NMR ($CDCl_3$): *δ* [ppm] = 30.23.

IR (KBr): $\tilde{\nu}$ [cm^{-1}] = 2194 (s), 2166 (s), 1588 (s), 1565 (s), 1439 (m), 1346 (s), 1321 (m), 1100.62 (m), 721 (m), 694 (m), 522 (s). – Anal. for $C_{28}H_{25}N_2OP$ (436.49 g/mol): calcd. C 77.05, H 5.77 N 6.42; found C 77.30, H 6.00, N 6.22.

2.1.12. 1,1-Dicyano-2-oxo-4-(triphenylphosphonio)oct-3-en-1-ide ((*E*)- and (*Z*)-3m)

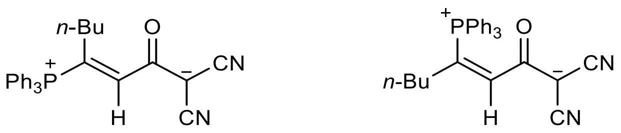

From triflamide **1** (R = *n*-butyl; 327 mg, 1.03 mmol), PPh_3 (262 mg, 1.00 mmol) and malononitrile (66 mg, 1.00 mmol); 2 hours. A mixture of diastereoisomers was obtained (388 mg, 0.93 mmol, 93%; *E*/*Z* = 83:17), which could be partly separated by solvent diffusion crystallization. The *E* isomer was obtained in pure form (310 mg, 0.71 mmol, 71%) as a light yellow solid, m.p. 185 °C (decomp.). The *Z* isomer was obtained only in admixture with **(*E*)-3m**.

(*E*)-3m: ^{1}H NMR ($CDCl_3$, 500.13 MHz): *δ* [ppm] = 0.61 (t, $^3J_{H,H}$ = 7.22 Hz, 3H, CH_3), 1.04–1.11 (m, 2H, CH_2), 1.13–1.22 (m, 2H, CH_2), 2.64–2.73 (m, 2H, CH_2), 7.02 (d, $^3J_{H,P}$ = 25.07 Hz, 1H, C=CH), 7.65–7.75 (m, 12H, H_{Ph}), 7.84–7.88 (m, 3H, H_{Ph}). – ^{13}C NMR ($CDCl_3$, 100.62 MHz): *δ* [ppm] = 13.42

(CH_3), 22.70 (CH_2), 29.64 (d, $^2J_{C,P}$ = 9.7 Hz, CH_2), 31.98 (d, $^3J_{C,P}$ = 1.3 Hz, CH_2), 53.33 (d, $^4J_{C,P}$ = 2.9 Hz, C^-), 117.19 (d, $^1J_{C,P}$ = 87.2 Hz, PC_{Ph}), 118.42 (C≡N), 121.78 (C≡N), 126.55 (d, $^1J_{C,P}$ = 68.8 Hz, P$\underline{C}$=CH), 130.65 (d, $^3J_{C,P}$ = 12.6 Hz, C_{PPh}), 134.52 (d, $^2J_{C,P}$ = 10.0 Hz, C_{PPh}), 135.66 (d, $^4J_{C,P}$ = 3.0 Hz, C_{PPh}), 151.64 (d, $^2J_{C,P}$ = 7.4 Hz, C=$\underline{C}$H), 182.21 (d, $^3J_{C,P}$ = 20.1 Hz, CH-$\underline{C}$=O). – ^{31}P NMR ($CDCl_3$): δ [ppm] = 26.92 ($^+PPh_3$, *E*); 26.09 ($^+PPh_3$, *Z*).

IR (KBr): $\tilde{\nu}$ [cm^{-1}] = 2195 (s), 2171 (s), 1611 (m), 1565 (s), 1438 (m), 1353 (m), 1107 (s), 995 (m), 723 (s), 691 (m), 526 (m). – HRMS (MALDI): *m/z* = 437.16654 $[M + H]^+$; calcd. 437.17828. – Anal. for $C_{28}H_{25}N_2OP$ (436.49 g/mol): calcd. C 77.05, H 5.77, N 6.42; found C 77.05, H 5.71, N 6.70.

2.1.13. (*E*)-1,1-Dicyano-4-cyclopropyl-2-oxo-4-(triphenylphosphonio)but-3-en-1-ide ((*E*)-3n)

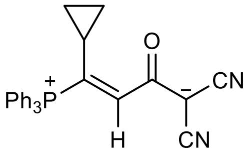

From triflamide **1** (R = cyclopropyl; 327 mg, 1.03 mmol), PPh_3 (262 mg, 1.00 mmol) and malononitrile (66 mg, 1.00 mmol), 2 hours. ***E*-3n** was obtained as a light yellow solid, which decomposed at 234 °C. Yield: 370 mg (0.88 mmol, 88%). The *Z* isomer was not observed. – ^{1}H NMR ($CDCl_3$, 500.13 MHz): δ [ppm] = 0.74–0.83 (m, 4H, CH_2), 1.53–1.58 (m, 1H CH), 6.86 (dd, $^3J_{H,P}$ = 24.04 Hz, $^4J_{H,H}$ =1.73 Hz, 1H, C=CH), 7.69–7.75 (m, 12H, H_{Ph}), 7.83–7.89 (m, 3H, H_{Ph}). – ^{13}C NMR ($CDCl_3$, 100.62 MHz): δ [ppm] = 9.12 (d, $^3J_{C,P}$ = 5.5 Hz, CH_2), 13.43 (d, $^2J_{C,P}$ = 14.4 Hz, CH), 52.46 (d, $^4J_{C,P}$ = 2.4 Hz, C^-), 117.65 (d, $^1J_{C,P}$ = 87.0 Hz, PC_{Ph}), 118.19 (C≡N), 122.18 (C≡N), 125.57 (d, $^1J_{C,P}$ = 72.2 Hz, P$\underline{C}$=CH), 130.54 (d, $^3J_{C,P}$ = 12.6 Hz, C_{PPh}), 134.58 (d, $^2J_{C,P}$ = 10.1 Hz, C_{PPh}), 135.58 (d, $^4J_{C,P}$ = 3.0 Hz, C_{PPh}), 153.91 (d, $^2J_{C,P}$ = 9.5 Hz, C=$\underline{C}$H), 183.49 (d, $^3J_{C,P}$ = 19.2 Hz, CH-$\underline{C}$=O). – ^{31}P NMR ($CDCl_3$): δ [ppm] = 26.48.

IR (KBr): $\tilde{\nu}$ [cm^{-1}] = 2195 (s), 2171 (s), 1614 (w), 1560 (s), 1438 (m), 1353 (m), 1107 (s), 753 (m), 727 (m), 692 (m), 523 (m). – Anal. for $C_{27}H_{21}N_2OP$ (420.45 g/mol): calcd. C 77.13, H 5.03, N 6.66; found C 77.22, H 4.88, N 6.80.

2.1.14. (*E*)-1,1-Dicyano-2-oxo-4-(triphenylphosphonio)but-3-en-1-ide ((*E*)-3o)

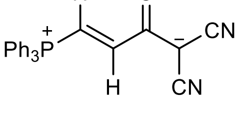

From *N*,3-Diphenyl-3-trimethylsilyl-*N*-triflyl-propiolamide (**1**, R = $SiMe_3$) (360 mg, 1.03 mmol), PPh_3 (262 mg, 1.00 mmol) and malononitrile (66 mg, 1.00 mmol); 2 hours at 0 °C. ***E*-3o** was obtained as an ocre solid (269 mg, 0.71 mmol, 71%), which decomposed at 220 °C. The *Z* isomer was not observed.

^{1}H NMR ($CDCl_3$, 500.13 MHz): δ [ppm] = 7.33 (dd, *J* = 20.27 and 16.27 Hz, 1H, C=CH), 7.55–7.65 (m, 6H, H_{Ph}, HC=C), 7.69–7.75 (m, 7H, H_{Ph}, HC=C), 7.84–7.87 (m, 3H, H_{Ph}). – ^{13}C NMR ($CDCl_3$, 100.62 MHz): δ [ppm] = 56.19 (C^-), 113.69 (d, $^1J_{C,P}$ = 87.9 Hz, P$\underline{C}$=CH), 117.54 (d, $^1J_{C,P}$ = 91.3 Hz,

PC_{Ph}), 118.38 (C≡N), 120.40 (C≡N), 130.79 (d, $^3J_{C,P}$ = 13.1 Hz, C_{PPh}), 133.91 (d, $^2J_{C,P}$ = 10.7 Hz, C_{PPh}), 135.86 (d, $^4J_{C,P}$ = 3.1 Hz, C_{PPh}), 152.49 (d, $^2J_{C,P}$ = 3.9 Hz, C=<u>C</u>H), 177.07 (d, $^3J_{C,P}$ = 18.6 Hz, CH-<u>C</u>=O).

^{31}P NMR ($CDCl_3$): δ [ppm] = 20.51.

IR (KBr): $\tilde{\nu}$ [cm^{-1}] = 2198 (s), 2178 (s), 1613 (m), 1548 (s), 1438 (m), 1365 (m), 1110 (m), 726 (m). – Anal. for $C_{24}H_{17}N_2OP$ (380.39 g/mol): calcd. C 75.78, H 4.50, N 7.36; found C 75.77, H 4.52, N 7.26.

3. X-ray crystal structure determinations

CCDC-1937388 (*E*-**3a**), -1937389 (*E*-**3b**), -1937390 (*E*-**3e**), and -1937391 (*Z*-**3e)** contains the crystallographic data for this paper. These data can be obtained free of charge from The Cambridge Crystallographic Data Centre via www.ccdc.cam.ac.uk/getstructures.

3.1. Crystallographic data for (*E*)-3a

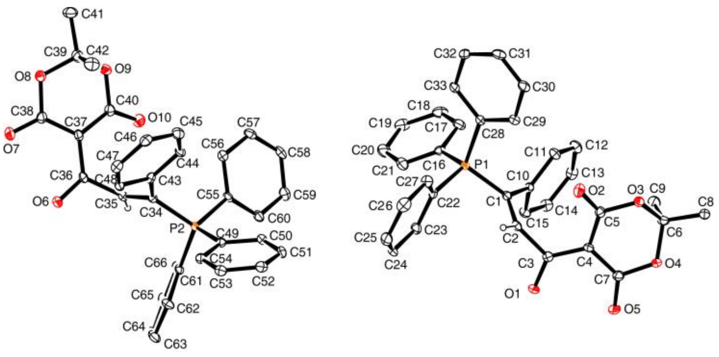

Data collection was performed on an Oxford Diffraction Rigaku instrument (SuperNova, Dual Source, Atlas CCD, Mo K_α radiation). Suitable crystals were obtained as pale yellow thin platelets by diffusion crystallization from CH_2Cl_2/diethyl ether at 8 °C. Data collection at 150(1) K. Structure solution: SHELXS-97 [2]; refinement: SHELXL-2014/6 [3]; molecule plot: ORTEP-3 for Windows [4]. The triclinic unit cell contains two symmetry-independent molecules.

Table: Crystallographic data for **(*E*)-3a**

Identification code	AF291	
Empirical formula	$C_{33}H_{27}O_5P$	
Formula weight	534.51	
Temperature	149.95(10) K	
Wavelength	0.71073 Å	
Crystal system	Triclinic	
Space group	*P*-1	
Unit cell dimensions	*a* = 8.6737(4) Å	α = 92.180(3)°
	b = 13.2993(4) Å	β = 98.750(4)°
	c = 23.5244(12) Å	γ = 90.155(3)°
Volume	2680.0(2) Å^3	
Z	4	
Density (calculated)	1.325 Mg/m^3	
Absorption coefficient	0.145 mm^{-1}	
F(000)	1120	
Crystal size	0.18 x 0.16 x 0.04 mm^3	
Theta range for data collection	2.839 to 25.681°.	
Index ranges	-10<=*h*<=8, -13<=*k*<=16, -24<=*l*<=28	
Reflections collected	26828	
Independent reflections	100.6283 [*R*(int) = 0.0597]	
Completeness to theta = 25.242°	99.8 %	
Absorption correction	Semi-empirical from equivalents	
Max. and min. transmission	1.00000 and 0.84204	

Refinement method	Full-matrix least-squares on F^2
Data / restraints / parameters	100.6283 / 0 / 707
Goodness-of-fit on F^2	1.194
Final R indices [I>2sigma(I)]	$R1$ = 0.1186, $wR2$ = 0.2714
R indices (all data)	$R1$ = 0.1393, $wR2$ = 0.2810
Extinction coefficient	n/a
Largest diff. peak and hole	0.652 and -0.433 e.Å^{-3}

3.2. Crystallographic data for (*E*)-3b·CH_2Cl_2

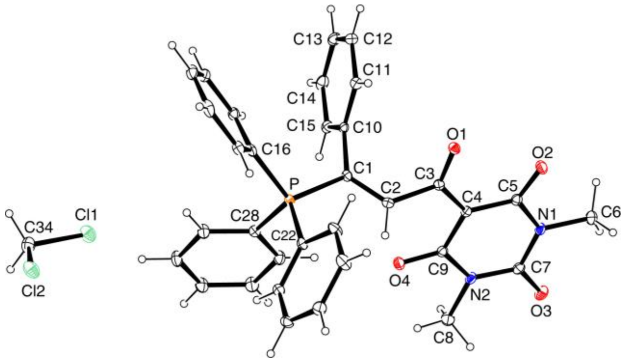

Data collection was performed on an Oxford Diffraction Rigaku instrument (SuperNova, Dual Source, Atlas CCD, Mo K_α radiation). Colorless crystals were obtained by diffusion crystallization from CH_2Cl_2/*n*-pentane at 8 °C. A crystal piece cut from a larger columnar crystal was used. Data collection at 150(1) K. Structure solution: SHELXS [2]; refinement: SHELXL-2014 [3]; molecule plot: ORTEP-3 for Windows [4].

Table: Crystallographic data for (***E***)**-3b** × CH_2Cl_2.

Identification code	CF323	
Empirical formula	$C_{34}H_{29}Cl_2N_2O_4P$	
Formula weight	631.46	
Temperature	150.00(10) K	
Wavelength	0.71073 Å	
Crystal system	Triclinic	
Space group	*P*-1	
Unit cell dimensions	a = 9.8481(7) Å	α = 111.210(7)°
	b = 11.3627(9) Å	β = 106.220(6)°
	c = 15.3689(11) Å	γ = 93.351(6)°
Volume	1514.8(2) $Å^3$	
Z	2	
Density (calculated)	1.384 Mg/m^3	
Absorption coefficient	0.310 mm^{-1}	
F(000)	656	
Crystal size	0.22 x 0.16 x 0.15 mm^3	
Theta range for data collection	2.866 to 25.681°.	
Index ranges	-11<=*h*<=12, -13<=*k*<=13, -18<=*l*<=17	
Reflections collected	11748	
Independent reflections	5732 [*R*(int) = 0.0278]	
Completeness to theta = 25.242°	99.9 %	
Absorption correction	Semi-empirical from equivalents	
Max. and min. transmission	1.00000 and 0.88639	
Refinement method	Full-matrix least-squares on F^2	
Data / restraints / parameters	5732 / 0 / 390	
Goodness-of-fit on F^2	1.069	
Final *R* indices [*I*>2sigma(*I*)]	*R*1 = 0.0455, *wR*2 = 0.1174	
R indices (all data)	*R*1 = 0.0588, *wR*2 = 0.1275	
Extinction coefficient	n/a	
Largest diff. peak and hole	0.473 and -0.462 e.$Å^{-3}$	

3.3. Crystallographic data for (*Z*)-3e

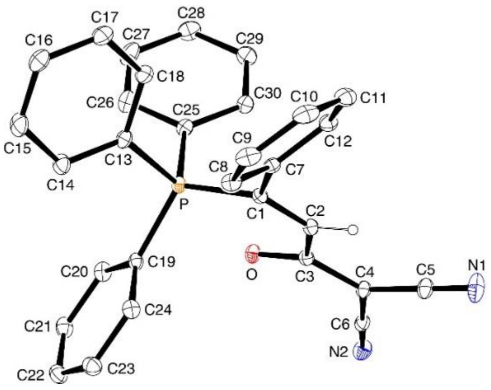

Data collection was performed on an Oxford Diffraction Rigaku instrument (SuperNova, Dual Source, Atlas CCD, Cu K_α radiation). Crystals were obtained as yellow needles by diffusion crystallization from CH_2Cl_2/n-pentane at 8 °C. A crystal piece cut from a larger needle was used. Data collection at 150(1) K. Structure solution: SIR-92 [5]; refinement: SHELXL-2014/6 [3]; molecule plot: ORTEP-3 for Windows [4].

Table: Crystallographic data of **(Z)-3e**.

Identification code	CF325	
Empirical formula	$C_{30}H_{21}N_2OP$	
Formula weight	456.46	
Temperature	150.00(10) K	
Wavelength	1.54184 Å	
Crystal system	Monoclinic	
Space group	*I*2/*a*	
Unit cell dimensions	*a* = 18.9715(3) Å	α = 90°
	b = 8.8404 (2) Å	β = 99.6230(14)°
	c = 28.5247(4) Å	γ = 90°
Volume	4716.72(13) Å^3	
Z	8	
Density (calculated)	1.286 Mg/m^3	
Absorption coefficient	1.228 mm^{-1}	
F(000)	1904	
Crystal size	0.18 x 0.15 x 0.14 mm^3	
Theta range for data collection	4.728 to 70.060°.	
Index ranges	-23<=*h*<=18, -10<=*k*<=10, -20<=*l*<=34	
Reflections collected	9147	
Independent reflections	4469 [*R*(int) = 0.0223]	
Completeness to theta = 67.684°	99.9 %	
Absorption correction	Semi-empirical from equivalents	
Max. and min. transmission	1.00000 and 0.92939	
Refinement method	Full-matrix least-squares on F^2	
Data / restraints / parameters	4469 / 0 / 307	
Goodness-of-fit on F^2	1.039	
Final *R* indices [*I*>2sigma(I)]	*R*1 = 0.0338, *wR*2 = 0.0858	
R indices (all data)	*R*1 = 0.0383, *wR*2 = 0.0899	
Extinction coefficient	n/a	
Largest diff. peak and hole	0.287 and -0.285 e.Å^{-3}	

3.4. Crystallographic data for (*E*)-3e·H_2O·CH_2Cl_2

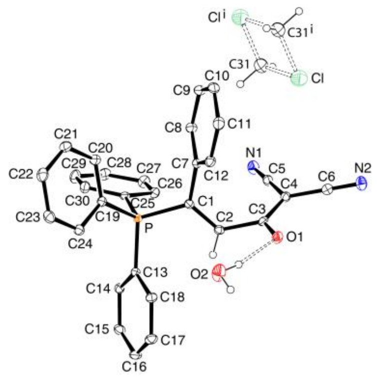

Data collection was performed on an Oxford Diffraction Rigaku instrument (SuperNova, Dual Source, Atlas CCD, Cu K_α radiation). Crystals were obtained as colorless prisms by diffusion crystallization from CH_2Cl_2/n-pentane at 8 °C. A crystal piece cut from a larger needle was used. Data collection at 150(2) K. Structure solution: SIR-92 [5]; refinement: SHELXL-2014/6 [3]; molecule plot: ORTEP-3 for Windows [4]. Hydrogen atoms were in geometrically calculated positions and included in the refinement using the riding model. The positions of the water hydrogen atoms were taken from a difference Fourier electron map and refined. The dichloromethane solvate molecule is disordered around a crystallographic inversion center.

Table: Crystallographic data of **(*E*)-3e**.

Identification code	CF326	
Empirical formula	$C_{30.50}H_{24}ClN_2O_2P$	
Formula weight	516.94	
Temperature	150(2) K	
Wavelength	1.54184 Å	
Crystal system	Triclinic	
Space group	*P*-1	
Unit cell dimensions	*a* = 9.7424(5) Å	α = 99.933(4)°
	b = 12.5721(5) Å	β = 110.790(5)°
	c = 12.8181(7) Å	γ = 110.690(4)°
Volume	1290.99(12) Å^3	
Z	2	
Density (calculated)	1.330 Mg/m^3	
Absorption coefficient	2.143 mm^{-1}	
F(000)	538	
Crystal size	0.18 x 0.15 x 0.14 mm^3	
Theta range for data collection	4.550 to 70.059°.	
Index ranges	-11<=*h*<=10, -14<=*k*<=15, -15<=*l*<=15	
Reflections collected	8900	
Independent reflections	4881 [*R*(int) = 0.0214]	
Completeness to theta = 67.684°	99.9 %	
Refinement method	Full-matrix least-squares on F^2	
Data / restraints / parameters	4881 / 0 / 350	
Goodness-of-fit on F^2	1.046	
Final *R* indices [*I*>2sigma(*I*)]	*R*1 = 0.0365, *wR*2 = 0.0924	
R indices (all data)	*R*1 = 0.0412, *wR*2 = 0.0955	
Extinction coefficient	n/a	
Largest diff. peak and hole	0.679 and -0.584 e.Å^{-3}	

3. References

1. Fiore, V. A.; Maas, G. *Tetrahedron* **2019**, *75*, 3586–3595. DOI: 10.100.626/j.tet.2019.05.027.
2. SHELXS97: Sheldrick, G. M. *Acta Crystallogr.* **2008**, *A64*, 112–122.
3. SHELXL-2014: Sheldrick, G. M. Crystal Structure Refinement with SHELXL. *Acta Crystallogr.* **2015**, *C71*, 3–8.
4. ORTEP-3 for Windows: Farrugia, L. J. *J. Appl. Crystallogr.* **2012**, *45*, 849–854.
5. SIR97: Altomare, A.; Burla, M. C.; Camalli, M.; Cascarano, G. L.; Giacovazzo, C.; Guagliardi, A.; Moliterni, A. G. G.; Polidori, G.; Spagna, R. *J. Appl. Cryst.* **1999**, *32*, 115–119.

4. Spectra of betaines 3 (^{1}H NMR, ^{13}C NMR, ^{19}F, ^{31}P NMR, IR)

4.1. Betaine (*E*)-3a

^{1}H NMR

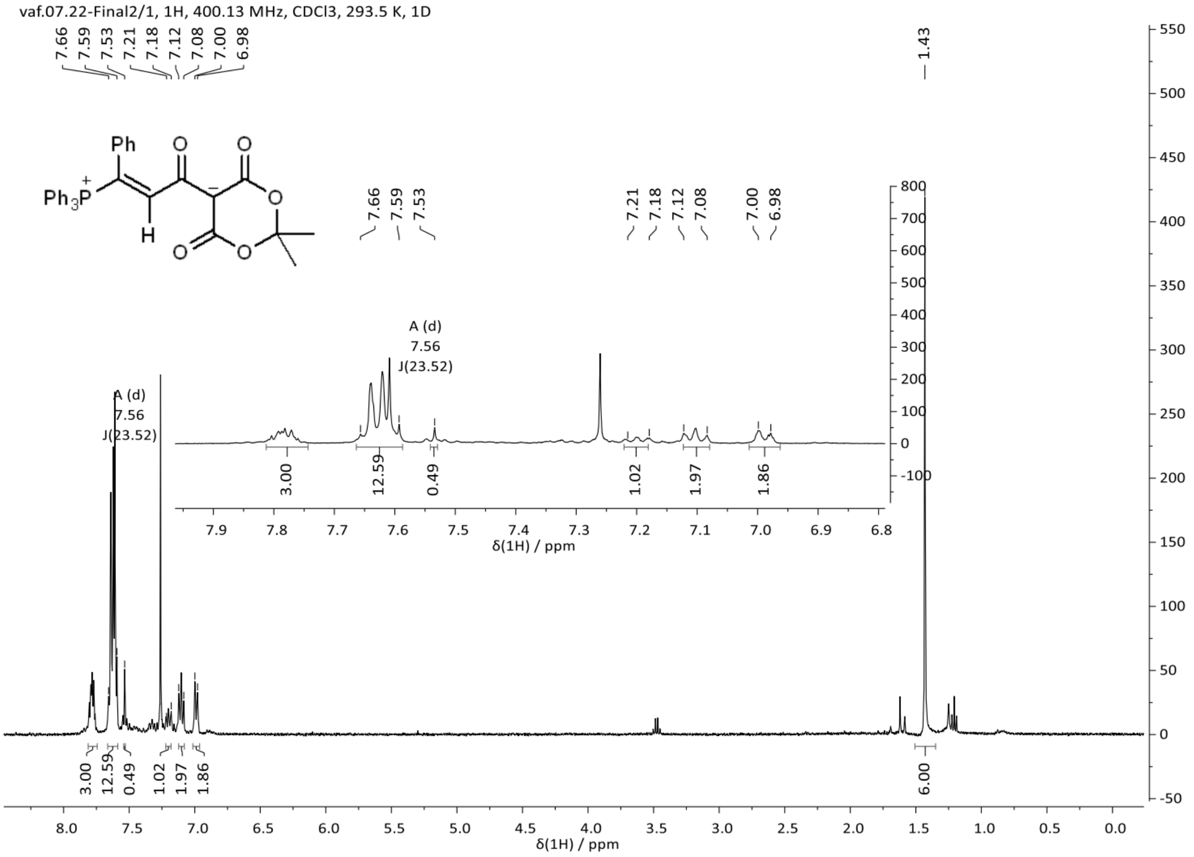

^{13}C NMR

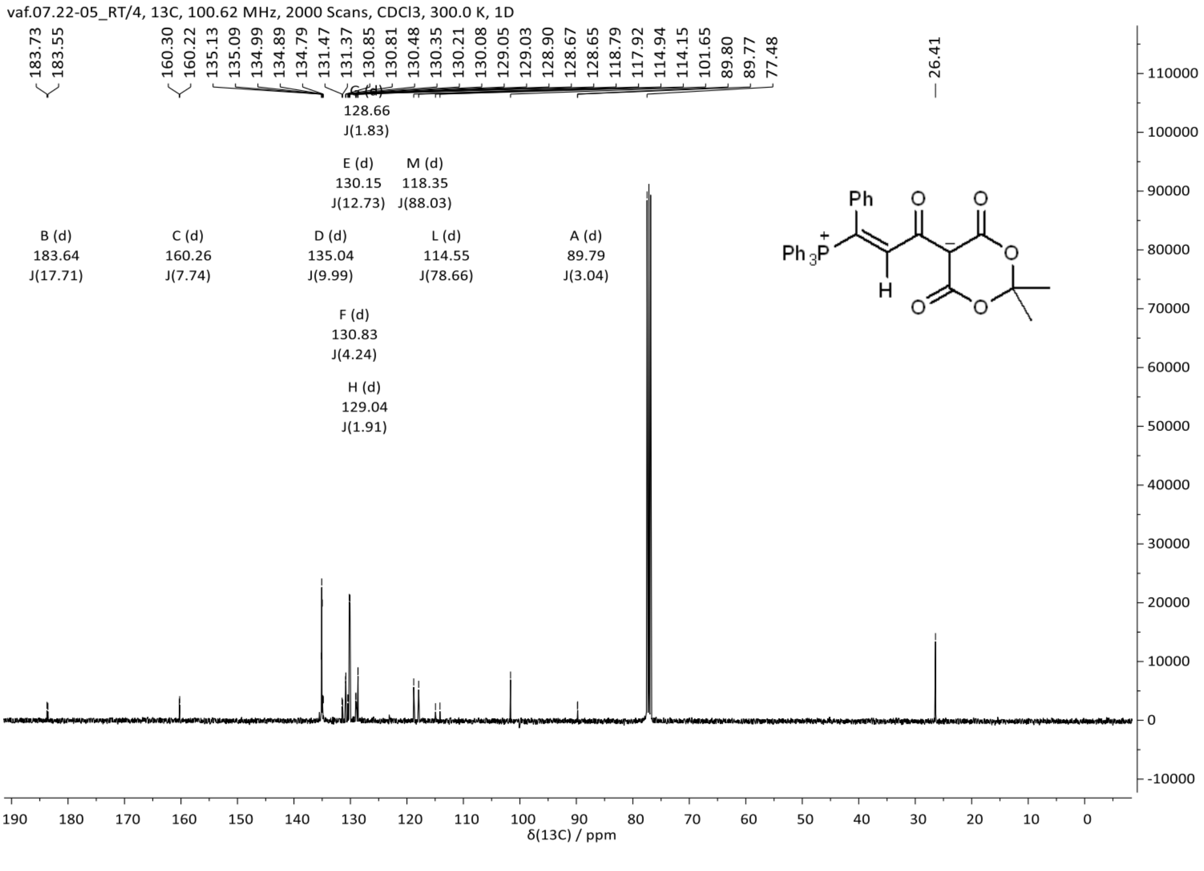

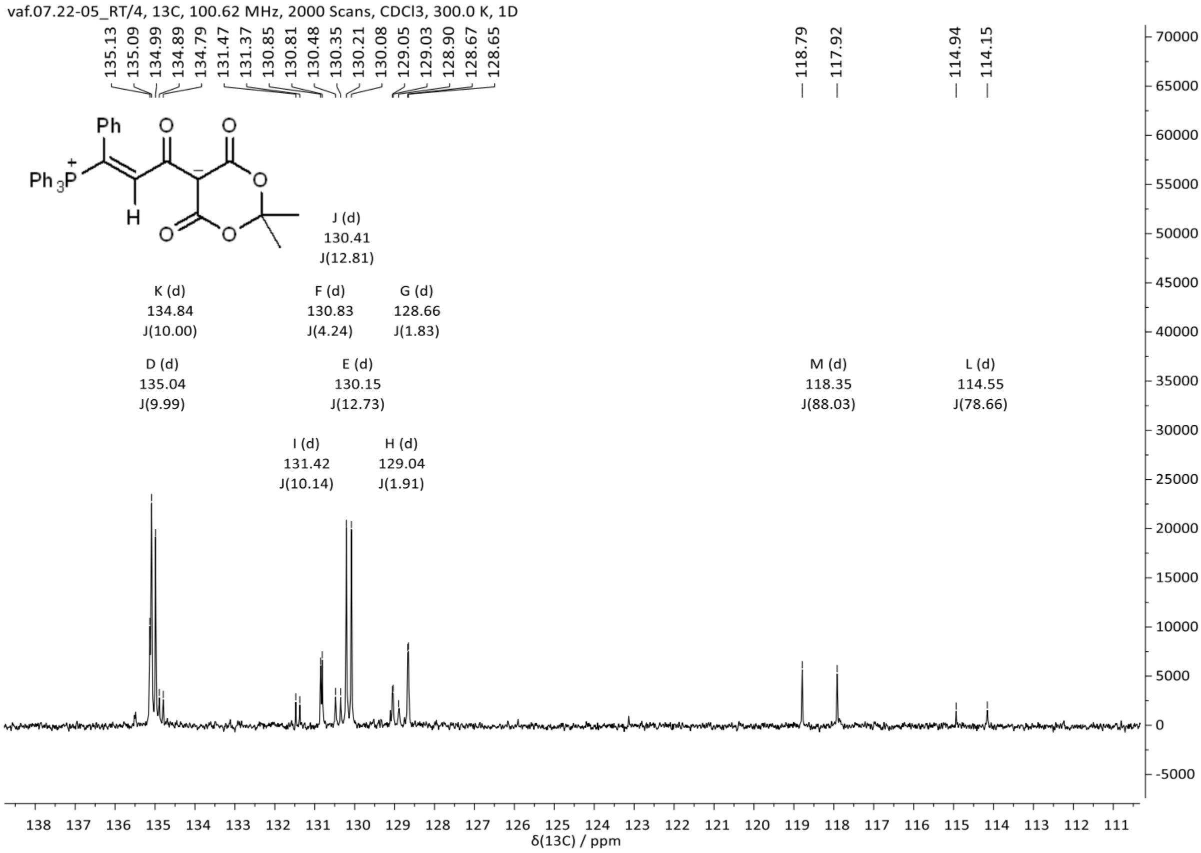
vaf.07.22-05_RT/4, 13C, 100.62 MHz, 2000 Scans, CDCl3, 300.0 K, 1D
135.13 135.09 134.99 134.89 134.79 131.47 131.37 130.85 130.81 130.48 130.35 130.21 130.08 129.05 129.03 128.90 128.67 128.65
118.79 117.92 114.94 114.15
Ph
Ph3P
H
O
J (d) 130.41 J(12.81)
K (d) 134.84 J(10.00)
F (d) 130.83 J(4.24)
G (d) 128.66 J(1.83)
D (d) 135.04 J(9.99)
E (d) 130.15 J(12.73)
M (d) 118.35 J(88.03)
L (d) 114.55 J(78.66)
I (d) 131.42 J(10.14)
H (d) 129.04 J(1.91)
δ(13C) / ppm

^{31}P NMR

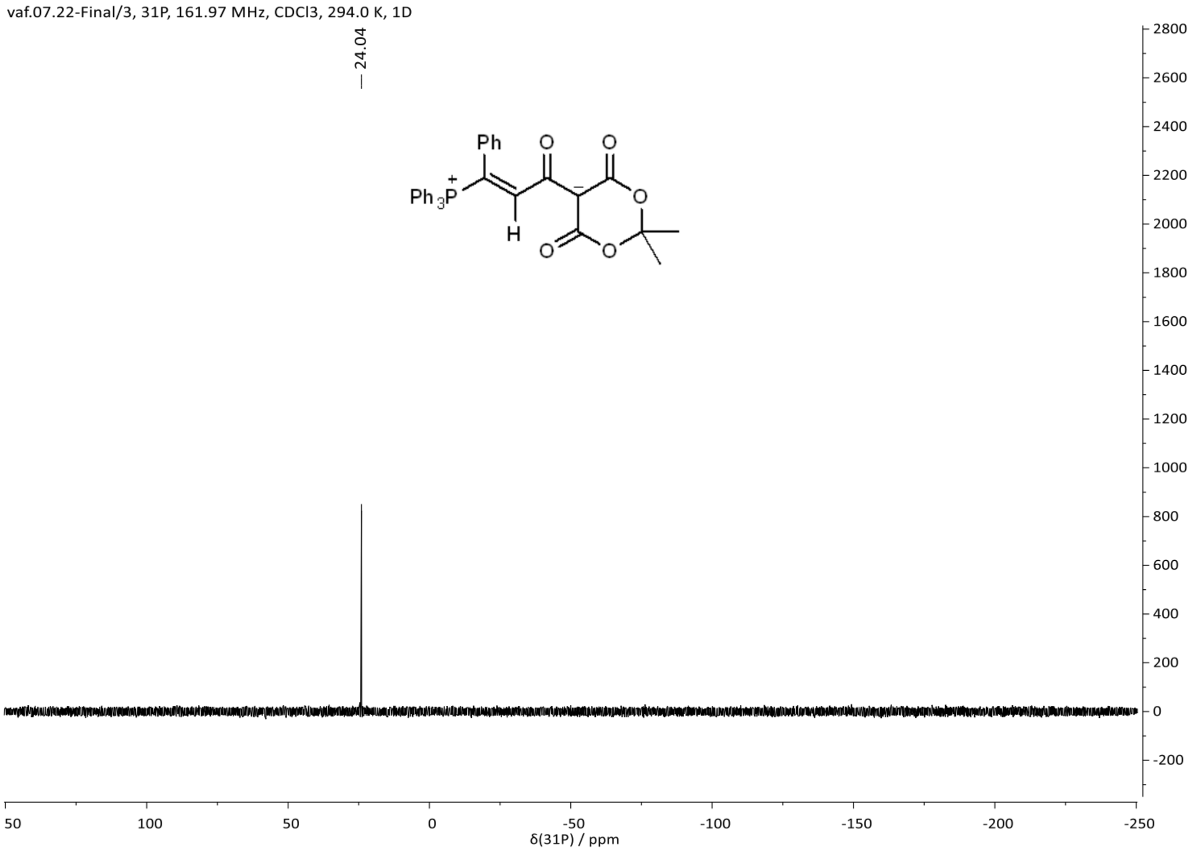
vaf.07.22-Final/3, 31P, 161.97 MHz, CDCl3, 294.0 K, 1D
24.04
Ph
Ph3P
H
O
δ(31P) / ppm

IR

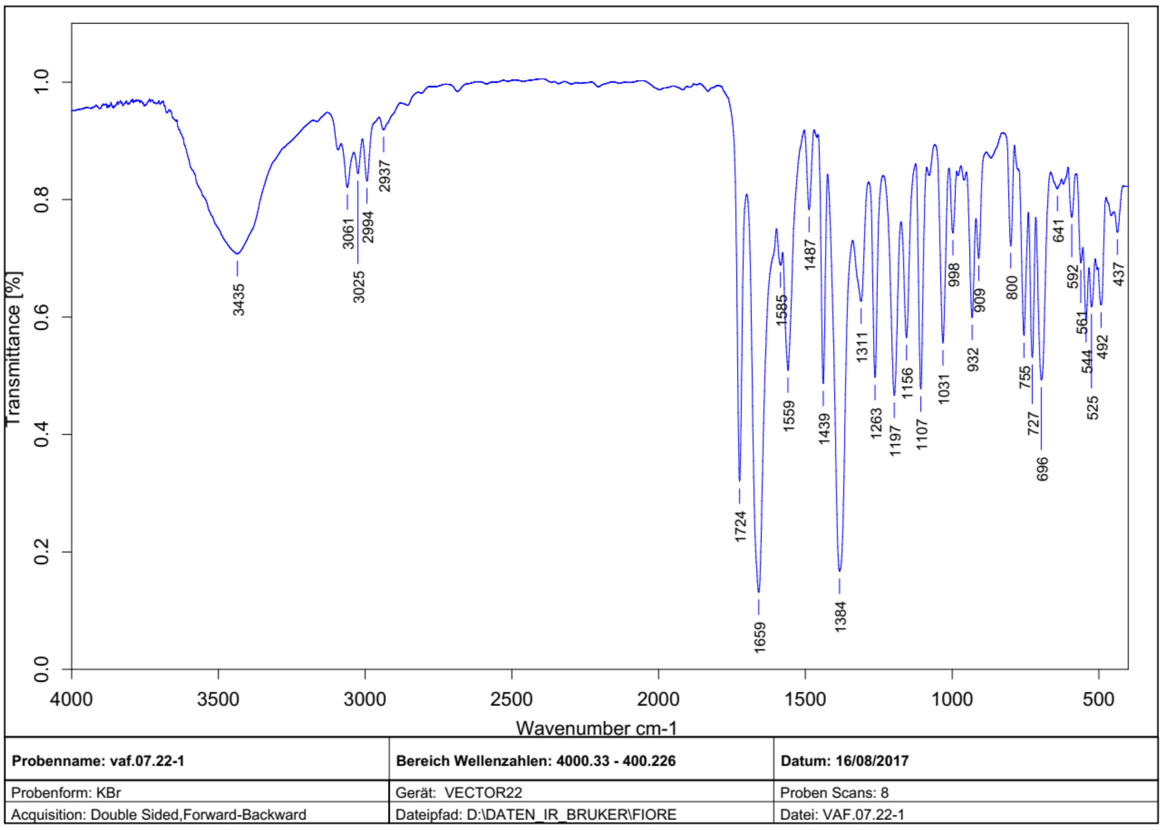

Probenname: vaf.07.22-1	Bereich Wellenzahlen: 4000.33 - 400.226	Datum: 16/08/2017
Probenform: KBr	Gerät: VECTOR22	Proben Scans: 8
Acquisition: Double Sided,Forward-Backward	Dateipfad: D:\DATEN_IR_BRUKER\FIORE	Datei: VAF.07.22-1

4.2. Betaines (*E*)- and (*Z*)-3b

***(E)*-3b**

^{1}H NMR

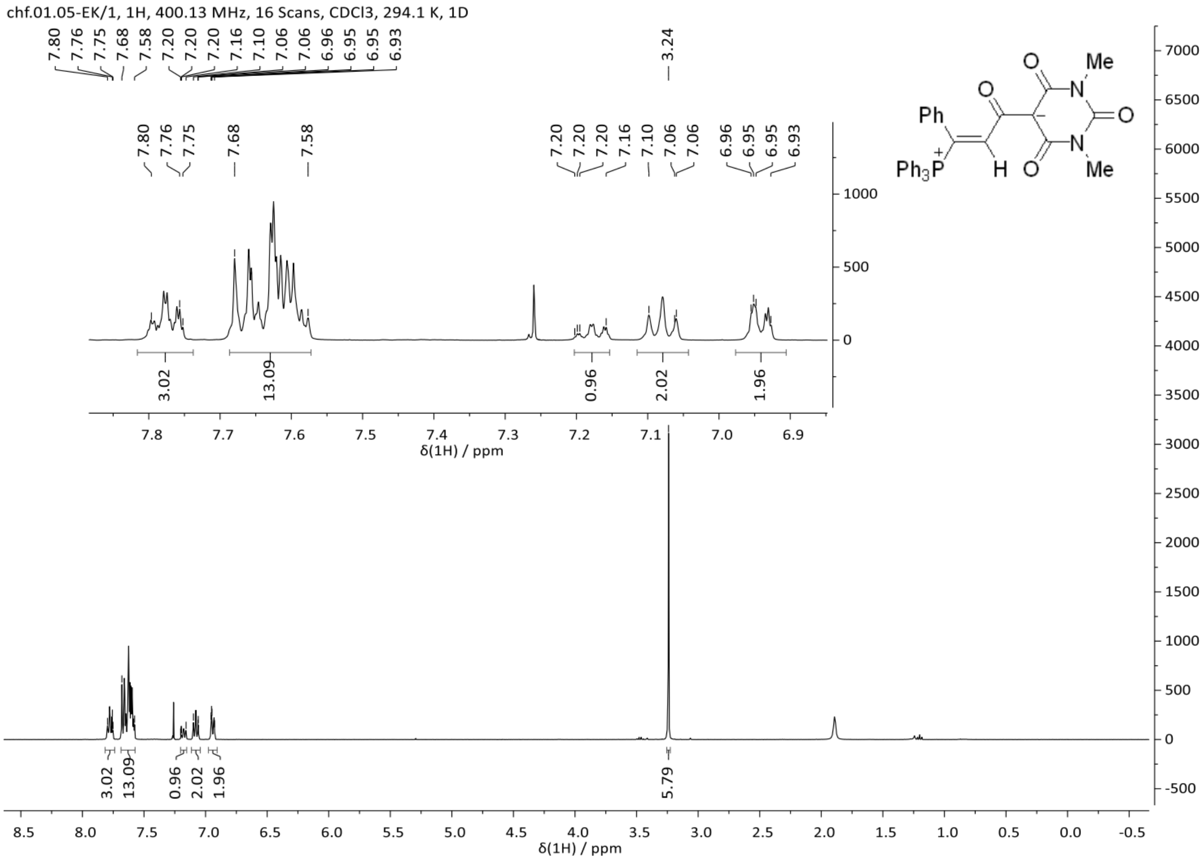

^{13}C NMR

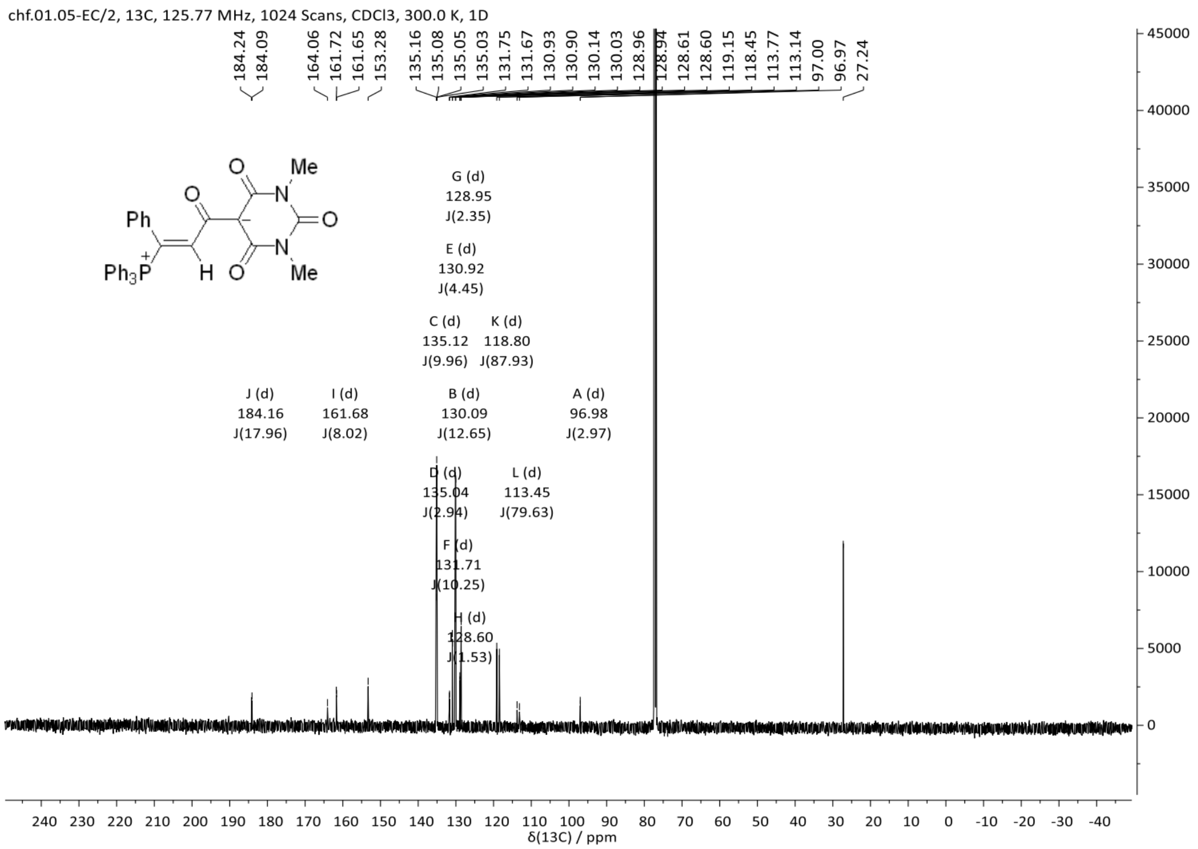

chf.01.05-EC/2, 13C, 125.77 MHz, 1024 Scans, CDCl3, 300.0 K, 1D
184.24
184.09
164.06
161.72
161.65
153.28
135.16
135.08
135.05
135.03
131.75
131.67
130.93
130.90
130.14
130.03
128.96
128.94
128.61
128.60
119.15
118.45
113.77
113.14
97.00
96.97
27.24
G (d) 128.95 J(2.35)
E (d) 130.92 J(4.45)
C (d) 135.12 J(9.96)
K (d) 118.80 J(87.93)
J (d) 184.16 J(17.96)
I (d) 161.68 J(8.02)
B (d) 130.09 J(12.65)
A (d) 96.98 J(2.97)
D (d) 135.04 J(2.94)
L (d) 113.45 J(79.63)
F (d) 131.71 J(10.25)
H (d) 128.60 J(1.53)
δ(13C) / ppm

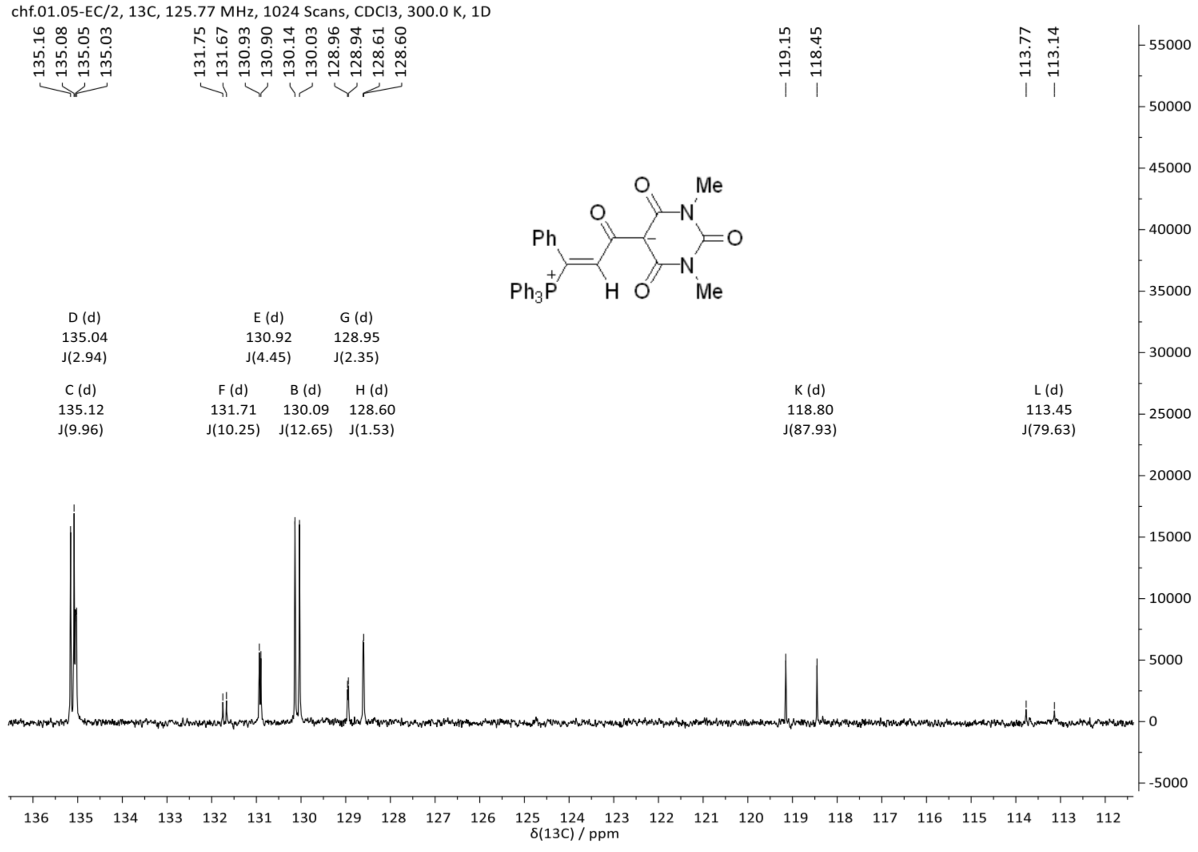

chf.01.05-EC/2, 13C, 125.77 MHz, 1024 Scans, CDCl3, 300.0 K, 1D
135.16
135.08
135.05
135.03
131.75
131.67
130.93
130.90
130.14
130.03
128.96
128.94
128.61
128.60
119.15
118.45
113.77
113.14
D (d) 135.04 J(2.94)
E (d) 130.92 J(4.45)
G (d) 128.95 J(2.35)
C (d) 135.12 J(9.96)
F (d) 131.71 J(10.25)
B (d) 130.09 J(12.65)
H (d) 128.60 J(1.53)
K (d) 118.80 J(87.93)
L (d) 113.45 J(79.63)
δ(13C) / ppm

^{31}P NMR

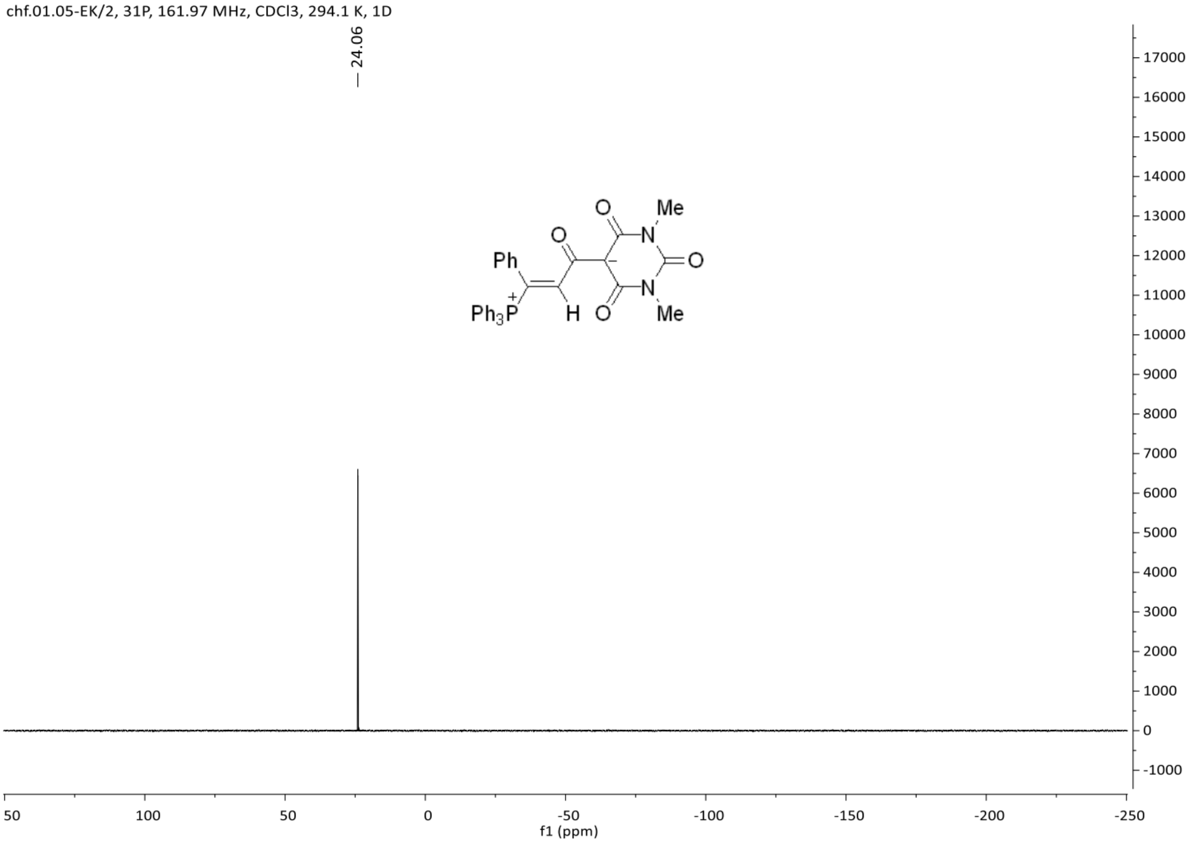

IR

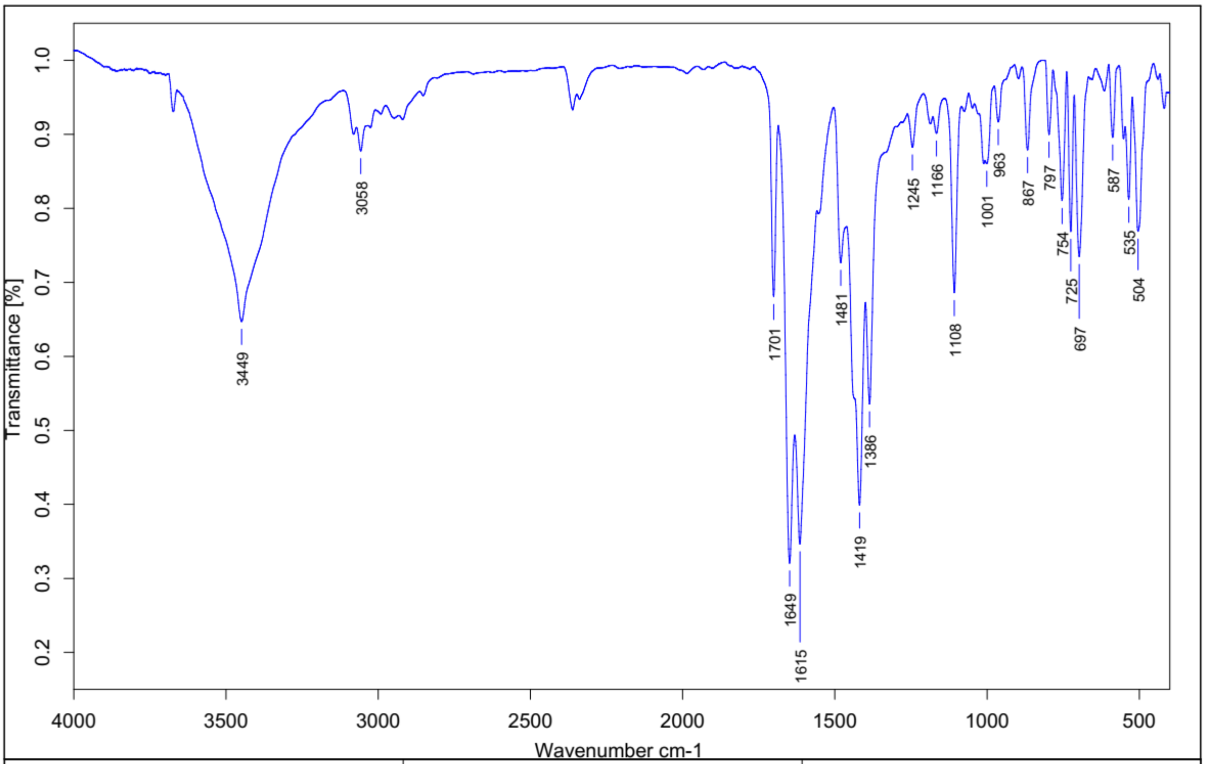

Probenname: vaf.20.08-1	Bereich Wellenzahlen: 4000.33 - 400.226	Datum: 26/05/2015
Probenform: KBr	Gerät: VECTOR22	Proben Scans: 8
Acquisition: Double Sided,Forward-Backward	Dateipfad: D:\DATEN_IR_BRUKER\FIORE	Datei: VAF.20.08-1.4

***(Z)*-3b**

^{1}H NMR

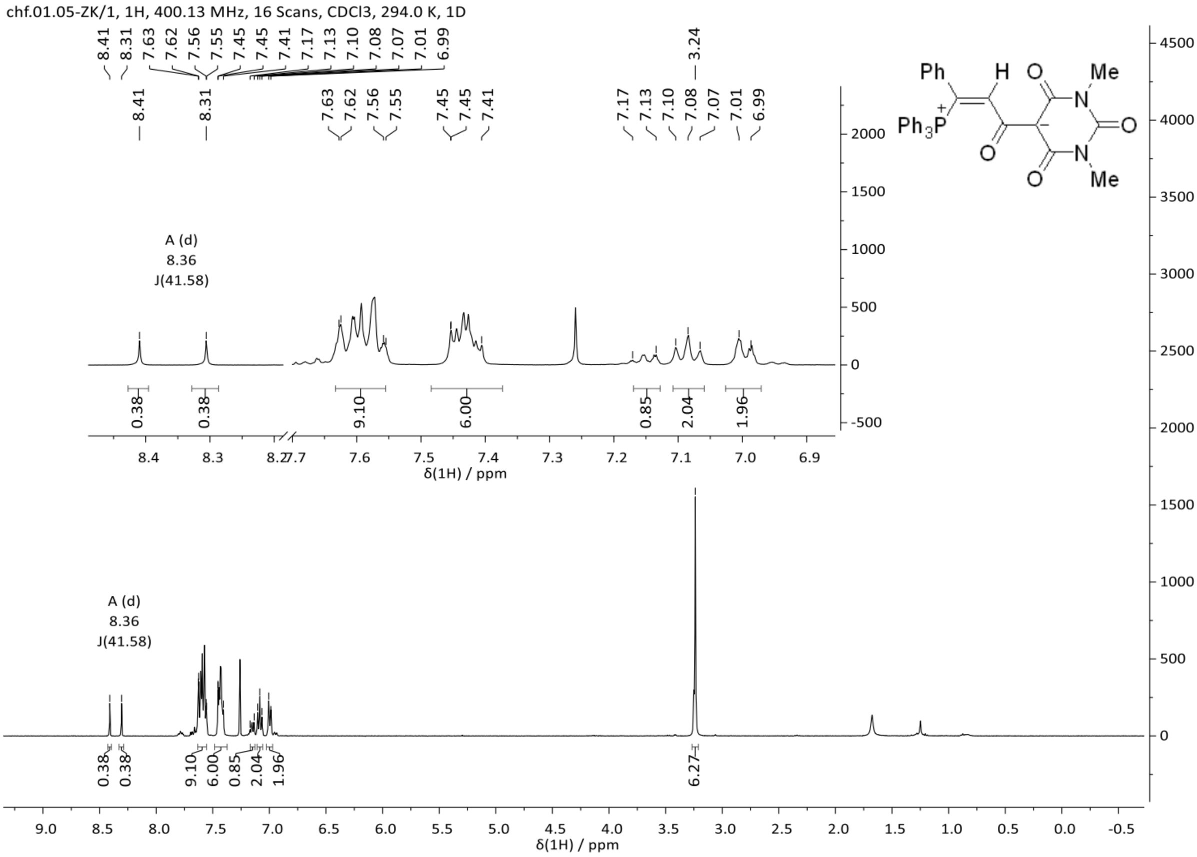

^{13}C NMR

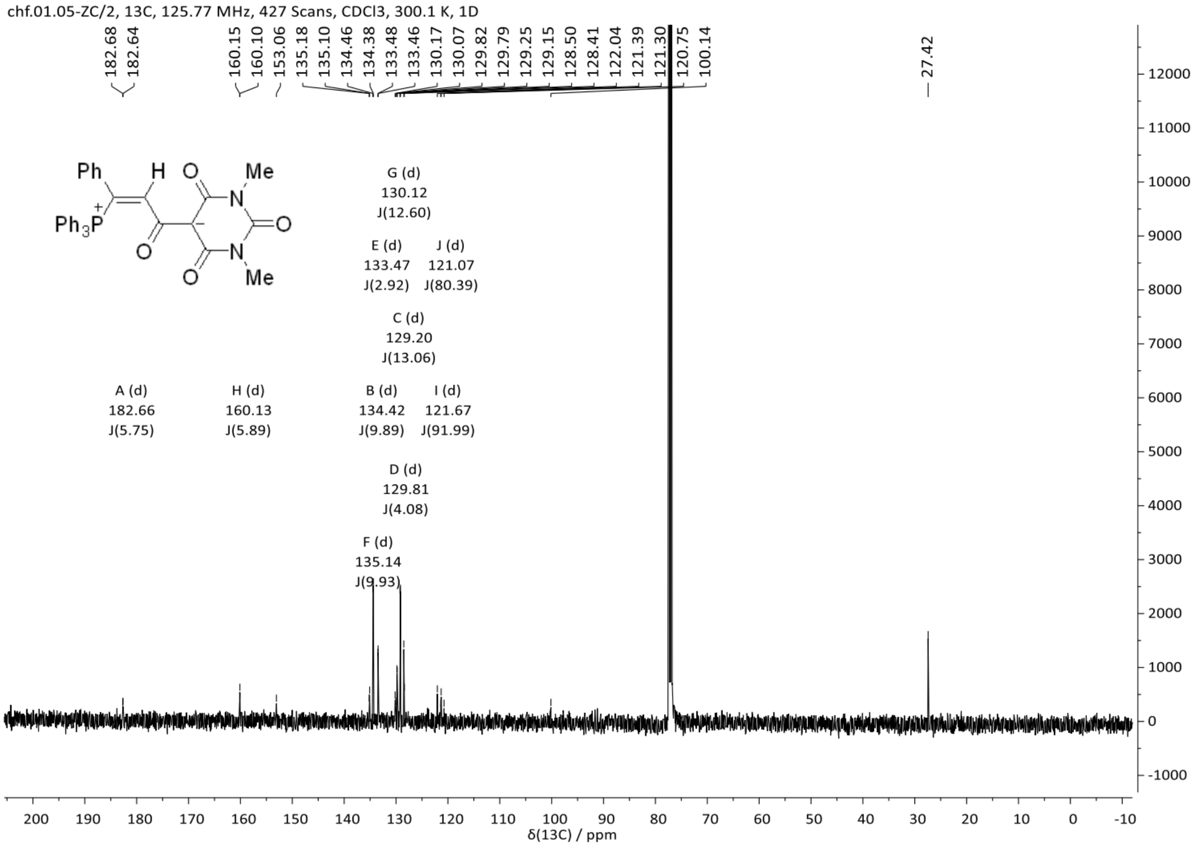

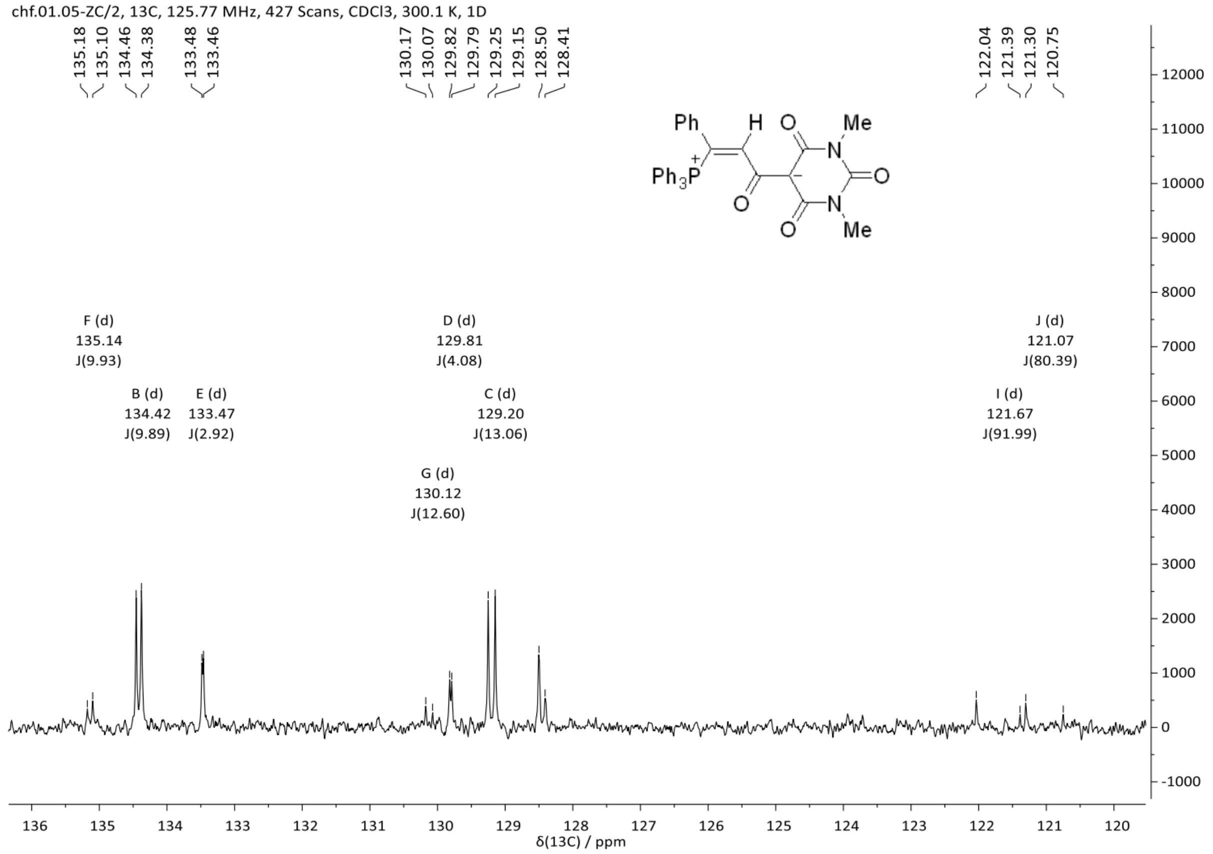
chf.01.05-ZC/2, 13C, 125.77 MHz, 427 Scans, CDCl3, 300.1 K, 1D
135.18
135.10
134.46
134.38
133.48
133.46
130.17
130.07
129.82
129.79
129.25
129.15
128.50
128.41
122.04
121.39
121.30
120.75
F (d) 135.14 J(9.93)
B (d) 134.42 J(9.89)
E (d) 133.47 J(2.92)
D (d) 129.81 J(4.08)
C (d) 129.20 J(13.06)
G (d) 130.12 J(12.60)
J (d) 121.07 J(80.39)
I (d) 121.67 J(91.99)
δ(13C) / ppm

^{31}P NMR

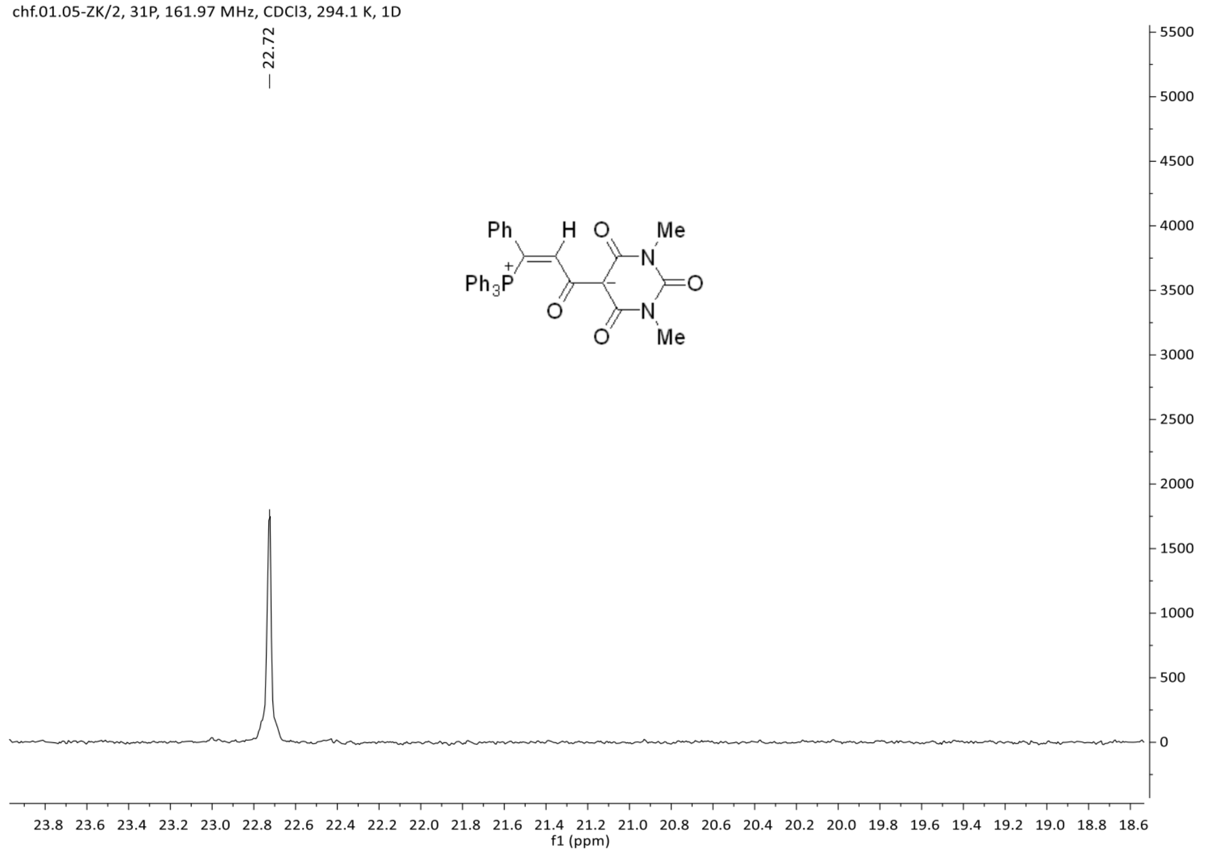
chf.01.05-ZK/2, 31P, 161.97 MHz, CDCl3, 294.1 K, 1D
22.72
f1 (ppm)

IR

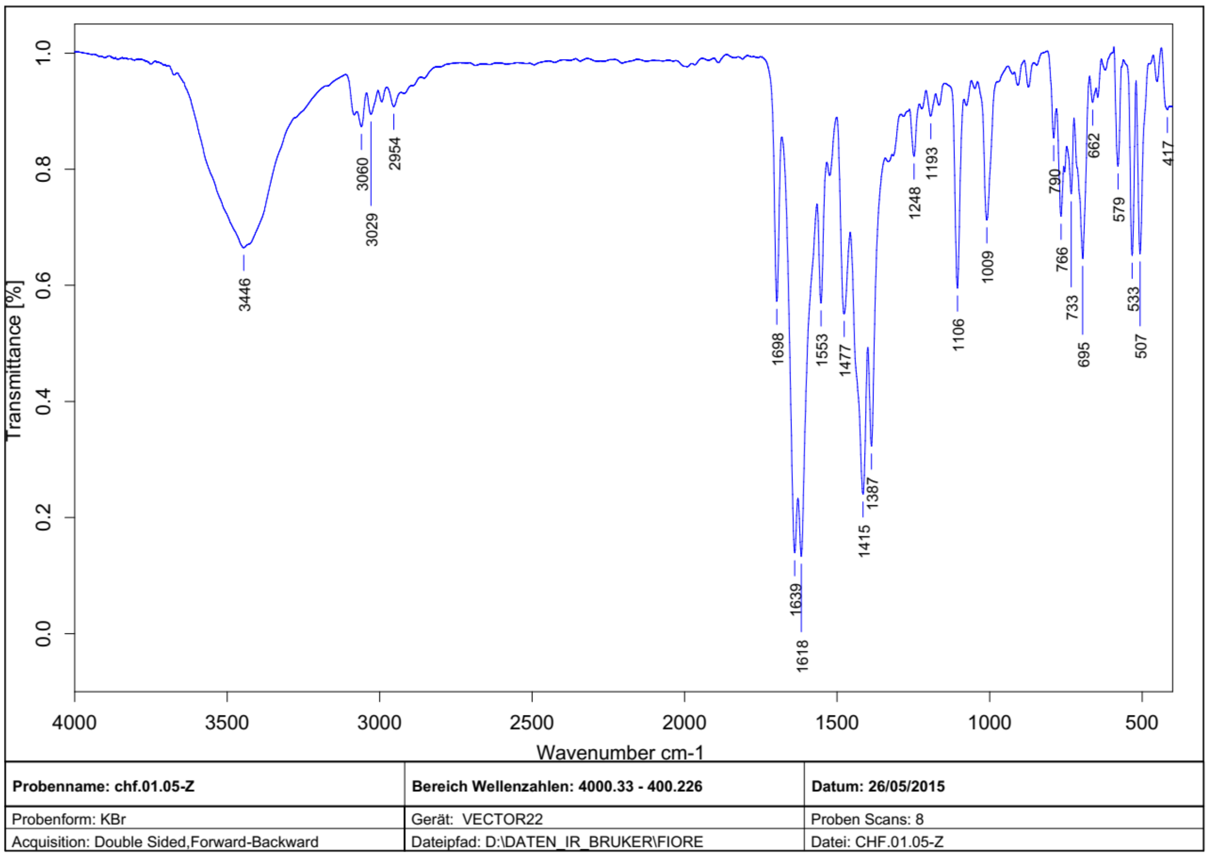

Probenname: chf.01.05-Z	Bereich Wellenzahlen: 4000.33 - 400.226	Datum: 26/05/2015
Probenform: KBr	Gerät: VECTOR22	Proben Scans: 8
Acquisition: Double Sided,Forward-Backward	Dateipfad: D:\DATEN_IR_BRUKER\FIORE	Datei: CHF.01.05-Z

4.3. Betaine (*E*)-3c

^{1}H NMR

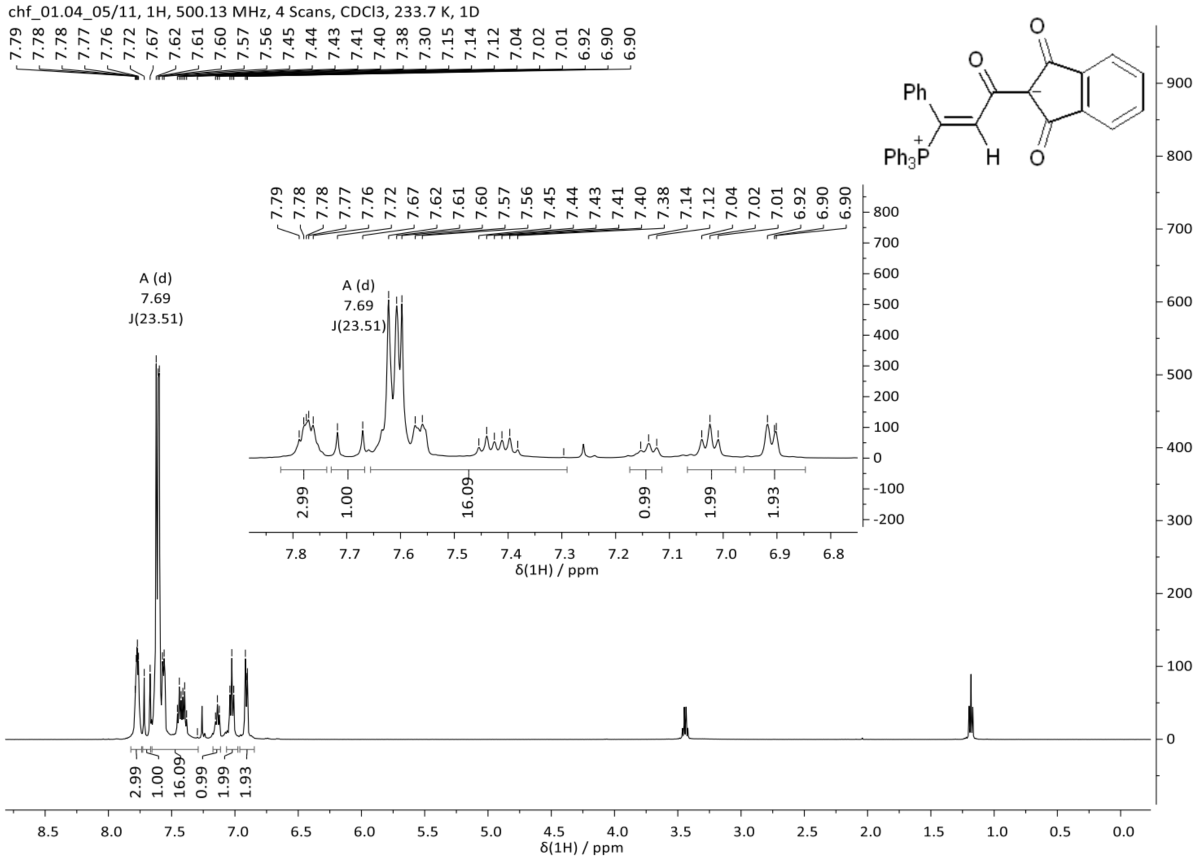

^{13}C NMR

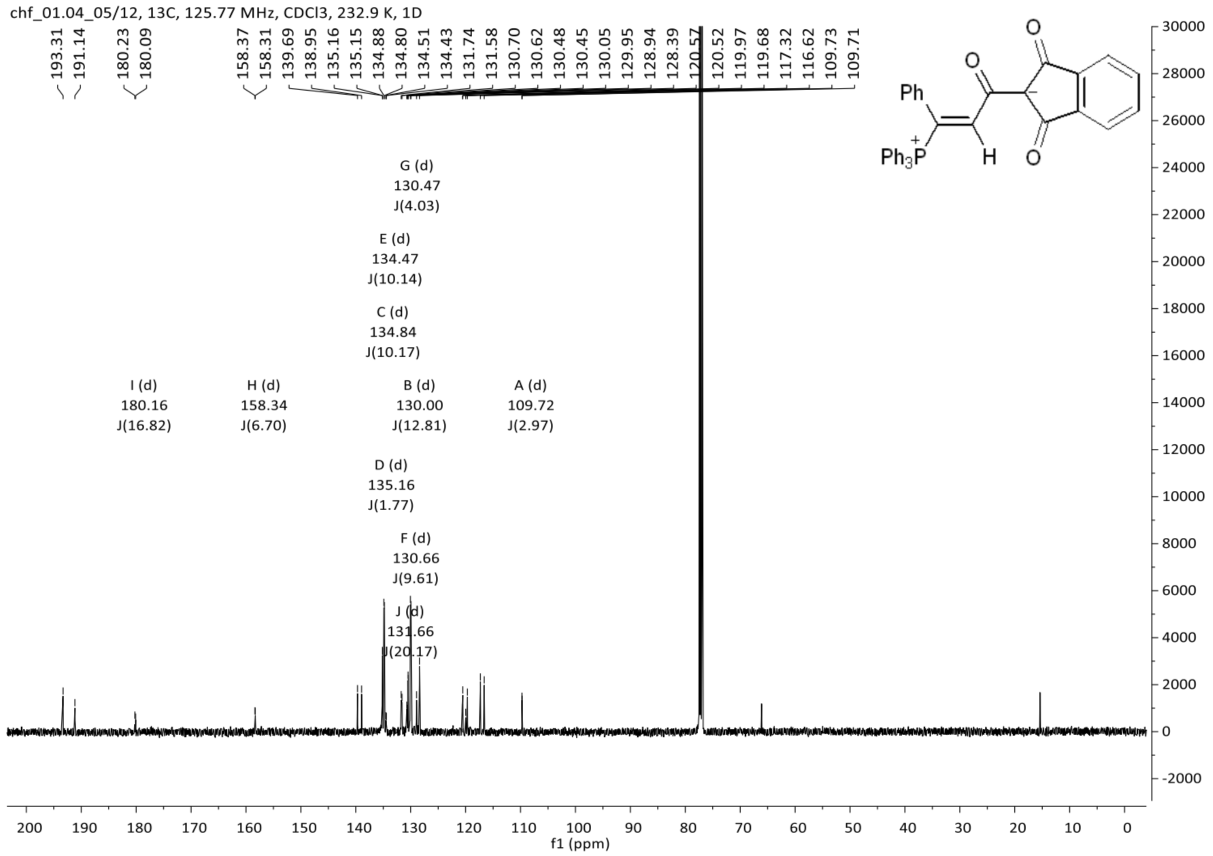

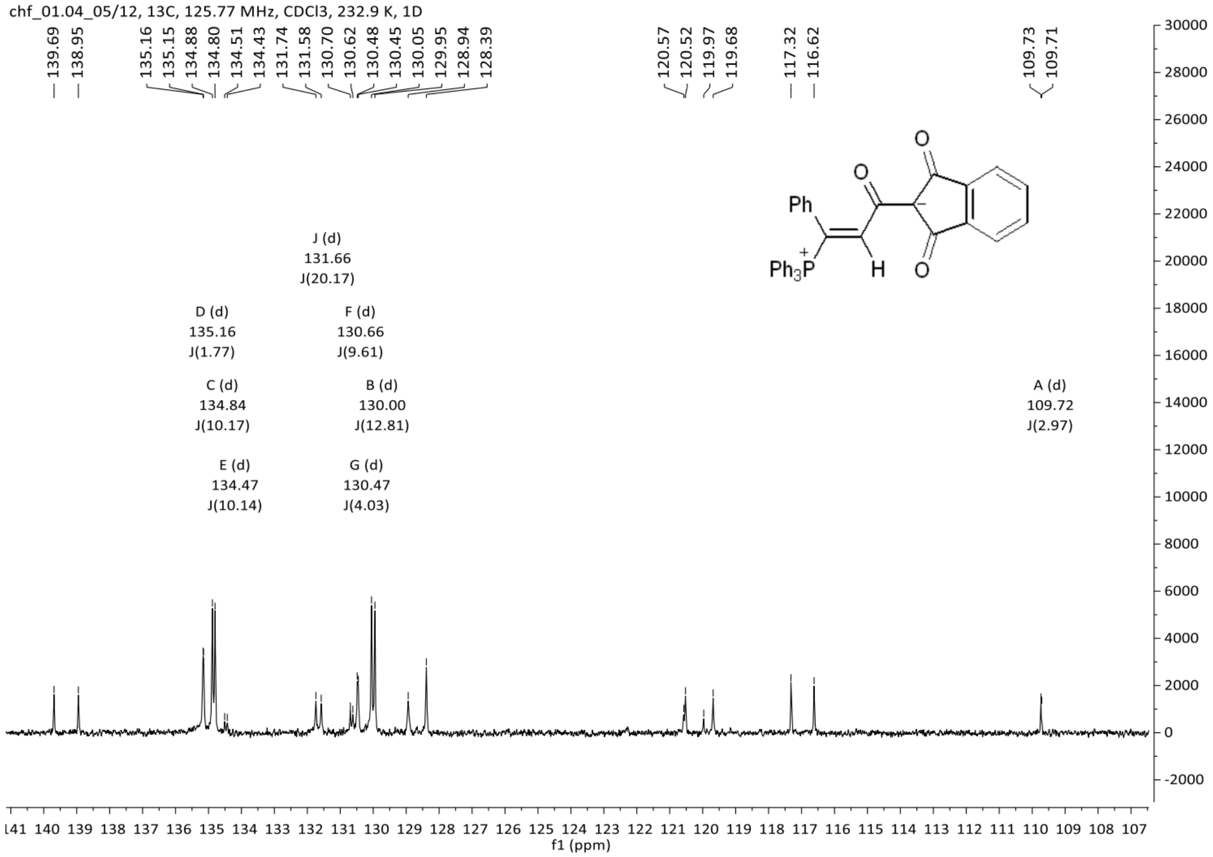

^{31}P NMR

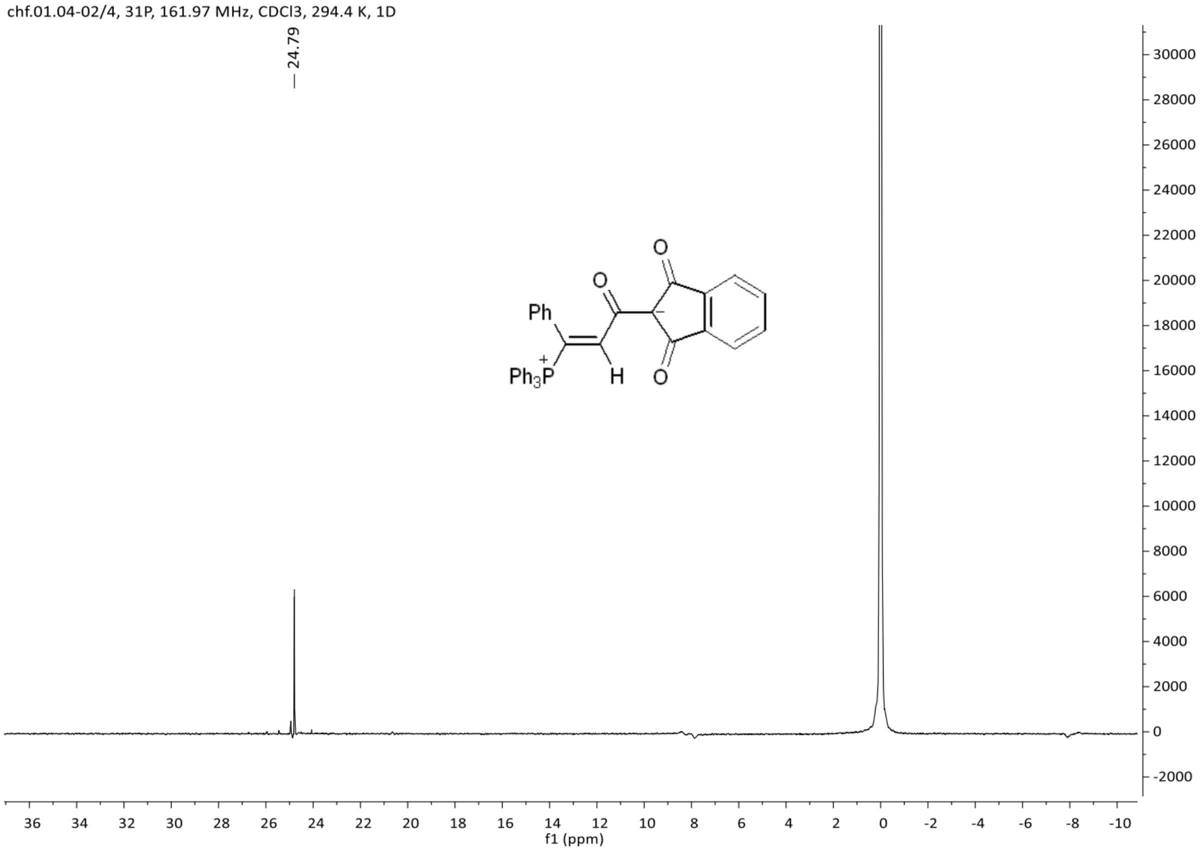

IR

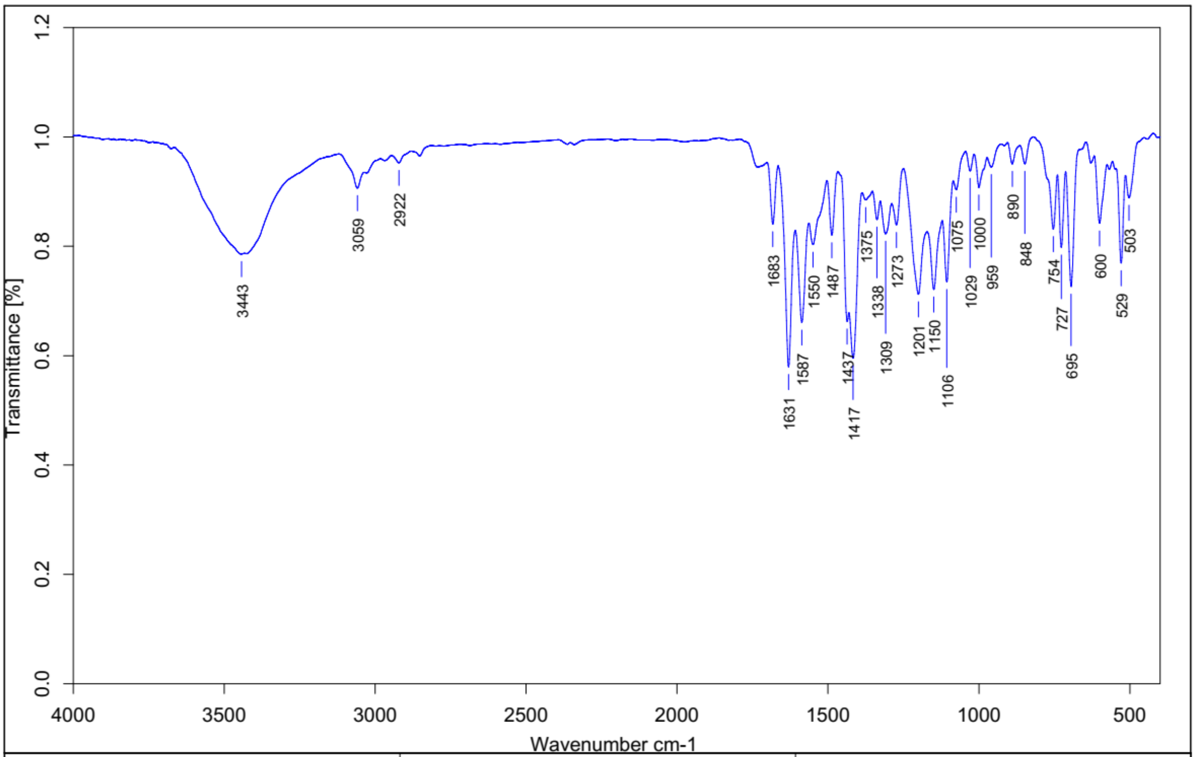

Probenname: chf.01.04-2	Bereich Wellenzahlen: 4000.33 - 400.226	Datum: 18/03/2015
Probenform: KBr	Gerät: VECTOR22	Proben Scans: 8
Acquisition: Double Sided,Forward-Backward	Dateipfad: D:\DATEN_IR_BRUKER\FIORE	Datei: CHF.01.04-2

4.4. Betaine (*E*)-3d

^{13}C NMR

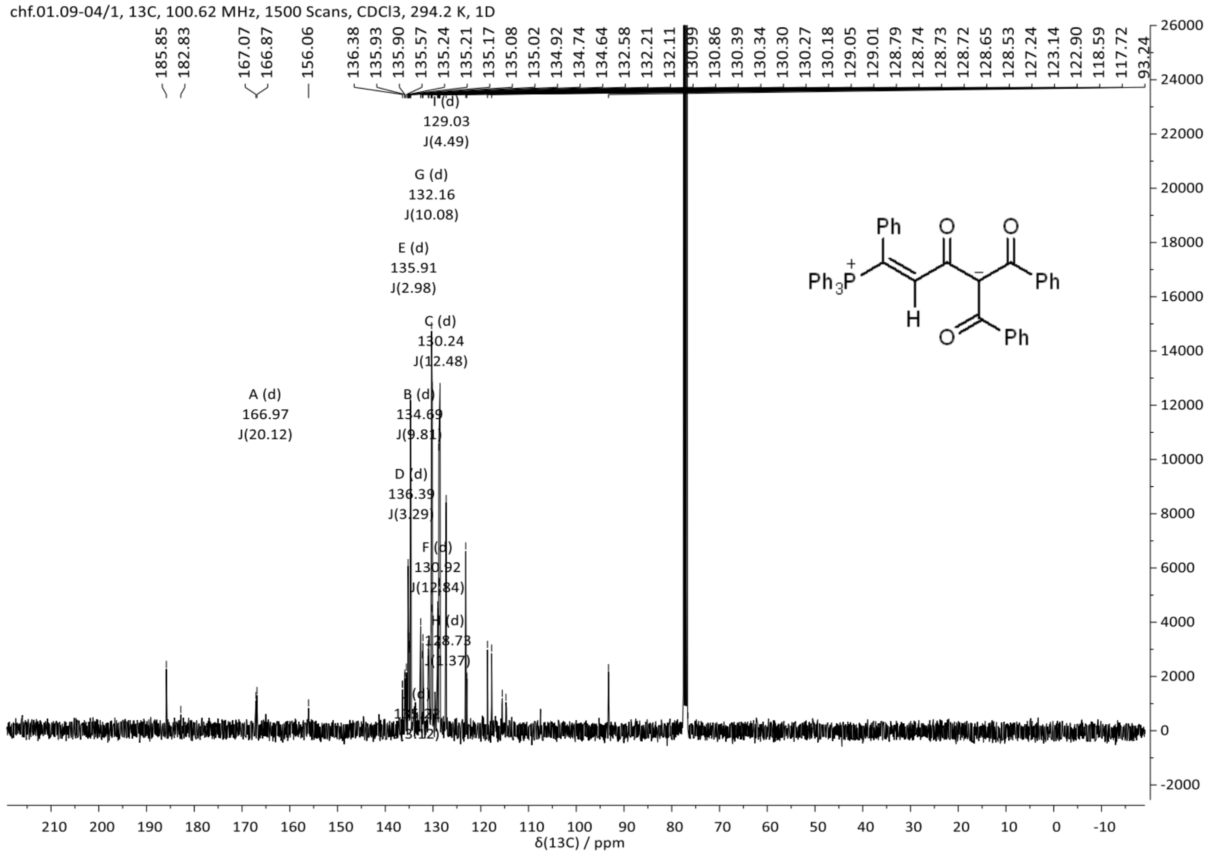

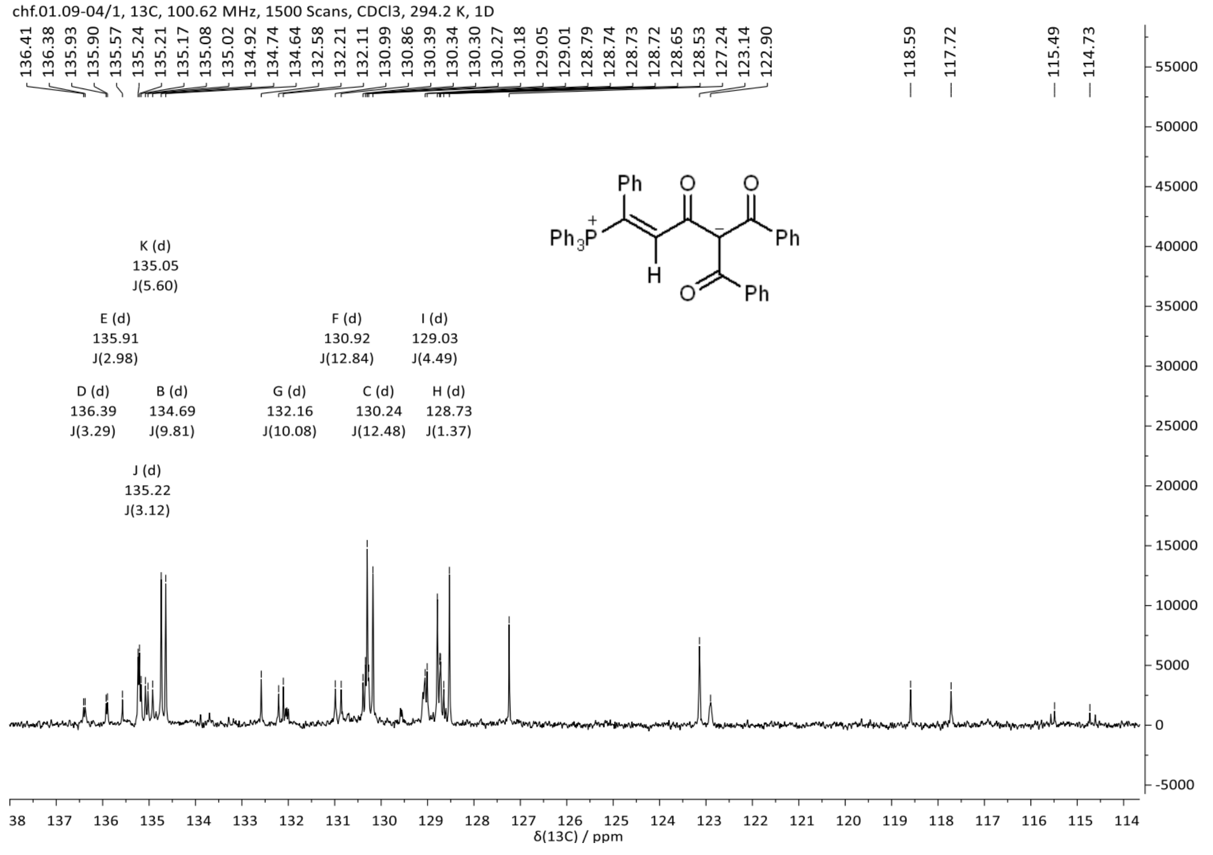

^{31}P NMR

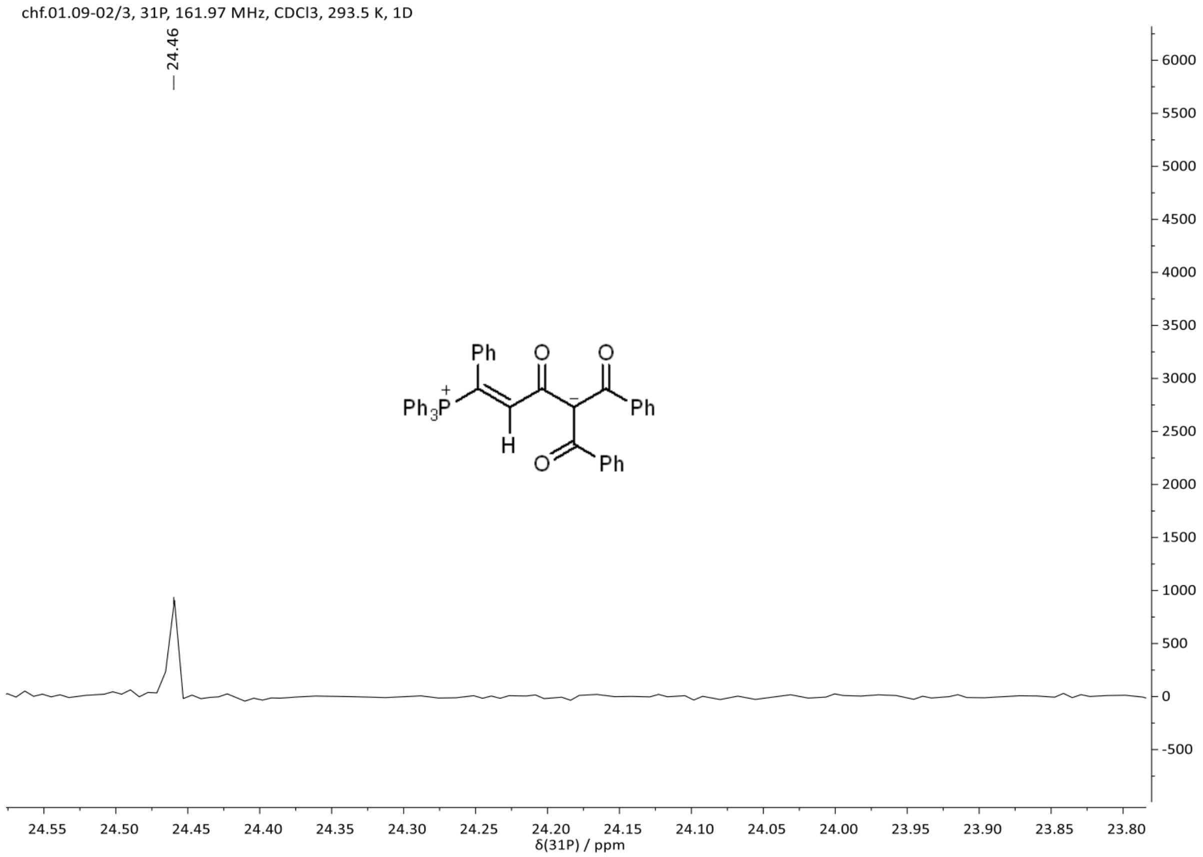

IR

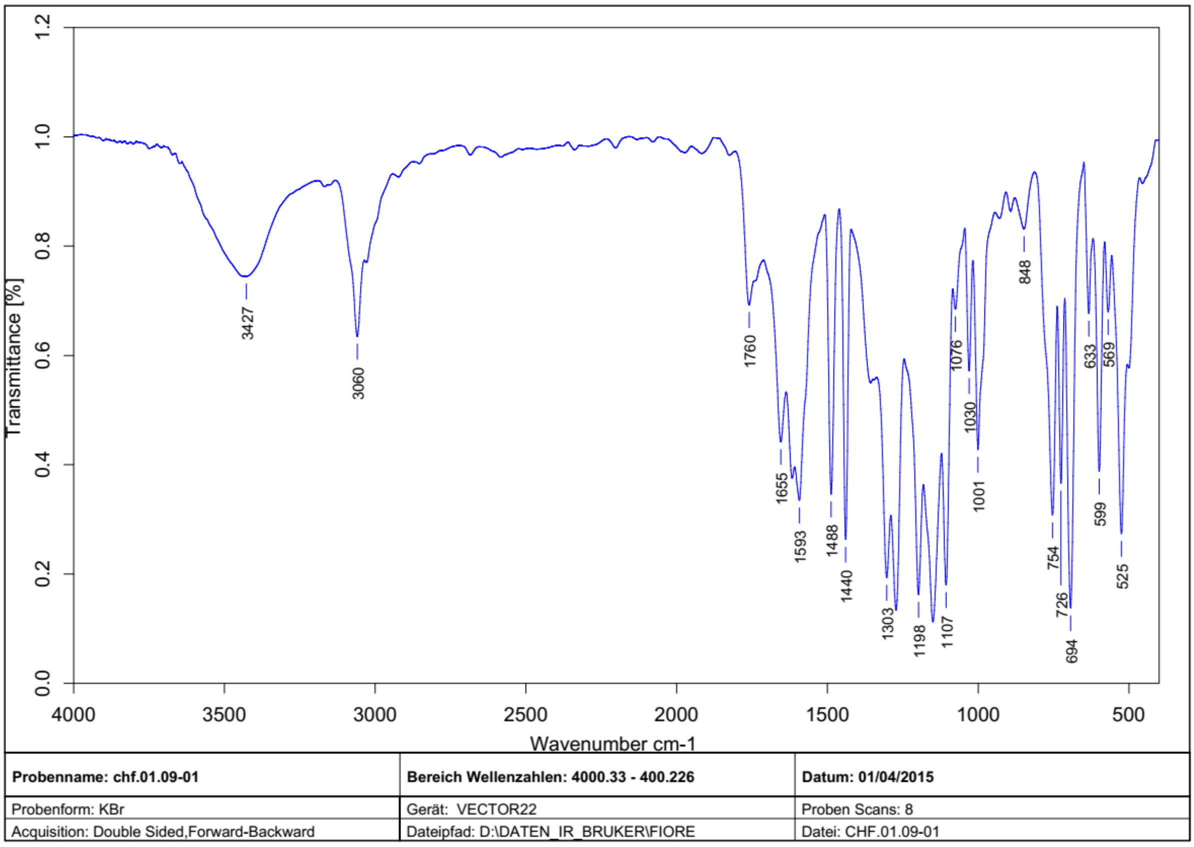

Probenname: chf.01.09-01	Bereich Wellenzahlen: 4000.33 - 400.226	Datum: 01/04/2015
Probenform: KBr	Gerät: VECTOR22	Proben Scans: 8
Acquisition: Double Sided,Forward-Backward	Dateipfad: D:\DATEN_IR_BRUKER\FIORE	Datei: CHF.01.09-01

4.5. Betaines (*E*)- and (*Z*)-3e

***(E)*-3e**

^{1}H NMR

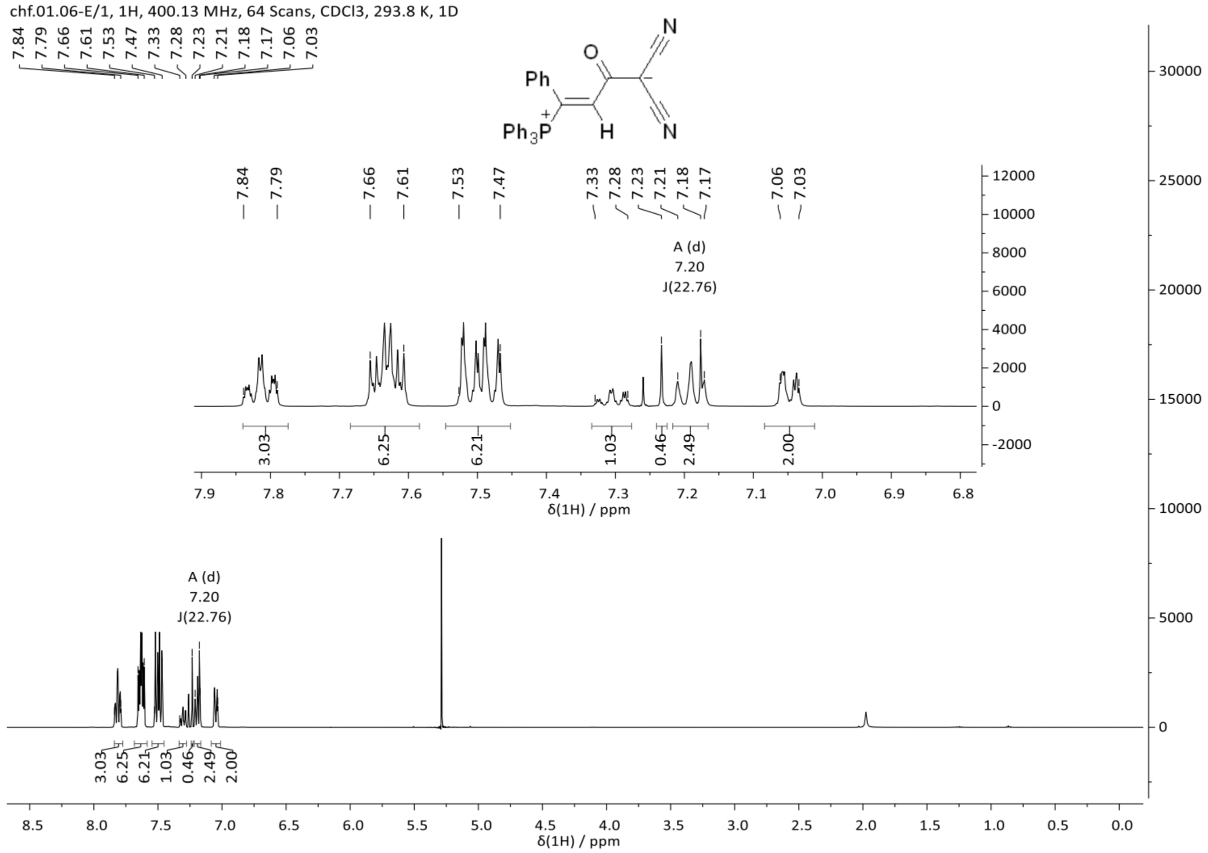

^{13}C NMR

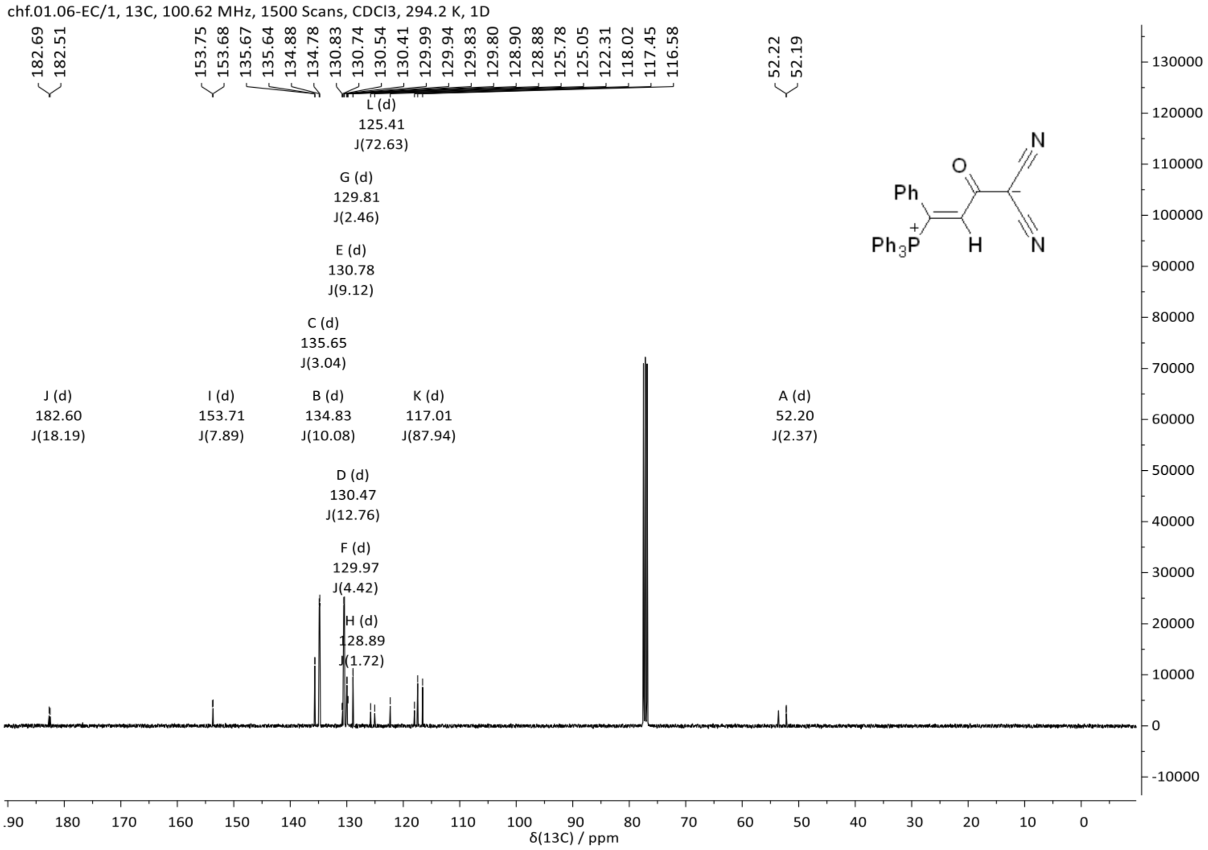

chf.01.06-EC/1, 13C, 100.62 MHz, 1500 Scans, CDCl3, 294.2 K, 1D
182.69
182.51
153.75
153.68
135.67
135.64
134.88
134.78
130.83
130.74
130.54
130.41
129.99
129.94
129.83
129.80
128.90
128.88
125.78
125.05
122.31
118.02
117.45
116.58
52.22
52.19
L (d) 125.41 J(72.63)
G (d) 129.81 J(2.46)
E (d) 130.78 J(9.12)
C (d) 135.65 J(3.04)
J (d) 182.60 J(18.19)
I (d) 153.71 J(7.89)
B (d) 134.83 J(10.08)
K (d) 117.01 J(87.94)
A (d) 52.20 J(2.37)
D (d) 130.47 J(12.76)
F (d) 129.97 J(4.42)
H (d) 128.89 J(1.72)
Ph
O
N
N
H
Ph3P
δ(13C) / ppm

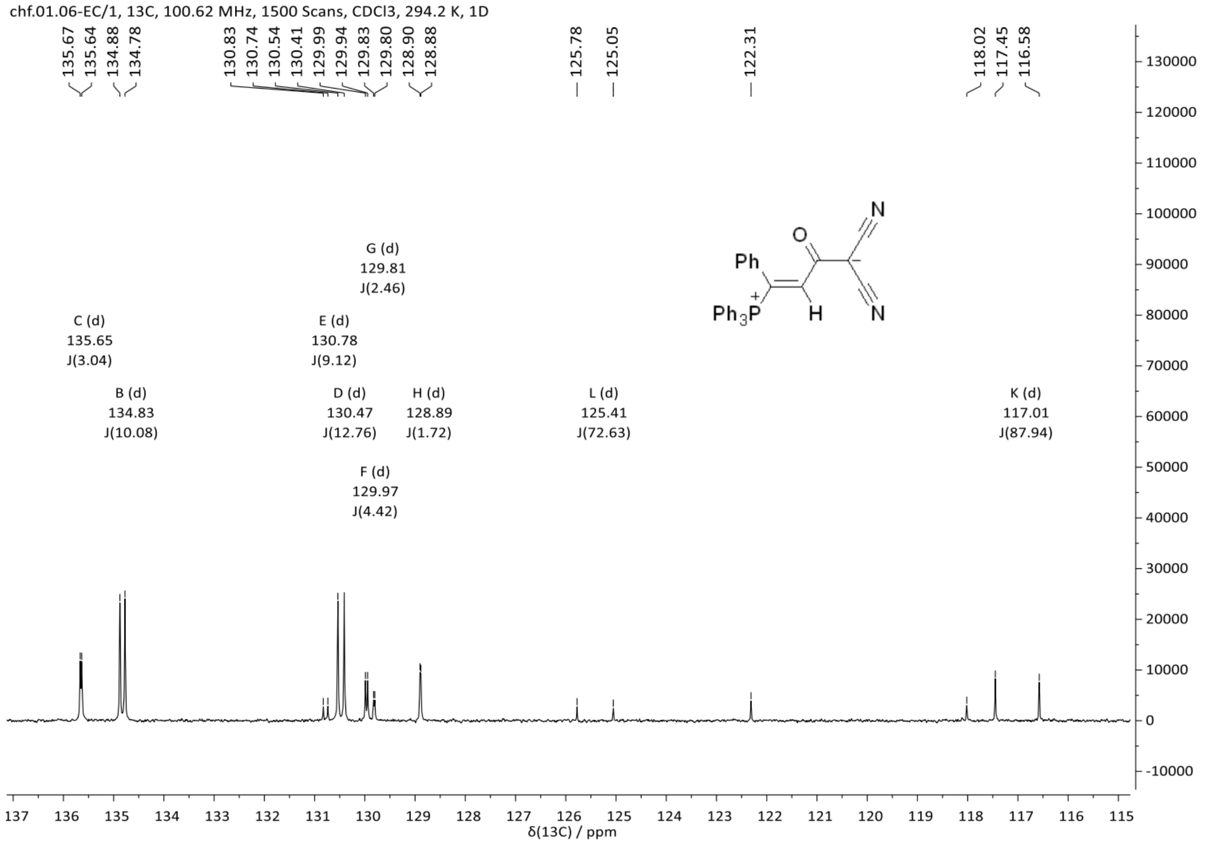

chf.01.06-EC/1, 13C, 100.62 MHz, 1500 Scans, CDCl3, 294.2 K, 1D
135.67
135.64
134.88
134.78
130.83
130.74
130.54
130.41
129.99
129.94
129.83
129.80
128.90
128.88
125.78
125.05
122.31
118.02
117.45
116.58
G (d) 129.81 J(2.46)
C (d) 135.65 J(3.04)
E (d) 130.78 J(9.12)
B (d) 134.83 J(10.08)
D (d) 130.47 J(12.76)
H (d) 128.89 J(1.72)
L (d) 125.41 J(72.63)
K (d) 117.01 J(87.94)
F (d) 129.97 J(4.42)
Ph
O
N
N
H
Ph3P
δ(13C) / ppm

^{31}P NMR

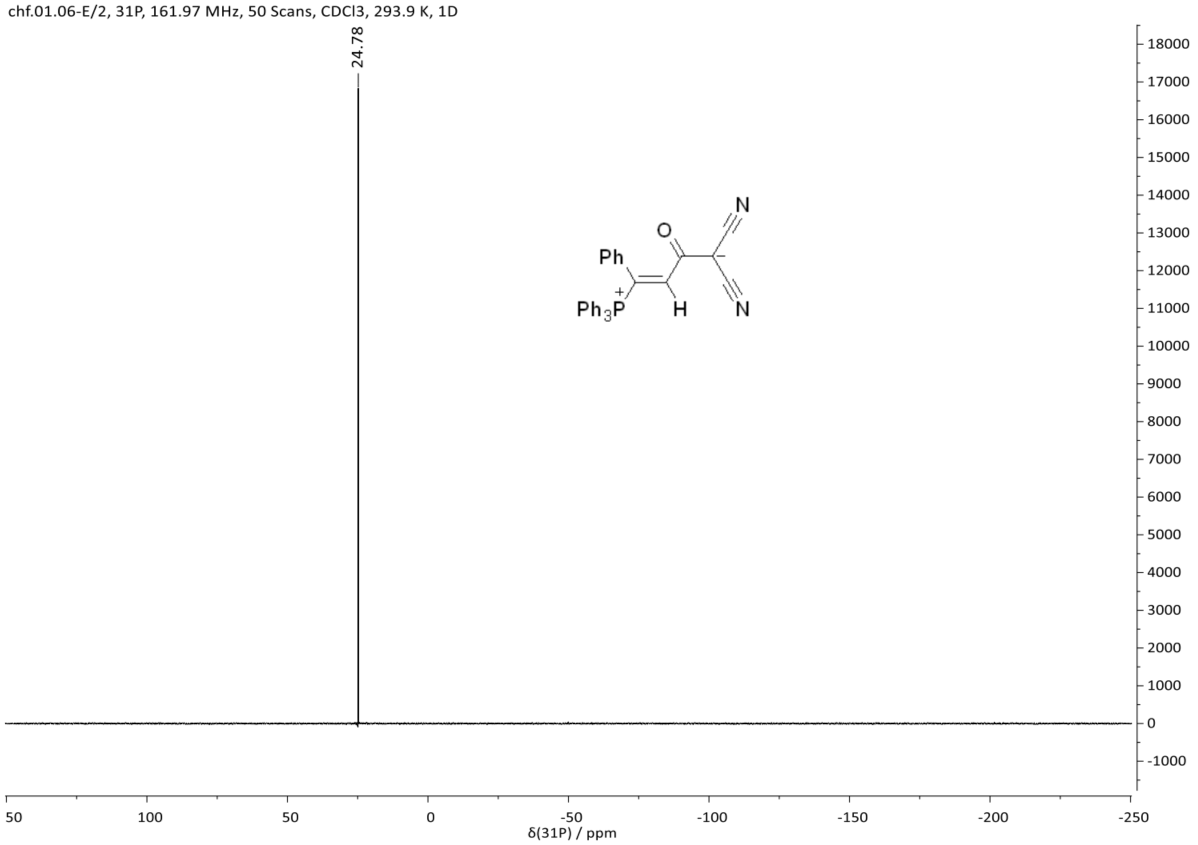

IR

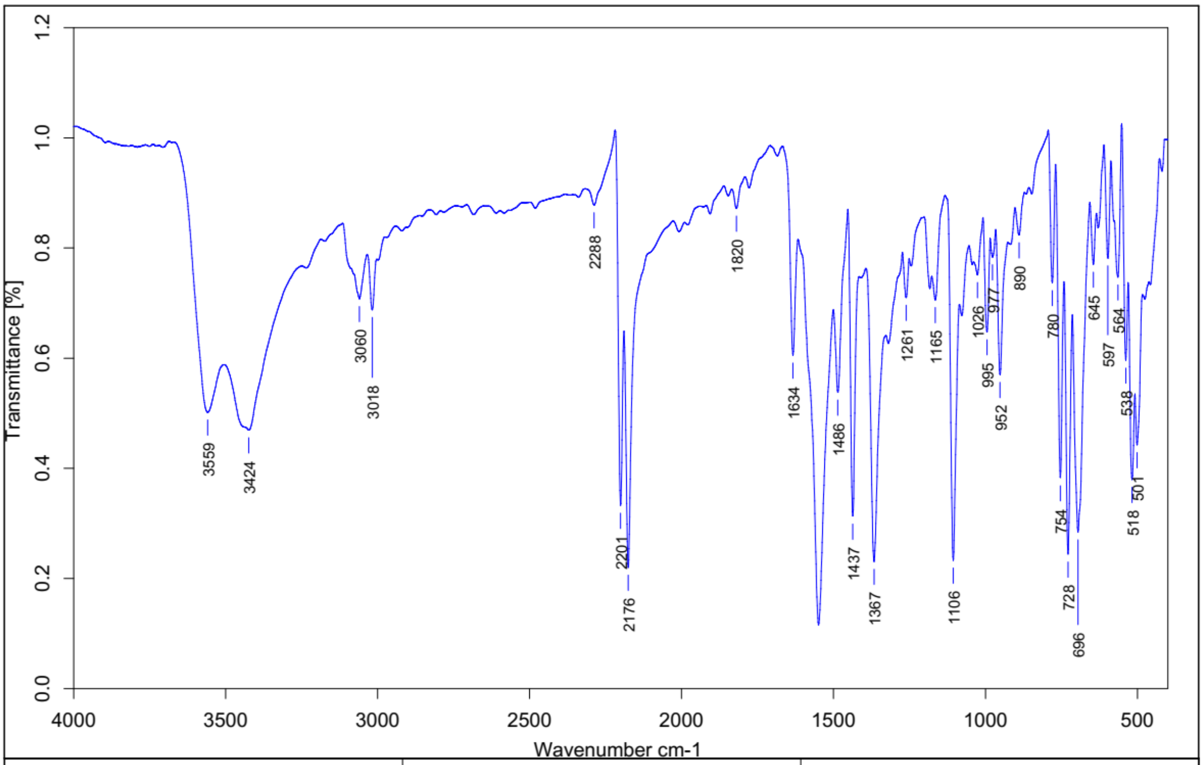

Probenname: chf.01.06-E	Bereich Wellenzahlen: 4000.33 - 400.226	Datum: 26/05/2015
Probenform: KBr	Gerät: VECTOR22	Proben Scans: 8
Acquisition: Double Sided,Forward-Backward	Dateipfad: D:\DATEN_IR_BRUKER\FIORE	Datei: CHF.01.06-E

(Z)-3e

^{1}H NMR

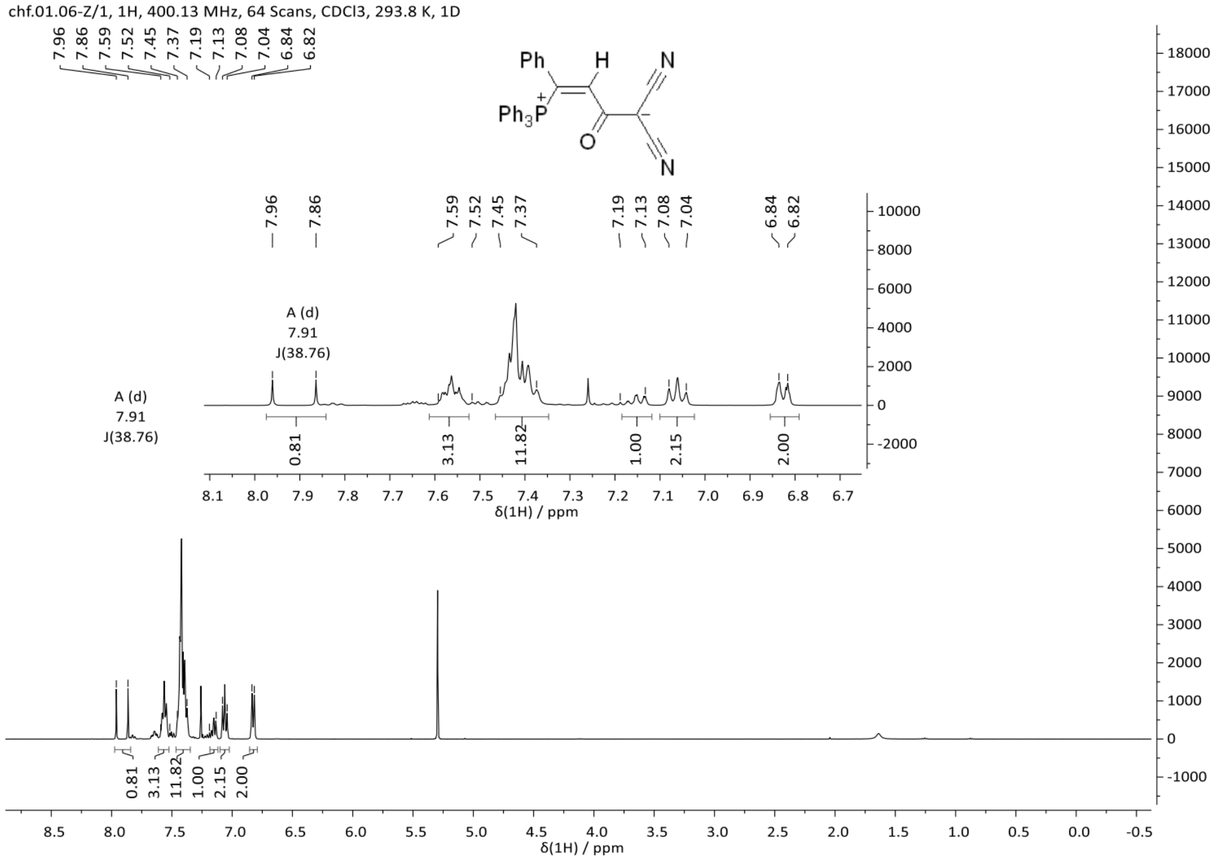

chf.01.06-Z/1, 1H, 400.13 MHz, 64 Scans, CDCl3, 293.8 K, 1D
7.96
7.86
7.59
7.52
7.45
7.37
7.19
7.13
7.08
7.04
6.84
6.82
Ph
H
N
Ph3P
O
A (d)
7.91
J(38.76)
0.81
3.13
11.82
1.00
2.15
2.00
δ(1H) / ppm

^{13}C NMR

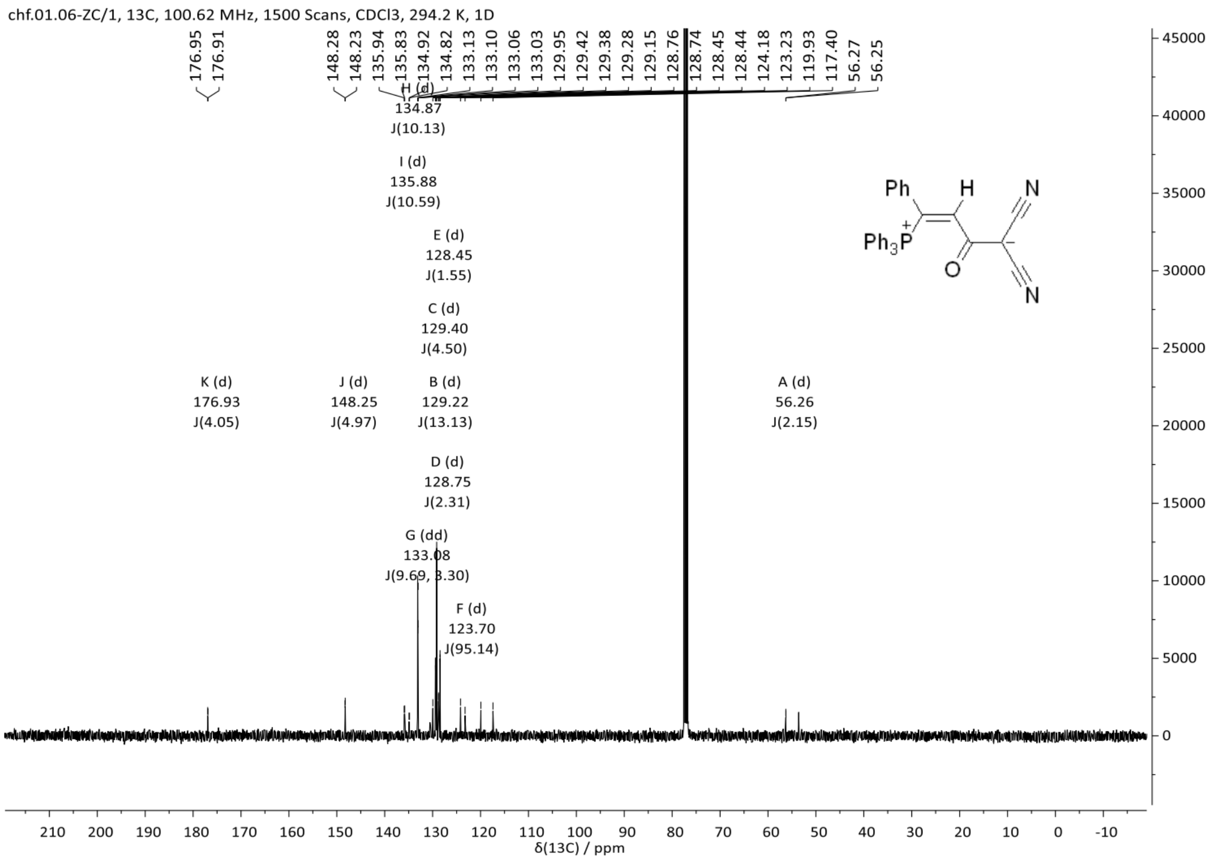
chf.01.06-ZC/1, 13C, 100.62 MHz, 1500 Scans, CDCl3, 294.2 K, 1D
176.95 176.91 148.28 148.23 135.94 135.83 134.92 134.82 133.13 133.10 133.06 133.03 129.95 129.42 129.38 129.28 129.15 128.76 128.74 128.45 128.44 124.18 123.23 119.93 117.40 56.27 56.25
H (d) 134.87 J(10.13)
I (d) 135.88 J(10.59)
E (d) 128.45 J(1.55)
C (d) 129.40 J(4.50)
K (d) 176.93 J(4.05)
J (d) 148.25 J(4.97)
B (d) 129.22 J(13.13)
A (d) 56.26 J(2.15)
D (d) 128.75 J(2.31)
G (dd) 133.08 J(9.69, 3.30)
F (d) 123.70 J(95.14)
Ph H N
Ph3P
O
N
δ(13C) / ppm

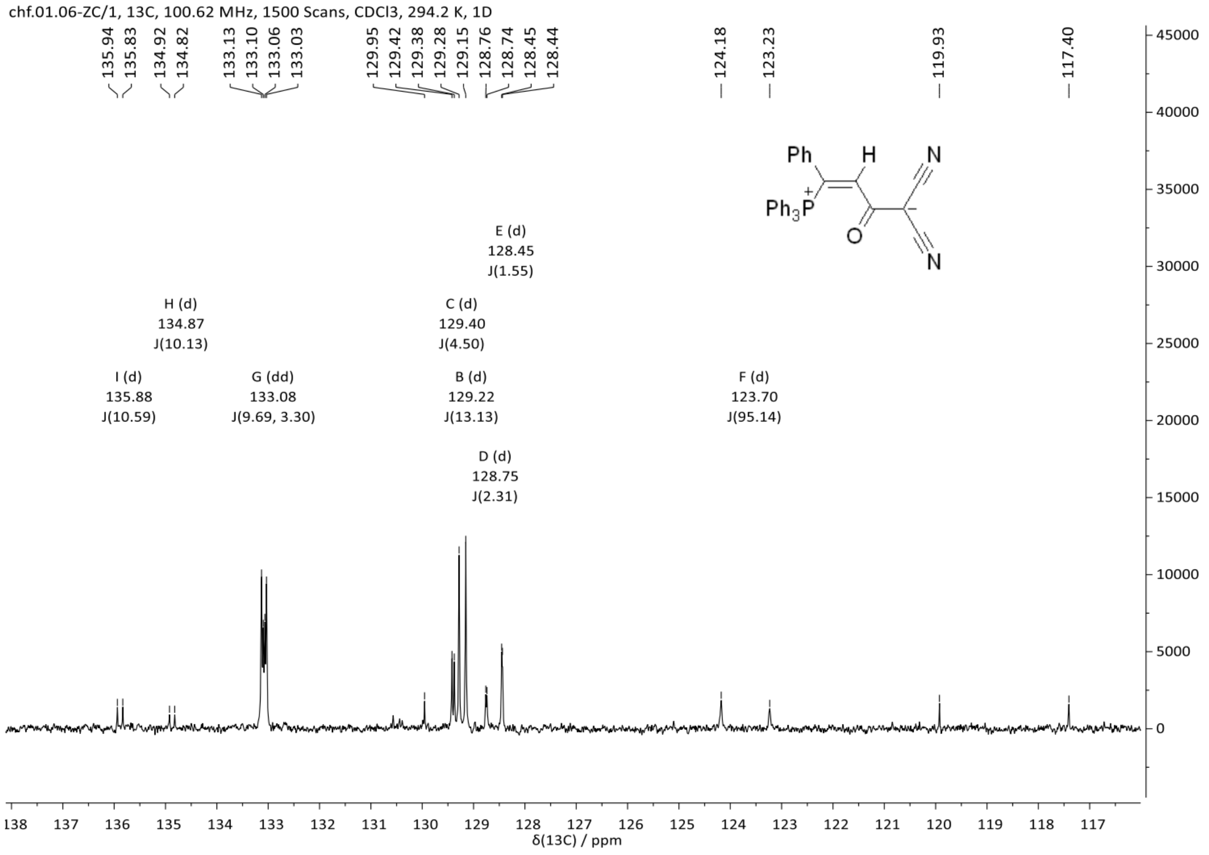
chf.01.06-ZC/1, 13C, 100.62 MHz, 1500 Scans, CDCl3, 294.2 K, 1D
135.94 135.83 134.92 134.82 133.13 133.10 133.06 133.03 129.95 129.42 129.38 129.28 129.15 128.76 128.74 128.45 128.44 124.18 123.23 119.93 117.40
E (d) 128.45 J(1.55)
H (d) 134.87 J(10.13)
C (d) 129.40 J(4.50)
I (d) 135.88 J(10.59)
G (dd) 133.08 J(9.69, 3.30)
B (d) 129.22 J(13.13)
F (d) 123.70 J(95.14)
D (d) 128.75 J(2.31)
Ph H N
Ph3P
O
N
δ(13C) / ppm

^{31}P NMR

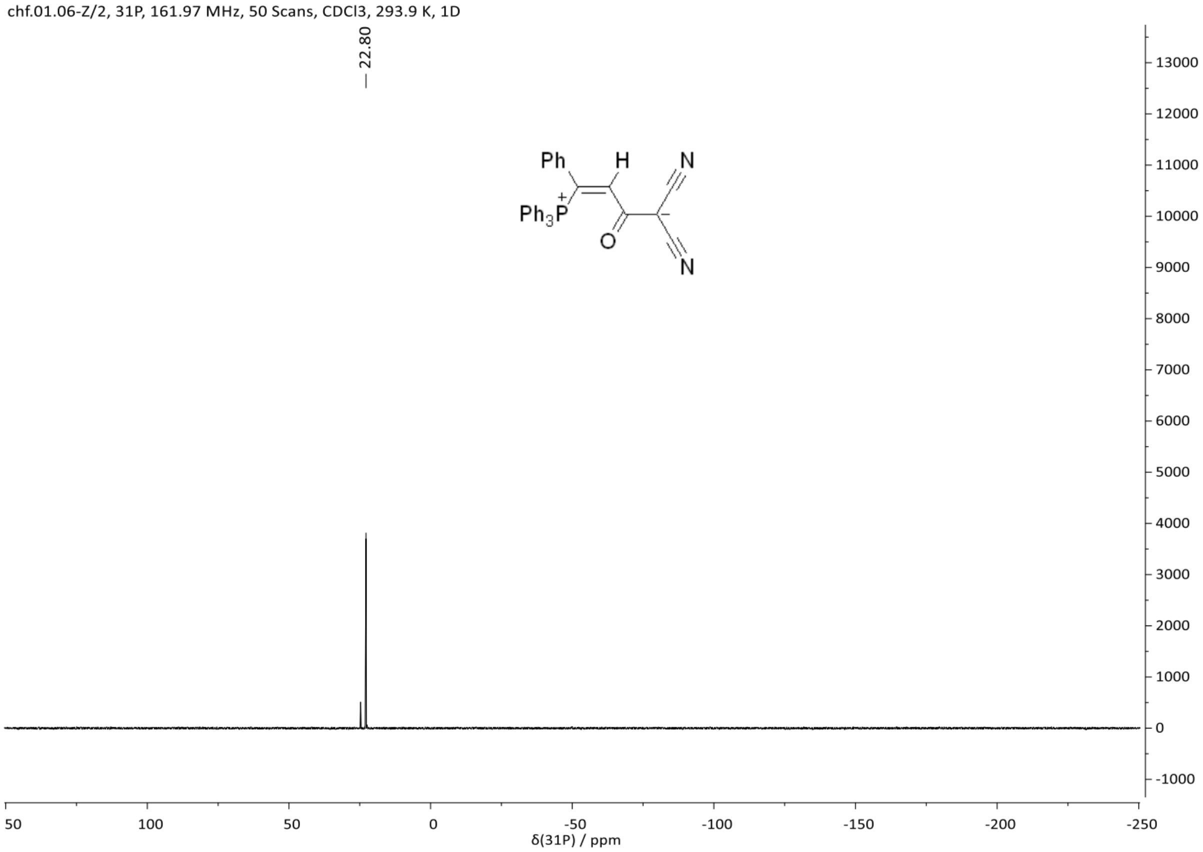

chf.01.06-Z/2, 31P, 161.97 MHz, 50 Scans, CDCl3, 293.9 K, 1D
22.80
δ(31P) / ppm

IR

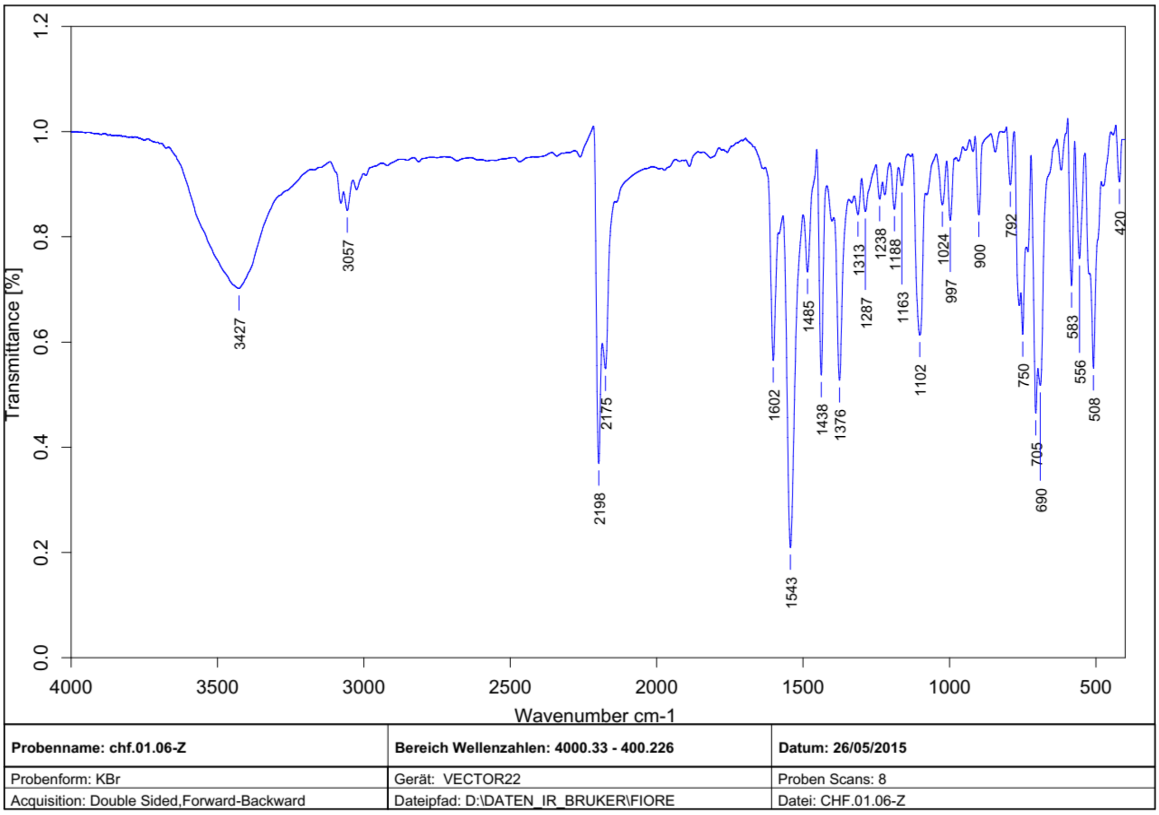

Transmittance [%]
Wavenumber cm-1
3427
3057
2198
2175
1602
1543
1485
1438
1376
1313
1287
1238
1188
1163
1102
1024
997
900
792
750
705
690
583
556
508
420
Probenname: chf.01.06-Z
Bereich Wellenzahlen: 4000.33 - 400.226
Datum: 26/05/2015
Probenform: KBr
Gerät: VECTOR22
Proben Scans: 8
Acquisition: Double Sided,Forward-Backward
Dateipfad: D:\DATEN_IR_BRUKER\FIORE
Datei: CHF.01.06-Z

4.6. Betaines (*E*)- and (*Z*)-3f

(E)-3f

^{1}H NMR

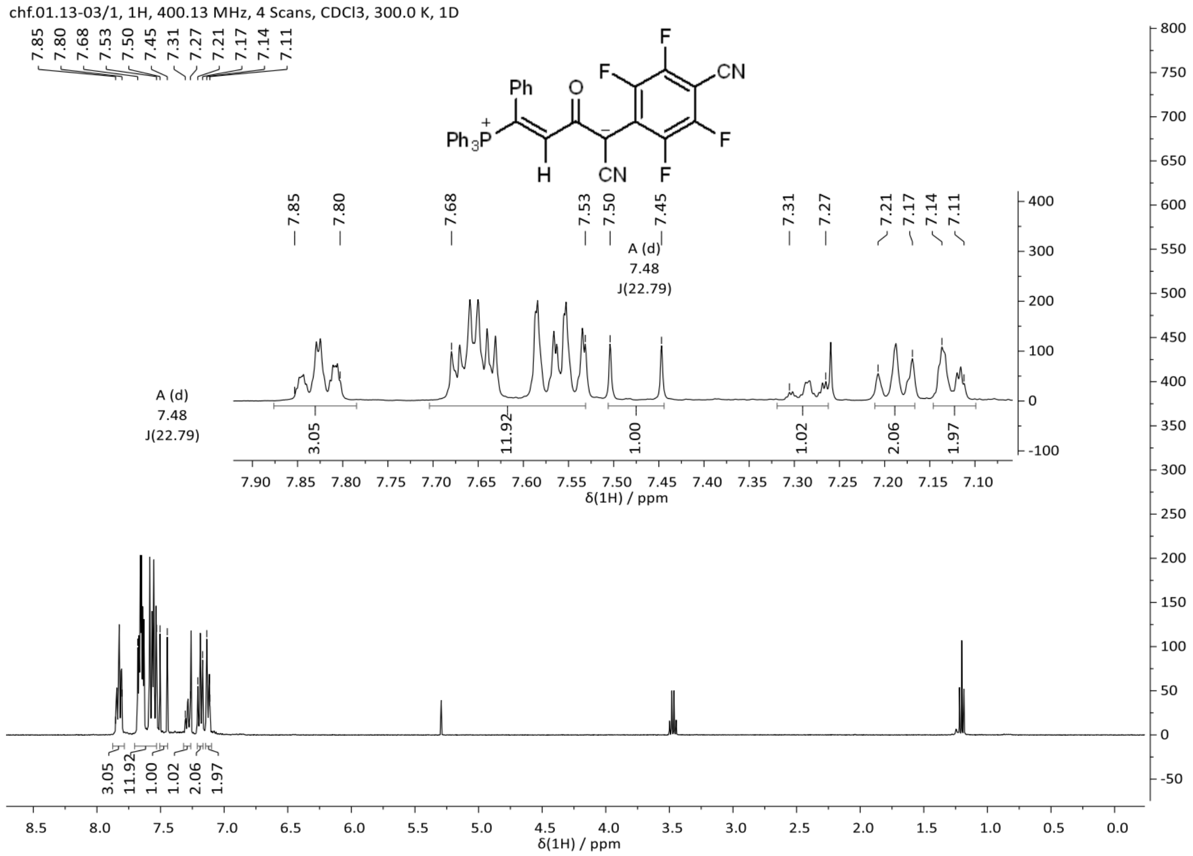

^{13}C NMR

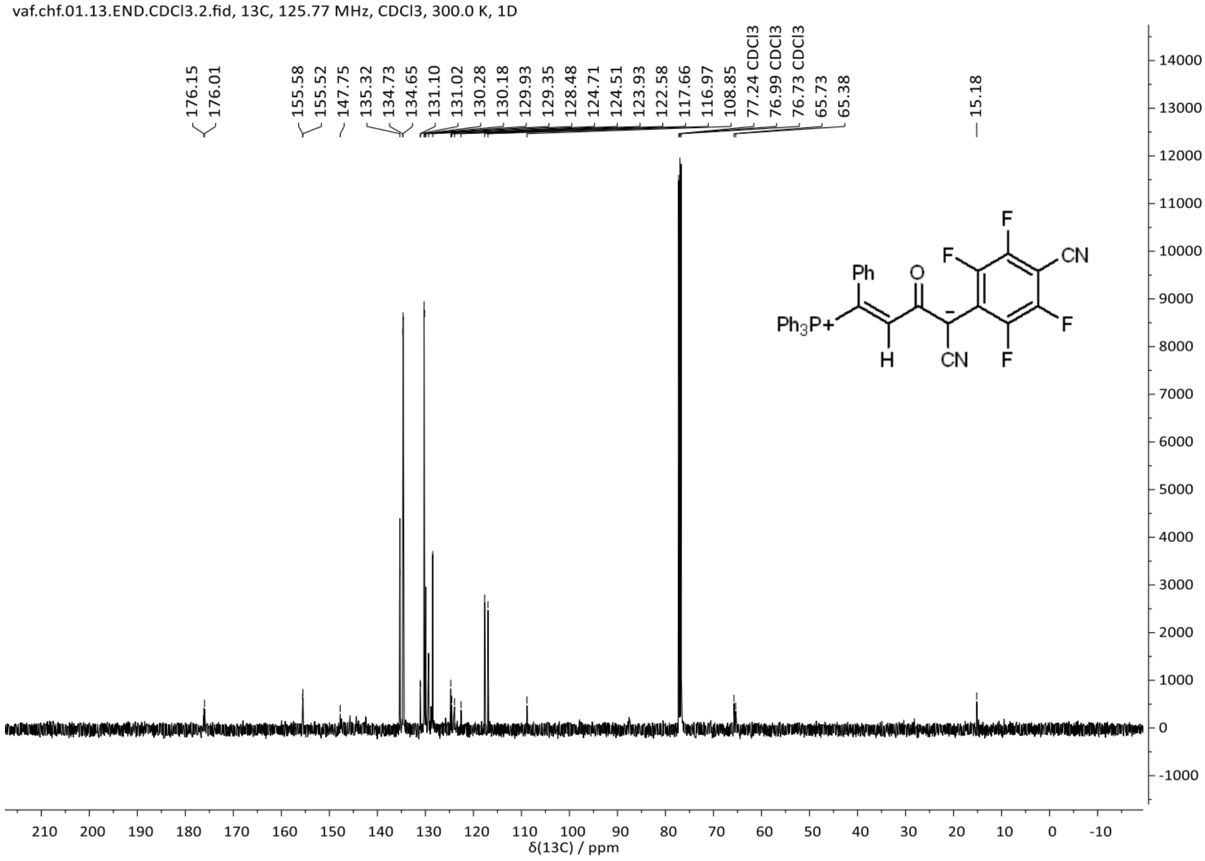
vaf.chf.01.13.END.CDCl3.2.fid, 13C, 125.77 MHz, CDCl3, 300.0 K, 1D
176.15
176.01
155.58
155.52
147.75
135.32
134.73
134.65
131.10
131.02
130.28
130.18
129.93
129.35
128.48
124.71
124.51
123.93
122.58
117.66
116.97
108.85
77.24 CDCl3
76.99 CDCl3
76.73 CDCl3
65.73
65.38
15.18
Ph
O
F
CN
Ph3P+
H
CN
δ(13C) / ppm

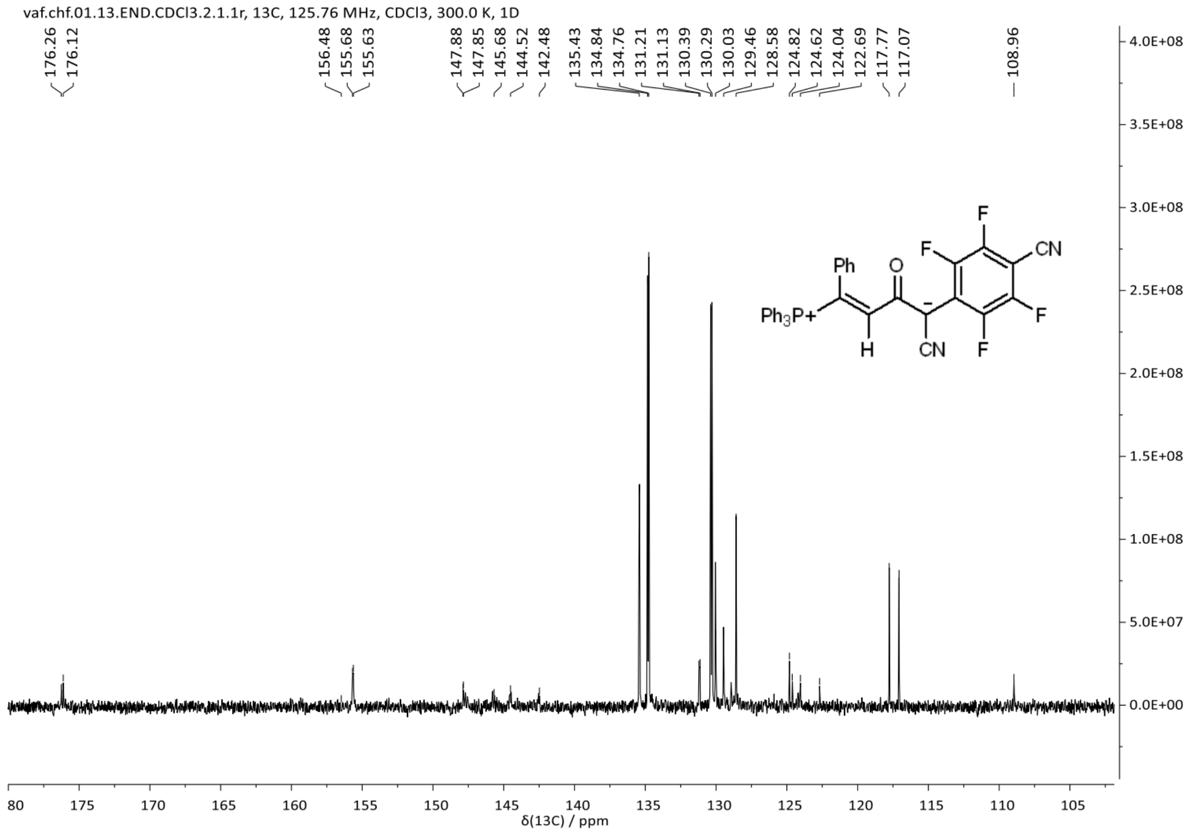
vaf.chf.01.13.END.CDCl3.2.1.1r, 13C, 125.76 MHz, CDCl3, 300.0 K, 1D
176.26
176.12
156.48
155.68
155.63
147.88
147.85
145.68
144.52
142.48
135.43
134.84
134.76
131.21
131.13
130.39
130.29
130.03
129.46
128.58
124.82
124.62
124.04
122.69
117.77
117.07
108.96
δ(13C) / ppm

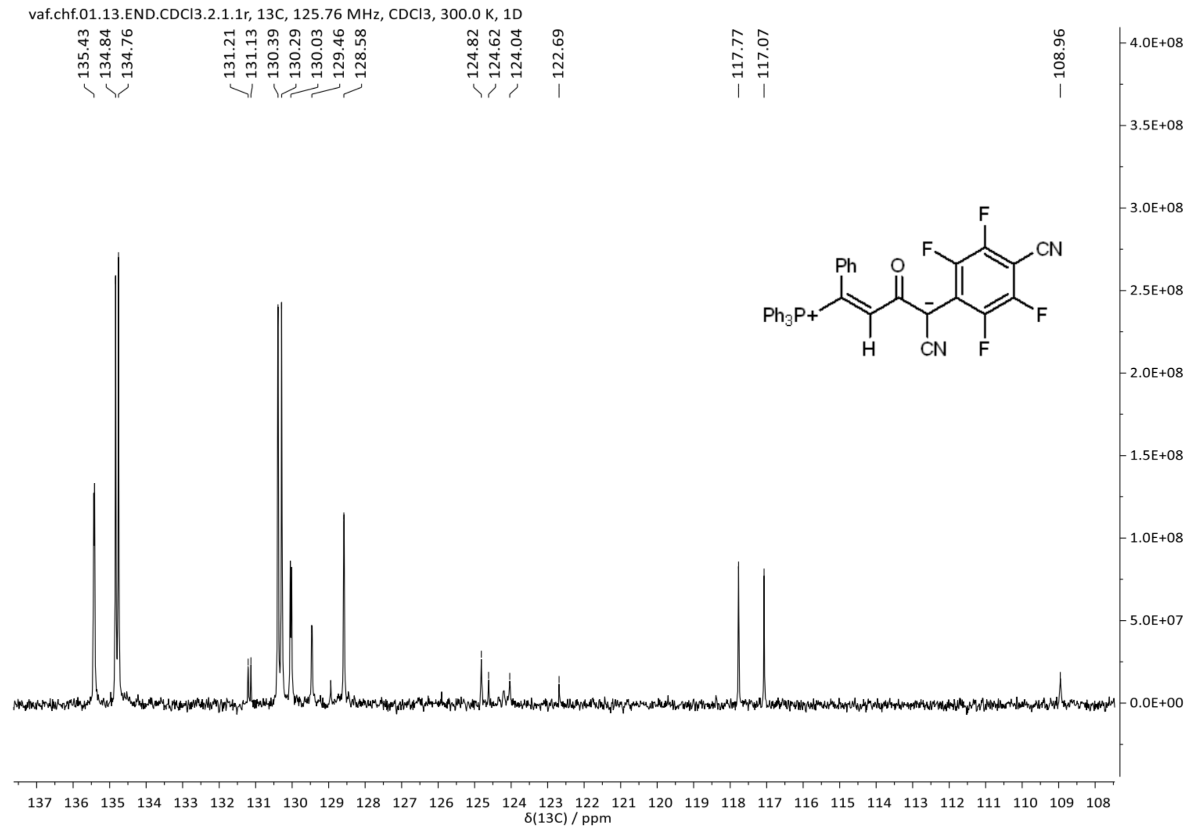
vaf.chf.01.13.END.CDCl3.2.1.1r, 13C, 125.76 MHz, CDCl3, 300.0 K, 1D
135.43
134.84
134.76
131.21
131.13
130.39
130.29
130.03
129.46
128.58
124.82
124.62
124.04
122.69
117.77
117.07
108.96
δ(13C) / ppm

^{19}F NMR

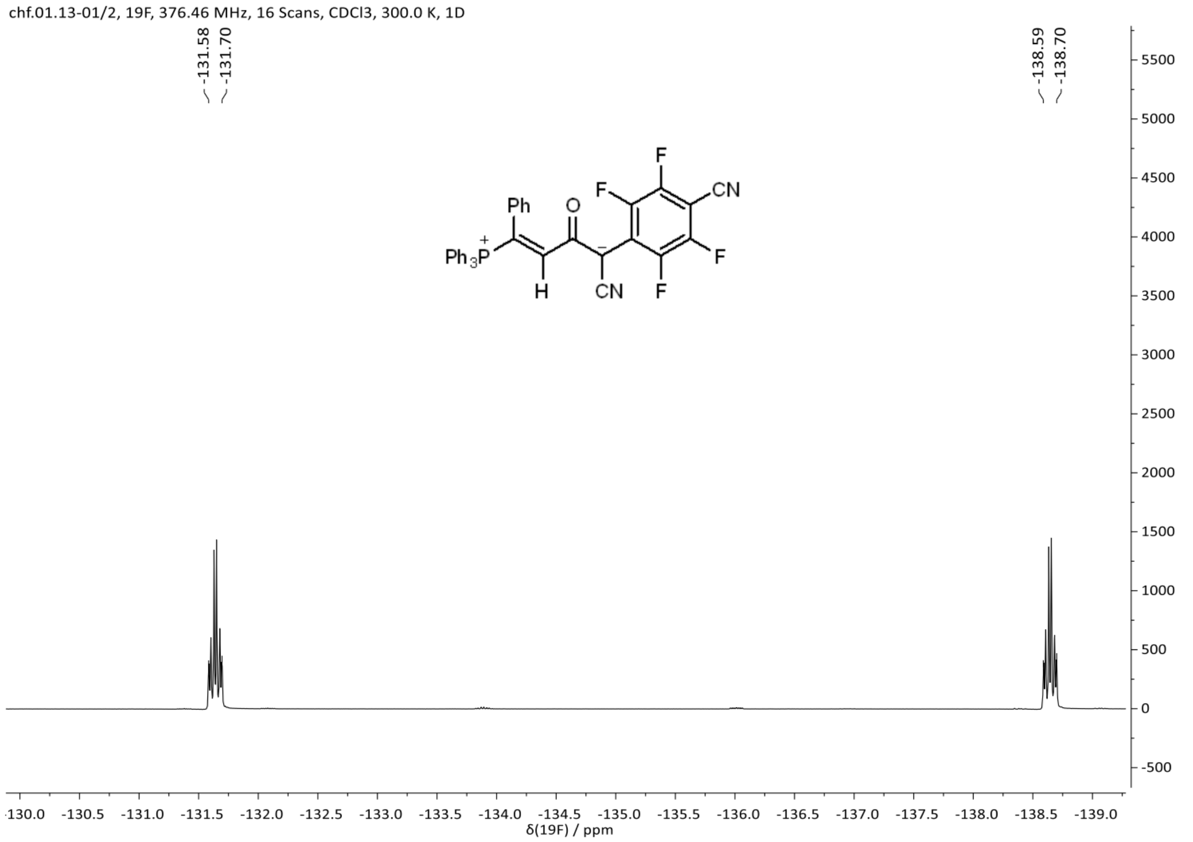

^{31}P NMR

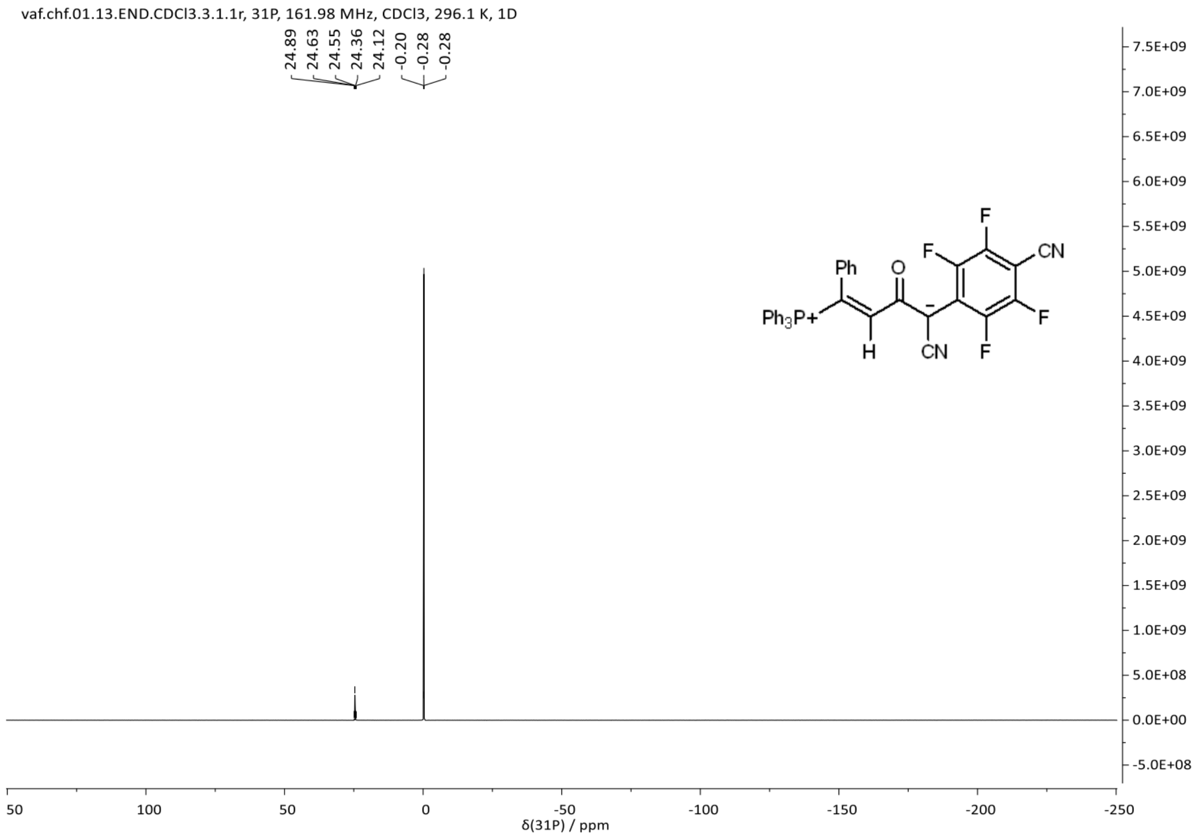

IR

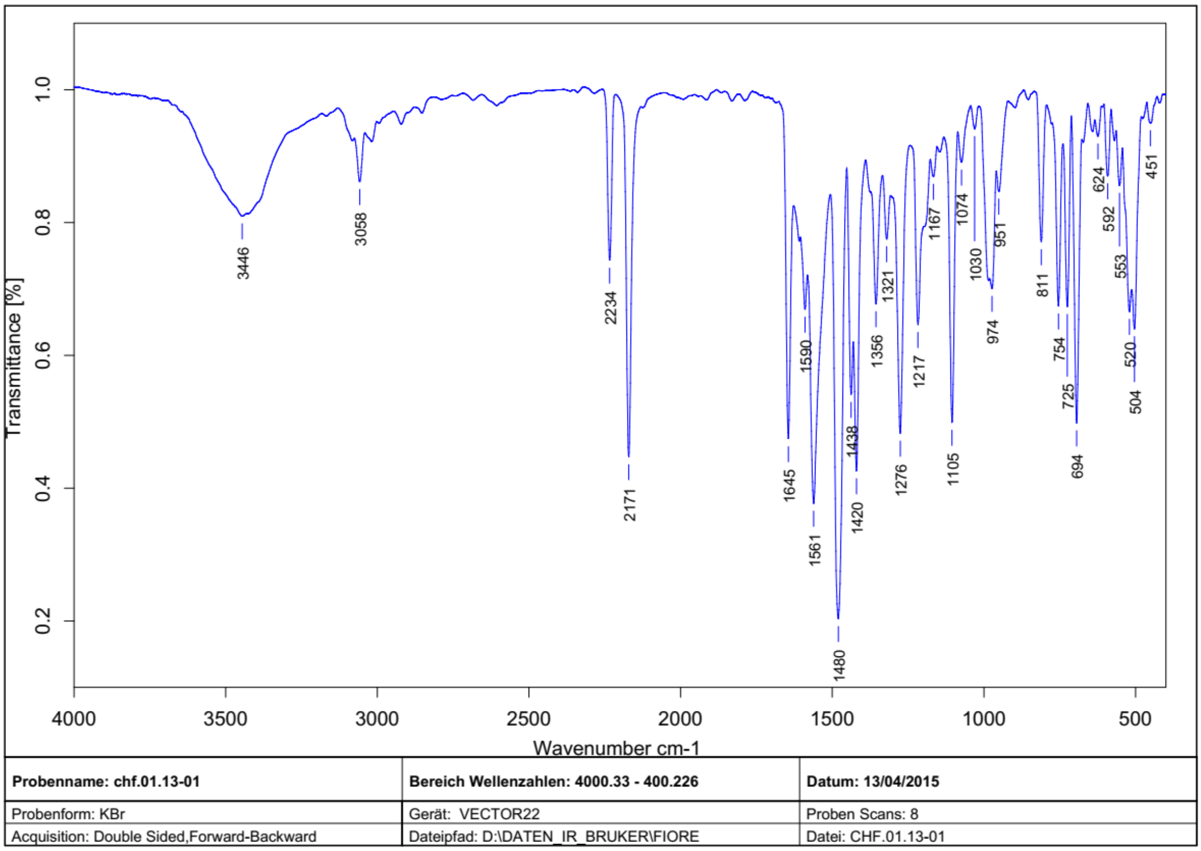

**Probenname: chf.01.13-01**	**Bereich Wellenzahlen: 4000.33 - 400.226**	**Datum: 13/04/2015**
Probenform: KBr	Gerät: VECTOR22	Proben Scans: 8
Acquisition: Double Sided,Forward-Backward	Dateipfad: D:\DATEN_IR_BRUKER\FIORE	Datei: CHF.01.13-01

***(Z)*-3f**

^{1}H NMR

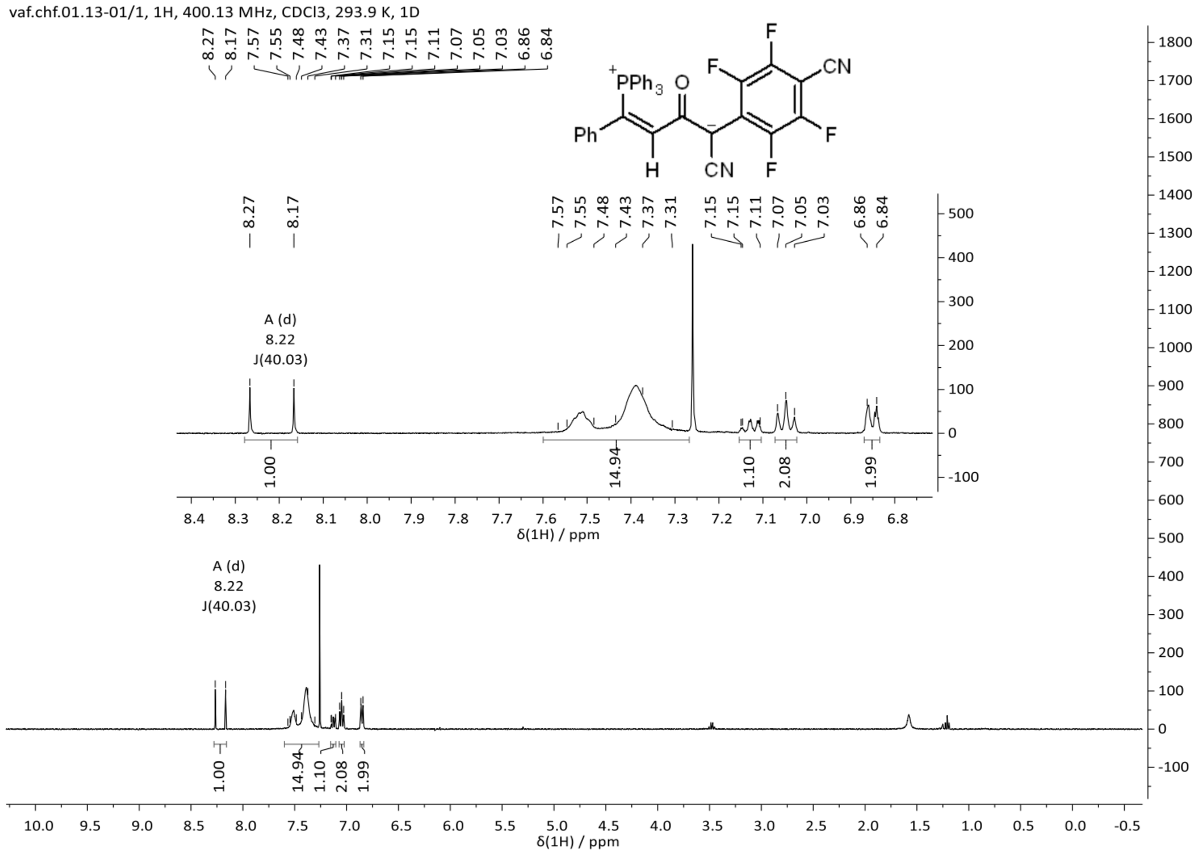
vaf.chf.01.13-01/1, 1H, 400.13 MHz, CDCl3, 293.9 K, 1D
A (d)
8.22
J(40.03)
δ(1H) / ppm

^{19}F NMR

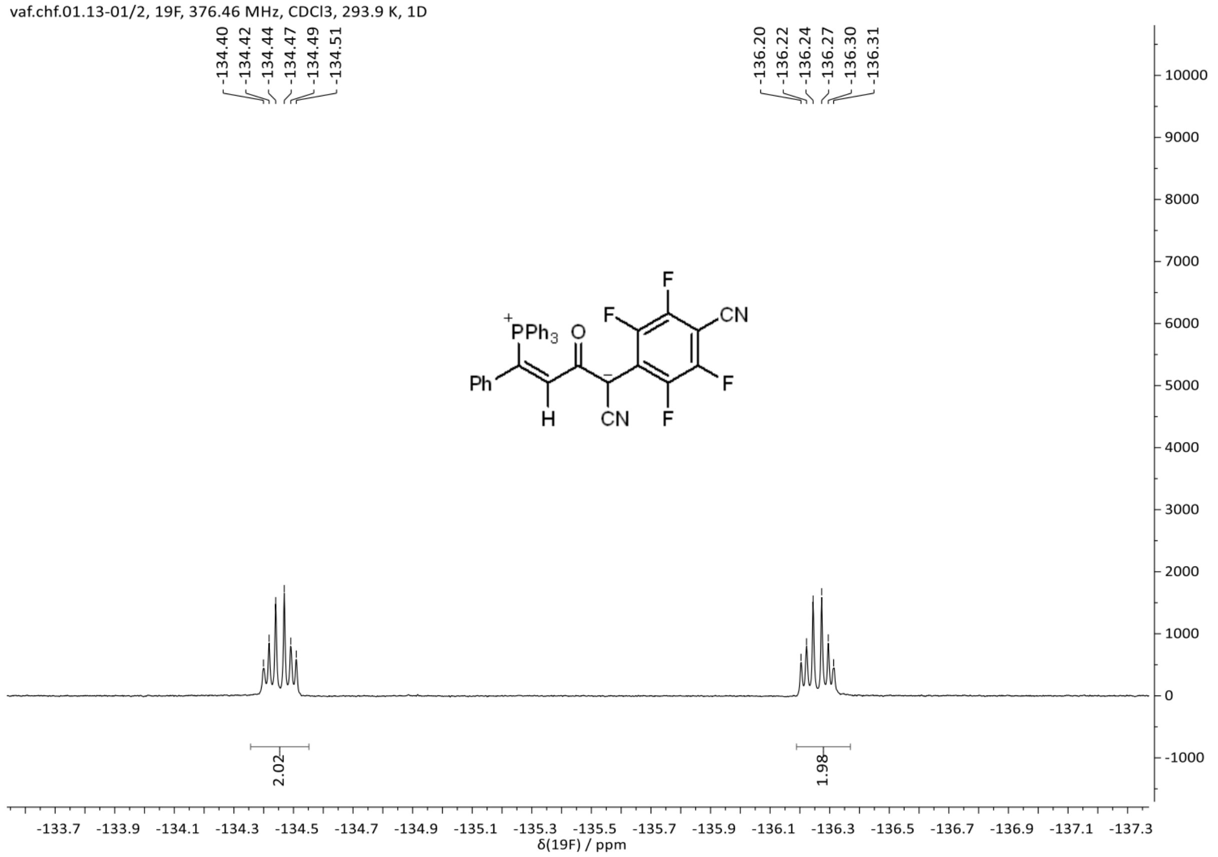

^{31}P NMR

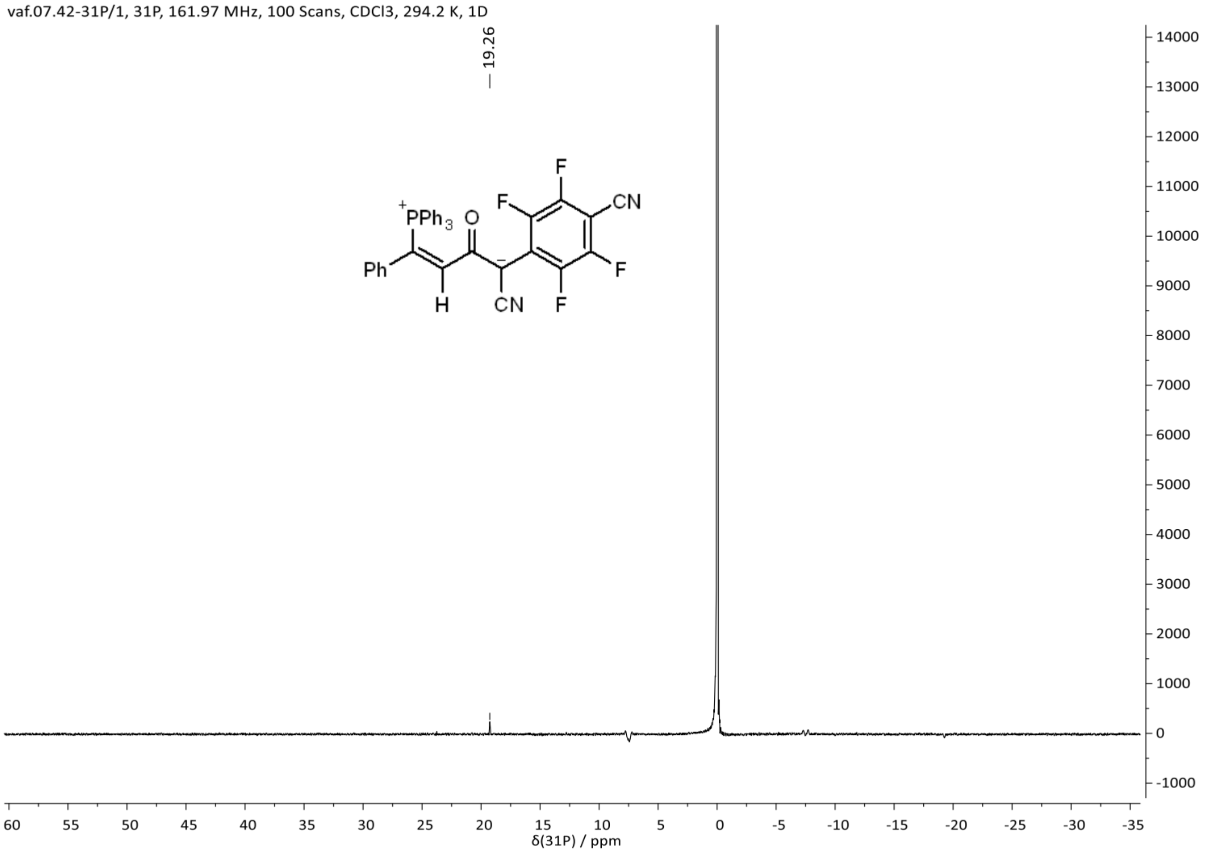

4.7. Betaines ((*E*)-3g)

^{1}H NMR

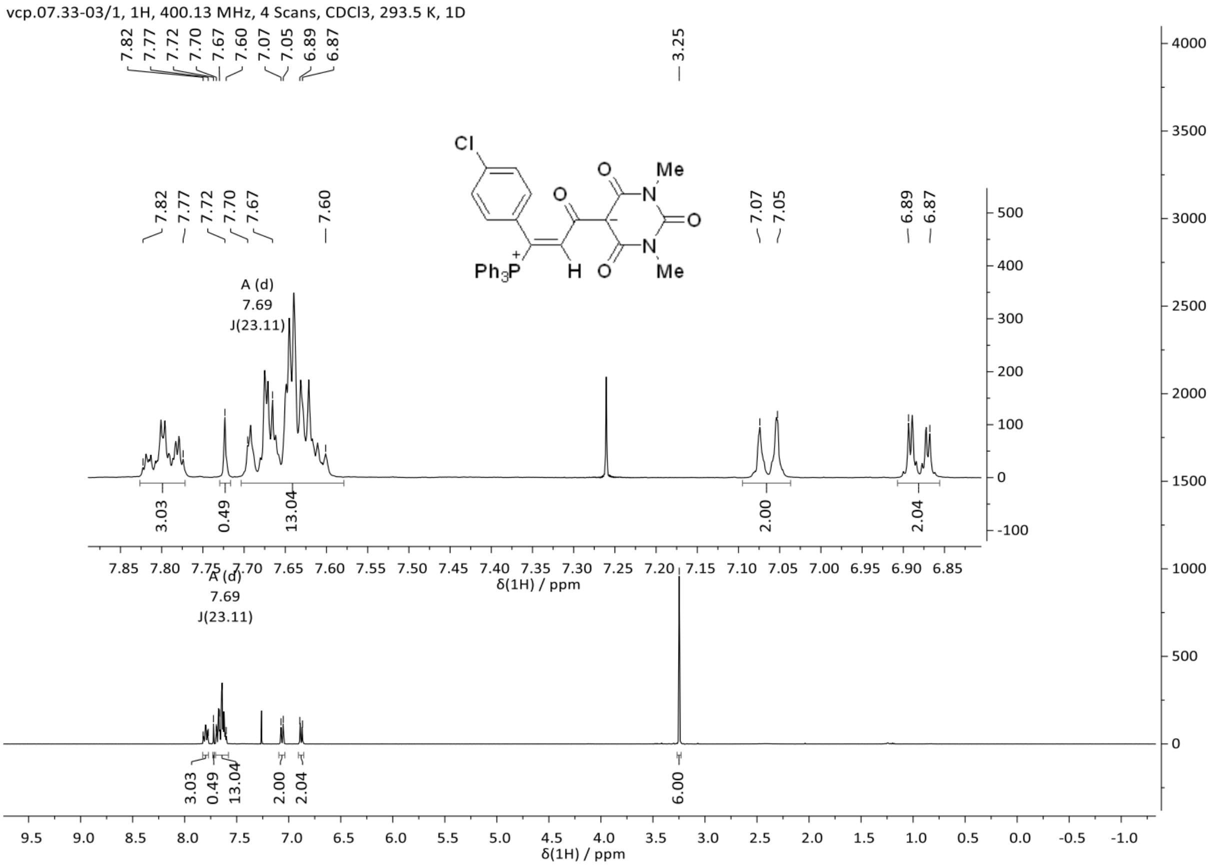

^{13}C NMR

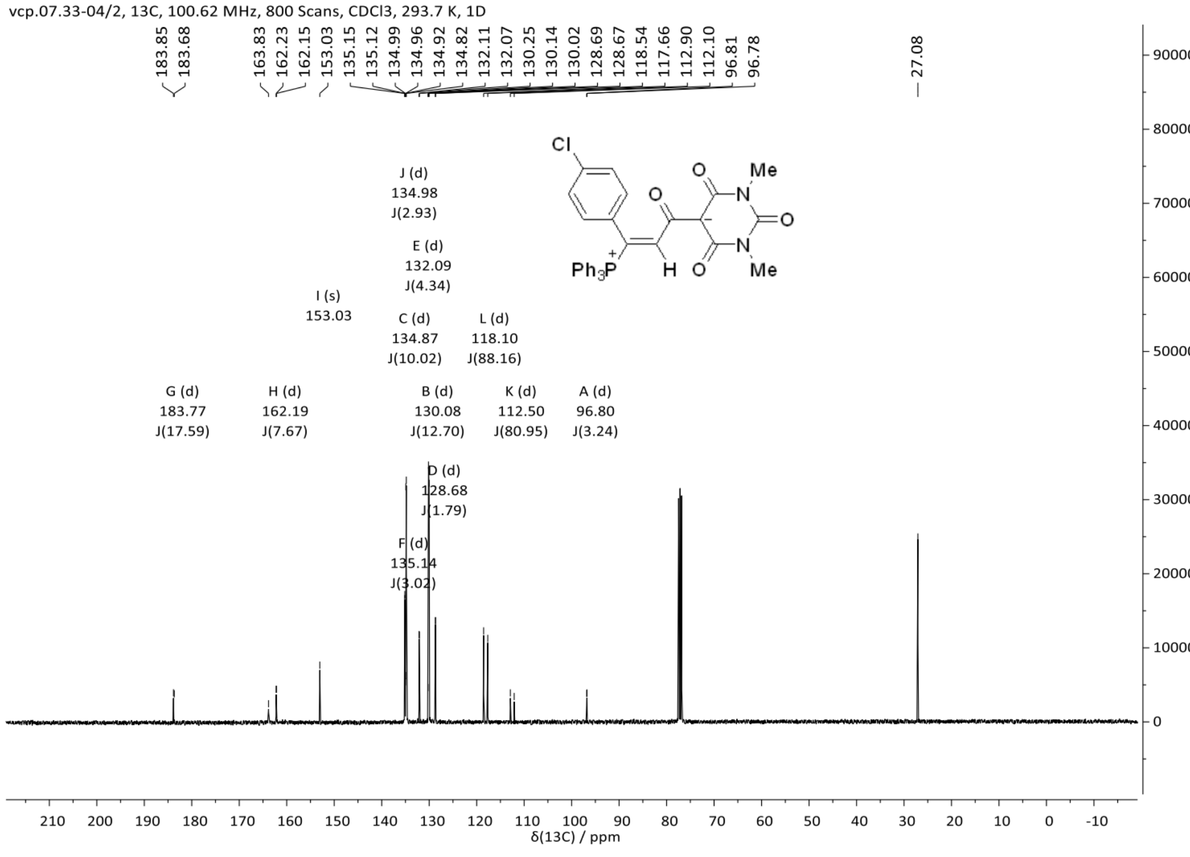

^{31}P NMR

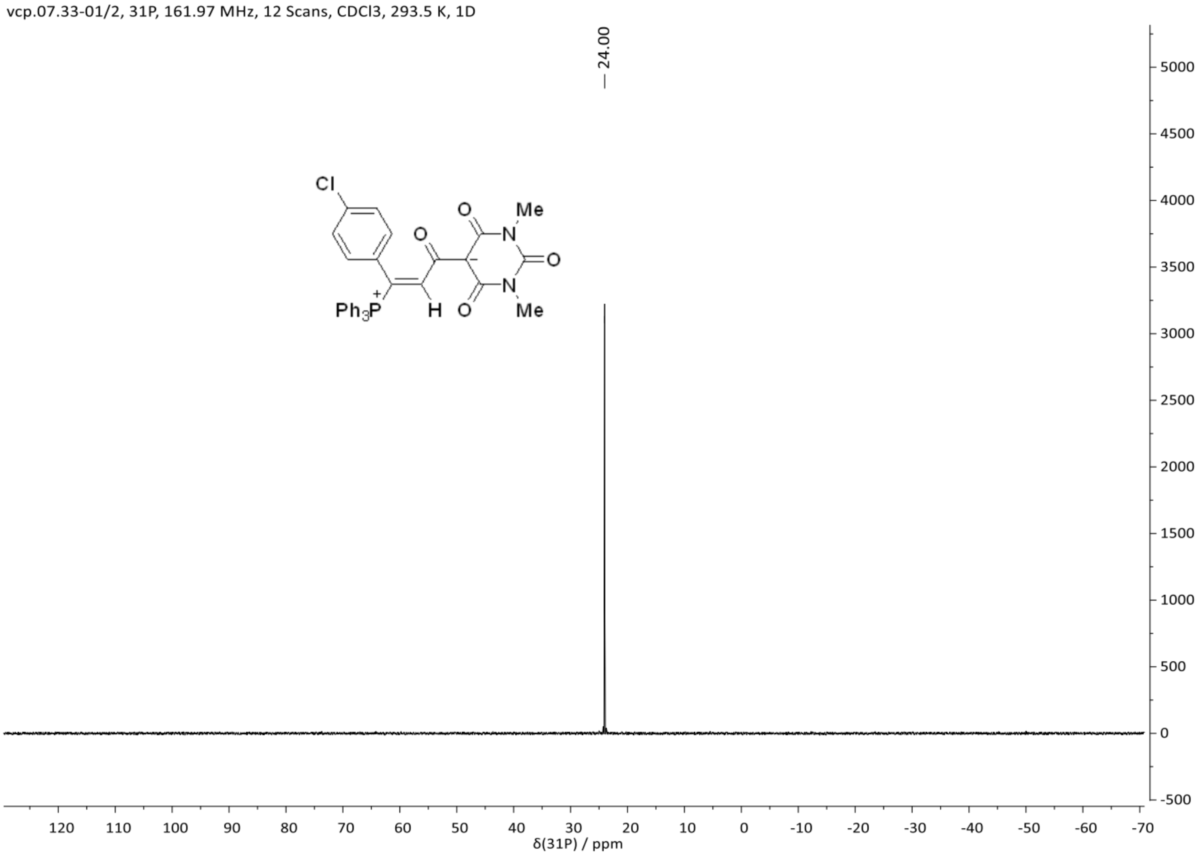

IR

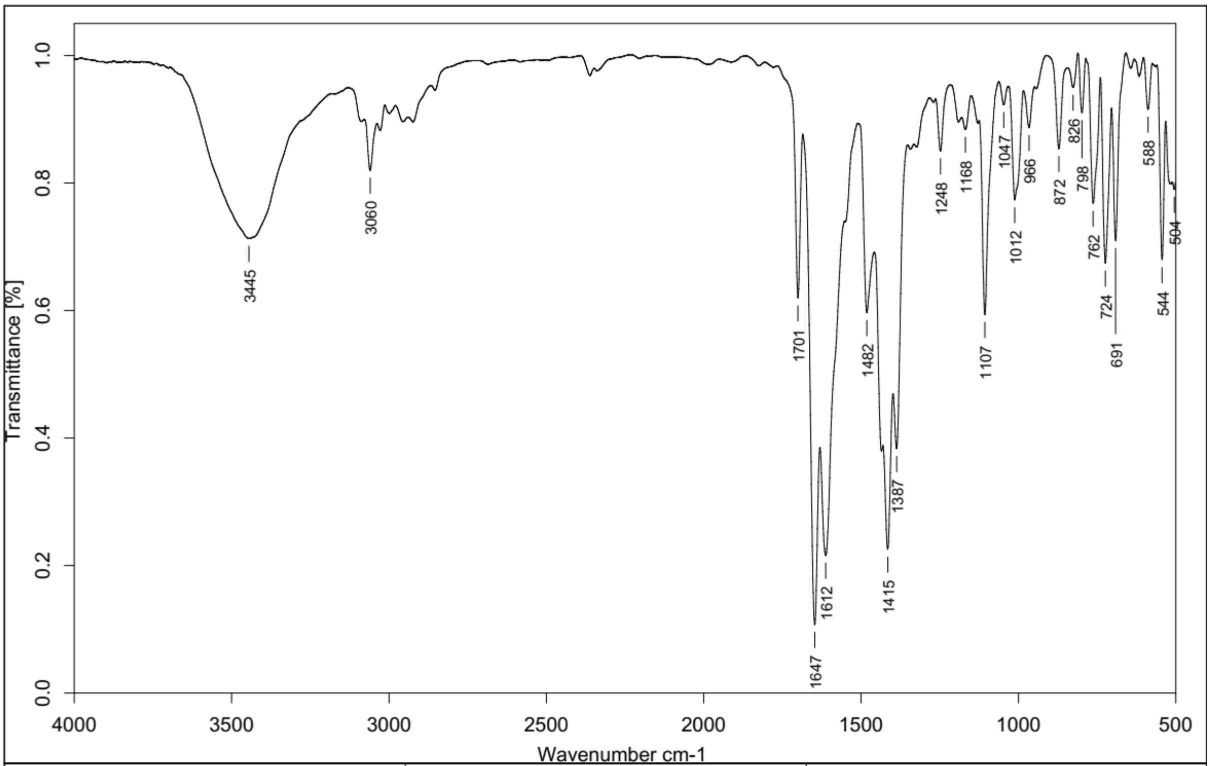

Probenname: vcp.07.33-1	Bereich Wellenzahlen: 4000.33 - 400.226	Datum: 10/03/2016
Probenform: KBr	Gerät: VECTOR22	Proben Scans: 8
Acquisition: Double Sided,Forward-Backward	Dateipfad: D:\DATEN_IR_BRUKER\FIORE	Datei: VCP.07.33-1

4.8 Betaines (*E*)- and (*Z*)-3h

^{1}H NMR

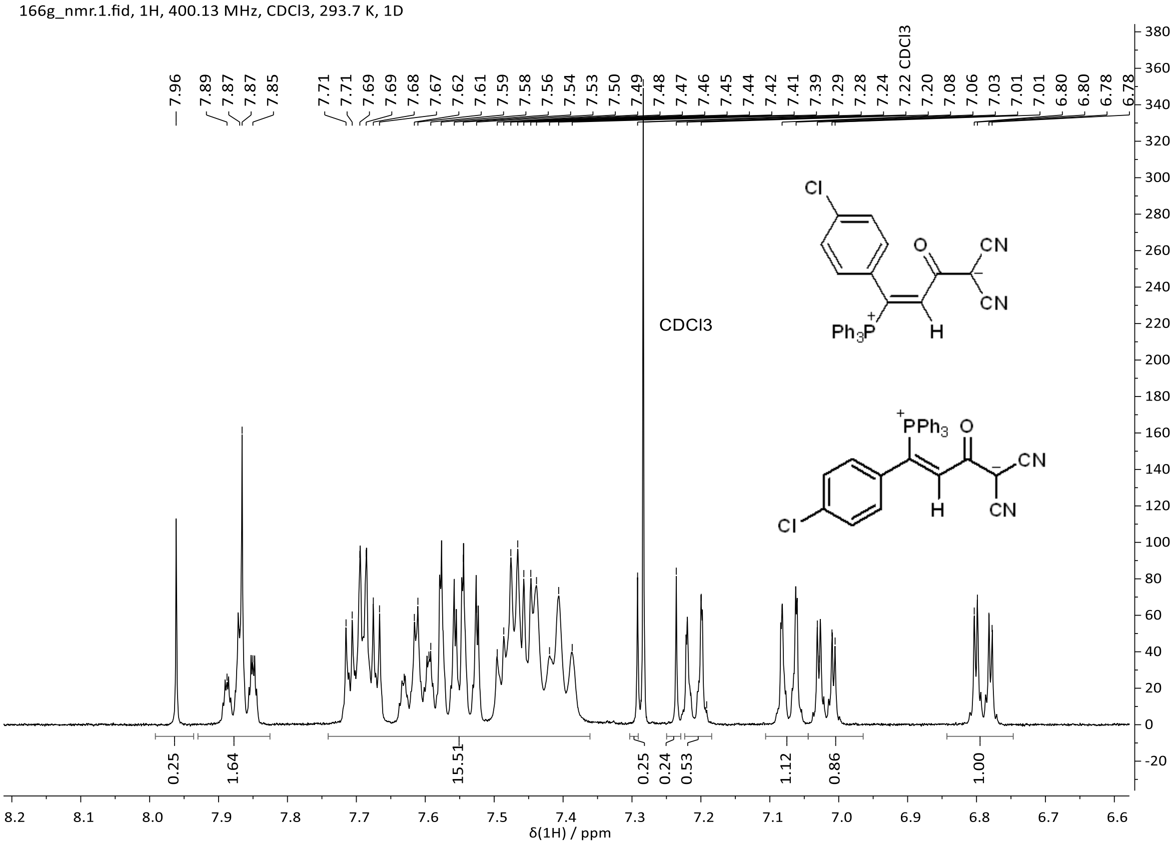

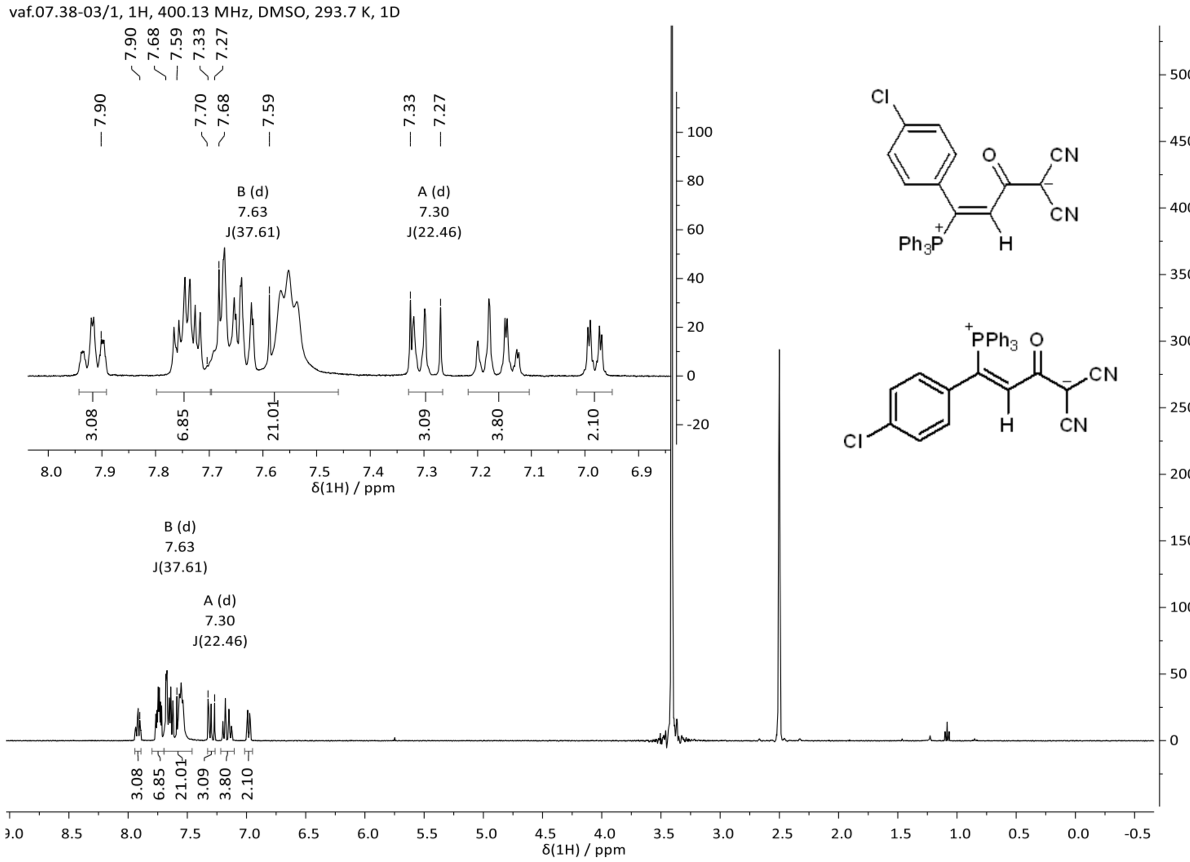

^{13}C NMR

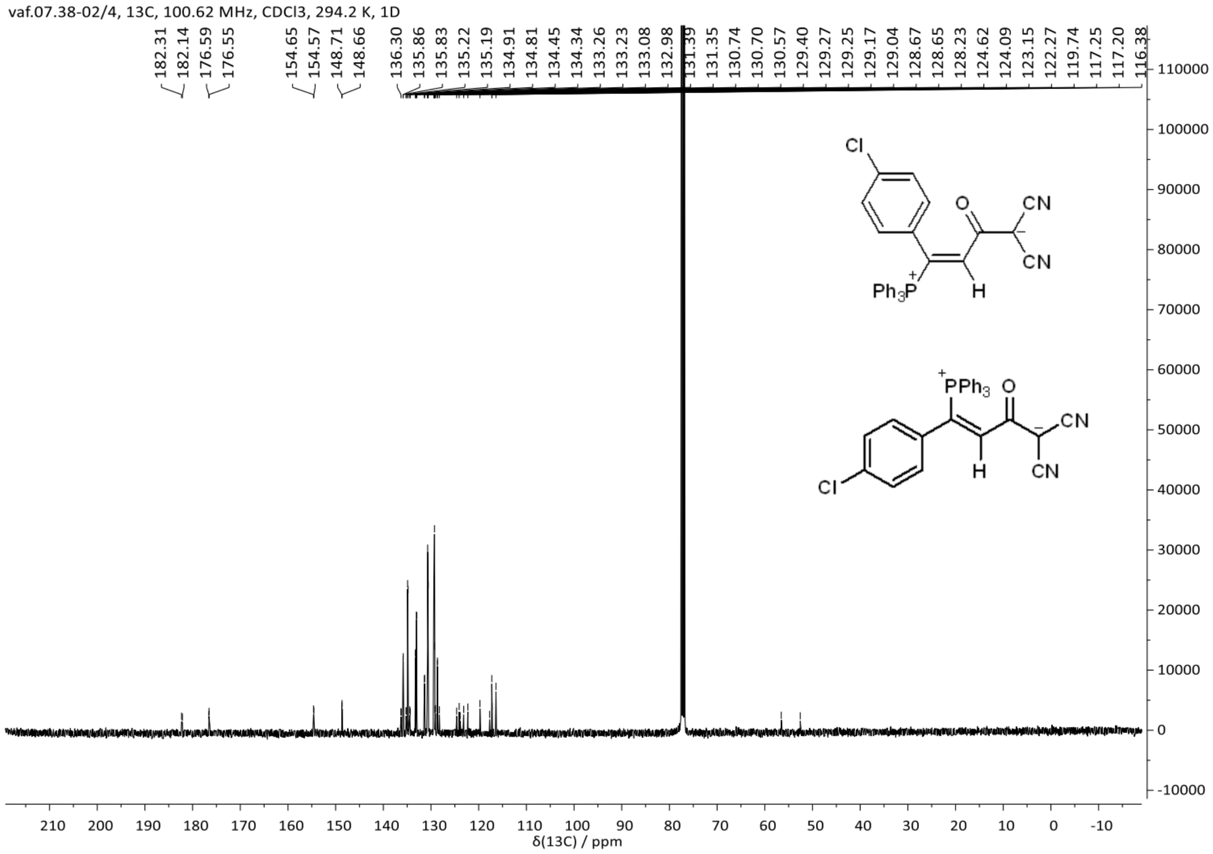
vaf.07.38-02/4, 13C, 100.62 MHz, CDCl3, 294.2 K, 1D
δ(13C) / ppm

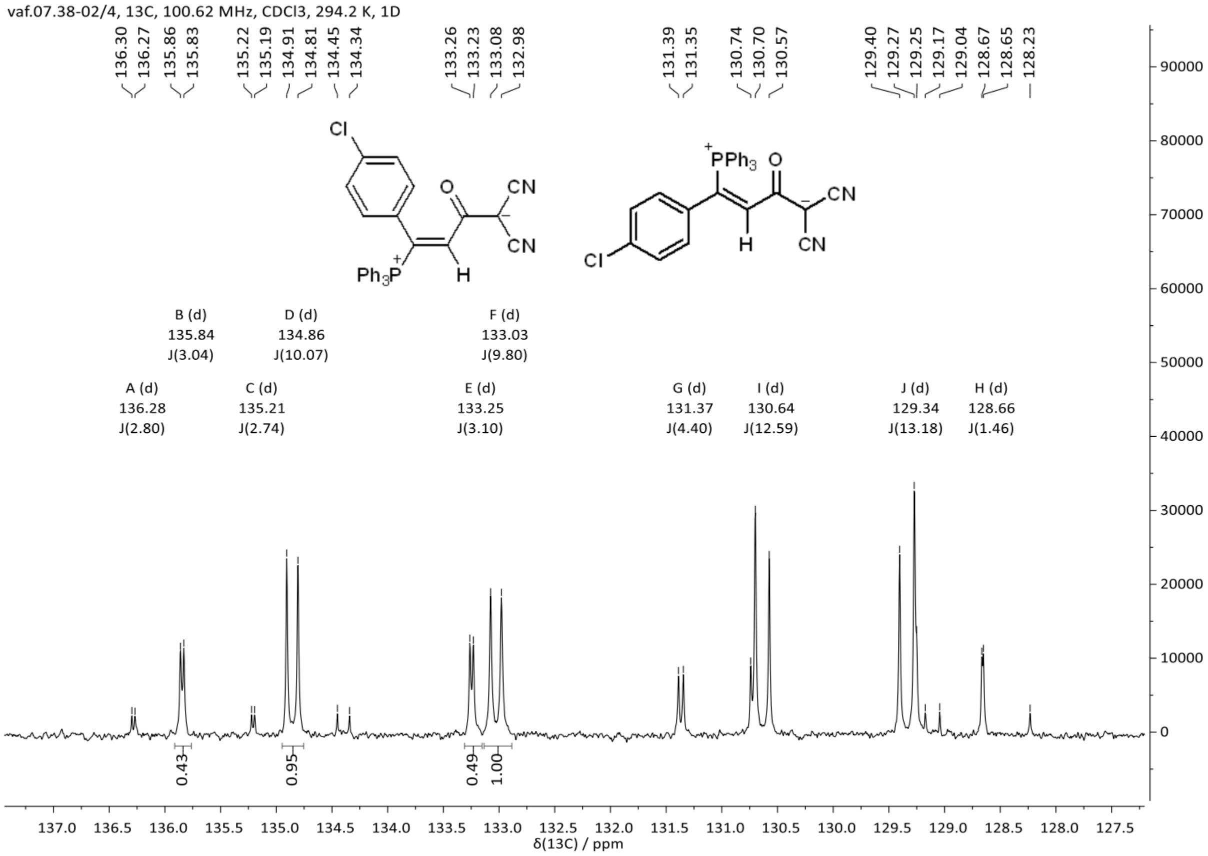
vaf.07.38-02/4, 13C, 100.62 MHz, CDCl3, 294.2 K, 1D
A (d) 136.28 J(2.80)
B (d) 135.84 J(3.04)
C (d) 135.21 J(2.74)
D (d) 134.86 J(10.07)
E (d) 133.25 J(3.10)
F (d) 133.03 J(9.80)
G (d) 131.37 J(4.40)
I (d) 130.64 J(12.59)
J (d) 129.34 J(13.18)
H (d) 128.66 J(1.46)
δ(13C) / ppm

^{31}P NMR

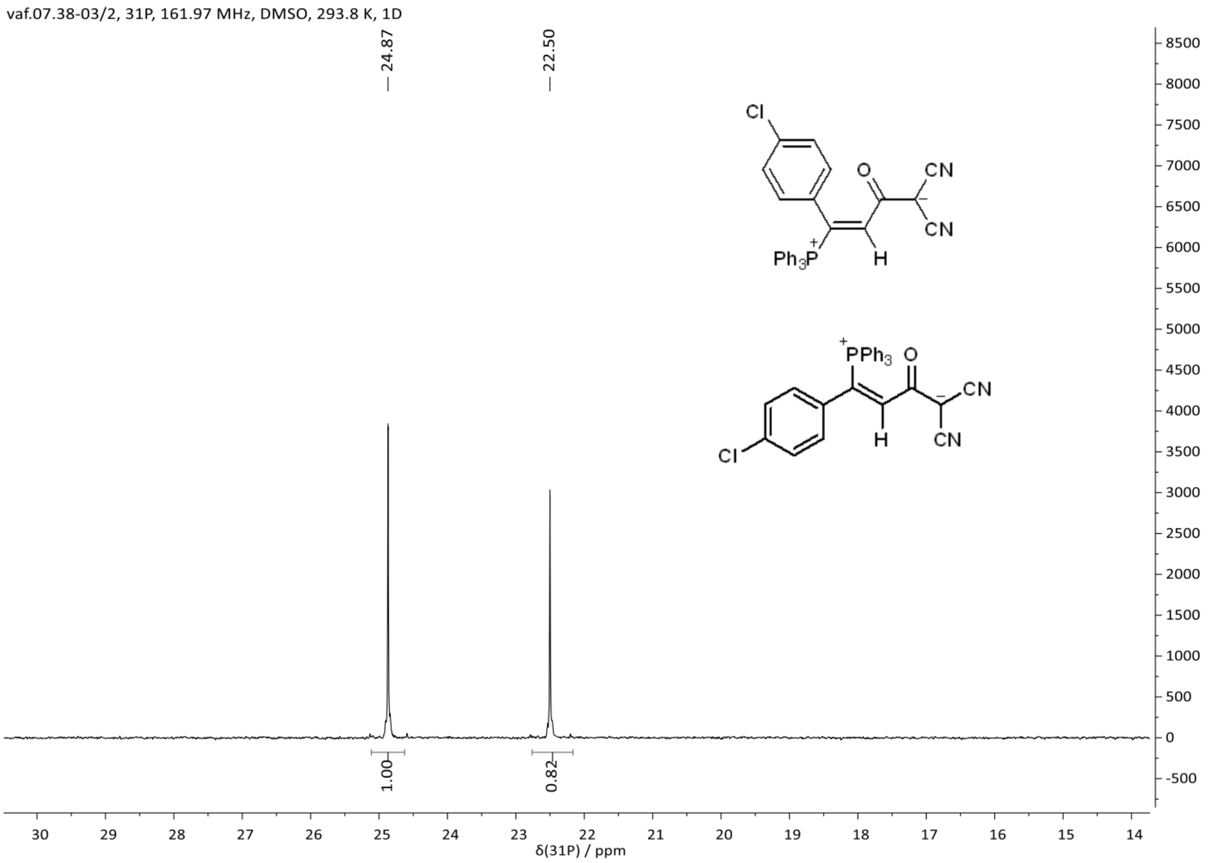

IR

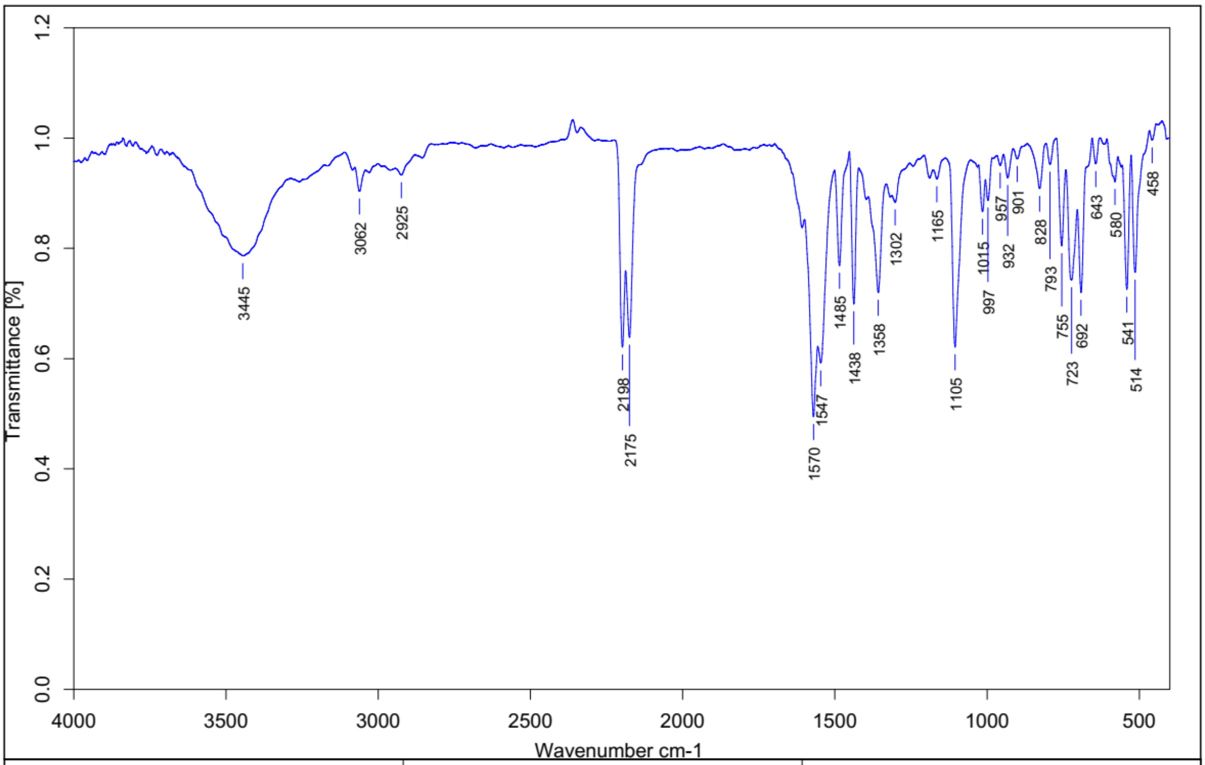

**Probenname: vaf.07.38-1**	**Bereich Wellenzahlen: 4000.33 - 400.226**	**Datum: 17/03/2017**
Probenform: KBr	Gerät: VECTOR22	Proben Scans: 8
Acquisition: Double Sided,Forward-Backward	Dateipfad: D:\DATEN_IR_BRUKER\FIORE	Datei: VAF.07.38-1

4.9. Betaines (*E*)- and (Z)-3i (mixture)

^{1}H NMR

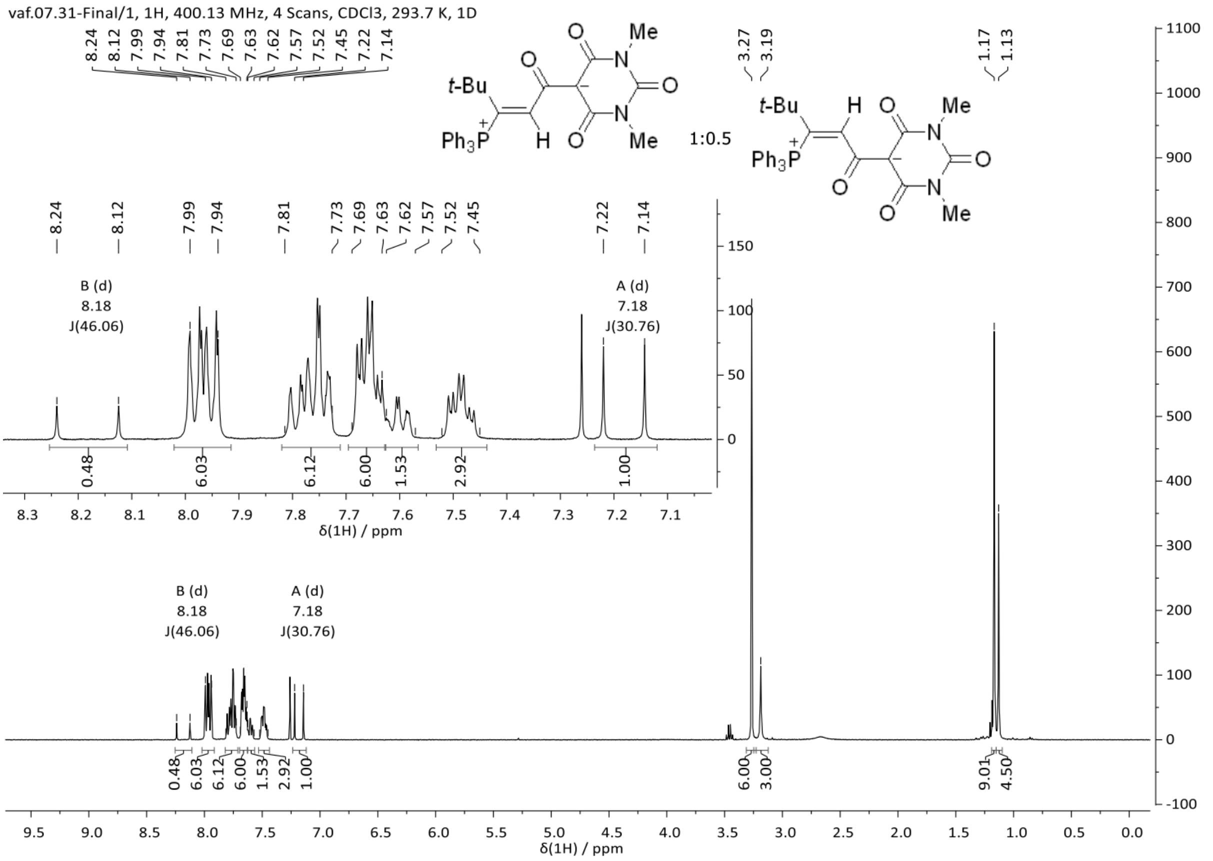

^{13}C NMR

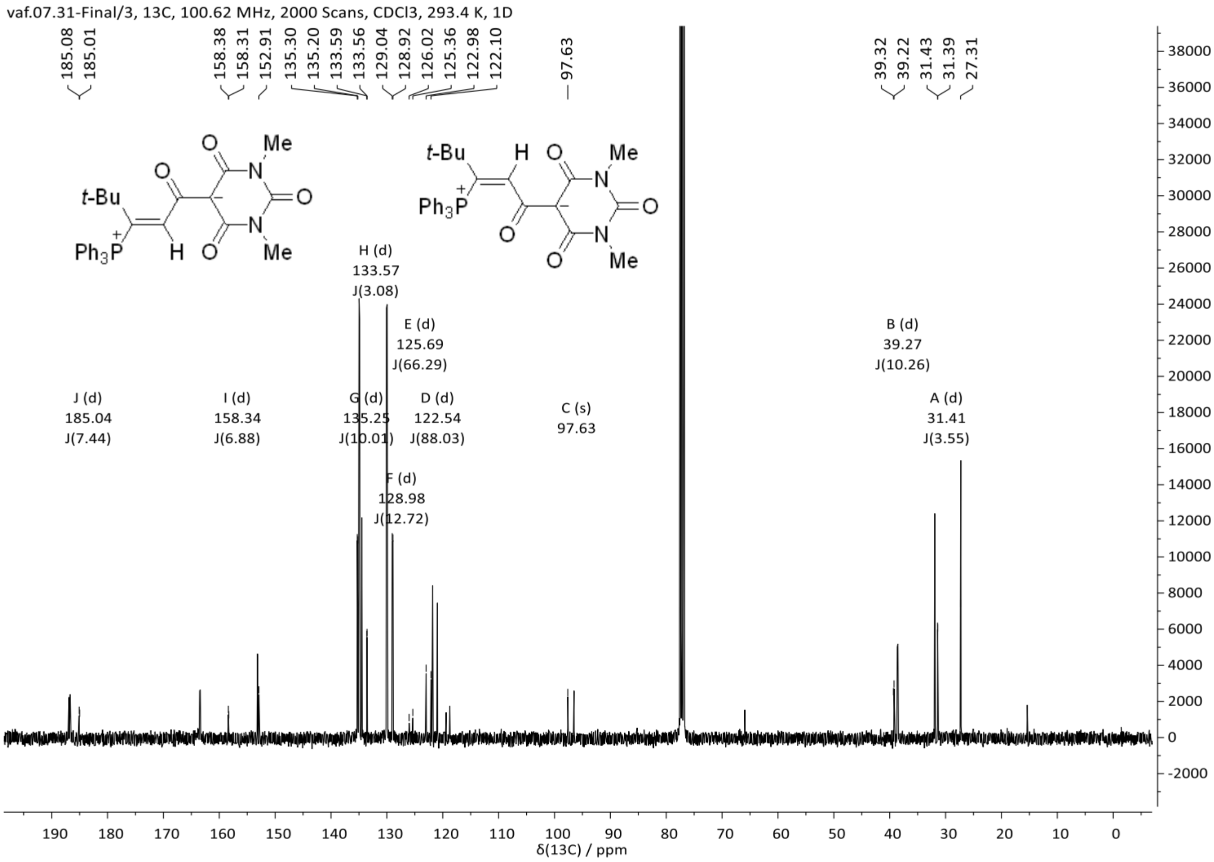

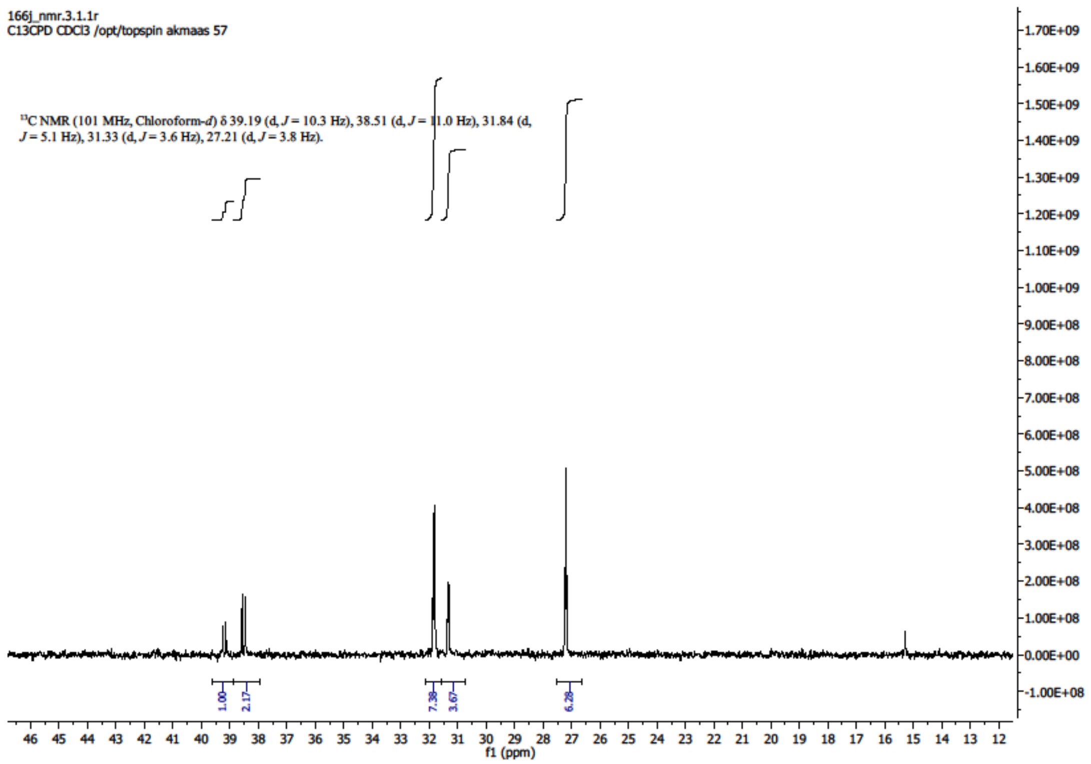
166j_nmr.3.1.1r
C13CPD CDCl3 /opt/topspin akmaas 57
^{13}C NMR (101 MHz, Chloroform-*d*) δ 39.19 (d, *J* = 10.3 Hz), 38.51 (d, *J* = 11.0 Hz), 31.84 (d, *J* = 5.1 Hz), 31.33 (d, *J* = 3.6 Hz), 27.21 (d, *J* = 3.8 Hz).
1.00
2.17
7.38
3.67
6.28
f1 (ppm)

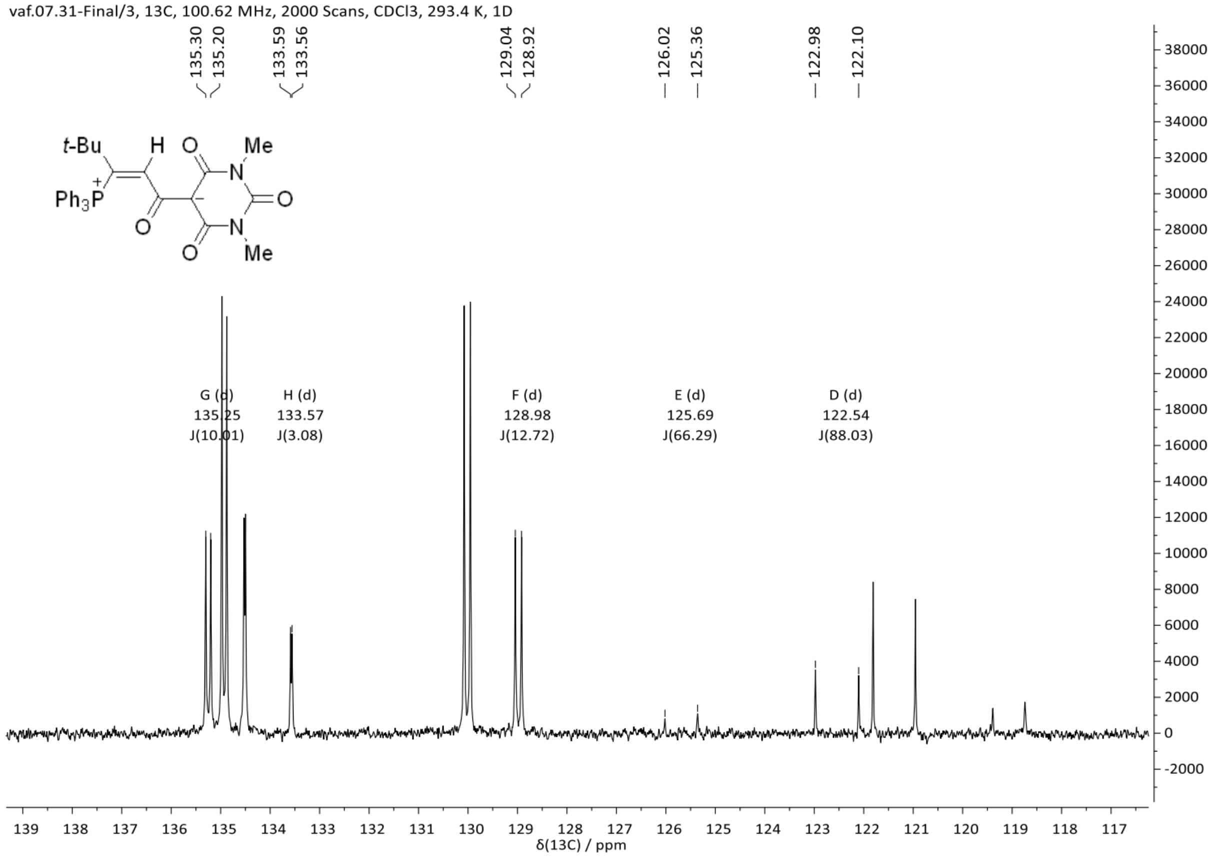
vaf.07.31-Final/3, 13C, 100.62 MHz, 2000 Scans, CDCl3, 293.4 K, 1D
135.30
135.20
133.59
133.56
129.04
128.92
126.02
125.36
122.98
122.10
t-Bu
H
O
Me
N
Ph3P
+
O
N
O
Me
G (d)
135.25
J(10.01)
H (d)
133.57
J(3.08)
F (d)
128.98
J(12.72)
E (d)
125.69
J(66.29)
D (d)
122.54
J(88.03)
δ(13C) / ppm

^{31}P NMR

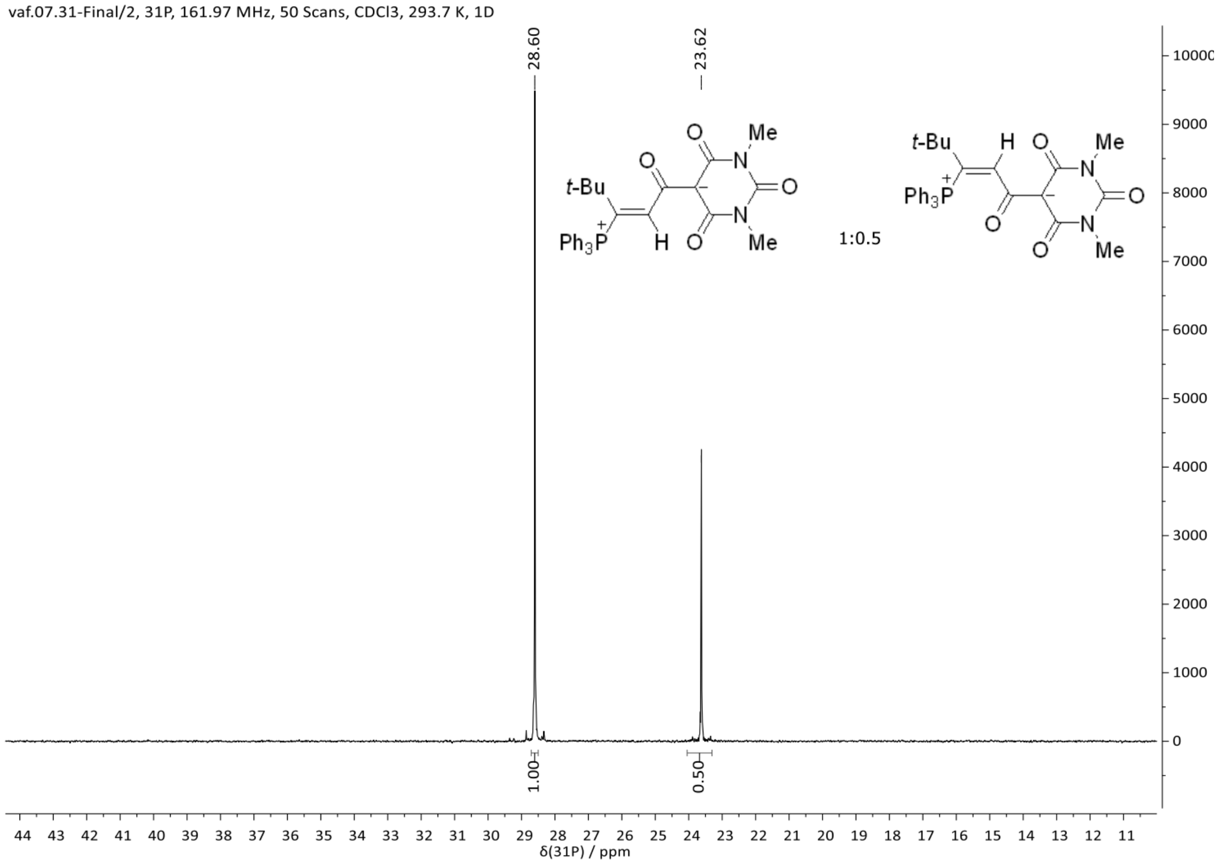

vaf.07.31-Final/2, 31P, 161.97 MHz, 50 Scans, CDCl3, 293.7 K, 1D
28.60
23.62
1:0.5
t-Bu
Ph3P
H
O
N
Me
1.00
0.50
δ(31P) / ppm

IR

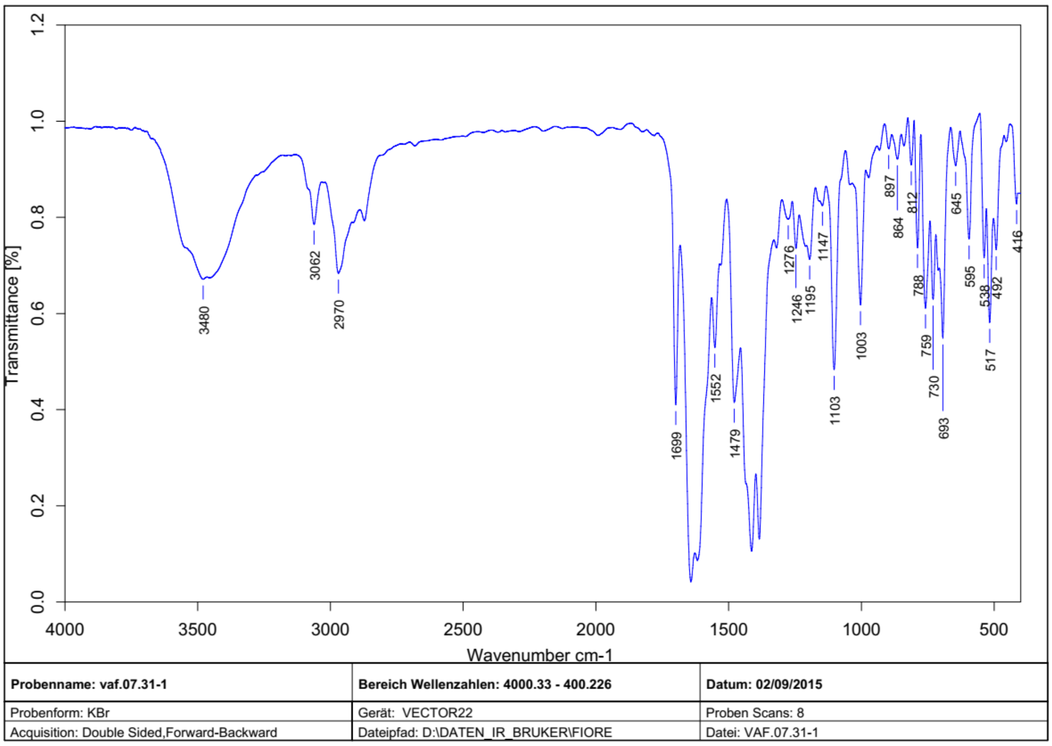

Transmittance [%]
Wavenumber cm-1
3480
3062
2970
1699
1552
1479
1276
1246
1195
1147
1103
1003
897
864
812
788
759
730
693
645
595
538
517
492
416
Probenname: vaf.07.31-1
Bereich Wellenzahlen: 4000.33 - 400.226
Datum: 02/09/2015
Probenform: KBr
Gerät: VECTOR22
Proben Scans: 8
Acquisition: Double Sided,Forward-Backward
Dateipfad: D:\DATEN_IR_BRUKER\FIORE
Datei: VAF.07.31-1

4.10. Betaine (*E*)-3j

^{1}H NMR

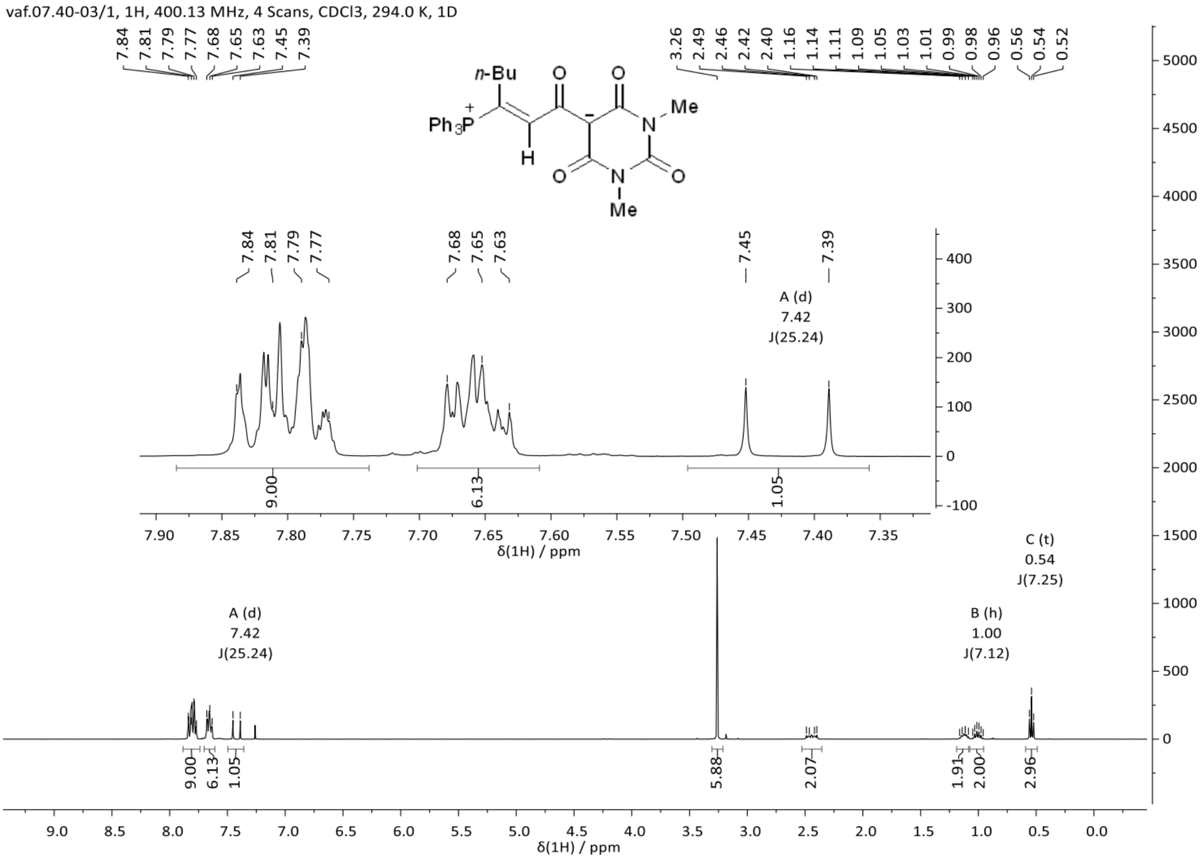

^{13}C NMR

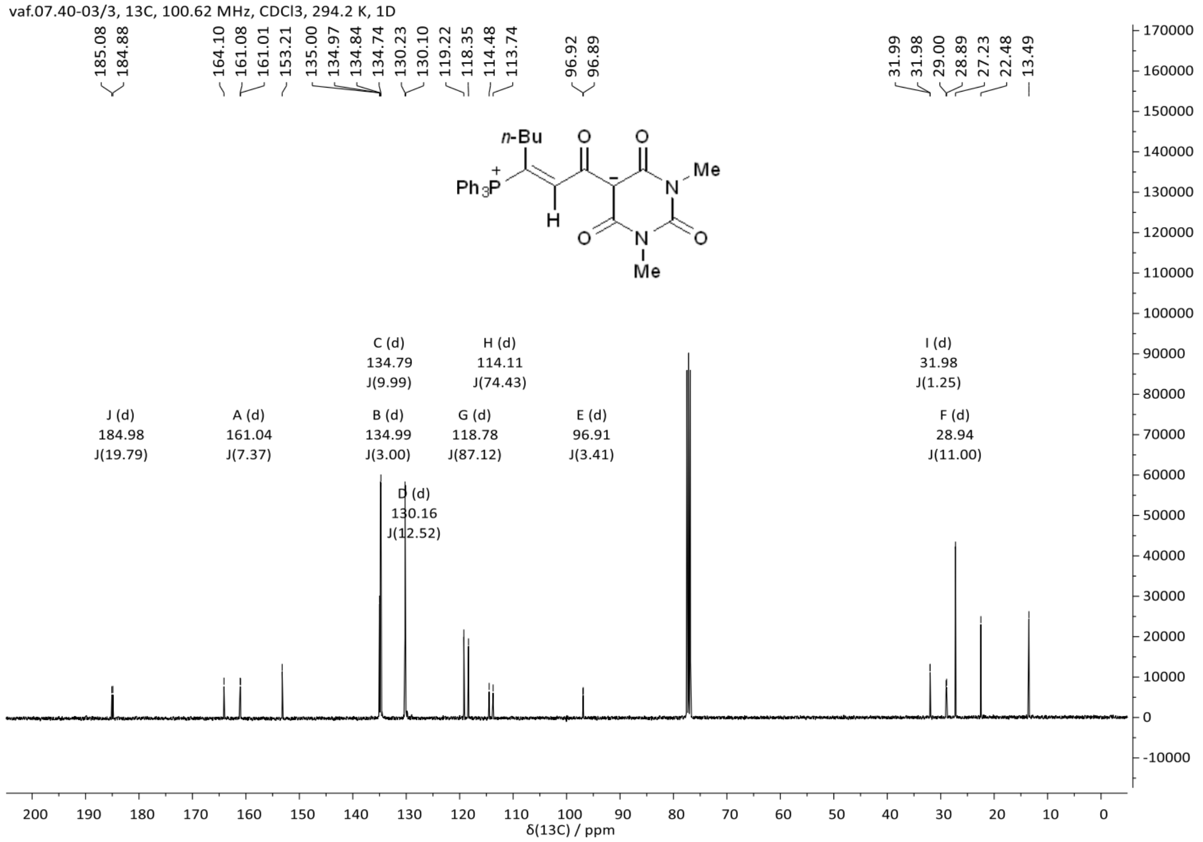

vaf.07.40-03/3, 13C, 100.62 MHz, CDCl3, 294.2 K, 1D
185.08
184.88
164.10
161.08
161.01
153.21
135.00
134.97
134.84
134.74
130.23
130.10
119.22
118.35
114.48
113.74
96.92
96.89
31.99
31.98
29.00
28.89
27.23
22.48
13.49
n-Bu
Ph3P
H
Me
C (d) 134.79 J(9.99)
H (d) 114.11 J(74.43)
I (d) 31.98 J(1.25)
J (d) 184.98 J(19.79)
A (d) 161.04 J(7.37)
B (d) 134.99 J(3.00)
G (d) 118.78 J(87.12)
E (d) 96.91 J(3.41)
F (d) 28.94 J(11.00)
D (d) 130.16 J(12.52)
δ(13C) / ppm

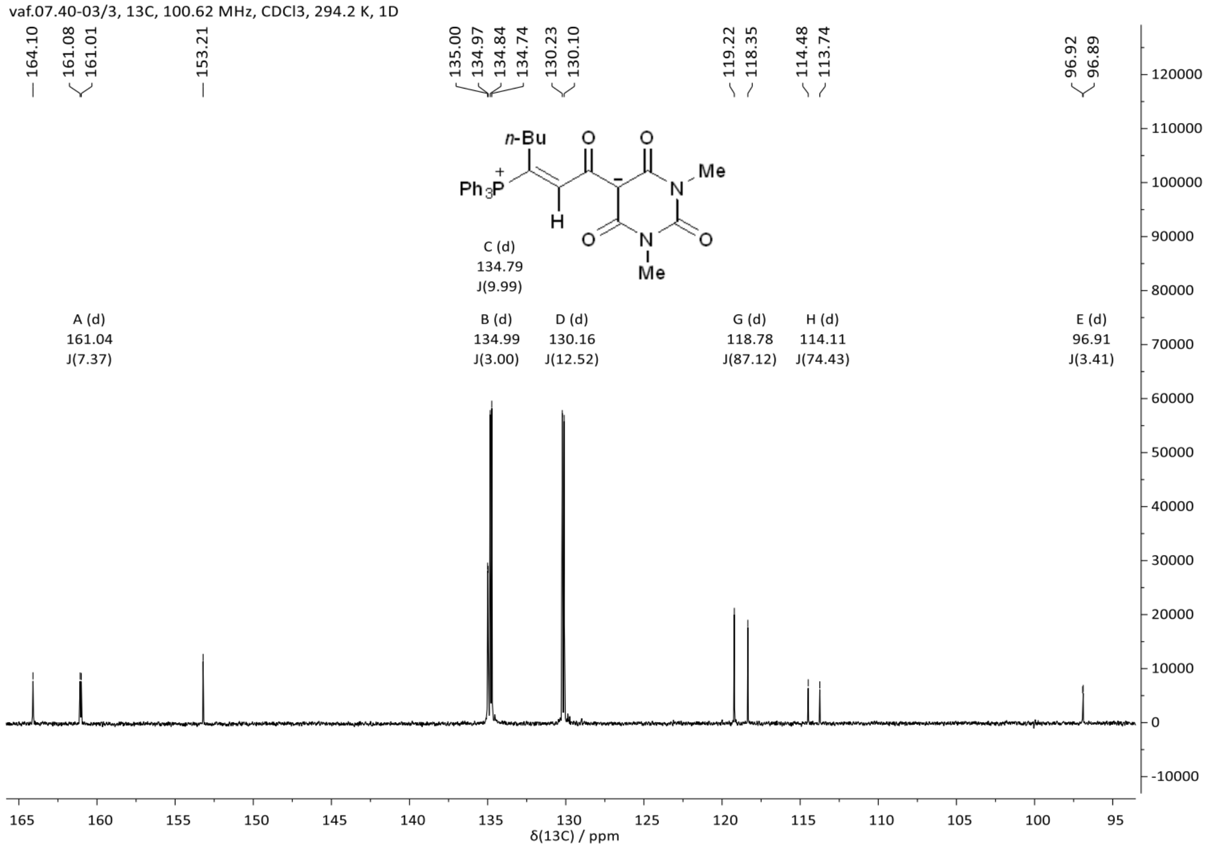

vaf.07.40-03/3, 13C, 100.62 MHz, CDCl3, 294.2 K, 1D
164.10
161.08
161.01
153.21
135.00
134.97
134.84
134.74
130.23
130.10
119.22
118.35
114.48
113.74
96.92
96.89
n-Bu
Ph3P
H
Me
C (d) 134.79 J(9.99)
A (d) 161.04 J(7.37)
B (d) 134.99 J(3.00)
D (d) 130.16 J(12.52)
G (d) 118.78 J(87.12)
H (d) 114.11 J(74.43)
E (d) 96.91 J(3.41)
δ(13C) / ppm

31P NMR

vaf.07.40-31P/1, 31P, 161.97 MHz, 100 Scans, CDCl3, 294.4 K, 1D

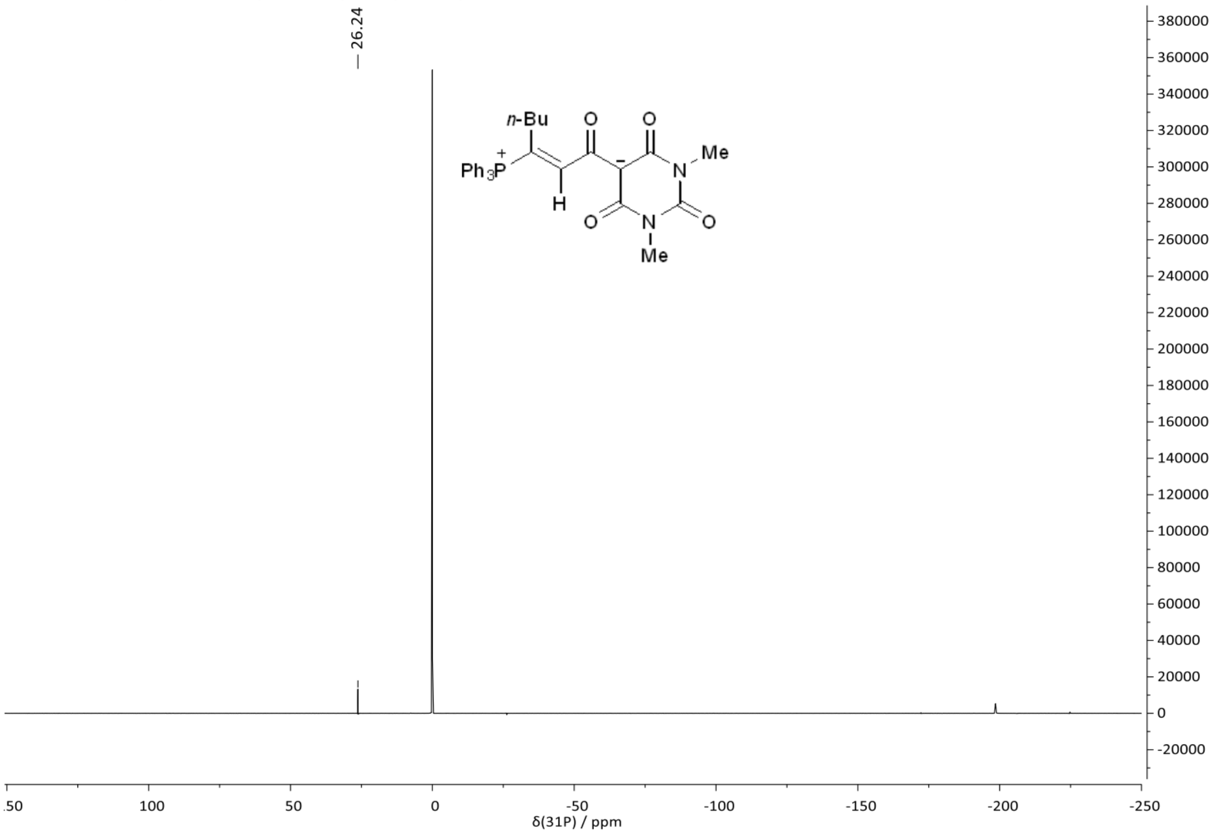

IR

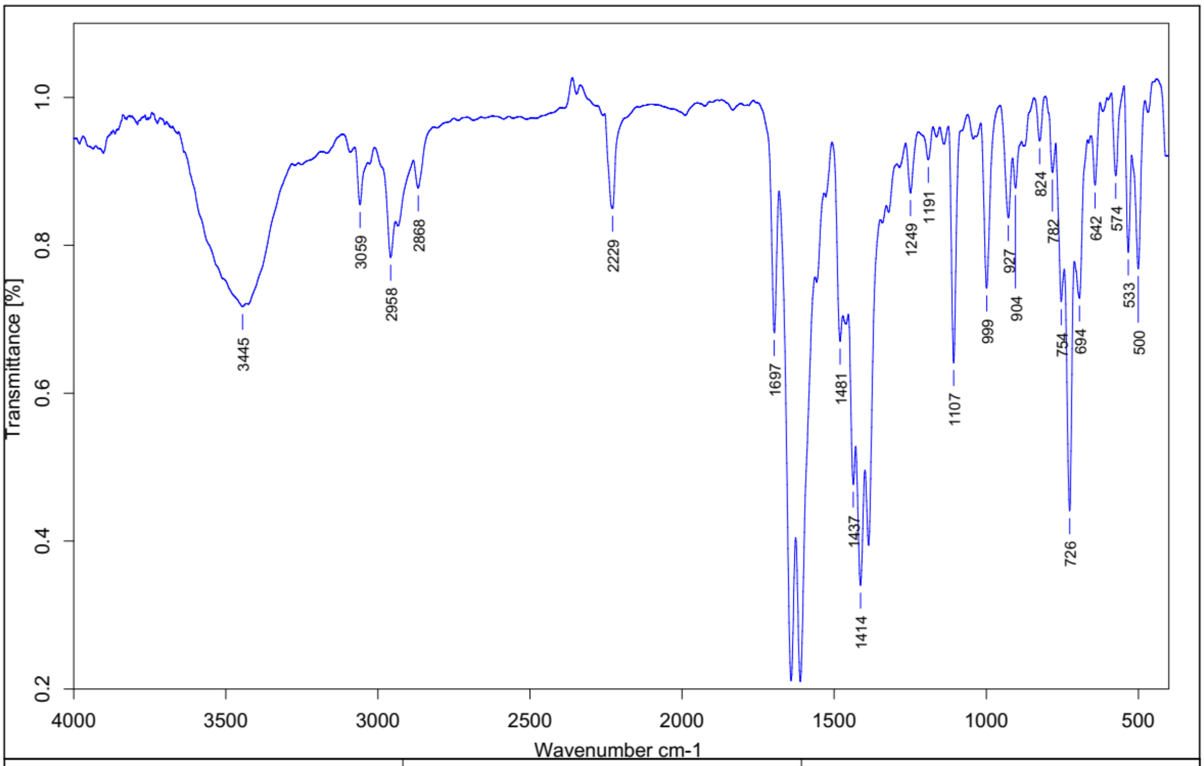

Probenname: vaf.07.40-1	Bereich Wellenzahlen: 4000.33 - 400.226	Datum: 21/03/2017
Probenform: KBr	Gerät: VECTOR22	Proben Scans: 8
Acquisition: Double Sided,Forward-Backward	Dateipfad: D:\DATEN_IR_BRUKER\FIORE	Datei: VAF.07.40-1.0

4.11. Betaines (*E*)- and (*Z*)-3k (mixture of diastereoisomers)

^{1}H NMR

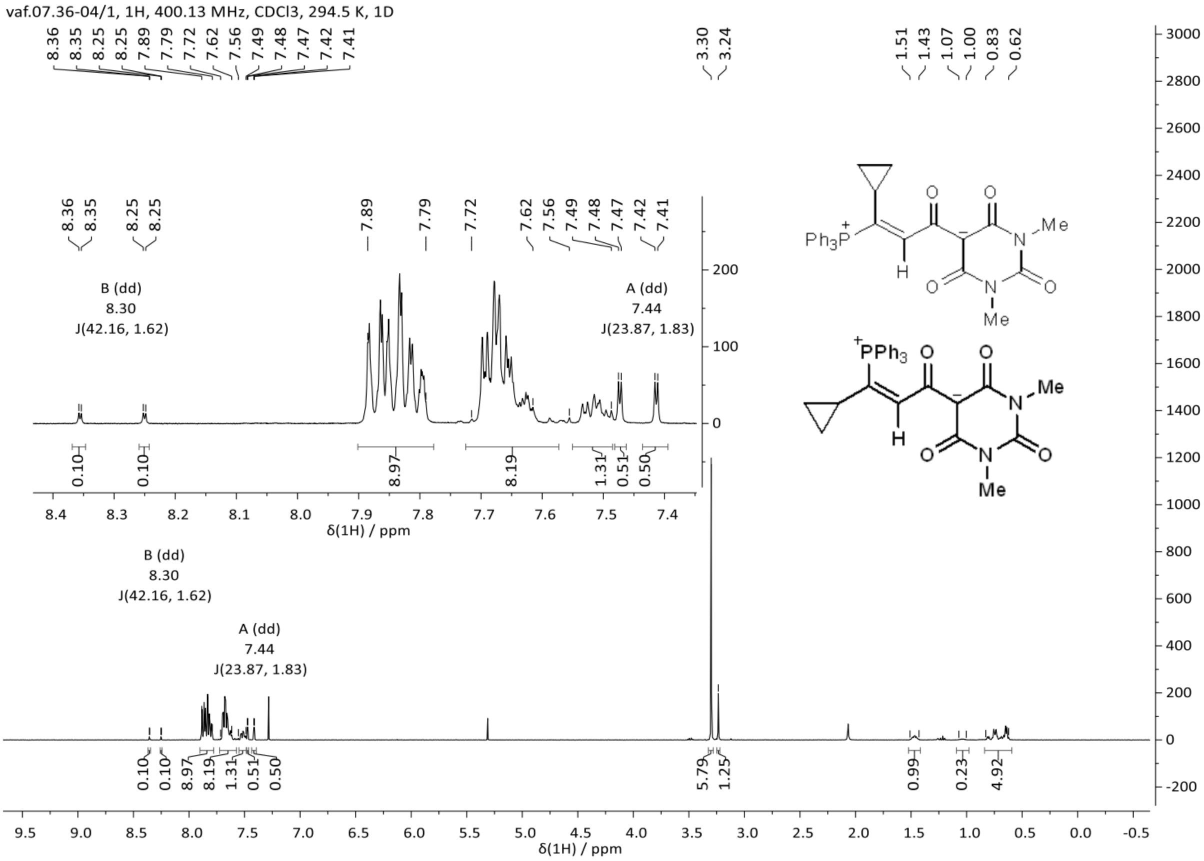

^{13}C NMR

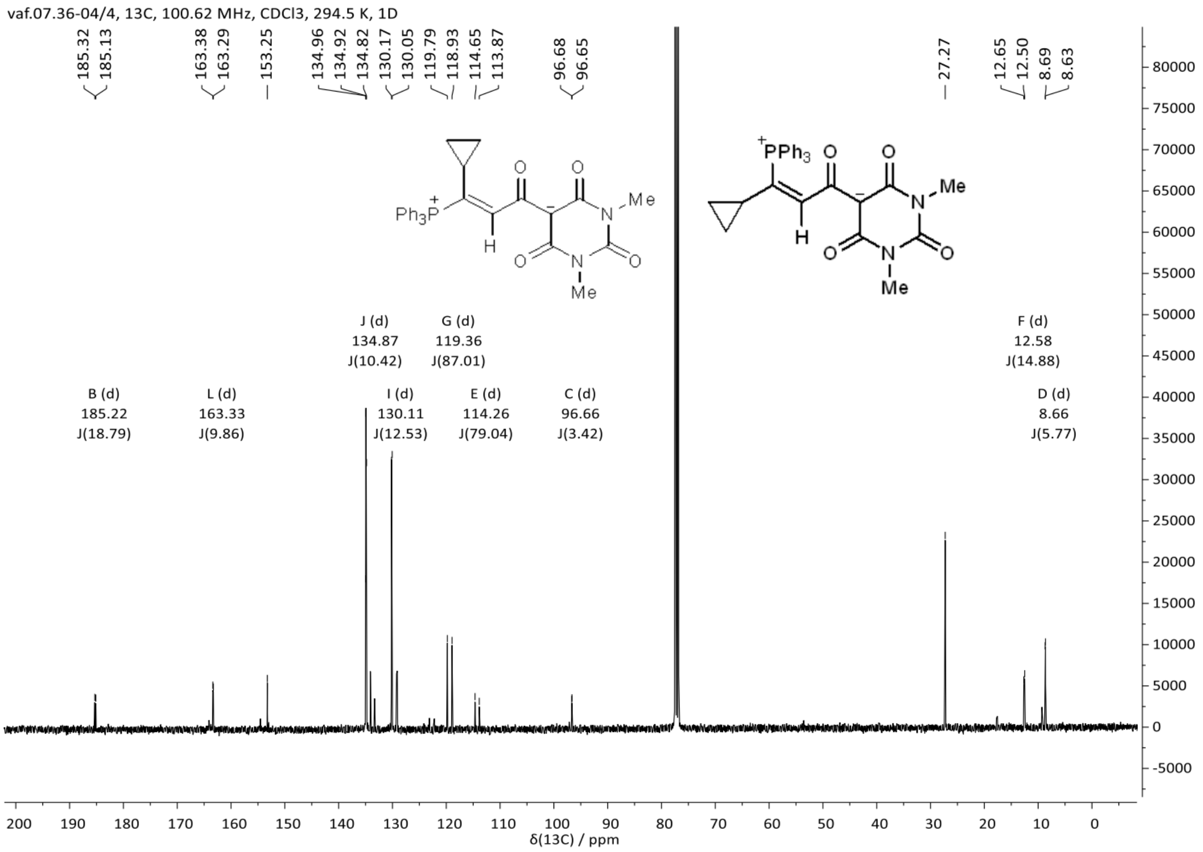

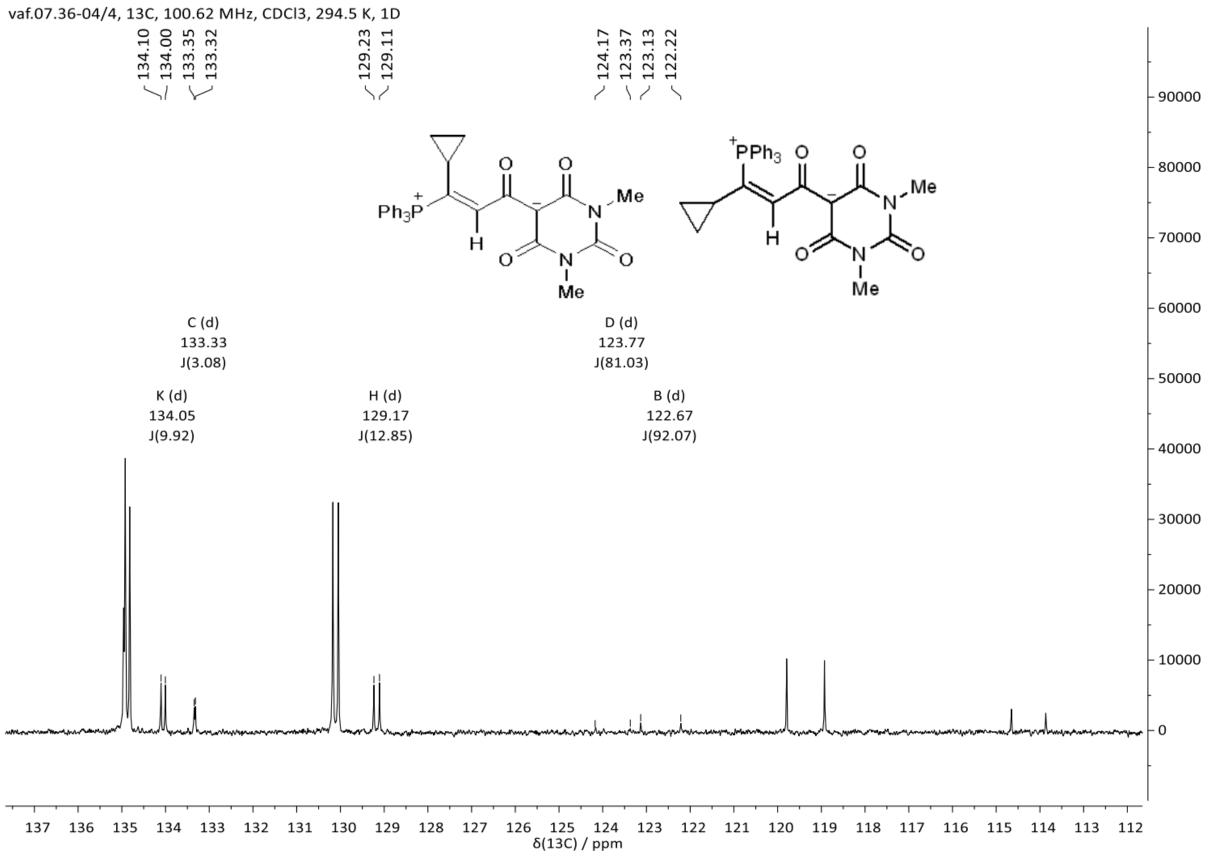
vaf.07.36-04/4, 13C, 100.62 MHz, CDCl3, 294.5 K, 1D
134.10
134.00
133.35
133.32
129.23
129.11
124.17
123.37
123.13
122.22
C (d)
133.33
J(3.08)
D (d)
123.77
J(81.03)
K (d)
134.05
J(9.92)
H (d)
129.17
J(12.85)
B (d)
122.67
J(92.07)
δ(13C) / ppm

^{31}P NMR

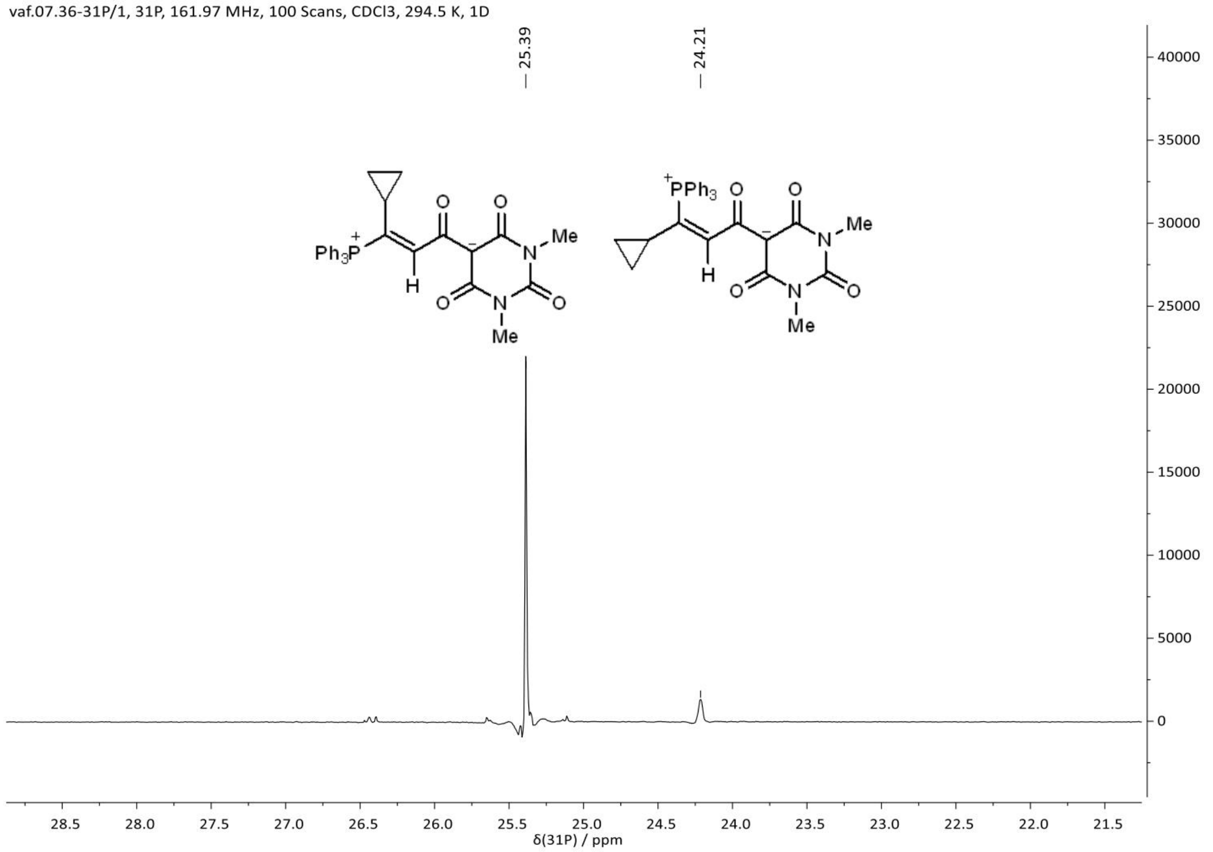
vaf.07.36-31P/1, 31P, 161.97 MHz, 100 Scans, CDCl3, 294.5 K, 1D
25.39
24.21
δ(31P) / ppm

IR

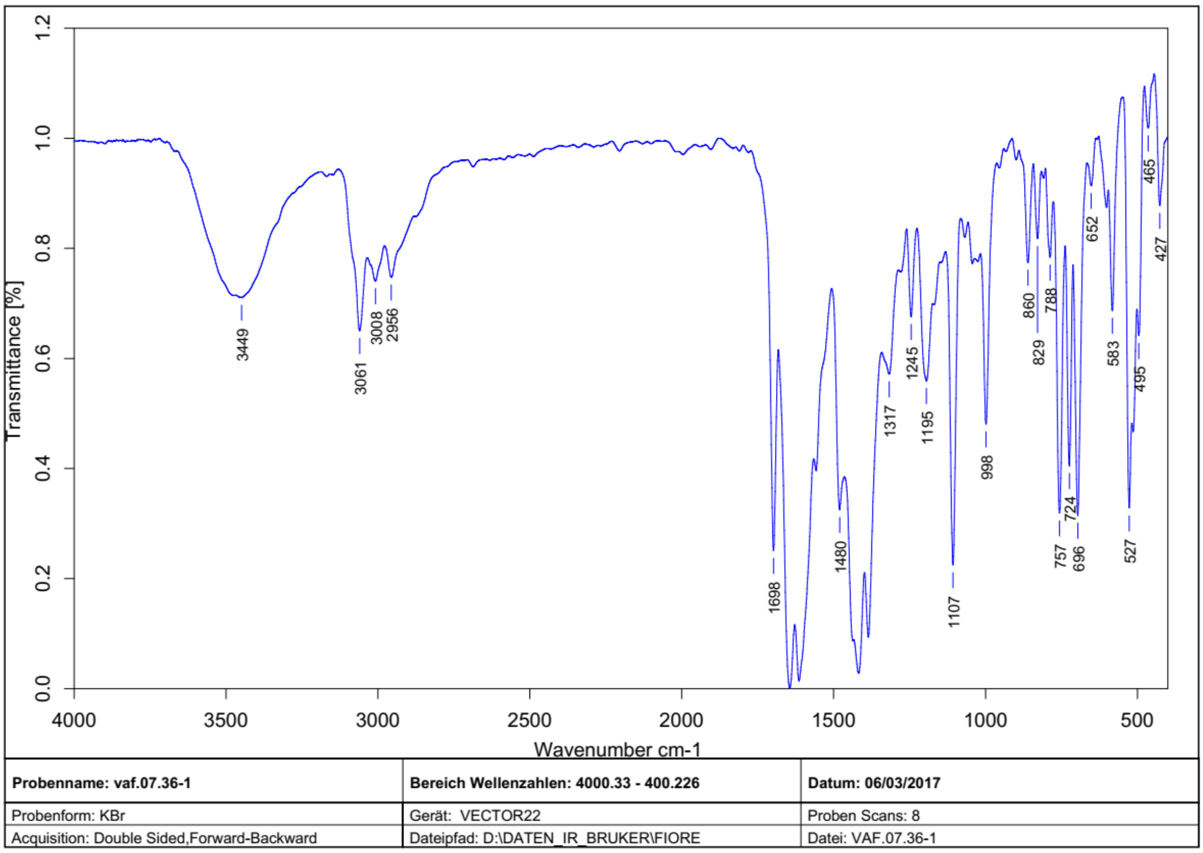

Probenname: vaf.07.36-1	Bereich Wellenzahlen: 4000.33 - 400.226	Datum: 06/03/2017
Probenform: KBr	Gerät: VECTOR22	Proben Scans: 8
Acquisition: Double Sided,Forward-Backward	Dateipfad: D:\DATEN_IR_BRUKER\FIORE	Datei: VAF.07.36-1

4.12. Betaine (*E*)-3l

^{1}H NMR

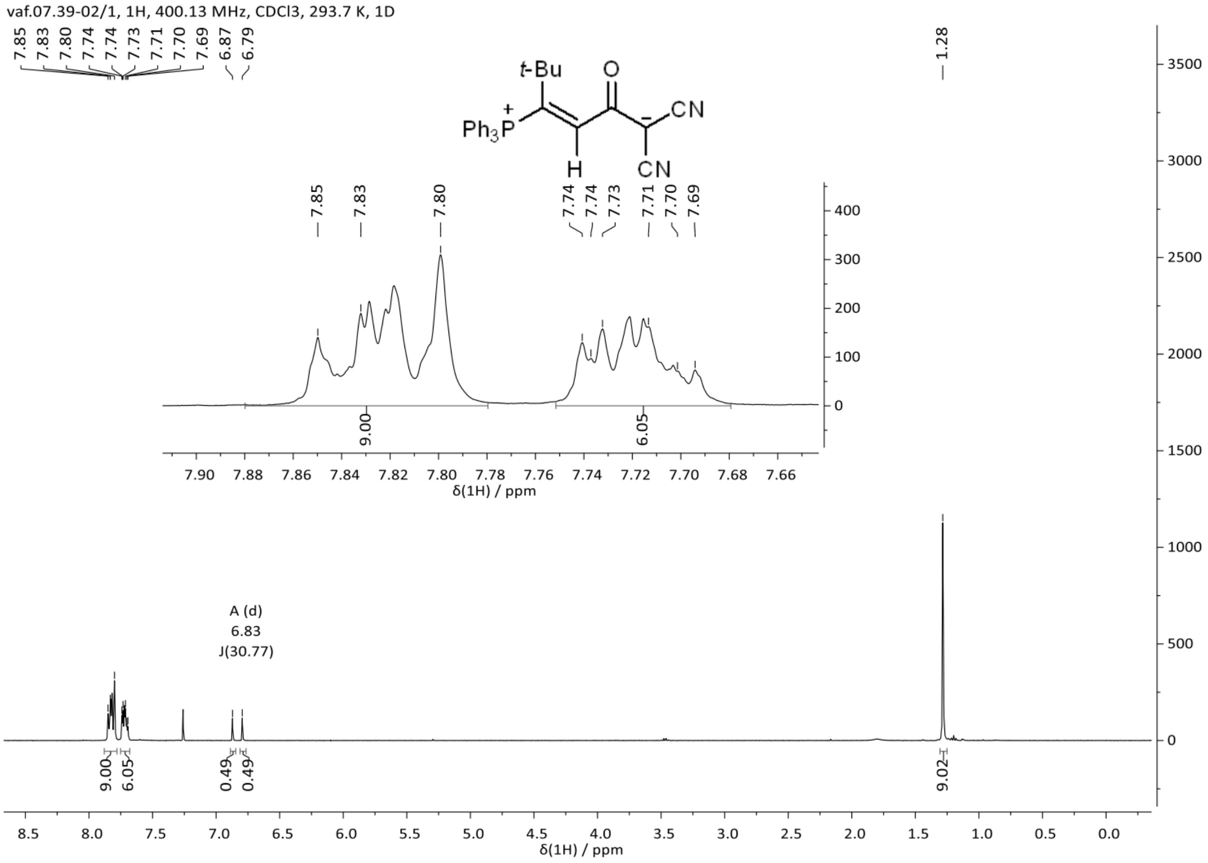

^{13}C NMR

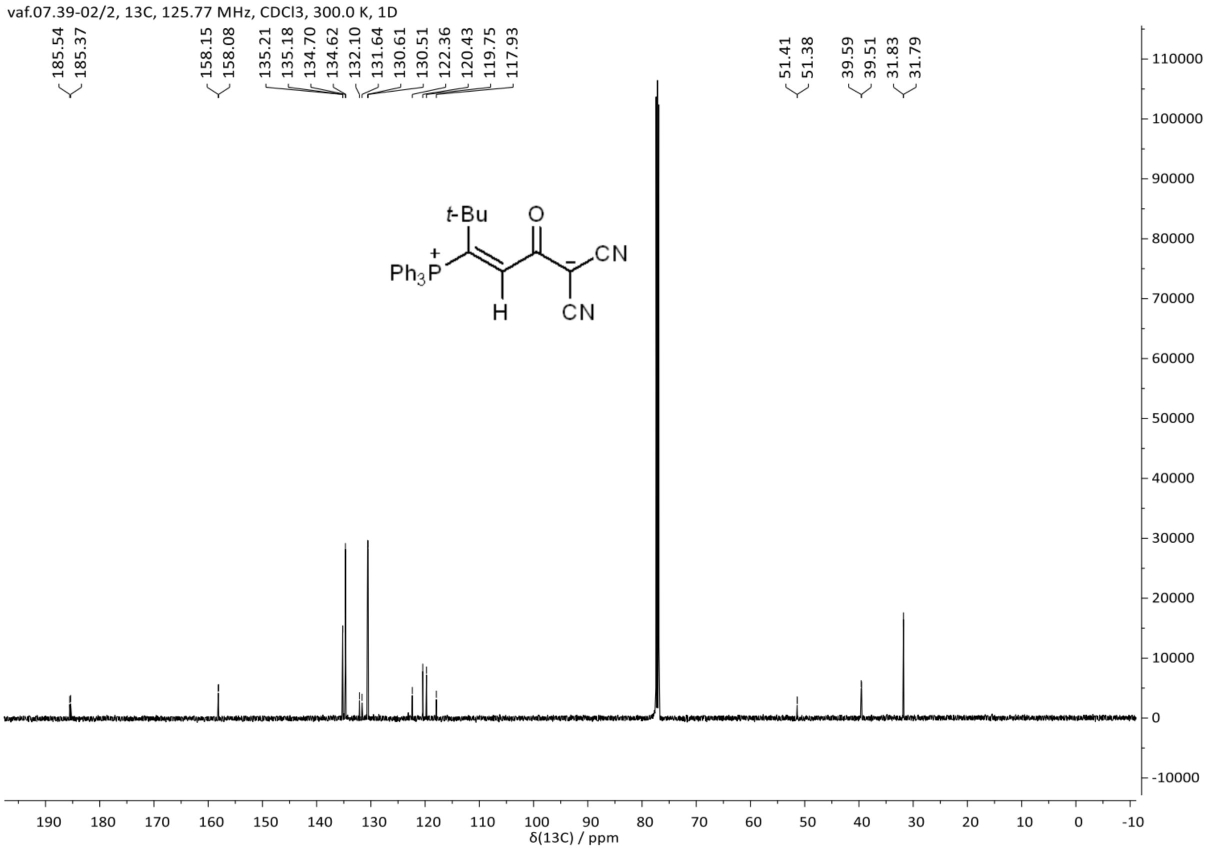

vaf.07.39-02/2, 13C, 125.77 MHz, CDCl3, 300.0 K, 1D
185.54
185.37
158.15
158.08
135.21
135.18
134.70
134.62
132.10
131.64
130.61
130.51
122.36
120.43
119.75
117.93
51.41
51.38
39.59
39.51
31.83
31.79
t-Bu
O
CN
CN
Ph3P
H
δ(13C) / ppm

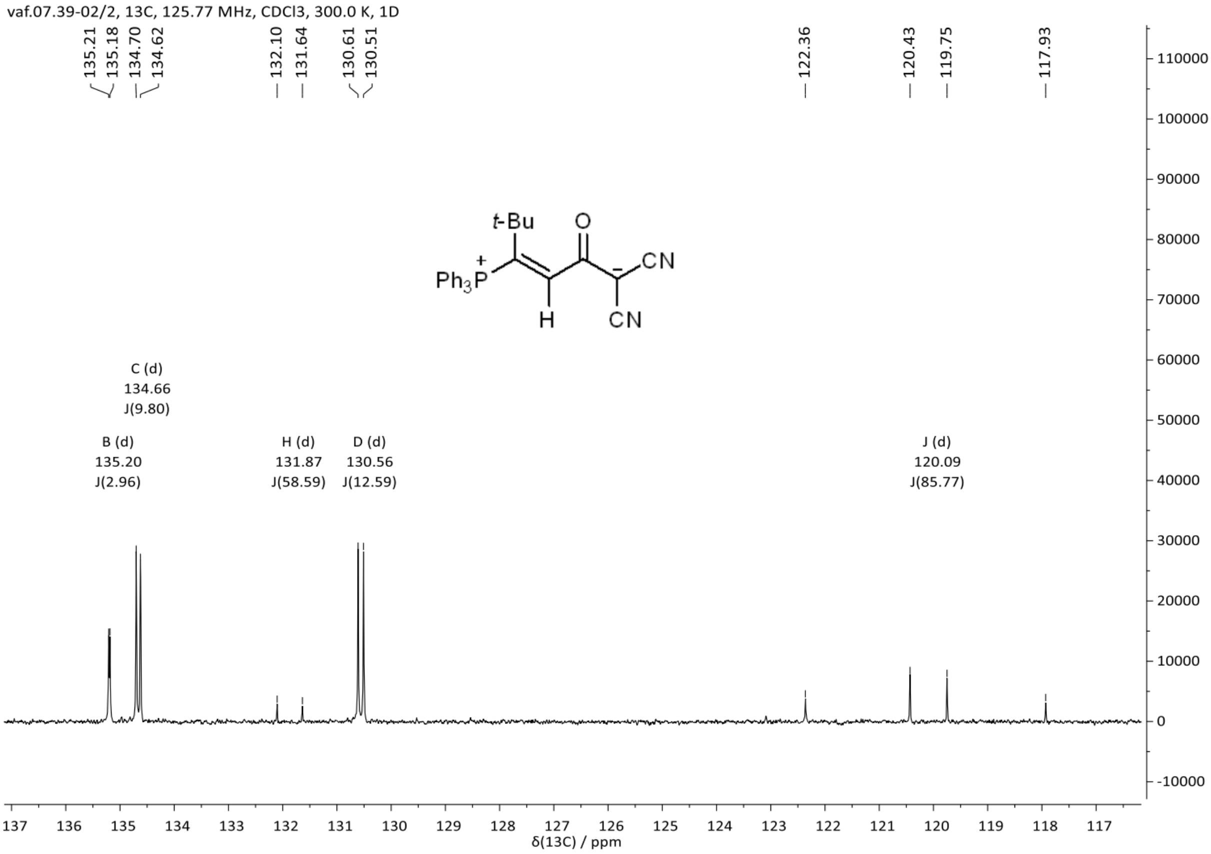

vaf.07.39-02/2, 13C, 125.77 MHz, CDCl3, 300.0 K, 1D
135.21
135.18
134.70
134.62
132.10
131.64
130.61
130.51
122.36
120.43
119.75
117.93
C (d)
134.66
J(9.80)
B (d)
135.20
J(2.96)
H (d)
131.87
J(58.59)
D (d)
130.56
J(12.59)
J (d)
120.09
J(85.77)
δ(13C) / ppm

^{31}P NMR

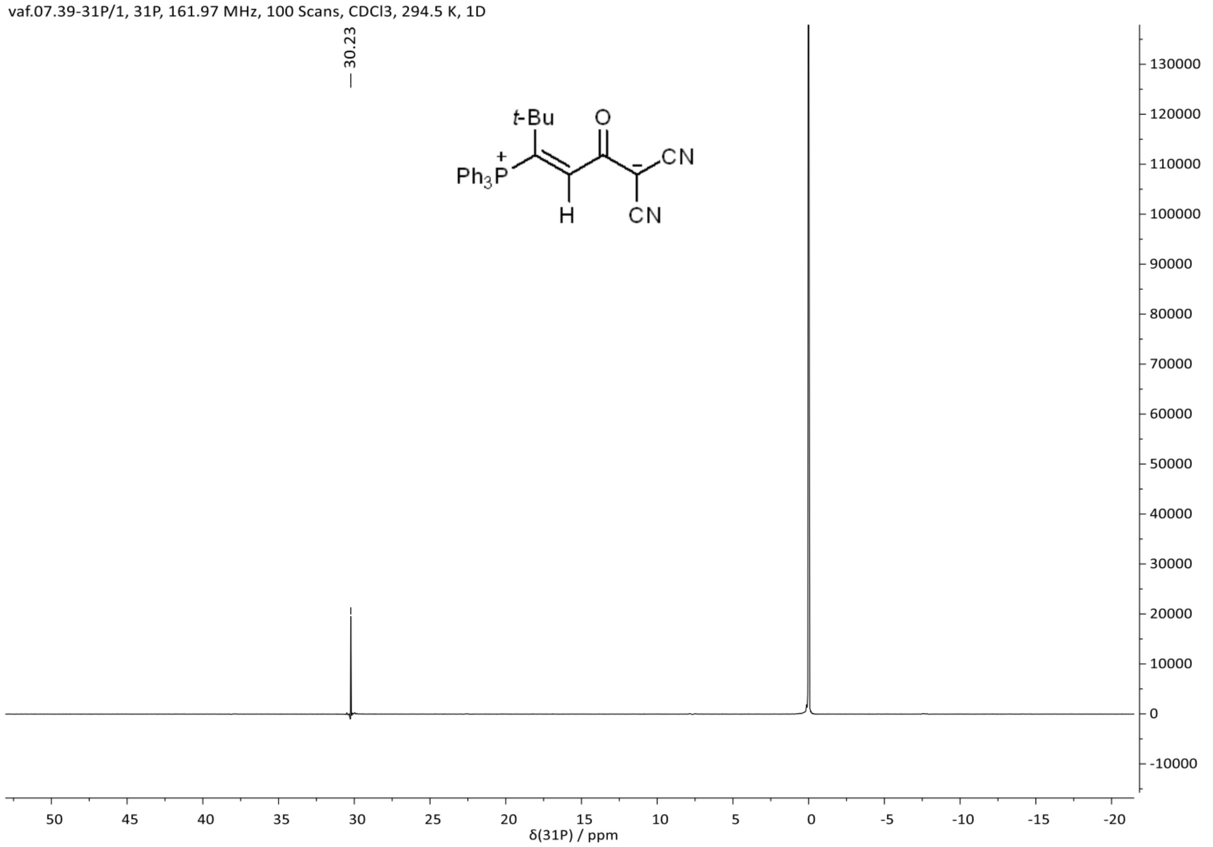

IR

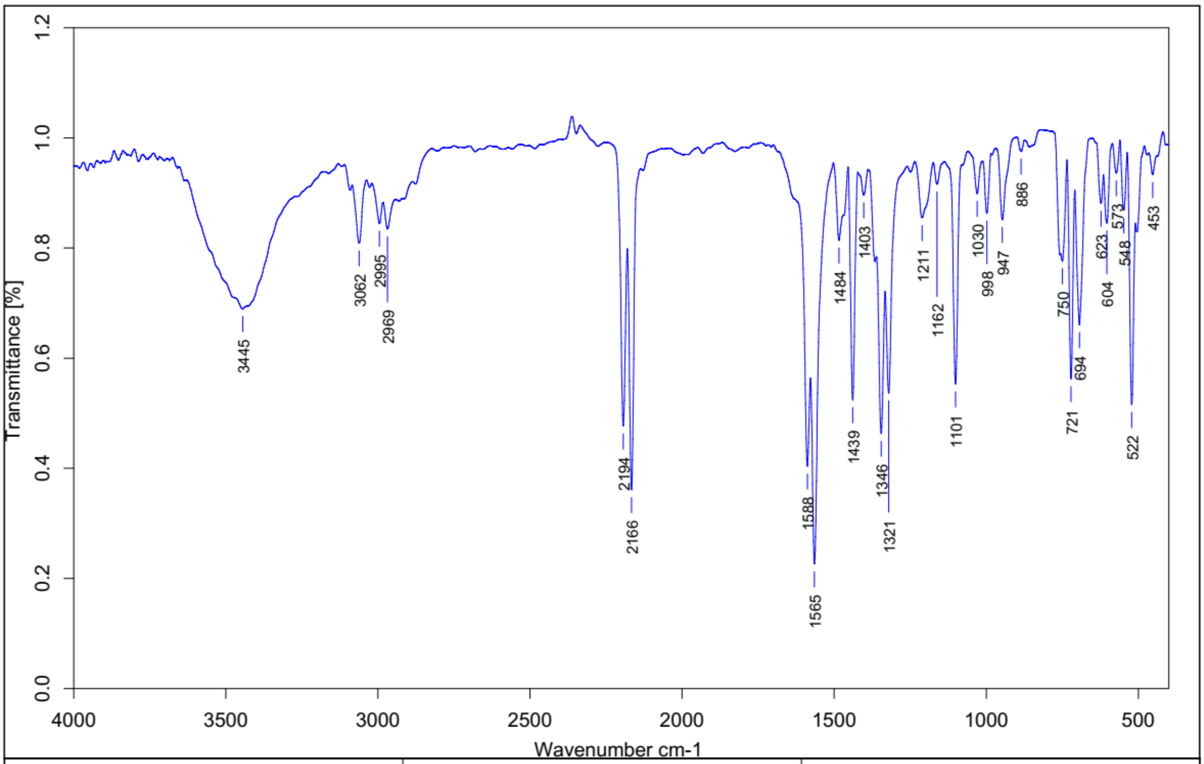

Probenname: vaf.07.39-1	Bereich Wellenzahlen: 4000.33 - 400.226	Datum: 17/03/2017
Probenform: KBr	Gerät: VECTOR22	Proben Scans: 8
Acquisition: Double Sided,Forward-Backward	Dateipfad: D:\DATEN_IR_BRUKER\FIORE	Datei: VAF.07.39-1

4.13. Betaines (*E*)- and (*Z*)-3m

^{1}H NMR

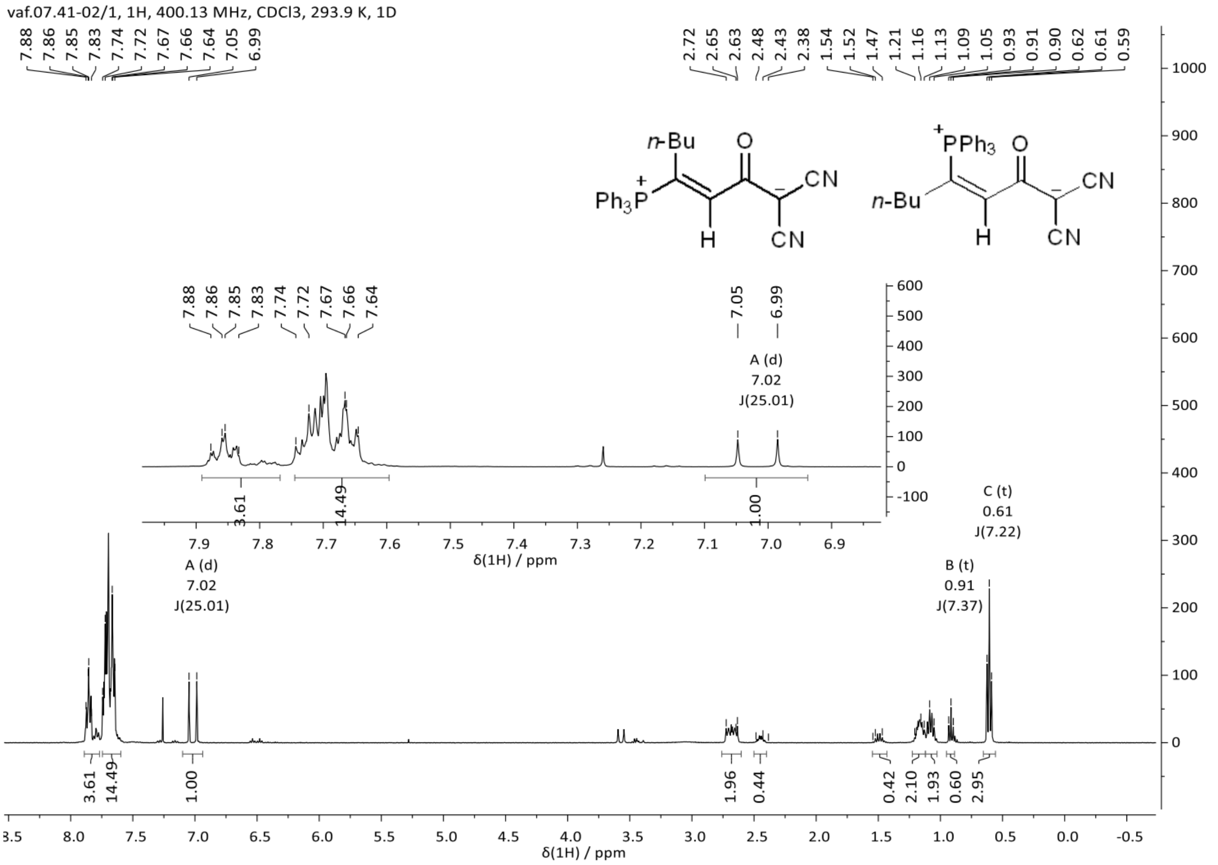

^{13}C NMR

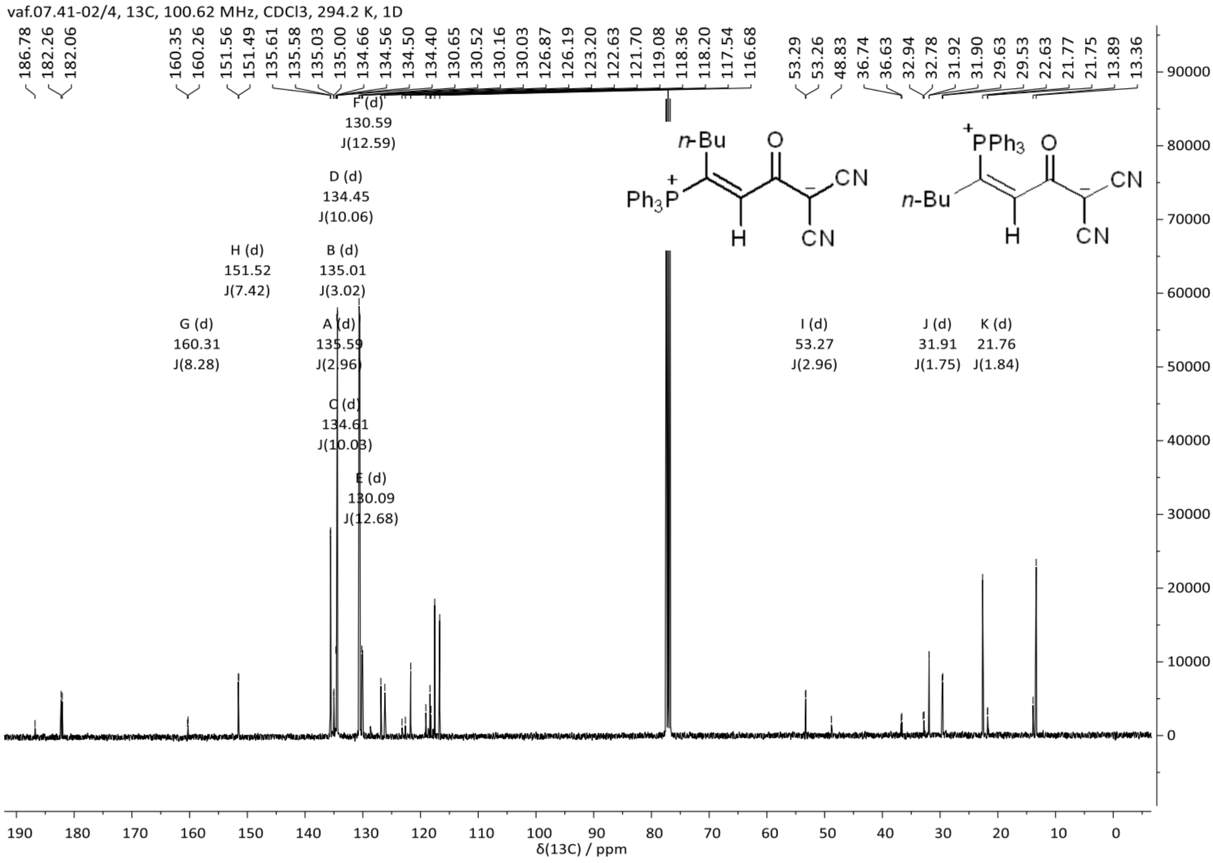

vaf.07.41-02/4, 13C, 100.62 MHz, CDCl3, 294.2 K, 1D
186.78 182.26 182.06 160.35 160.26 151.56 151.49 135.61 135.58 135.03 135.00 134.66 134.56 134.50 134.40 130.65 130.52 130.16 130.03 126.87 126.19 123.20 122.63 121.70 119.08 118.36 118.20 117.54 116.68 53.29 53.26 48.83 36.74 36.63 32.94 32.78 31.92 31.90 29.63 29.53 22.63 21.77 21.75 13.89 13.36
F (d) 130.59 J(12.59)
D (d) 134.45 J(10.06)
H (d) 151.52 J(7.42)
B (d) 135.01 J(3.02)
G (d) 160.31 J(8.28)
A (d) 135.59 J(2.96)
C (d) 134.61 J(10.03)
E (d) 130.09 J(12.68)
I (d) 53.27 J(2.96)
J (d) 31.91 J(1.75)
K (d) 21.76 J(1.84)
n-Bu
Ph3P
CN
H
PPh3
δ(13C) / ppm

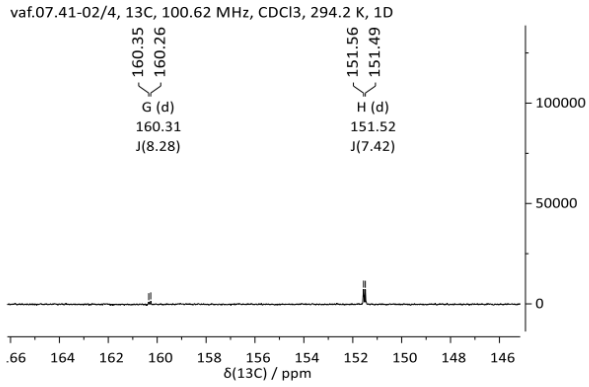

vaf.07.41-02/4, 13C, 100.62 MHz, CDCl3, 294.2 K, 1D
160.35 160.26 151.56 151.49
G (d) 160.31 J(8.28)
H (d) 151.52 J(7.42)
δ(13C) / ppm

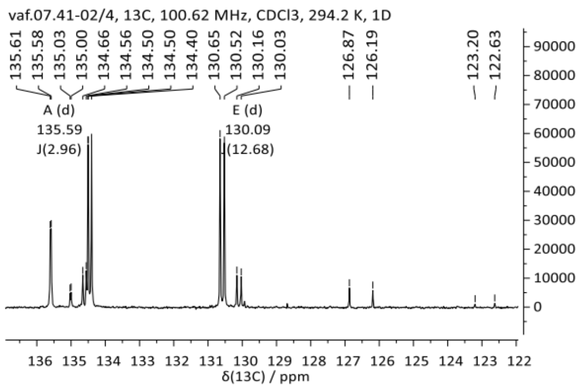

vaf.07.41-02/4, 13C, 100.62 MHz, CDCl3, 294.2 K, 1D
135.61 135.58 135.03 135.00 134.66 134.56 134.50 134.50 134.40 130.65 130.52 130.16 130.03 126.87 126.19 123.20 122.63
A (d) 135.59 J(2.96)
E (d) 130.09 J(12.68)
δ(13C) / ppm

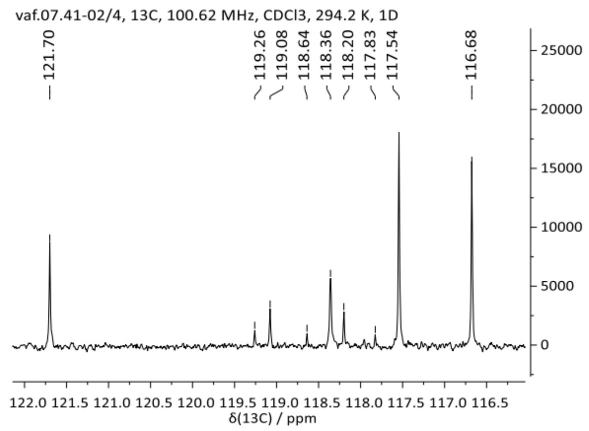

vaf.07.41-02/4, 13C, 100.62 MHz, CDCl3, 294.2 K, 1D
121.70 119.26 119.08 118.64 118.36 118.20 117.83 117.54 116.68
δ(13C) / ppm

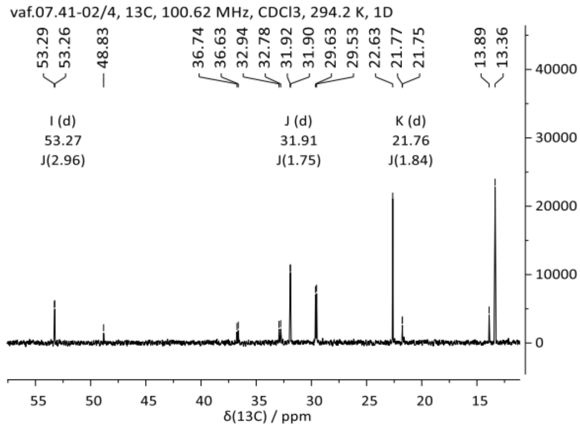

vaf.07.41-02/4, 13C, 100.62 MHz, CDCl3, 294.2 K, 1D
53.29 53.26 48.83 36.74 36.63 32.94 32.78 31.92 31.90 29.63 29.53 22.63 21.77 21.75 13.89 13.36
I (d) 53.27 J(2.96)
J (d) 31.91 J(1.75)
K (d) 21.76 J(1.84)
δ(13C) / ppm

^{31}P NMR

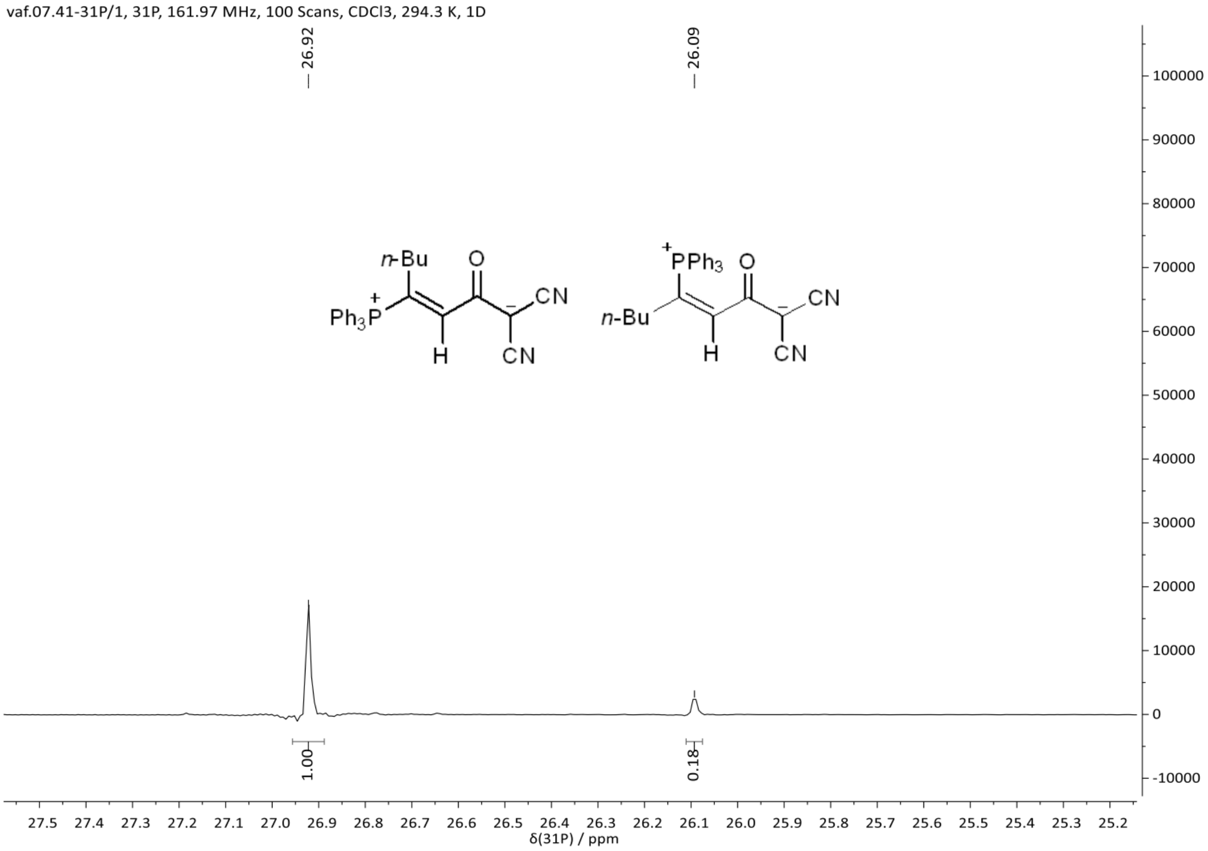

IR

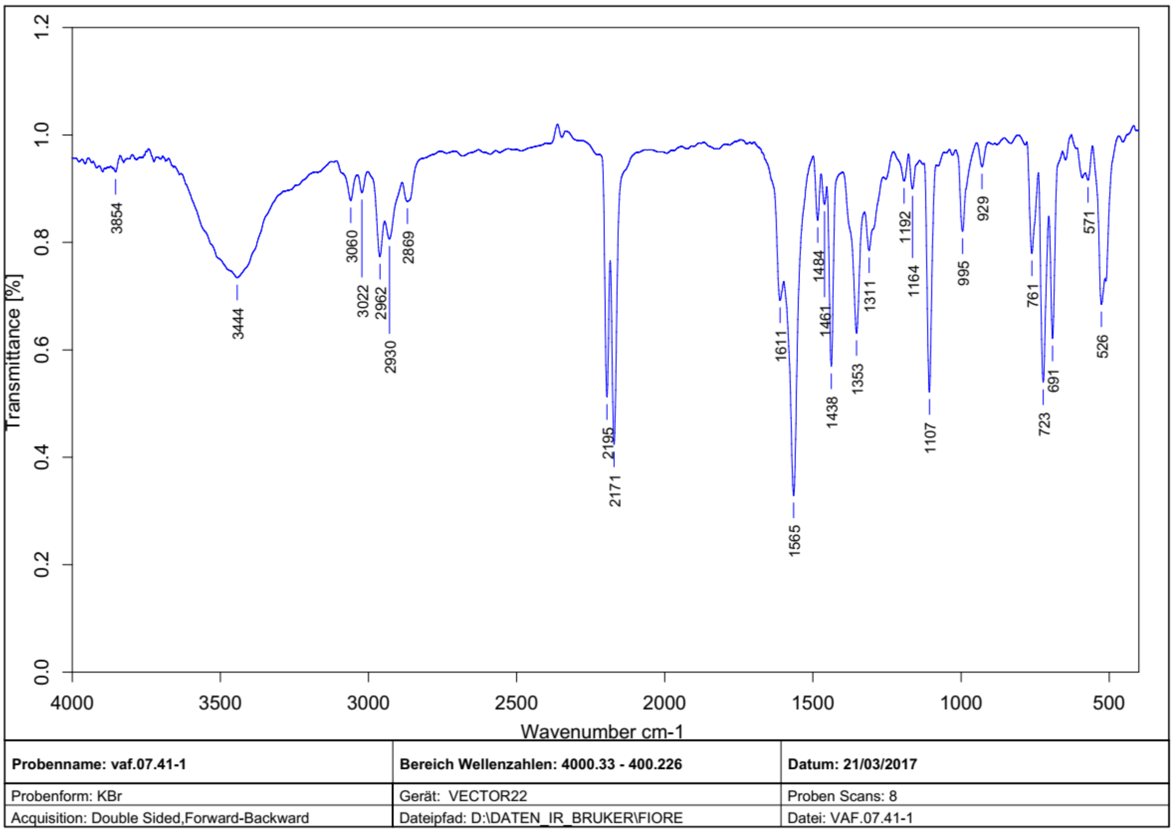

Probenname: vaf.07.41-1	Bereich Wellenzahlen: 4000.33 - 400.226	Datum: 21/03/2017
Probenform: KBr	Gerät: VECTOR22	Proben Scans: 8
Acquisition: Double Sided,Forward-Backward	Dateipfad: D:\DATEN_IR_BRUKER\FIORE	Datei: VAF.07.41-1

***(E)*-3m**

^{1}H NMR

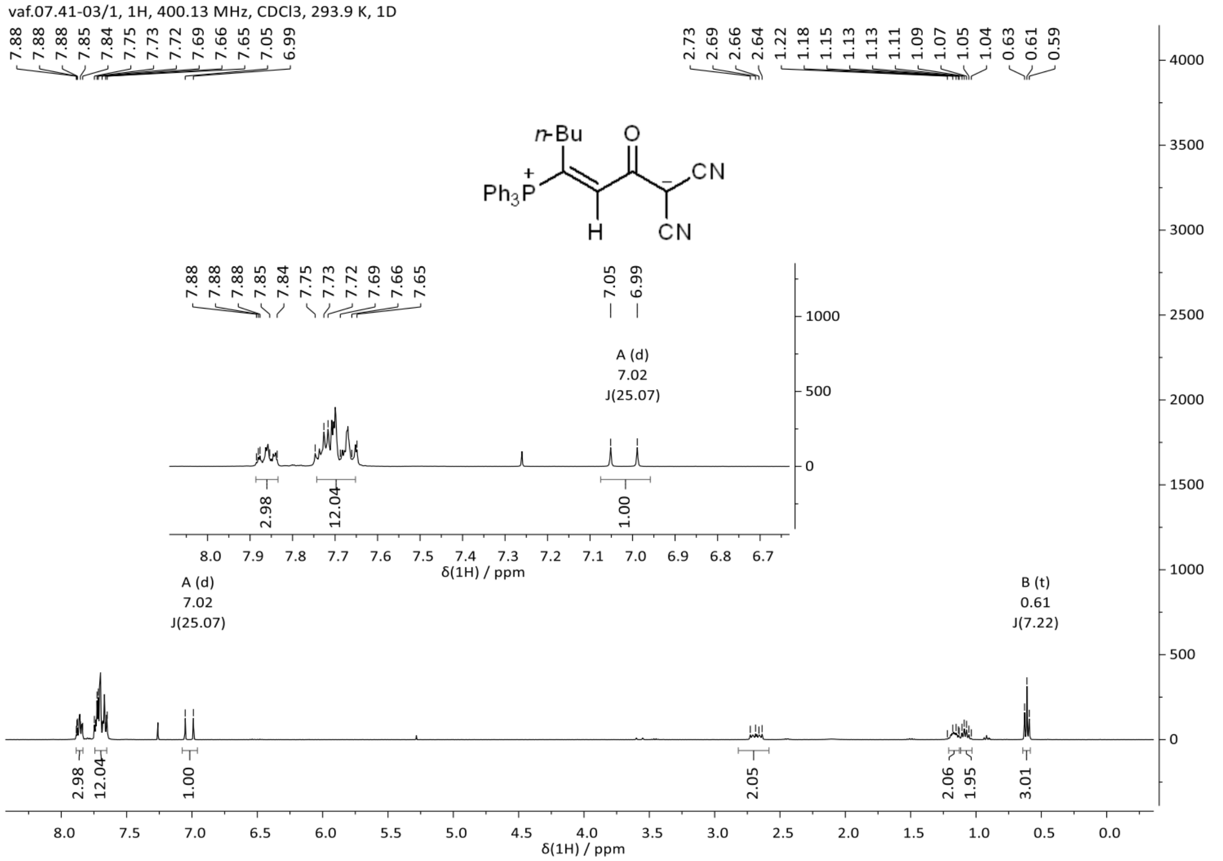

^{13}C NMR

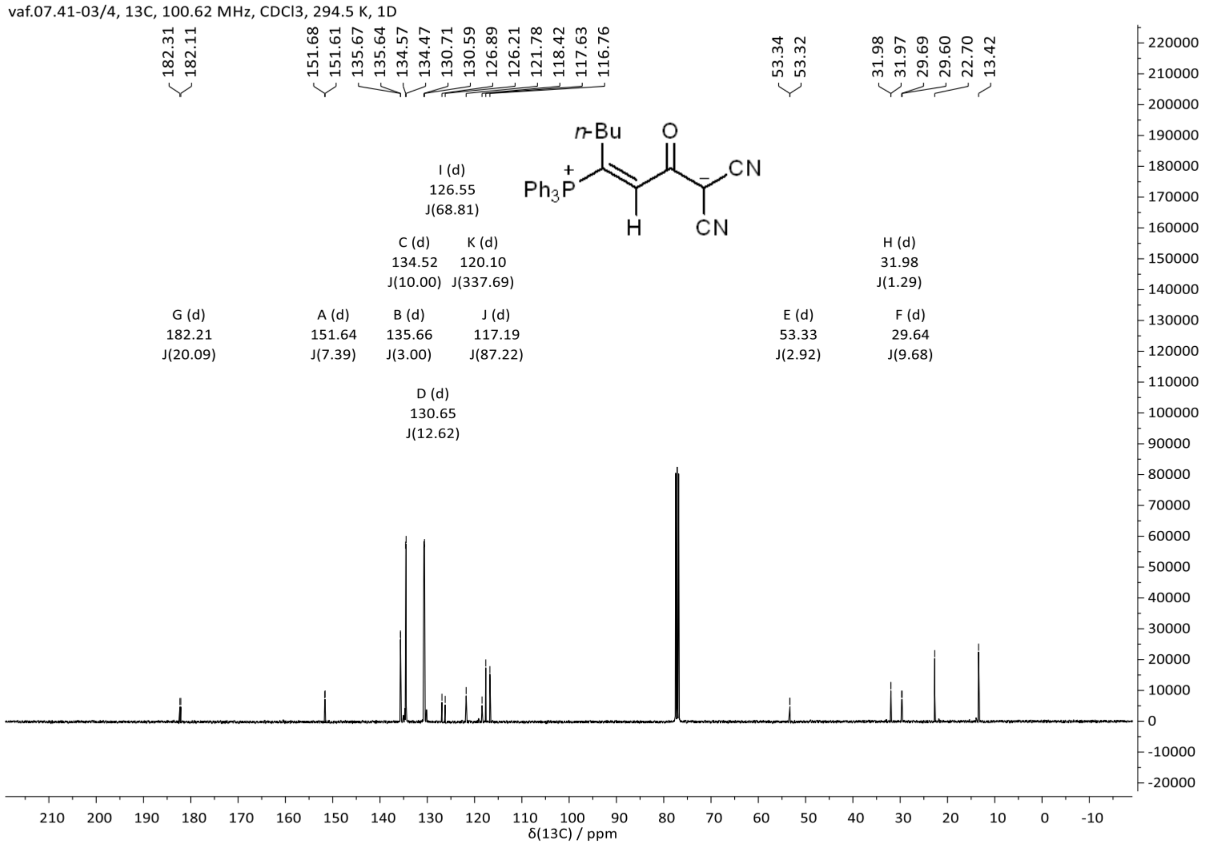

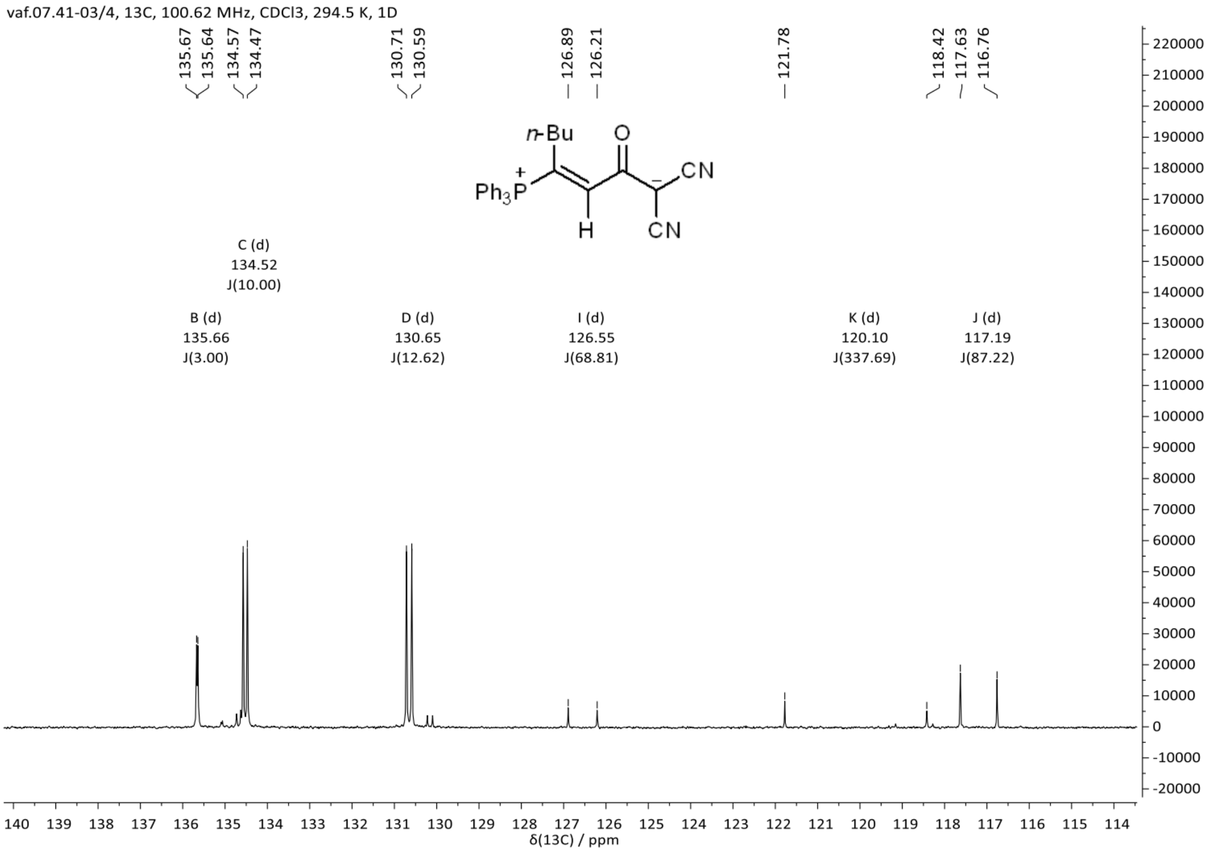
vaf.07.41-03/4, 13C, 100.62 MHz, CDCl3, 294.5 K, 1D
135.67
135.64
134.57
134.47
130.71
130.59
126.89
126.21
121.78
118.42
117.63
116.76
n-Bu
O
CN
Ph3P
H
CN
C (d)
134.52
J(10.00)
B (d)
135.66
J(3.00)
D (d)
130.65
J(12.62)
I (d)
126.55
J(68.81)
K (d)
120.10
J(337.69)
J (d)
117.19
J(87.22)
δ(13C) / ppm

4.14. Betaine (*E*)-3n

^{1}H NMR

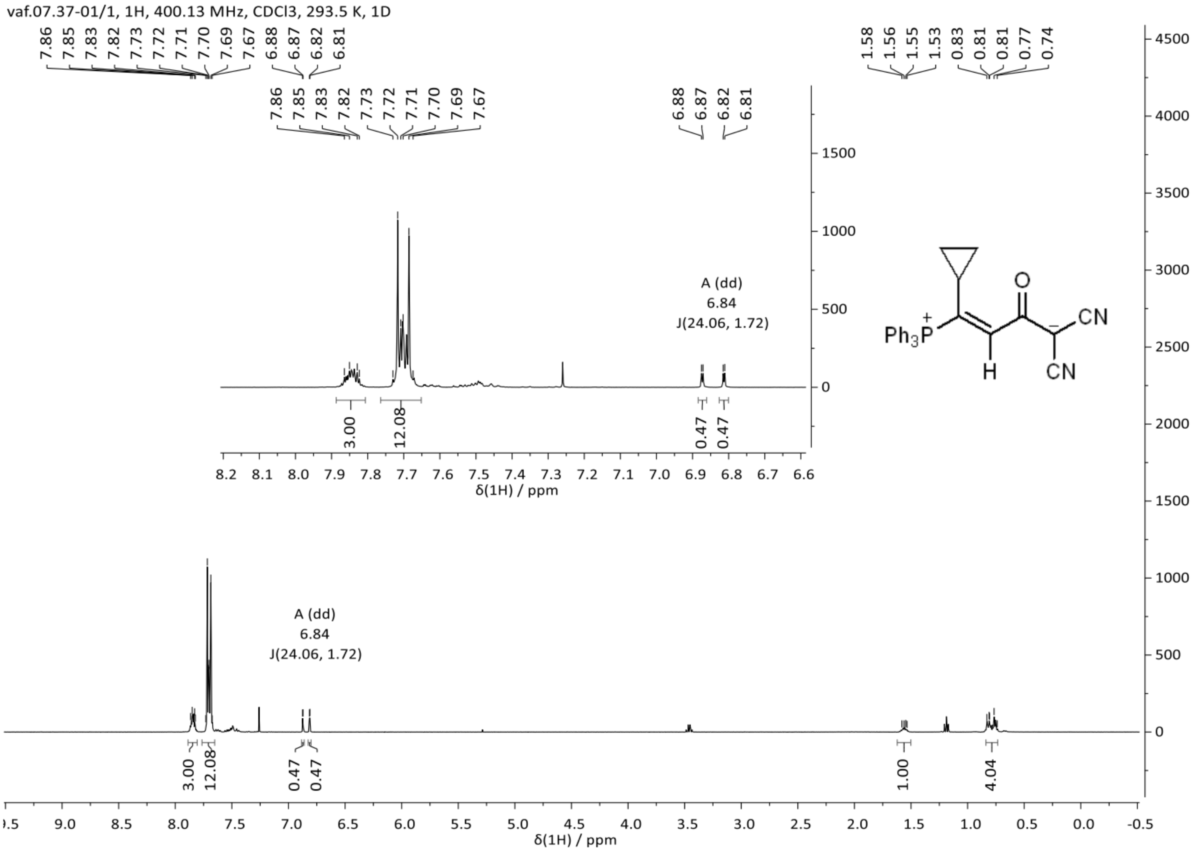

^{13}C NMR

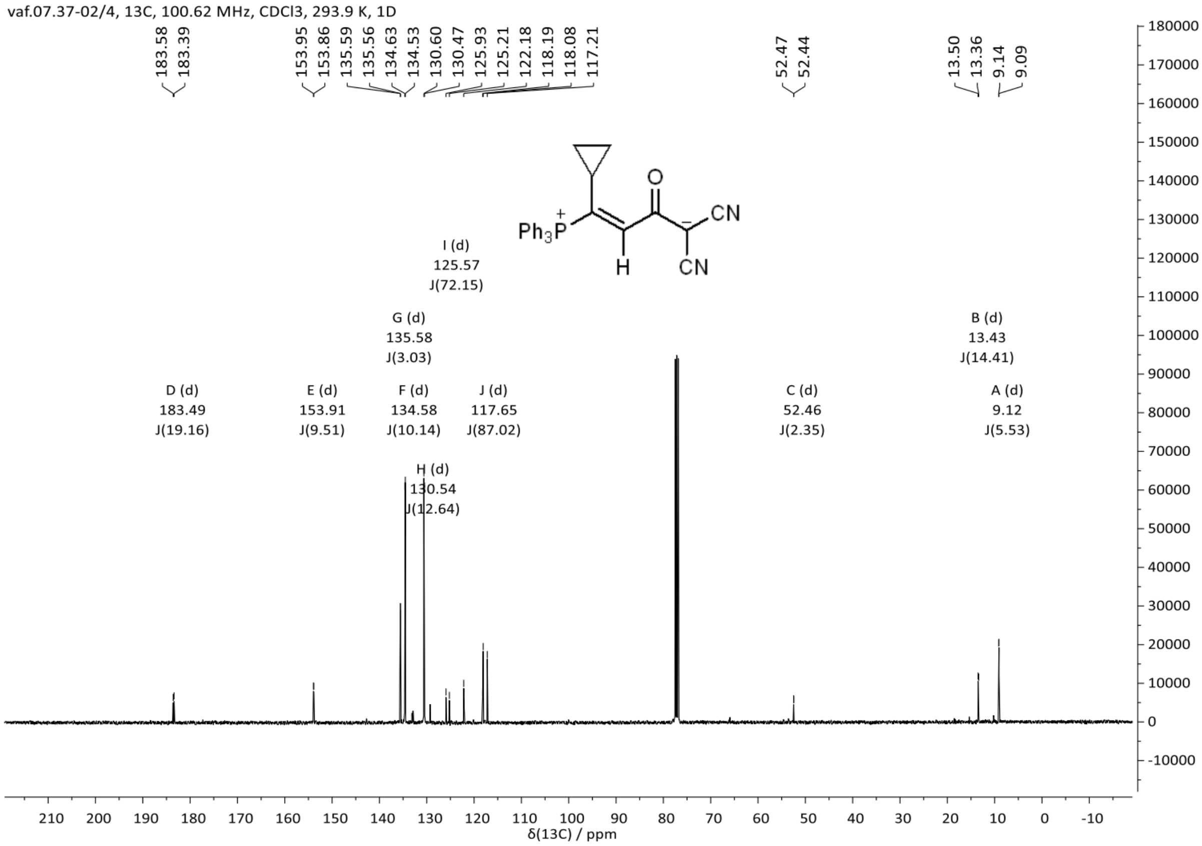

vaf.07.37-02/4, 13C, 100.62 MHz, CDCl3, 293.9 K, 1D
183.58
183.39
153.95
153.86
135.59
135.56
134.63
134.53
130.60
130.47
125.93
125.21
122.18
118.19
118.08
117.21
52.47
52.44
13.50
13.36
9.14
9.09
I (d)
125.57
J(72.15)
G (d)
135.58
J(3.03)
B (d)
13.43
J(14.41)
D (d)
183.49
J(19.16)
E (d)
153.91
J(9.51)
F (d)
134.58
J(10.14)
J (d)
117.65
J(87.02)
C (d)
52.46
J(2.35)
A (d)
9.12
J(5.53)
H (d)
130.54
J(12.64)
δ(13C) / ppm

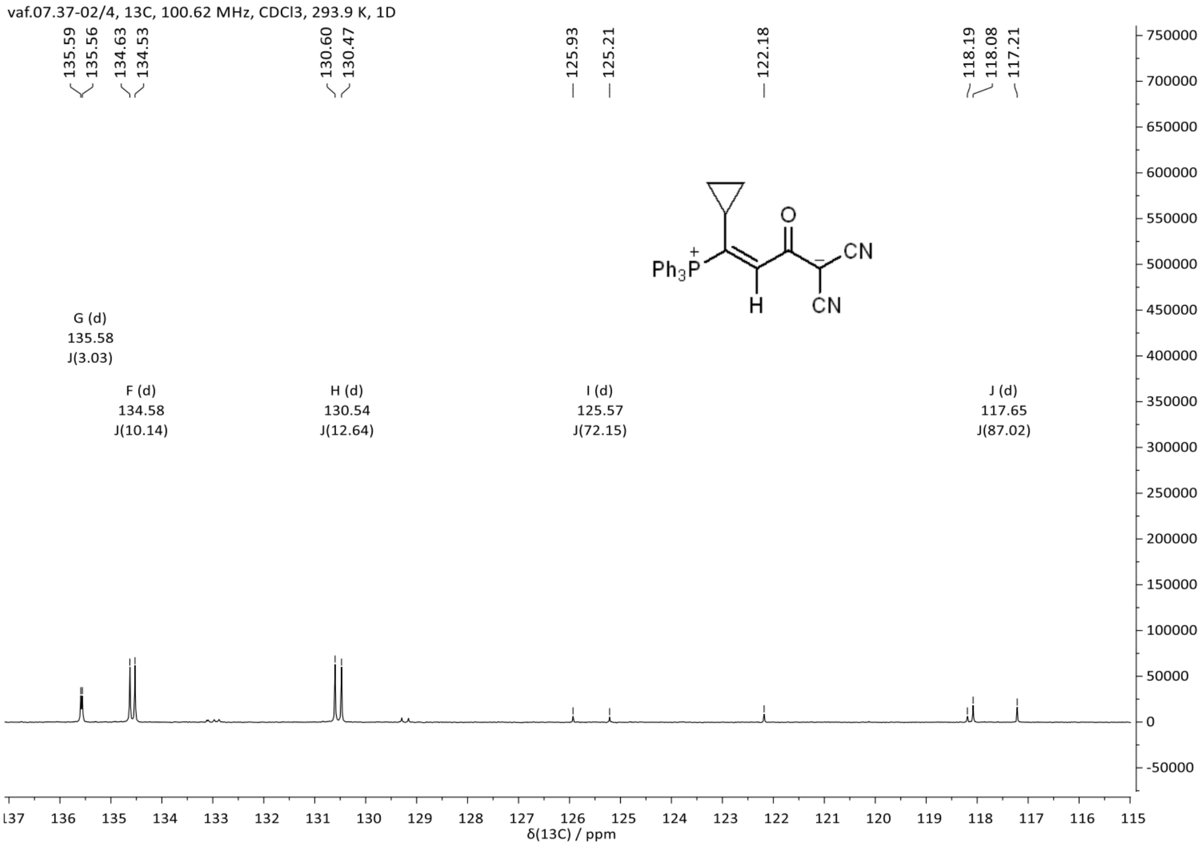

vaf.07.37-02/4, 13C, 100.62 MHz, CDCl3, 293.9 K, 1D
135.59
135.56
134.63
134.53
130.60
130.47
125.93
125.21
122.18
118.19
118.08
117.21
G (d)
135.58
J(3.03)
F (d)
134.58
J(10.14)
H (d)
130.54
J(12.64)
I (d)
125.57
J(72.15)
J (d)
117.65
J(87.02)
δ(13C) / ppm

^{31}P NMR

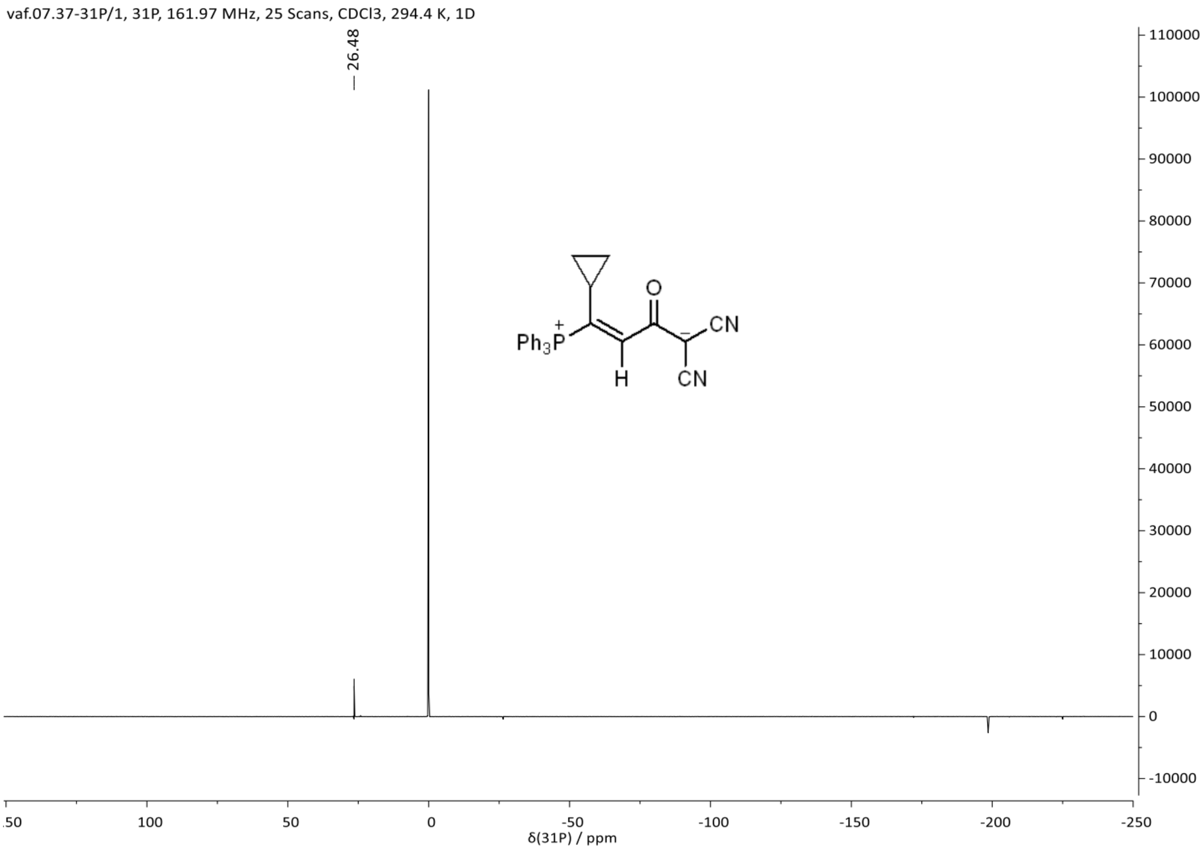

IR

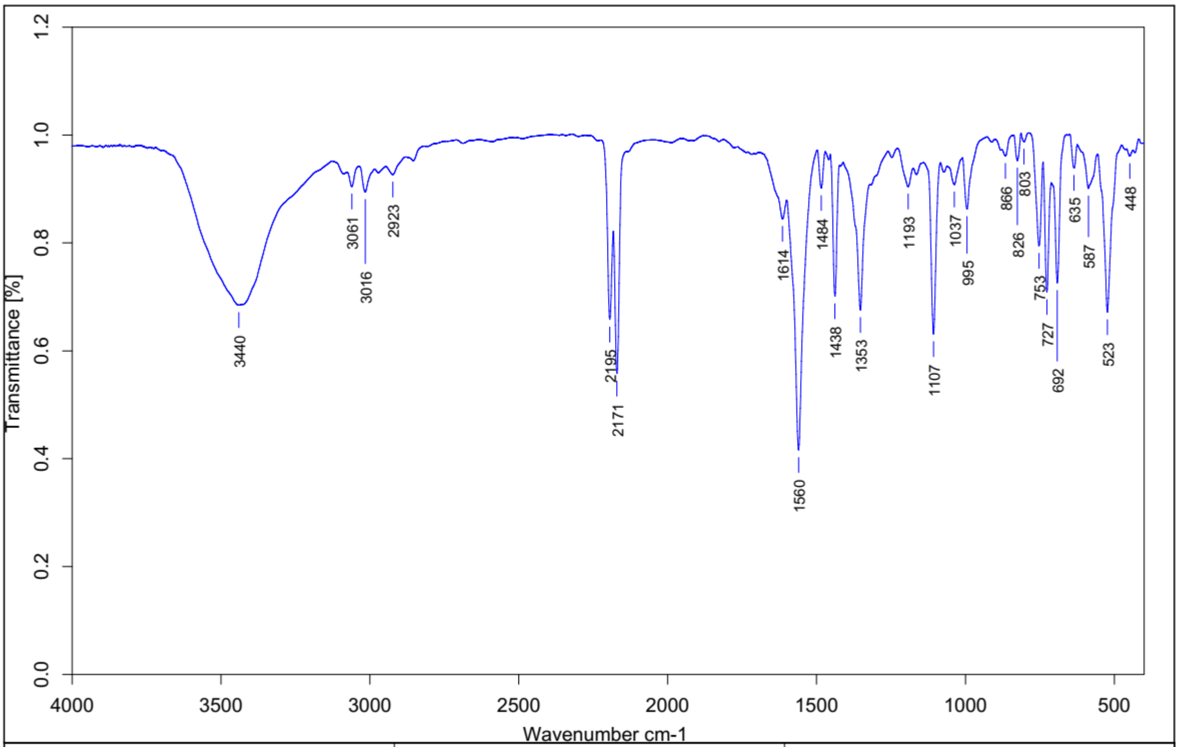

Probenname: vaf.07.37-1	Bereich Wellenzahlen: 4000.33 - 400.226	Datum: 08/03/2017
Probenform: KBr	Gerät: VECTOR22	Proben Scans: 8
Acquisition: Double Sided,Forward-Backward	Dateipfad: D:\DATEN_IR_BRUKER\FIORE	Datei: VAF.07.37-1

4.15. Betaine (*E*)-3o

^{1}H NMR

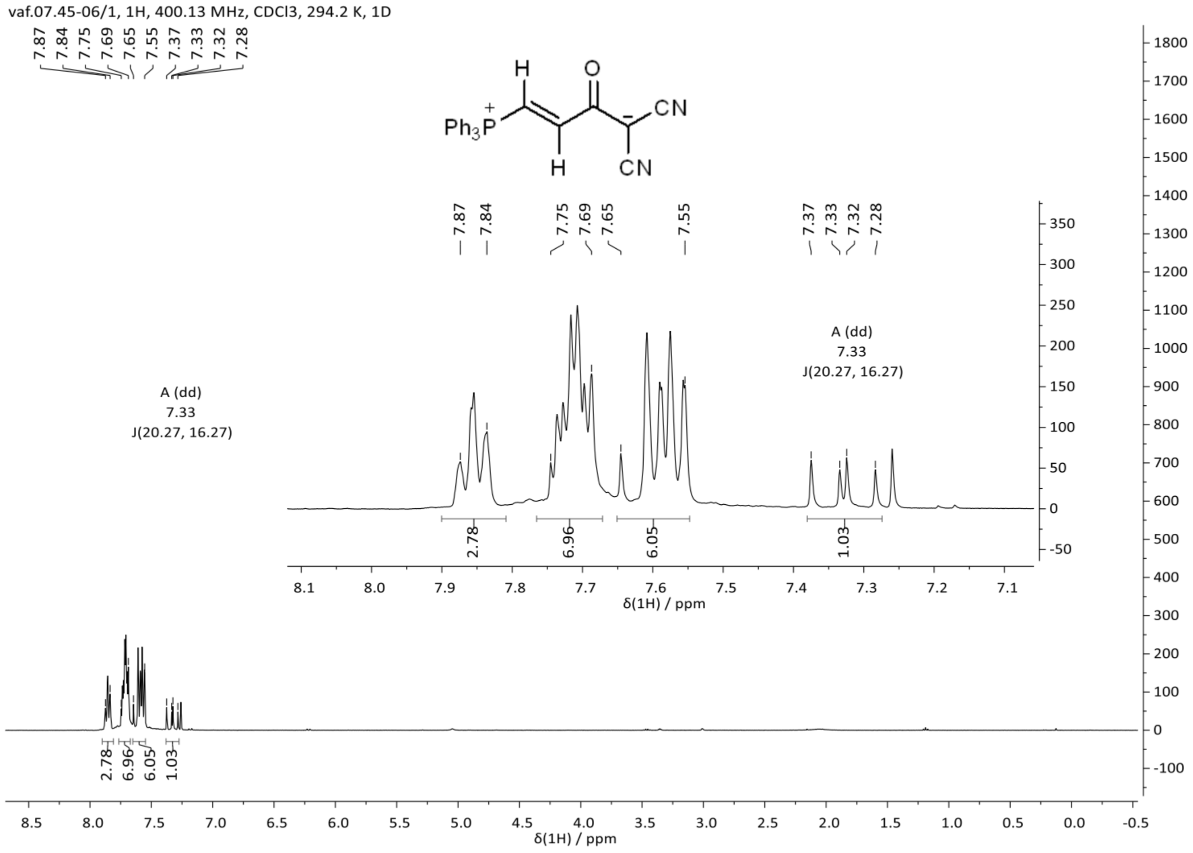

^{13}C NMR

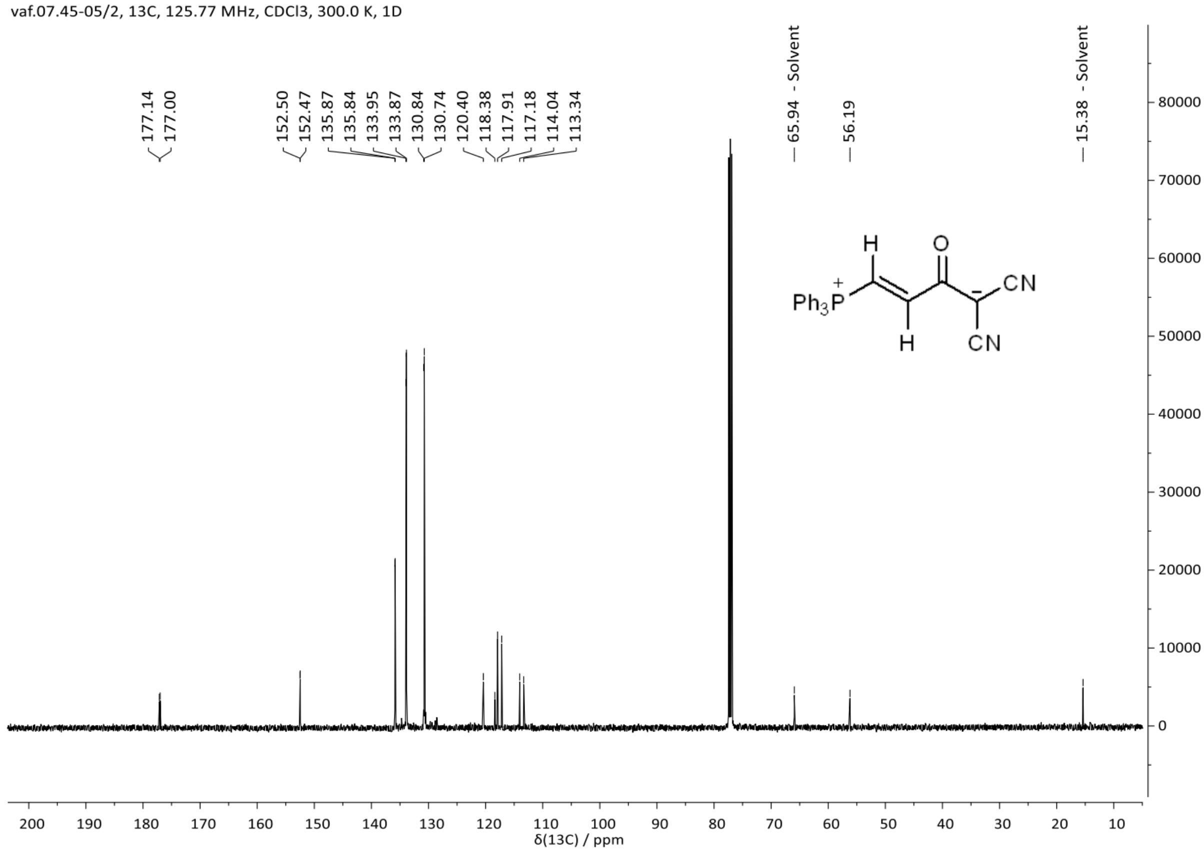

vaf.07.45-05/2, 13C, 125.77 MHz, CDCl3, 300.0 K, 1D
177.14
177.00
152.50
152.47
135.87
135.84
133.95
133.87
130.84
130.74
120.40
118.38
117.91
117.18
114.04
113.34
65.94 - Solvent
56.19
15.38 - Solvent
Ph3P
H
O
CN
δ(13C) / ppm

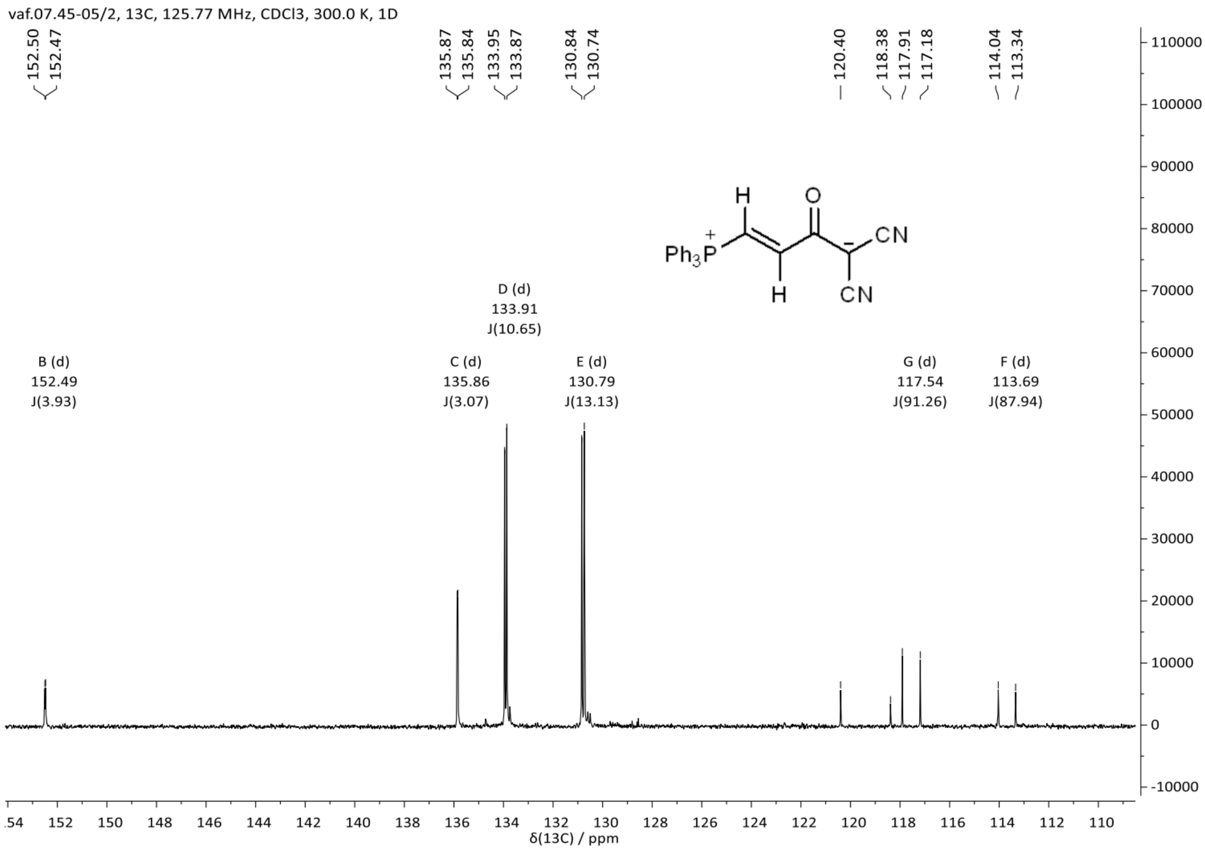

vaf.07.45-05/2, 13C, 125.77 MHz, CDCl3, 300.0 K, 1D
152.50
152.47
135.87
135.84
133.95
133.87
130.84
130.74
120.40
118.38
117.91
117.18
114.04
113.34
D (d)
133.91
J(10.65)
B (d)
152.49
J(3.93)
C (d)
135.86
J(3.07)
E (d)
130.79
J(13.13)
G (d)
117.54
J(91.26)
F (d)
113.69
J(87.94)
Ph3P
H
O
CN
δ(13C) / ppm

^{31}P NMR

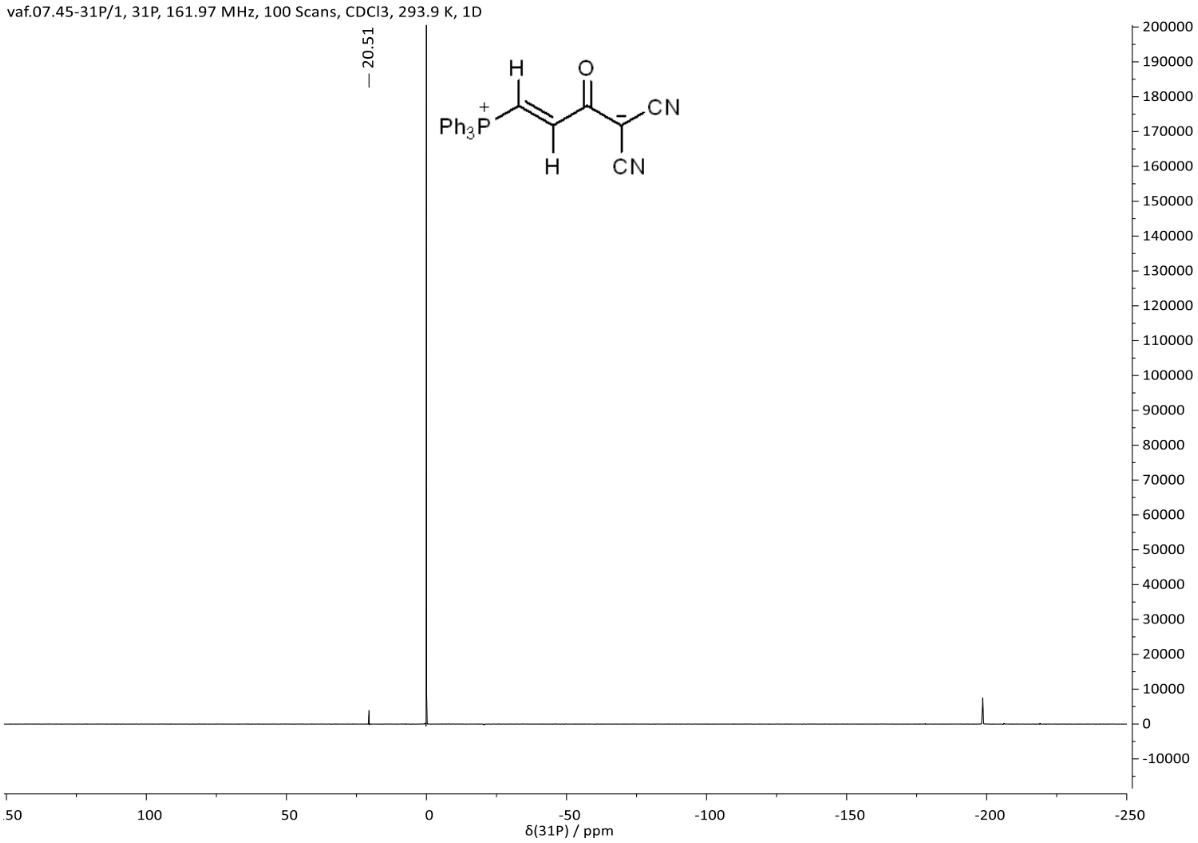

IR

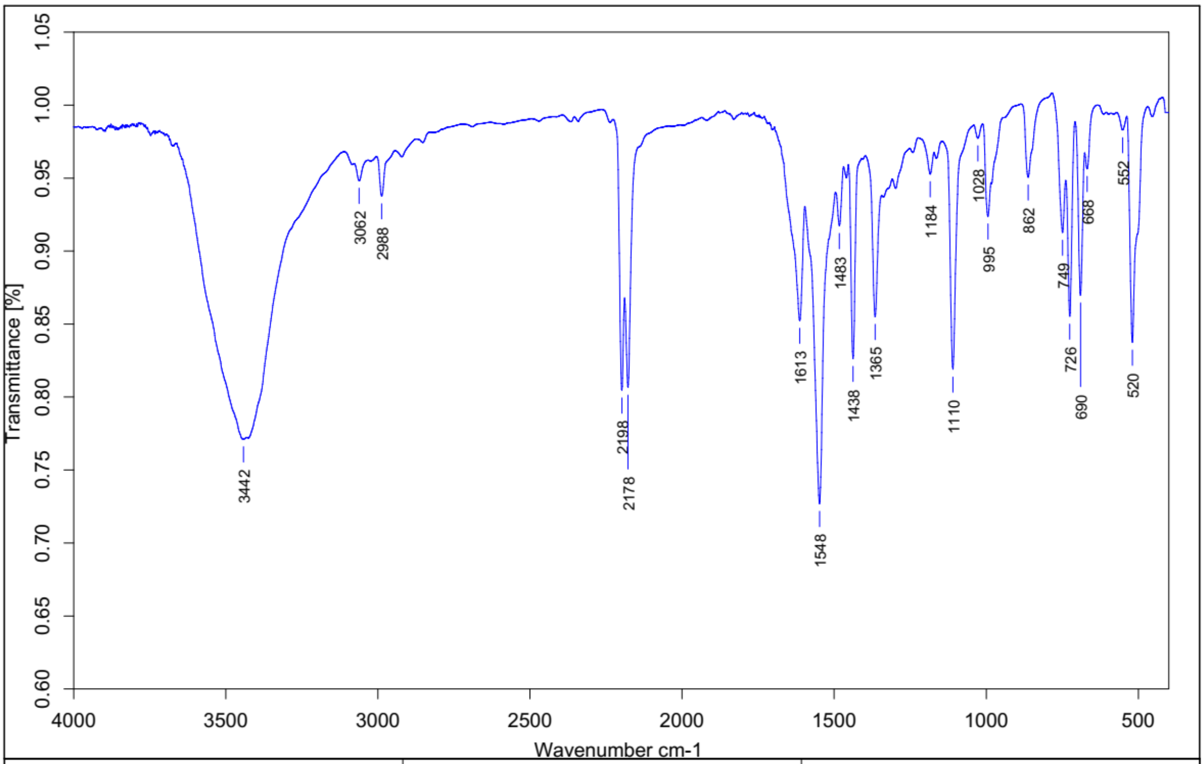

Probenname: vaf.07.45-1	Bereich Wellenzahlen: 4000.33 - 400.226	Datum: 11/05/2017
Probenform: KBr	Gerät: VECTOR22	Proben Scans: 8
Acquisition: Double Sided,Forward-Backward	Dateipfad: D:\DATEN_IR_BRUKER\FIORE	Datei: VAF.07.45-1
